# Supplementary material for: Transcriptional profiling of Hutchinson-Gilford Progeria syndrome fibroblasts reveals deficits in mesenchymal stem cell commitment to differentiation related to early events in endochondral ossification
Source: eLife. 2022 Dec 29;11:e81290. doi: 10.7554/eLife.81290 (PMC9833827; doi:10.7554/eLife.81290)
Supplement: Table 1—source data 1. — Childhood and teenaged patient samples compared to middle aged and older adults. [file elife-81290-table1-data1.zip › Table_1_Source_Data_1/Table_1-Source_Data_1_Description.docx]

Table_1-Source_Data_1

Description: Each compressed set of files includes the output for Metascape analysis. Refer to appropriate metascape_result.xlsx for results of gene ontology enrichment.

| Folder name | Age comparison | Type | Expression | Number of genes |
| --- | --- | --- | --- | --- |
| 0-7 agematched_UP260 | Children | Age Matched | Upregulated | 260 |
| 07_agematched_DOWN63 | Children | Age Matched | Downregulated | 63 |
| 0-7_M_UP574 | Children | Vs middle aged | Upregulated | 574 |
| 0-7_M_DOWN241 | Children | Vs middle aged | Downregulated | 241 |
| 0-7_O_UP_metascape_984 | Children | Vs Old aged | Upregulated | 984 |
| 0-7_O_DOWN_435 | Children | Vs Old aged | Downregulated | 435 |
| Teen_agematched_Down_81 | Teenagers | Age Matched | Downregulated | 81 |
| Teen_agematched_UP_237 | Teenagers | Age Matched | Upregulated | 237 |
| Teen_M_DOWN­ 1138 | Teenagers | Vs middle aged | Downregulated | 1138 |
| Teen_M_UP­_1873 | Teenagers | Vs middle aged | Upregulated | 1873 |
| Teen_O_DOWN_1022 | Teenagers | Vs Old aged | Downregulated | 1022 |
| Teen-O_UP_600 | Teenagers | Vs Old aged | Upregulated | 600 |
