# Supplementary material for: Transcriptional profiling of Hutchinson-Gilford Progeria syndrome fibroblasts reveals deficits in mesenchymal stem cell commitment to differentiation related to early events in endochondral ossification
Source: eLife. 2022 Dec 29;11:e81290. doi: 10.7554/eLife.81290 (PMC9833827; doi:10.7554/eLife.81290)
Supplement: Table 2—source data 1. — Early and late childhood patient samples compared to healthy children, middle aged, and older adults. [file elife-81290-table2-data1.zip › Table_2-Source_Data_1/Table_2-Source_Data_1_Description.docx]

Table_2-Source_Data_1

Description: Each compressed set of files includes the output for Metascape analysis. Refer to appropriate metascape_result.xlsx for results of gene ontology enrichment.

| Folder name | Age comparison | Type | Expression | Number of genes |
| --- | --- | --- | --- | --- |
| 0-3-AM-UP-113.zip | 0-3 years of age | Age Matched | Upregulated | 113 |
| 0-3-AM-DOWN.zip | 0-3 years of age | Age Matched | Downregulated | 25 |
| 0-3-M-DOWN-172.zip | 0-3 years of age | Vs middle aged | Downregulated | 172 |
| 0-3-M-UP-540.zip | 0-3 years of age | Vs middle aged | Upregulated | 540 |
| 0-3-O-DOWN_388.zip | 0-3 years of age | Vs Old aged | Downregulated | 388 |
| 0-3-O-UP_646.zip | 0-3 years of age | Vs Old aged | Upregulated | 646 |
| 4-7-AM-DOWN_102.zip | 4-7 years of age | Age Matched | Downregulated | 102 |
| 4-7-AM-UP-203.zip | 4-7 years of age | Age Matched | Upregulated | 203 |
| 4-7-M-DOWN-184.zip | 4-7 years of age | Vs middle aged | Downregulated | 184 |
| 4-7-M-UP-173.zip | 4-7 years of age | Vs middle aged | Upregulated | 173 |
| 4-7-O_UP_305.zip | 4-7 years of age | Vs Old aged | Upregulated | 305 |
| 4-7-O-DOWN_394.zip | 4-7 years of age | Vs Old aged | Downregulated | 394 |
