# Supplementary material for: Transcriptional profiling of Hutchinson-Gilford Progeria syndrome fibroblasts reveals deficits in mesenchymal stem cell commitment to differentiation related to early events in endochondral ossification
Source: eLife. 2022 Dec 29;11:e81290. doi: 10.7554/eLife.81290 (PMC9833827; doi:10.7554/eLife.81290)
Supplement: Table 1—source data 1. — Childhood and teenaged patient samples compared to middle aged and older adults. [file elife-81290-table1-data1.zip › Table_1_Source_Data_1/0-7 agematched_UP260.zip!/0-7 agematched_UP260/Enrichment_GO/ColorByCluster.pdf]

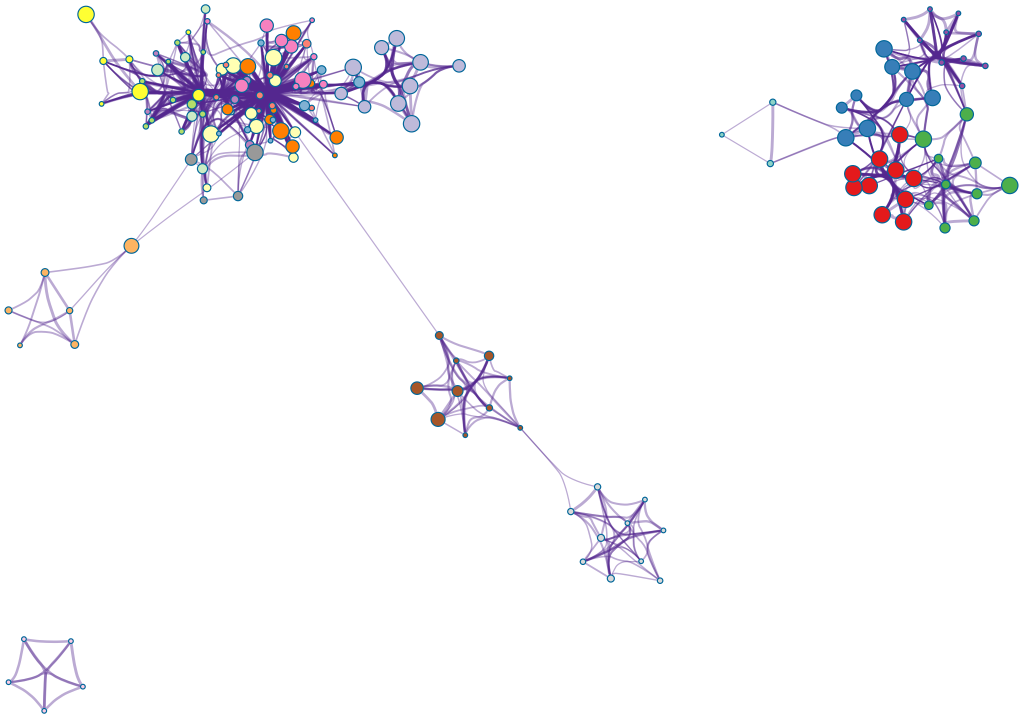

- HDACs deacetylate histones
- negative regulation of gene expression, epigenetic
- regulation of gene silencing
- Formation of Senescence-Associated Heterochromat
- tissue morphogenesis
- response to growth factor
- regulation of small GTPase mediated signal transduc
- sensory organ development
- regulation of cell adhesion
- E3 ubiquitin ligases ubiquitinate target proteins
- blood vessel morphogenesis
- cell morphogenesis involved in differentiation
- outflow tract morphogenesis
- mesenchyme development
- transmembrane receptor protein tyrosine kinase sign
- Hair follicle development: cytodifferentiation - part 3 o
- negative regulation of chemokine-mediated signaling
- Adrenergic signaling in cardiomyocytes
- inner ear development
- epithelial cell proliferation

created by
http://metascape.org
