# Supplementary material for: Transcriptional profiling of Hutchinson-Gilford Progeria syndrome fibroblasts reveals deficits in mesenchymal stem cell commitment to differentiation related to early events in endochondral ossification
Source: eLife. 2022 Dec 29;11:e81290. doi: 10.7554/eLife.81290 (PMC9833827; doi:10.7554/eLife.81290)
Supplement: Table 1—source data 1. — Childhood and teenaged patient samples compared to middle aged and older adults. [file elife-81290-table1-data1.zip › Table_1_Source_Data_1/0-7 agematched_UP260.zip!/0-7 agematched_UP260/Enrichment_heatmap/HeatmapSelectedGOTop100.pdf]

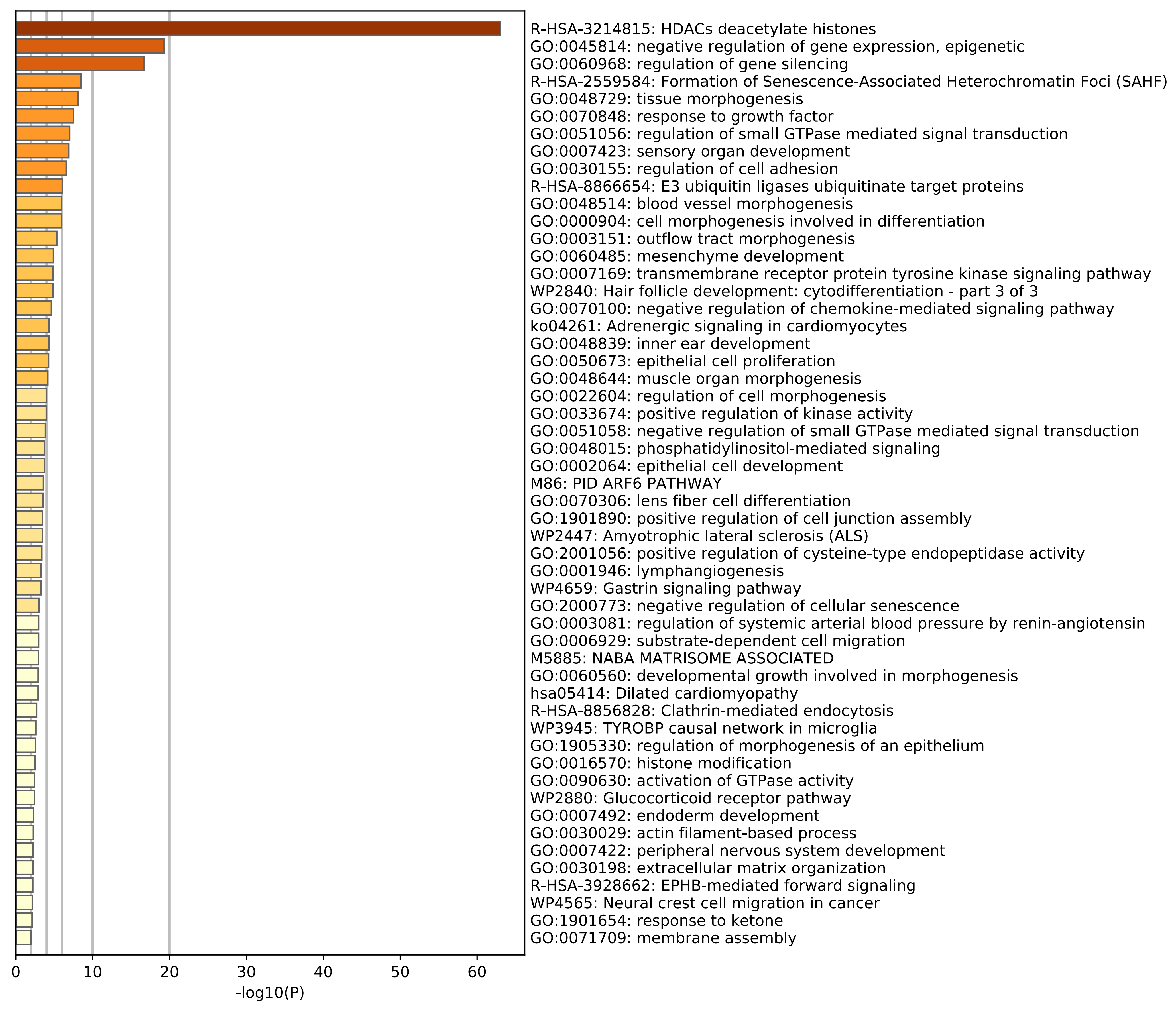
R-HSA-3214815: HDACs deacetylate histones
GO:0045814: negative regulation of gene expression, epigenetic
GO:0060968: regulation of gene silencing
R-HSA-2559584: Formation of Senescence-Associated Heterochromatin Foci (SAHF)
GO:0048729: tissue morphogenesis
GO:0070848: response to growth factor
GO:0051056: regulation of small GTPase mediated signal transduction
GO:0007423: sensory organ development
GO:0030155: regulation of cell adhesion
R-HSA-8866654: E3 ubiquitin ligases ubiquitinate target proteins
GO:0048514: blood vessel morphogenesis
GO:0000904: cell morphogenesis involved in differentiation
GO:0003151: outflow tract morphogenesis
GO:0060485: mesenchyme development
GO:0007169: transmembrane receptor protein tyrosine kinase signaling pathway
WP2840: Hair follicle development: cytodifferentiation - part 3 of 3
GO:0070100: negative regulation of chemokine-mediated signaling pathway
ko04261: Adrenergic signaling in cardiomyocytes
GO:0048839: inner ear development
GO:0050673: epithelial cell proliferation
GO:0048644: muscle organ morphogenesis
GO:0022604: regulation of cell morphogenesis
GO:0033674: positive regulation of kinase activity
GO:0051058: negative regulation of small GTPase mediated signal transduction
GO:0048015: phosphatidylinositol-mediated signaling
GO:0002064: epithelial cell development
M86: PID ARF6 PATHWAY
GO:0070306: lens fiber cell differentiation
GO:1901890: positive regulation of cell junction assembly
WP2447: Amyotrophic lateral sclerosis (ALS)
GO:2001056: positive regulation of cysteine-type endopeptidase activity
GO:0001946: lymphangiogenesis
WP4659: Gastrin signaling pathway
GO:2000773: negative regulation of cellular senescence
GO:0003081: regulation of systemic arterial blood pressure by renin-angiotensin
GO:0006929: substrate-dependent cell migration
M5885: NABA MATRISOME ASSOCIATED
GO:0060560: developmental growth involved in morphogenesis
hsa05414: Dilated cardiomyopathy
R-HSA-8856828: Clathrin-mediated endocytosis
WP3945: TYROBP causal network in microglia
GO:1905330: regulation of morphogenesis of an epithelium
GO:0016570: histone modification
GO:0090630: activation of GTPase activity
WP2880: Glucocorticoid receptor pathway
GO:0007492: endoderm development
GO:0030029: actin filament-based process
GO:0007422: peripheral nervous system development
GO:0030198: extracellular matrix organization
R-HSA-3928662: EPHB-mediated forward signaling
WP4565: Neural crest cell migration in cancer
GO:1901654: response to ketone
GO:0071709: membrane assembly
0
10
20
30
40
50
60
-log10(P)
