# Supplementary material for: Transcriptional profiling of Hutchinson-Gilford Progeria syndrome fibroblasts reveals deficits in mesenchymal stem cell commitment to differentiation related to early events in endochondral ossification
Source: eLife. 2022 Dec 29;11:e81290. doi: 10.7554/eLife.81290 (PMC9833827; doi:10.7554/eLife.81290)
Supplement: Table 1—source data 1. — Childhood and teenaged patient samples compared to middle aged and older adults. [file elife-81290-table1-data1.zip › Table_1_Source_Data_1/0-7 agematched_UP260.zip!/0-7 agematched_UP260/Enrichment_PPI/MyList_MCODE_ALL_PPIColorByCluster.pdf]

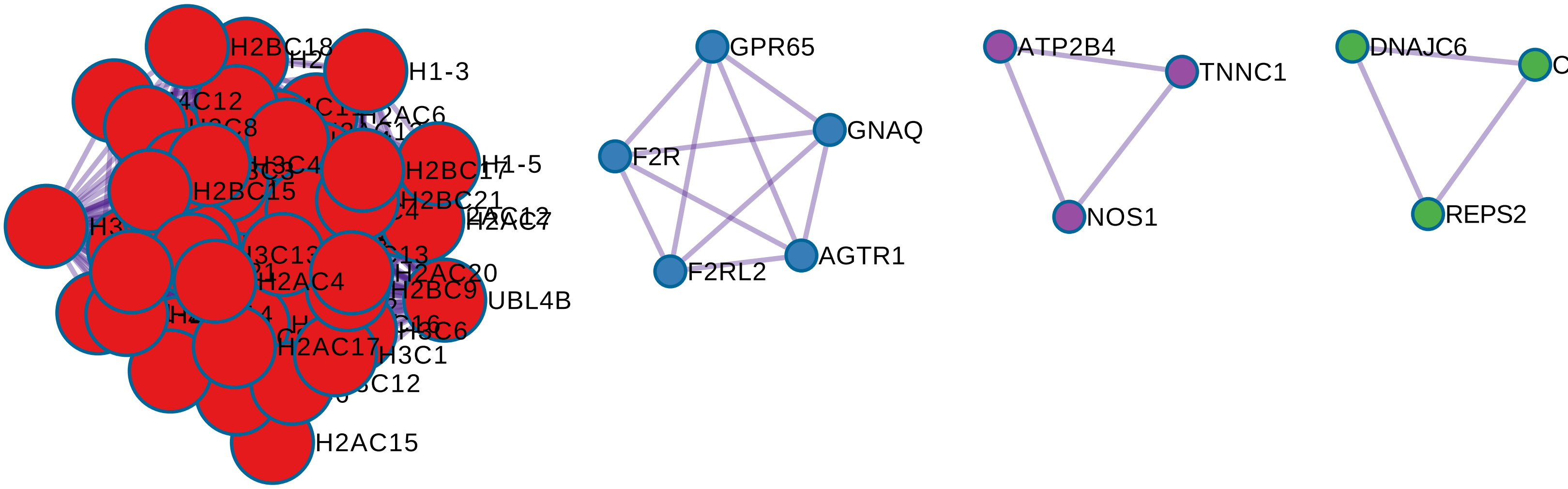
H2BC18
H1-3
H2AC6
H1-5
H2BC17
H3C4
H2BC15
H2BC21
H2AC7
H2AC20
H2AC4
H2BC9
UBL4B
H3C6
H3C1
H2AC17
H2AC15
GPR65
GNAQ
F2R
AGTR1
F2RL2
ATP2B4
TNNC1
NOS1
DNAJC6
REPS2

MCODE1
MCODE2
MCODE3
MCODE4

created by
http://metascape.org
