# Supplementary material for: Transcriptional profiling of Hutchinson-Gilford Progeria syndrome fibroblasts reveals deficits in mesenchymal stem cell commitment to differentiation related to early events in endochondral ossification
Source: eLife. 2022 Dec 29;11:e81290. doi: 10.7554/eLife.81290 (PMC9833827; doi:10.7554/eLife.81290)
Supplement: Table 1—source data 1. — Childhood and teenaged patient samples compared to middle aged and older adults. [file elife-81290-table1-data1.zip › Table_1_Source_Data_1/0-7 agematched_UP260.zip!/0-7 agematched_UP260/Enrichment_QC/HeatmapSelectedGO_COVID.pdf]

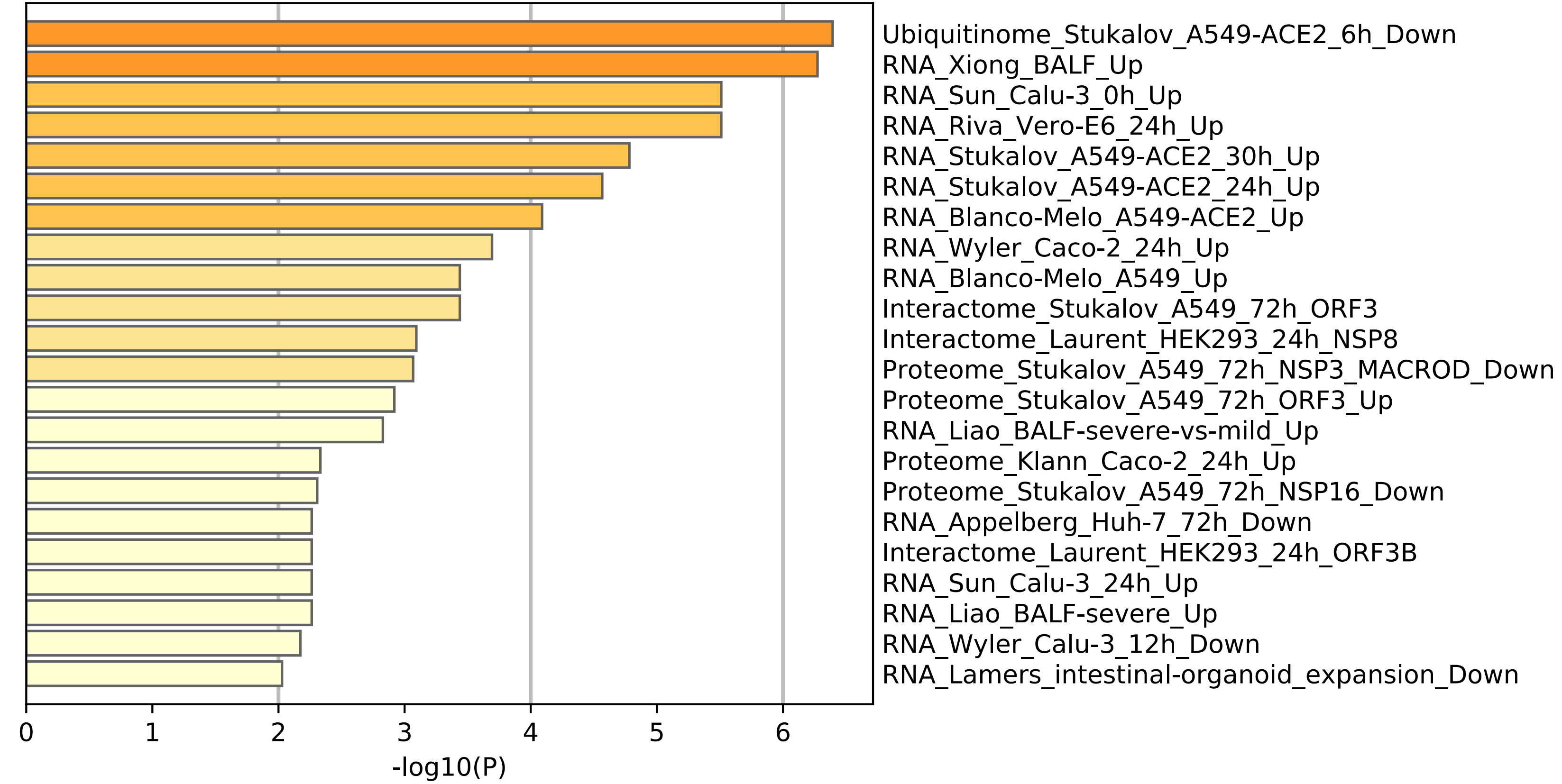

Ubiquitinome_Stukalov_A549-ACE2_6h_Down
RNA_Xiong_BALF_Up
RNA_Sun_Calu-3_0h_Up
RNA_Riva_Vero-E6_24h_Up
RNA_Stukalov_A549-ACE2_30h_Up
RNA_Stukalov_A549-ACE2_24h_Up
RNA_Blanco-Melo_A549-ACE2_Up
RNA_Wyler_Caco-2_24h_Up
RNA_Blanco-Melo_A549_Up
Interactome_Stukalov_A549_72h_ORF3
Interactome_Laurent_HEK293_24h_NSP8
Proteome_Stukalov_A549_72h_NSP3_MACROD_Down
Proteome_Stukalov_A549_72h_ORF3_Up
RNA_Liao_BALF-severe-vs-mild_Up
Proteome_Klann_Caco-2_24h_Up
Proteome_Stukalov_A549_72h_NSP16_Down
RNA_Appelberg_Huh-7_72h_Down
Interactome_Laurent_HEK293_24h_ORF3B
RNA_Sun_Calu-3_24h_Up
RNA_Liao_BALF-severe_Up
RNA_Wyler_Calu-3_12h_Down
RNA_Lamers_intestinal-organoid_expansion_Down
0
1
2
3
4
5
6
-log10(P)
