# Supplementary material for: Transcriptional profiling of Hutchinson-Gilford Progeria syndrome fibroblasts reveals deficits in mesenchymal stem cell commitment to differentiation related to early events in endochondral ossification
Source: eLife. 2022 Dec 29;11:e81290. doi: 10.7554/eLife.81290 (PMC9833827; doi:10.7554/eLife.81290)
Supplement: Table 1—source data 1. — Childhood and teenaged patient samples compared to middle aged and older adults. [file elife-81290-table1-data1.zip › Table_1_Source_Data_1/0-7 agematched_UP260.zip!/0-7 agematched_UP260/Enrichment_QC/HeatmapSelectedGO_DisGeNET.pdf]

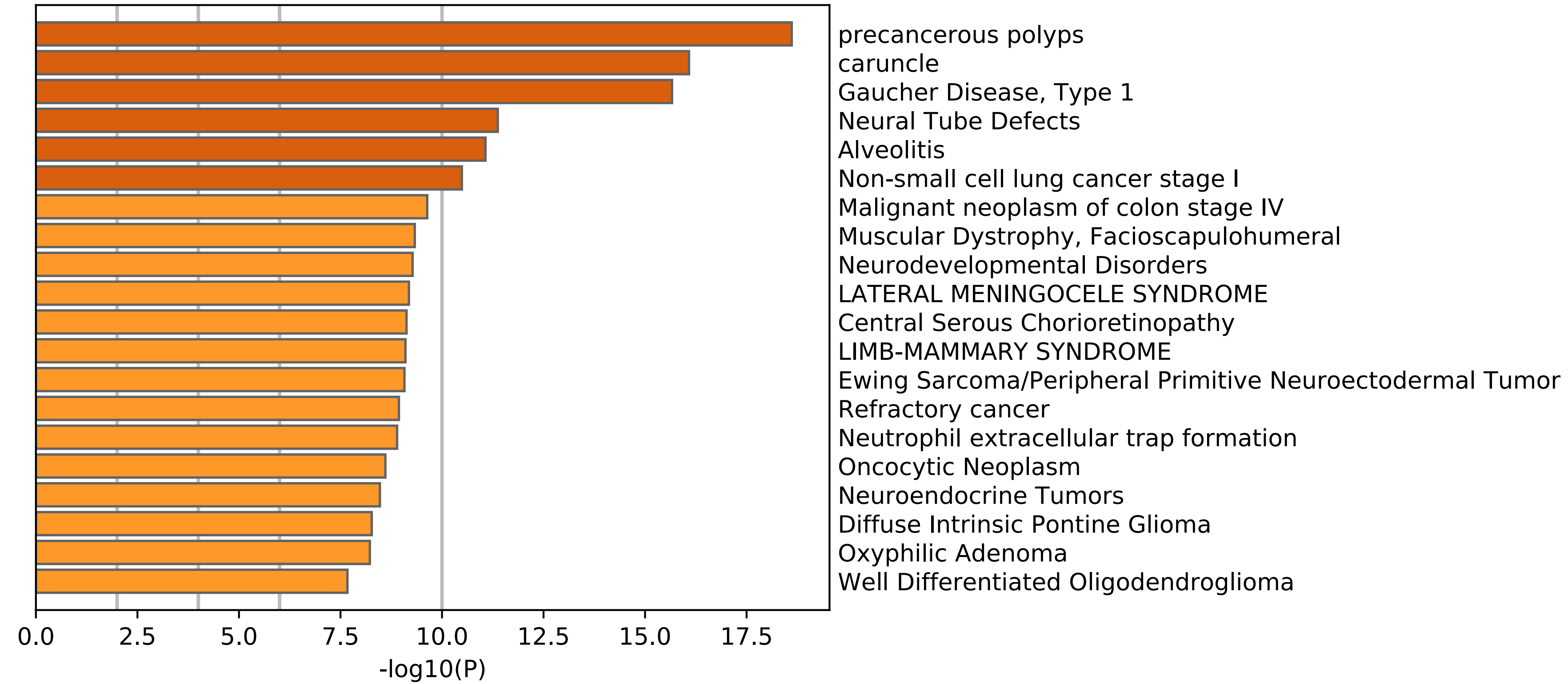
precancerous polyps
caruncle
Gaucher Disease, Type 1
Neural Tube Defects
Alveolitis
Non-small cell lung cancer stage I
Malignant neoplasm of colon stage IV
Muscular Dystrophy, Facioscapulohumeral
Neurodevelopmental Disorders
LATERAL MENINGOCELE SYNDROME
Central Serous Chorioretinopathy
LIMB-MAMMARY SYNDROME
Ewing Sarcoma/Peripheral Primitive Neuroectodermal Tumor
Refractory cancer
Neutrophil extracellular trap formation
Oncocytic Neoplasm
Neuroendocrine Tumors
Diffuse Intrinsic Pontine Glioma
Oxyphilic Adenoma
Well Differentiated Oligodendroglioma
0.0
2.5
5.0
7.5
10.0
12.5
15.0
17.5
-log10(P)
