# Supplementary material for: Transcriptional profiling of Hutchinson-Gilford Progeria syndrome fibroblasts reveals deficits in mesenchymal stem cell commitment to differentiation related to early events in endochondral ossification
Source: eLife. 2022 Dec 29;11:e81290. doi: 10.7554/eLife.81290 (PMC9833827; doi:10.7554/eLife.81290)
Supplement: Table 1—source data 1. — Childhood and teenaged patient samples compared to middle aged and older adults. [file elife-81290-table1-data1.zip › Table_1_Source_Data_1/0-7 agematched_UP260.zip!/0-7 agematched_UP260/Enrichment_QC/HeatmapSelectedGO_PaGenBase.pdf]

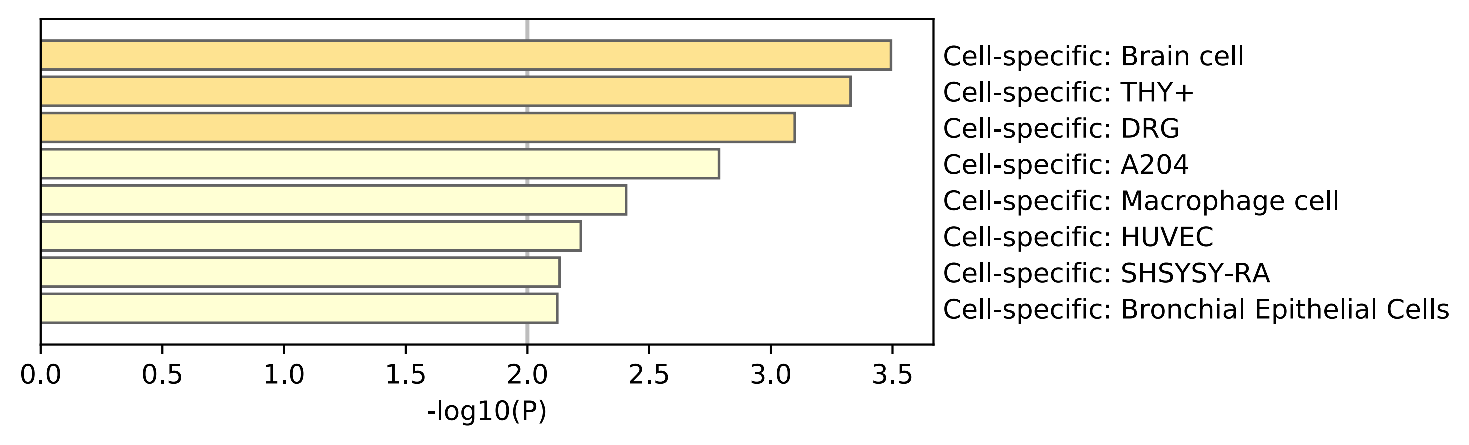

Cell-specific: Brain cell
Cell-specific: THY+
Cell-specific: DRG
Cell-specific: A204
Cell-specific: Macrophage cell
Cell-specific: HUVEC
Cell-specific: SHSYSY-RA
Cell-specific: Bronchial Epithelial Cells
0.0
0.5
1.0
1.5
2.0
2.5
3.0
3.5
-log10(P)
