# Supplementary material for: Transcriptional profiling of Hutchinson-Gilford Progeria syndrome fibroblasts reveals deficits in mesenchymal stem cell commitment to differentiation related to early events in endochondral ossification
Source: eLife. 2022 Dec 29;11:e81290. doi: 10.7554/eLife.81290 (PMC9833827; doi:10.7554/eLife.81290)
Supplement: Table 1—source data 1. — Childhood and teenaged patient samples compared to middle aged and older adults. [file elife-81290-table1-data1.zip › Table_1_Source_Data_1/0-7 agematched_UP260.zip!/0-7 agematched_UP260/Enrichment_QC/HeatmapSelectedGO_Transcription_Factor_Targets.pdf]

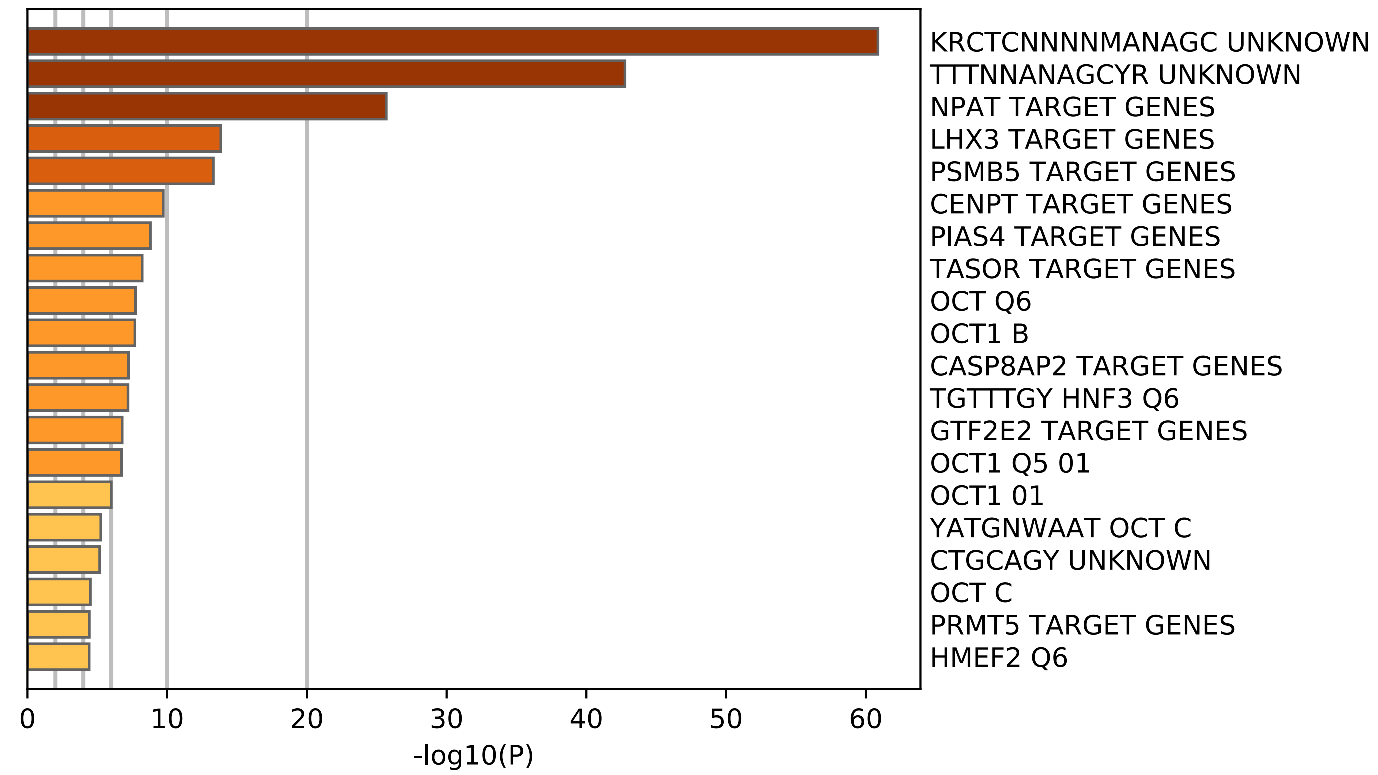
KRCTCNNNNMANAGC UNKNOWN
TTTNNANAGCYR UNKNOWN
NPAT TARGET GENES
LHX3 TARGET GENES
PSMB5 TARGET GENES
CENPT TARGET GENES
PIAS4 TARGET GENES
TASOR TARGET GENES
OCT Q6
OCT1 B
CASP8AP2 TARGET GENES
TGTTTGY HNF3 Q6
GTF2E2 TARGET GENES
OCT1 Q5 01
OCT1 01
YATGNWAAT OCT C
CTGCAGY UNKNOWN
OCT C
PRMT5 TARGET GENES
HMEF2 Q6
0
10
20
30
40
50
60
-log10(P)
