# Supplementary material for: Transcriptional profiling of Hutchinson-Gilford Progeria syndrome fibroblasts reveals deficits in mesenchymal stem cell commitment to differentiation related to early events in endochondral ossification
Source: eLife. 2022 Dec 29;11:e81290. doi: 10.7554/eLife.81290 (PMC9833827; doi:10.7554/eLife.81290)
Supplement: Table 1—source data 1. — Childhood and teenaged patient samples compared to middle aged and older adults. [file elife-81290-table1-data1.zip › Table_1_Source_Data_1/07_agematched_DOWN63.zip!/07_agematched_DOWN63/Enrichment_GO/ColorByCluster.pdf]

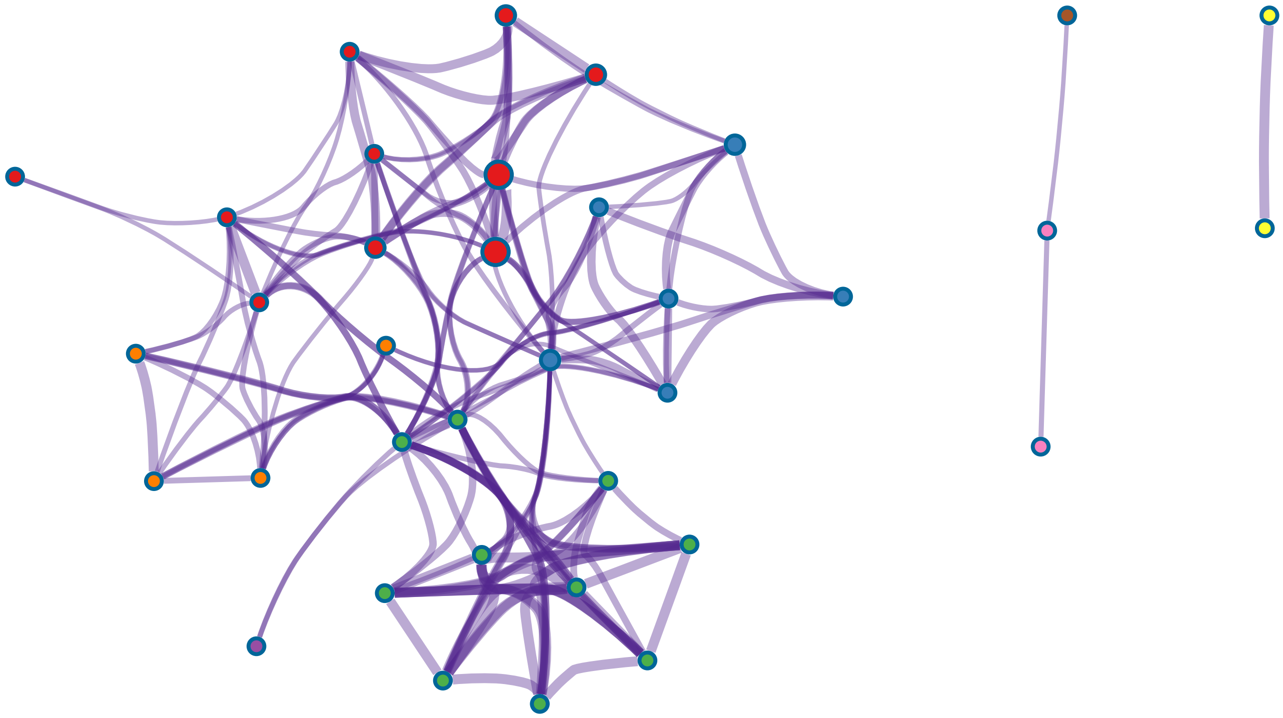

regulation of MAPK cascade
fat cell differentiation
regulation of muscle system process
thyroid hormone signaling pathway
regulation of G protein-coupled receptor signaling pa
NOD-like receptor signaling pathway
transmembrane receptor protein serine/threonine kin
anterior/posterior pattern specification

created by
http://metascape.org
