# Supplementary material for: Transcriptional profiling of Hutchinson-Gilford Progeria syndrome fibroblasts reveals deficits in mesenchymal stem cell commitment to differentiation related to early events in endochondral ossification
Source: eLife. 2022 Dec 29;11:e81290. doi: 10.7554/eLife.81290 (PMC9833827; doi:10.7554/eLife.81290)
Supplement: Table 1—source data 1. — Childhood and teenaged patient samples compared to middle aged and older adults. [file elife-81290-table1-data1.zip › Table_1_Source_Data_1/07_agematched_DOWN63.zip!/07_agematched_DOWN63/Enrichment_heatmap/HeatmapSelectedGO.pdf]

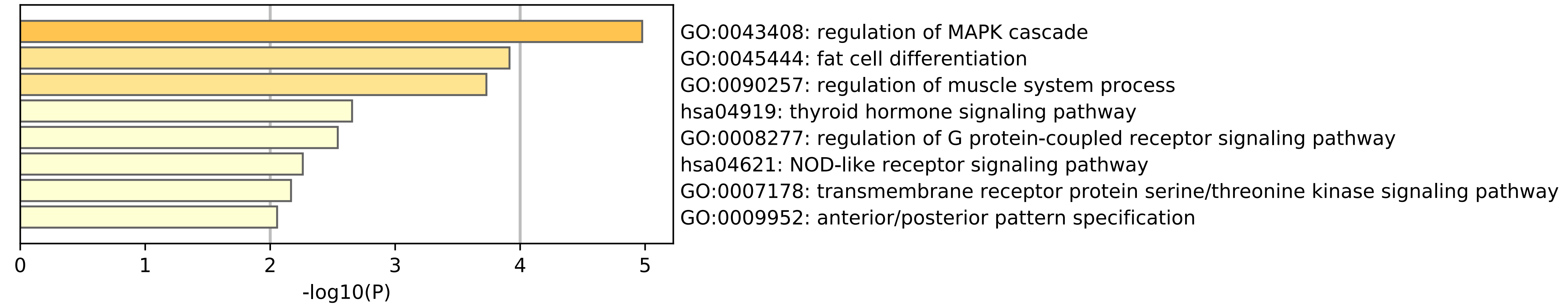
GO:0043408: regulation of MAPK cascade
GO:0045444: fat cell differentiation
GO:0090257: regulation of muscle system process
hsa04919: thyroid hormone signaling pathway
GO:0008277: regulation of G protein-coupled receptor signaling pathway
hsa04621: NOD-like receptor signaling pathway
GO:0007178: transmembrane receptor protein serine/threonine kinase signaling pathway
GO:0009952: anterior/posterior pattern specification
0
1
2
3
4
5
-log10(P)
