# Supplementary material for: Transcriptional profiling of Hutchinson-Gilford Progeria syndrome fibroblasts reveals deficits in mesenchymal stem cell commitment to differentiation related to early events in endochondral ossification
Source: eLife. 2022 Dec 29;11:e81290. doi: 10.7554/eLife.81290 (PMC9833827; doi:10.7554/eLife.81290)
Supplement: Table 1—source data 1. — Childhood and teenaged patient samples compared to middle aged and older adults. [file elife-81290-table1-data1.zip › Table_1_Source_Data_1/07_agematched_DOWN63.zip!/07_agematched_DOWN63/Enrichment_QC/HeatmapSelectedGO_DisGeNET.pdf]

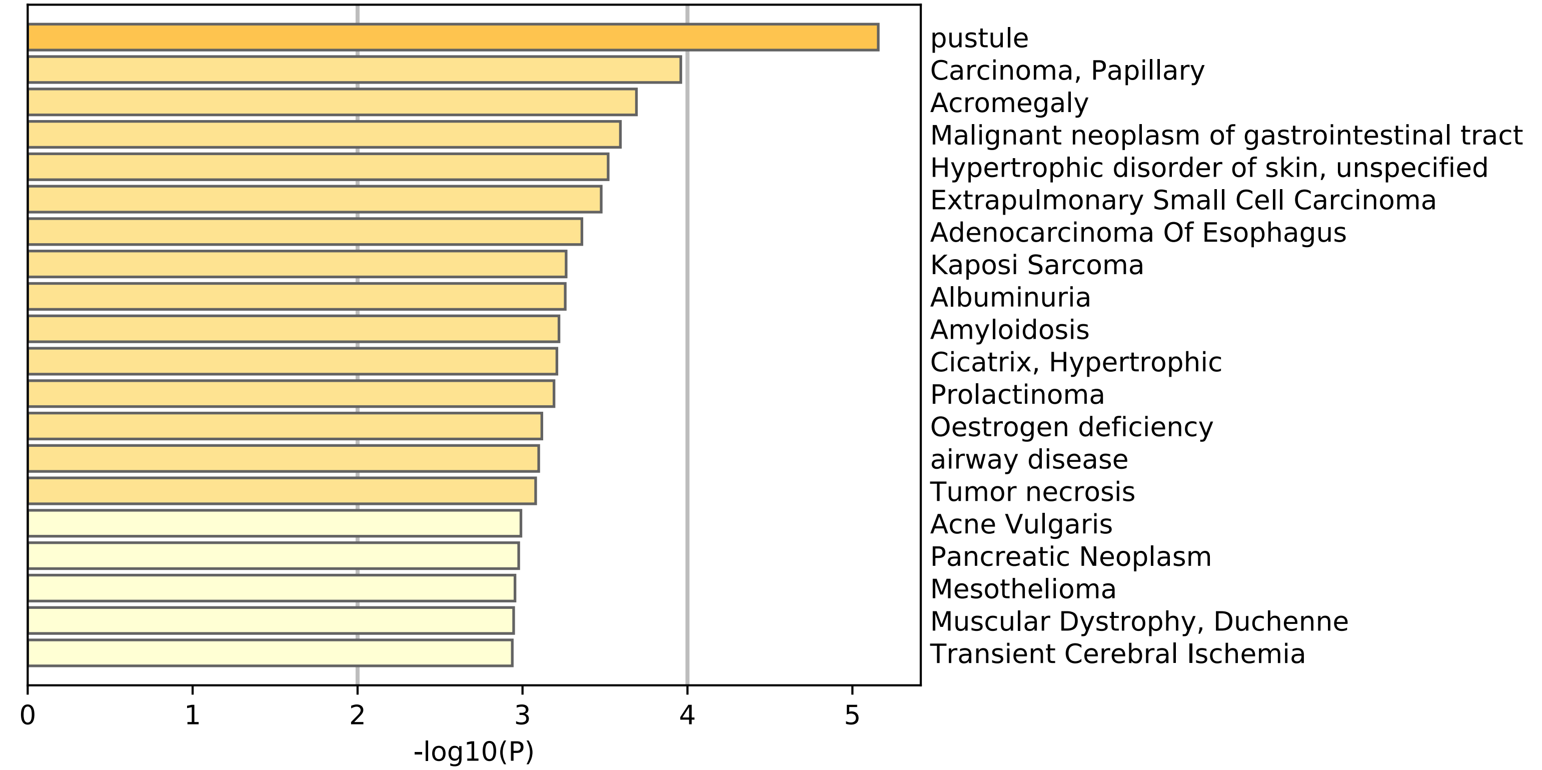
pustule
Carcinoma, Papillary
Acromegaly
Malignant neoplasm of gastrointestinal tract
Hypertrophic disorder of skin, unspecified
Extrapulmonary Small Cell Carcinoma
Adenocarcinoma Of Esophagus
Kaposi Sarcoma
Albuminuria
Amyloidosis
Cicatrix, Hypertrophic
Prolactinoma
Oestrogen deficiency
airway disease
Tumor necrosis
Acne Vulgaris
Pancreatic Neoplasm
Mesothelioma
Muscular Dystrophy, Duchenne
Transient Cerebral Ischemia
0
1
2
3
4
5
-log10(P)
