# Supplementary material for: Transcriptional profiling of Hutchinson-Gilford Progeria syndrome fibroblasts reveals deficits in mesenchymal stem cell commitment to differentiation related to early events in endochondral ossification
Source: eLife. 2022 Dec 29;11:e81290. doi: 10.7554/eLife.81290 (PMC9833827; doi:10.7554/eLife.81290)
Supplement: Table 1—source data 1. — Childhood and teenaged patient samples compared to middle aged and older adults. [file elife-81290-table1-data1.zip › Table_1_Source_Data_1/07_agematched_DOWN63.zip!/07_agematched_DOWN63/Enrichment_QC/HeatmapSelectedGO_Transcription_Factor_Targets.pdf]

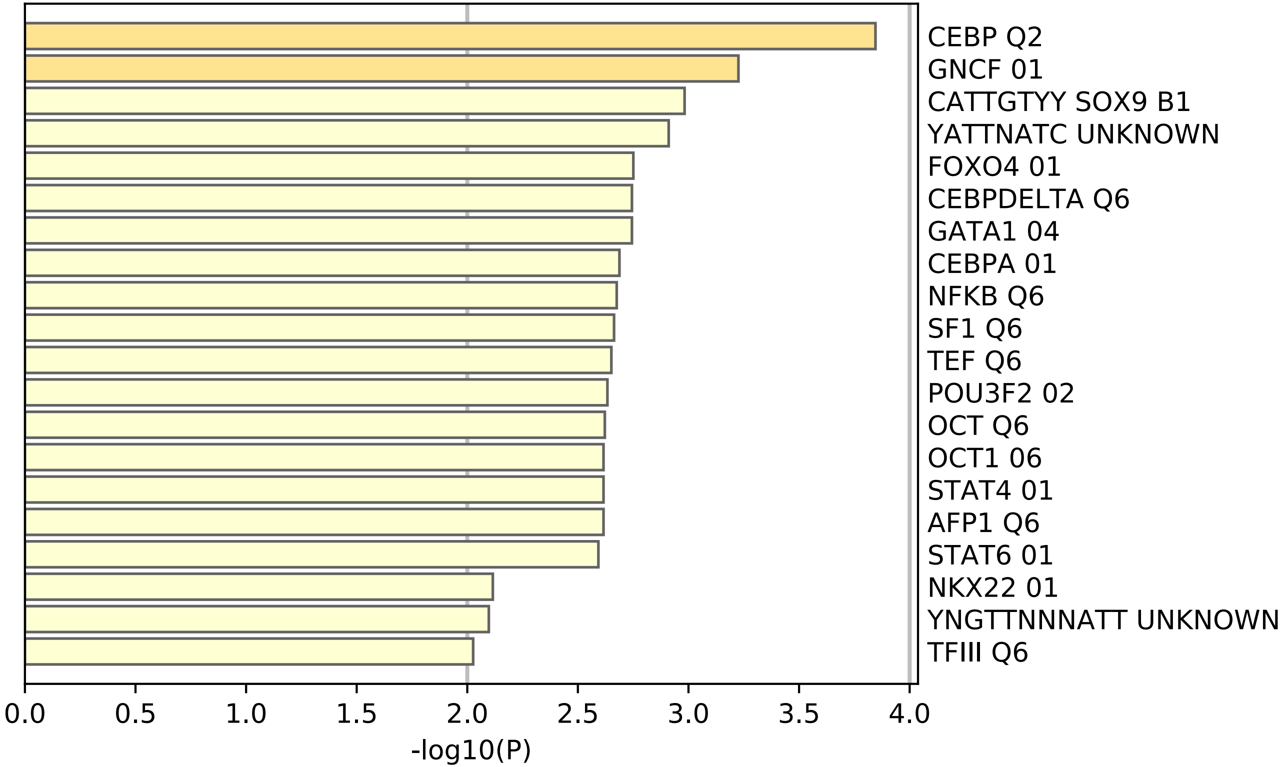
CEBP Q2
GNCF 01
CATTGTYY SOX9 B1
YATTNATC UNKNOWN
FOXO4 01
CEBPDELTA Q6
GATA1 04
CEBPA 01
NFKB Q6
SF1 Q6
TEF Q6
POU3F2 02
OCT Q6
OCT1 06
STAT4 01
AFP1 Q6
STAT6 01
NKX22 01
YNGTTNNNATT UNKNOWN
TFIII Q6
0.0
0.5
1.0
1.5
2.0
2.5
3.0
3.5
4.0
-log10(P)
