# Supplementary material for: Transcriptional profiling of Hutchinson-Gilford Progeria syndrome fibroblasts reveals deficits in mesenchymal stem cell commitment to differentiation related to early events in endochondral ossification
Source: eLife. 2022 Dec 29;11:e81290. doi: 10.7554/eLife.81290 (PMC9833827; doi:10.7554/eLife.81290)
Supplement: Table 1—source data 1. — Childhood and teenaged patient samples compared to middle aged and older adults. [file elife-81290-table1-data1.zip › Table_1_Source_Data_1/0-7_M_DOWN241.zip!/0-7_M_DOWN241/Enrichment_GO/ColorByCluster.pdf]

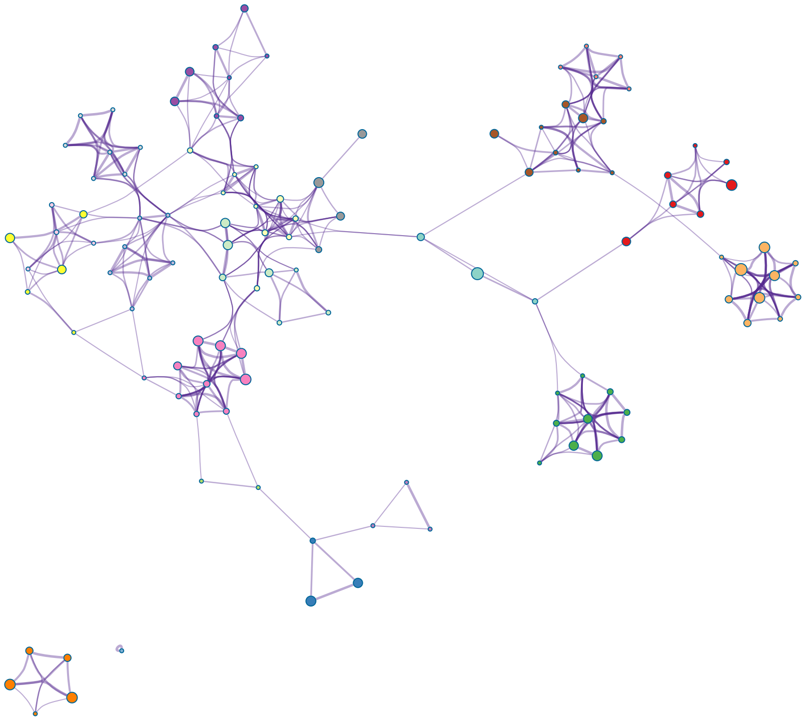

- Extracellular matrix organization
- Interleukin-4 and Interleukin-13 signaling
- transmembrane receptor protein serine/threonine kin
- peptidyl-tyrosine phosphorylation
- plasma membrane bounded cell projection assembly
- pattern specification process
- negative regulation of immunoglobulin production
- positive regulation of cellular component movement
- organic hydroxy compound metabolic process
- NABA MATRISOME ASSOCIATED
- regulation of cellular carbohydrate metabolic process
- amyloid precursor protein catabolic process
- macrophage cytokine production
- epithelial cell-cell adhesion
- positive regulation of cytosolic calcium ion concentrat
- trabecula morphogenesis
- formation of primary germ layer
- Hypertrophic cardiomyopathy (HCM)
- Histidine metabolism
- cellular response to ketone

created by
http://metascape.org
