# Supplementary material for: Transcriptional profiling of Hutchinson-Gilford Progeria syndrome fibroblasts reveals deficits in mesenchymal stem cell commitment to differentiation related to early events in endochondral ossification
Source: eLife. 2022 Dec 29;11:e81290. doi: 10.7554/eLife.81290 (PMC9833827; doi:10.7554/eLife.81290)
Supplement: Table 1—source data 1. — Childhood and teenaged patient samples compared to middle aged and older adults. [file elife-81290-table1-data1.zip › Table_1_Source_Data_1/0-7_M_DOWN241.zip!/0-7_M_DOWN241/Enrichment_heatmap/HeatmapSelectedGO.pdf]

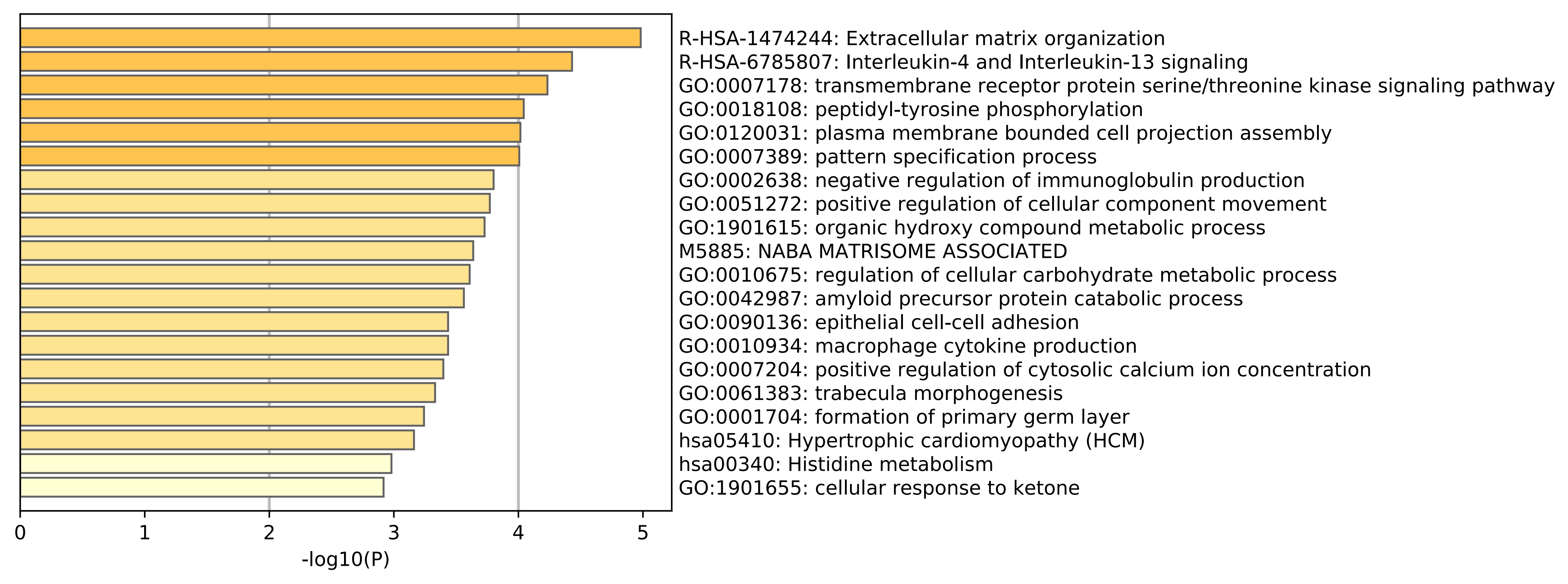
R-HSA-1474244: Extracellular matrix organization
R-HSA-6785807: Interleukin-4 and Interleukin-13 signaling
GO:0007178: transmembrane receptor protein serine/threonine kinase signaling pathway
GO:0018108: peptidyl-tyrosine phosphorylation
GO:0120031: plasma membrane bounded cell projection assembly
GO:0007389: pattern specification process
GO:0002638: negative regulation of immunoglobulin production
GO:0051272: positive regulation of cellular component movement
GO:1901615: organic hydroxy compound metabolic process
M5885: NABA MATRISOME ASSOCIATED
GO:0010675: regulation of cellular carbohydrate metabolic process
GO:0042987: amyloid precursor protein catabolic process
GO:0090136: epithelial cell-cell adhesion
GO:0010934: macrophage cytokine production
GO:0007204: positive regulation of cytosolic calcium ion concentration
GO:0061383: trabecula morphogenesis
GO:0001704: formation of primary germ layer
hsa05410: Hypertrophic cardiomyopathy (HCM)
hsa00340: Histidine metabolism
GO:1901655: cellular response to ketone
0
1
2
3
4
5
-log10(P)
