# Supplementary material for: Transcriptional profiling of Hutchinson-Gilford Progeria syndrome fibroblasts reveals deficits in mesenchymal stem cell commitment to differentiation related to early events in endochondral ossification
Source: eLife. 2022 Dec 29;11:e81290. doi: 10.7554/eLife.81290 (PMC9833827; doi:10.7554/eLife.81290)
Supplement: Table 1—source data 1. — Childhood and teenaged patient samples compared to middle aged and older adults. [file elife-81290-table1-data1.zip › Table_1_Source_Data_1/0-7_M_DOWN241.zip!/0-7_M_DOWN241/Enrichment_heatmap/HeatmapSelectedGOTop100.pdf]

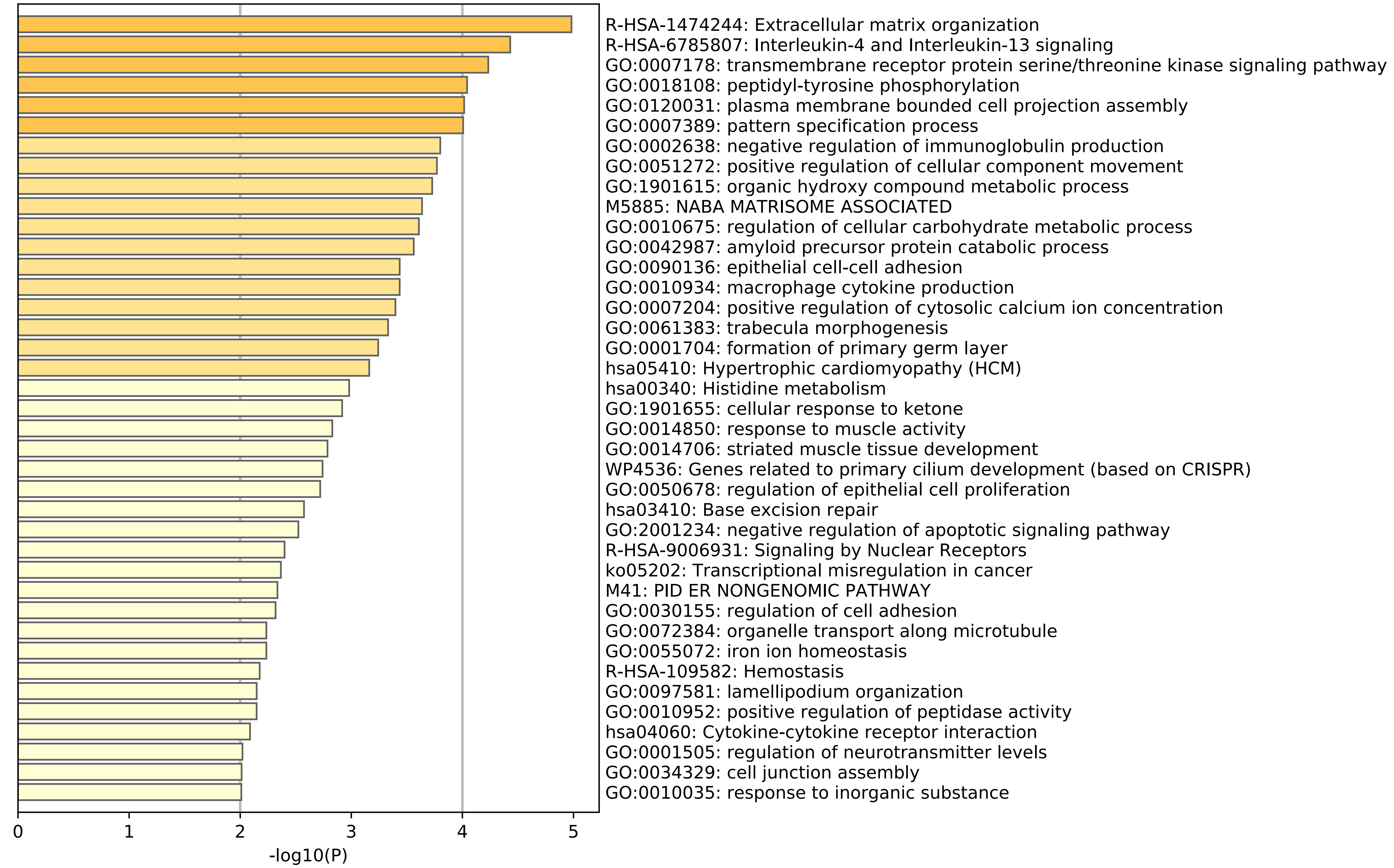
R-HSA-1474244: Extracellular matrix organization
R-HSA-6785807: Interleukin-4 and Interleukin-13 signaling
GO:0007178: transmembrane receptor protein serine/threonine kinase signaling pathway
GO:0018108: peptidyl-tyrosine phosphorylation
GO:0120031: plasma membrane bounded cell projection assembly
GO:0007389: pattern specification process
GO:0002638: negative regulation of immunoglobulin production
GO:0051272: positive regulation of cellular component movement
GO:1901615: organic hydroxy compound metabolic process
M5885: NABA MATRISOME ASSOCIATED
GO:0010675: regulation of cellular carbohydrate metabolic process
GO:0042987: amyloid precursor protein catabolic process
GO:0090136: epithelial cell-cell adhesion
GO:0010934: macrophage cytokine production
GO:0007204: positive regulation of cytosolic calcium ion concentration
GO:0061383: trabecula morphogenesis
GO:0001704: formation of primary germ layer
hsa05410: Hypertrophic cardiomyopathy (HCM)
hsa00340: Histidine metabolism
GO:1901655: cellular response to ketone
GO:0014850: response to muscle activity
GO:0014706: striated muscle tissue development
WP4536: Genes related to primary cilium development (based on CRISPR)
GO:0050678: regulation of epithelial cell proliferation
hsa03410: Base excision repair
GO:2001234: negative regulation of apoptotic signaling pathway
R-HSA-9006931: Signaling by Nuclear Receptors
ko05202: Transcriptional misregulation in cancer
M41: PID ER NONGENOMIC PATHWAY
GO:0030155: regulation of cell adhesion
GO:0072384: organelle transport along microtubule
GO:0055072: iron ion homeostasis
R-HSA-109582: Hemostasis
GO:0097581: lamellipodium organization
GO:0010952: positive regulation of peptidase activity
hsa04060: Cytokine-cytokine receptor interaction
GO:0001505: regulation of neurotransmitter levels
GO:0034329: cell junction assembly
GO:0010035: response to inorganic substance
0
1
2
3
4
5
-log10(P)
