# Supplementary material for: Transcriptional profiling of Hutchinson-Gilford Progeria syndrome fibroblasts reveals deficits in mesenchymal stem cell commitment to differentiation related to early events in endochondral ossification
Source: eLife. 2022 Dec 29;11:e81290. doi: 10.7554/eLife.81290 (PMC9833827; doi:10.7554/eLife.81290)
Supplement: Table 1—source data 1. — Childhood and teenaged patient samples compared to middle aged and older adults. [file elife-81290-table1-data1.zip › Table_1_Source_Data_1/0-7_M_DOWN241.zip!/0-7_M_DOWN241/Enrichment_PPI/MyList_MCODE_ALL_PPIColorByCluster.pdf]

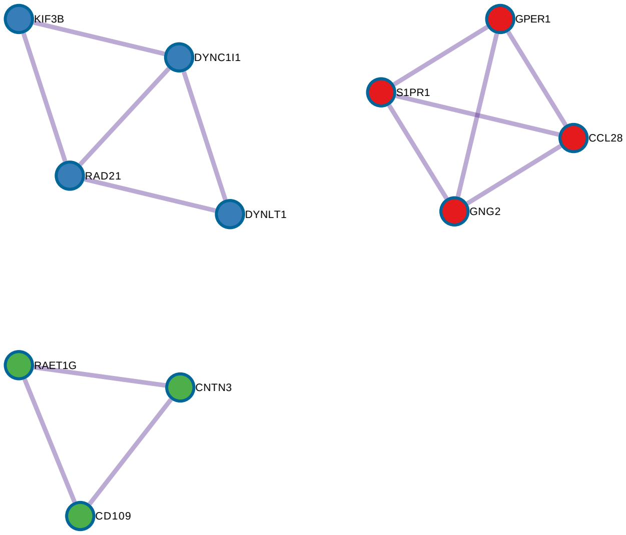
KIF3B
DYNC1I1
RAD21
DYNLT1
GPER1
S1PR1
CCL28
GNG2
RAET1G
CNTN3
CD109

MCODE1
MCODE2
MCODE3

created by
http://metascape.org
