# Supplementary material for: Transcriptional profiling of Hutchinson-Gilford Progeria syndrome fibroblasts reveals deficits in mesenchymal stem cell commitment to differentiation related to early events in endochondral ossification
Source: eLife. 2022 Dec 29;11:e81290. doi: 10.7554/eLife.81290 (PMC9833827; doi:10.7554/eLife.81290)
Supplement: Table 1—source data 1. — Childhood and teenaged patient samples compared to middle aged and older adults. [file elife-81290-table1-data1.zip › Table_1_Source_Data_1/0-7_M_DOWN241.zip!/0-7_M_DOWN241/Enrichment_QC/HeatmapSelectedGO_DisGeNET.pdf]

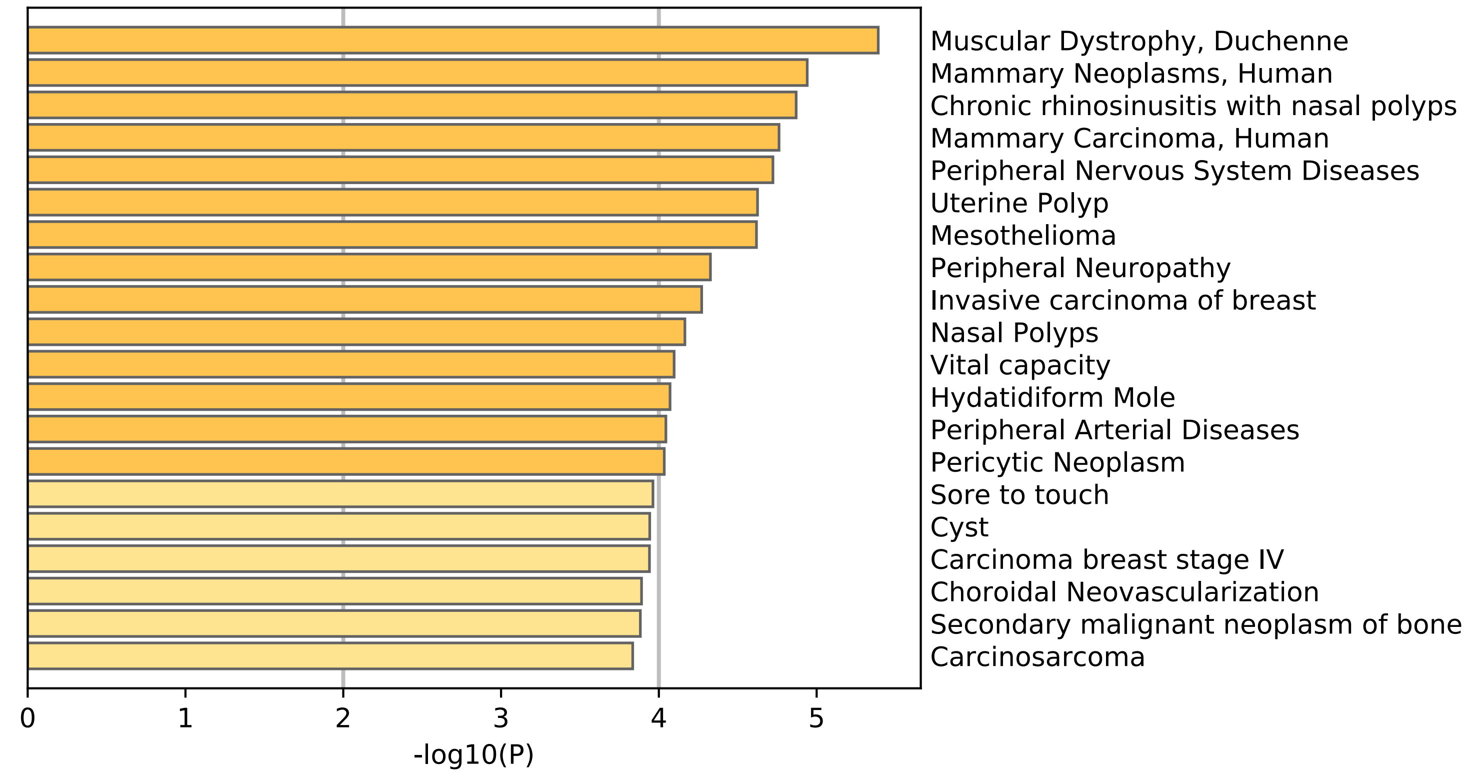

Muscular Dystrophy, Duchenne
Mammary Neoplasms, Human
Chronic rhinosinusitis with nasal polyps
Mammary Carcinoma, Human
Peripheral Nervous System Diseases
Uterine Polyp
Mesothelioma
Peripheral Neuropathy
Invasive carcinoma of breast
Nasal Polyps
Vital capacity
Hydatidiform Mole
Peripheral Arterial Diseases
Pericytic Neoplasm
Sore to touch
Cyst
Carcinoma breast stage IV
Choroidal Neovascularization
Secondary malignant neoplasm of bone
Carcinosarcoma
0
1
2
3
4
5
-log10(P)
