# Supplementary material for: Transcriptional profiling of Hutchinson-Gilford Progeria syndrome fibroblasts reveals deficits in mesenchymal stem cell commitment to differentiation related to early events in endochondral ossification
Source: eLife. 2022 Dec 29;11:e81290. doi: 10.7554/eLife.81290 (PMC9833827; doi:10.7554/eLife.81290)
Supplement: Table 1—source data 1. — Childhood and teenaged patient samples compared to middle aged and older adults. [file elife-81290-table1-data1.zip › Table_1_Source_Data_1/0-7_M_DOWN241.zip!/0-7_M_DOWN241/Enrichment_QC/HeatmapSelectedGO_Transcription_Factor_Targets.pdf]

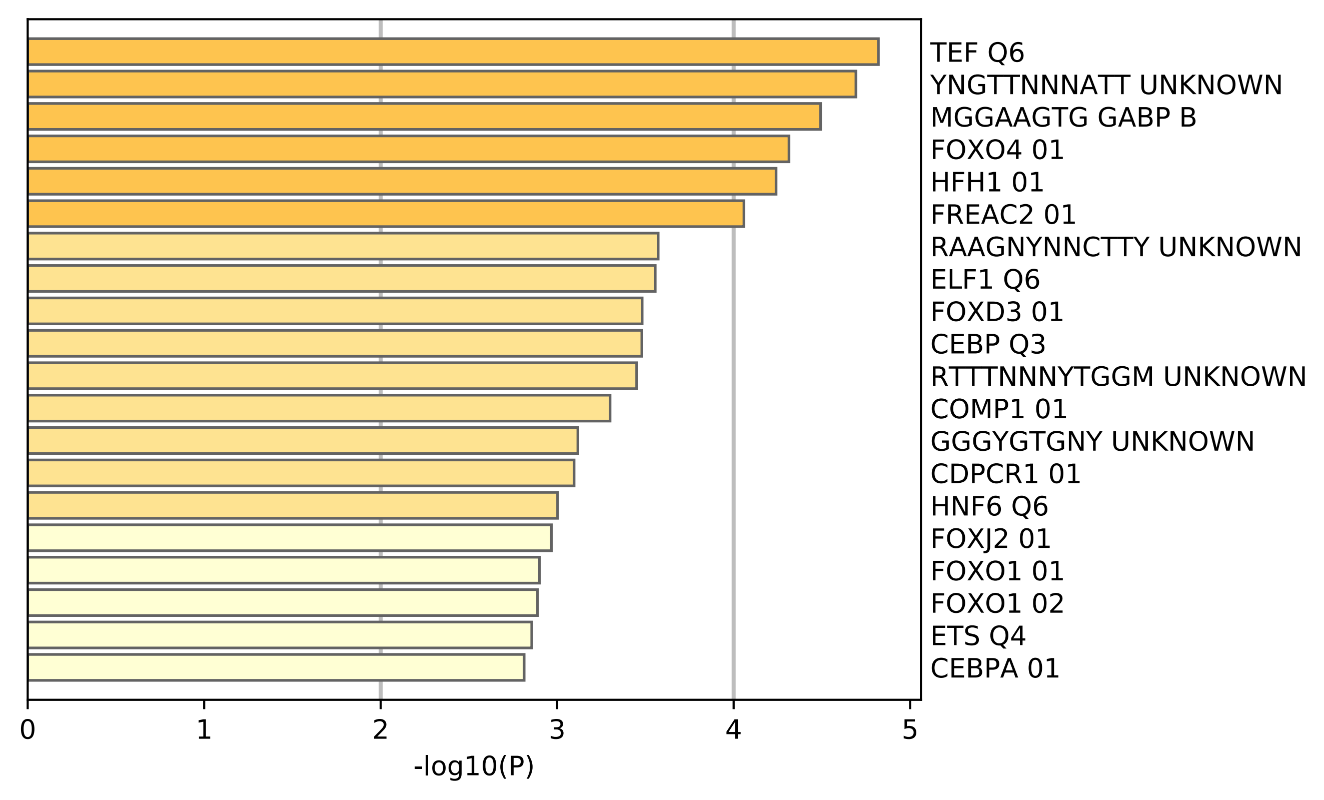

TEF Q6
YNGTTNNNATT UNKNOWN
MGGAAGTG GABP B
FOXO4 01
HFH1 01
FREAC2 01
RAAGNYNNCTTY UNKNOWN
ELF1 Q6
FOXD3 01
CEBP Q3
RTTTNNNYTGGM UNKNOWN
COMP1 01
GGGYGTGNY UNKNOWN
CDPCR1 01
HNF6 Q6
FOXJ2 01
FOXO1 01
FOXO1 02
ETS Q4
CEBPA 01
0
1
2
3
4
5
-log10(P)
