# Supplementary material for: Transcriptional profiling of Hutchinson-Gilford Progeria syndrome fibroblasts reveals deficits in mesenchymal stem cell commitment to differentiation related to early events in endochondral ossification
Source: eLife. 2022 Dec 29;11:e81290. doi: 10.7554/eLife.81290 (PMC9833827; doi:10.7554/eLife.81290)
Supplement: Table 1—source data 1. — Childhood and teenaged patient samples compared to middle aged and older adults. [file elife-81290-table1-data1.zip › Table_1_Source_Data_1/0-7_M_UP574.zip!/0-7_M_UP574/Enrichment_GO/ColorByCluster.pdf]

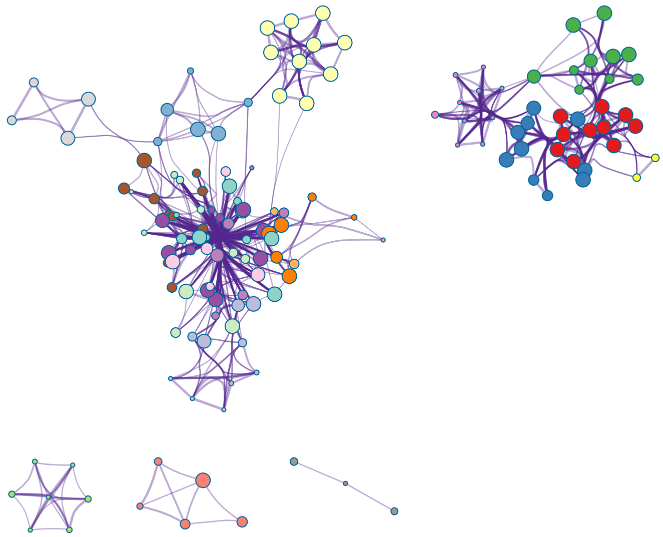

- HDACs deacetylate histones
- negative regulation of gene expression, epigenetic
- regulation of gene silencing
- tissue morphogenesis
- brain development
- E3 ubiquitin ligases ubiquitinate target proteins
- Pathways in cancer
- Formation of Senescence-Associated Heterochromat
- Genotoxicity pathway
- sensory organ development
- cell projection morphogenesis
- skin development
- regulation of cellular protein localization
- developmental growth
- neural precursor cell proliferation
- Amyotrophic lateral sclerosis (ALS)
- ossification
- regulation of neuron death
- Neural crest differentiation
- response to growth factor

created by
http://metascape.org
