# Supplementary material for: Transcriptional profiling of Hutchinson-Gilford Progeria syndrome fibroblasts reveals deficits in mesenchymal stem cell commitment to differentiation related to early events in endochondral ossification
Source: eLife. 2022 Dec 29;11:e81290. doi: 10.7554/eLife.81290 (PMC9833827; doi:10.7554/eLife.81290)
Supplement: Table 1—source data 1. — Childhood and teenaged patient samples compared to middle aged and older adults. [file elife-81290-table1-data1.zip › Table_1_Source_Data_1/0-7_M_UP574.zip!/0-7_M_UP574/Enrichment_heatmap/HeatmapSelectedGO.pdf]

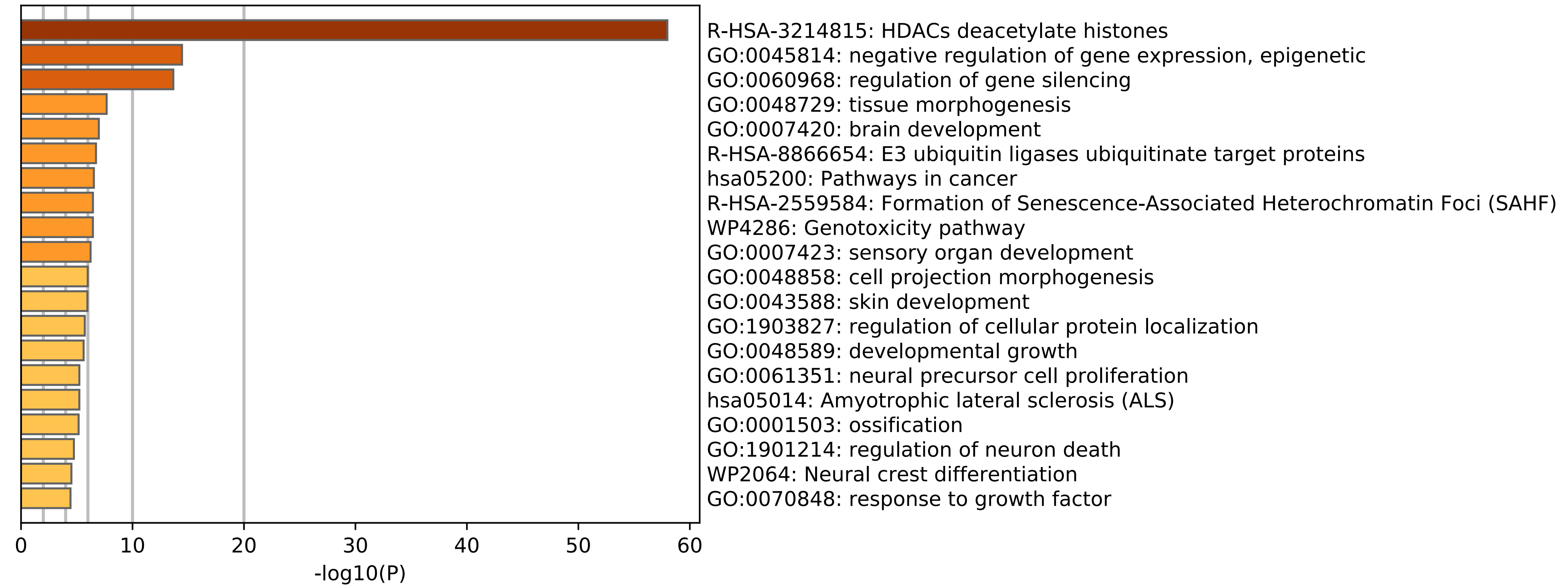
R-HSA-3214815: HDACs deacetylate histones
GO:0045814: negative regulation of gene expression, epigenetic
GO:0060968: regulation of gene silencing
GO:0048729: tissue morphogenesis
GO:0007420: brain development
R-HSA-8866654: E3 ubiquitin ligases ubiquitinate target proteins
hsa05200: Pathways in cancer
R-HSA-2559584: Formation of Senescence-Associated Heterochromatin Foci (SAHF)
WP4286: Genotoxicity pathway
GO:0007423: sensory organ development
GO:0048858: cell projection morphogenesis
GO:0043588: skin development
GO:1903827: regulation of cellular protein localization
GO:0048589: developmental growth
GO:0061351: neural precursor cell proliferation
hsa05014: Amyotrophic lateral sclerosis (ALS)
GO:0001503: ossification
GO:1901214: regulation of neuron death
WP2064: Neural crest differentiation
GO:0070848: response to growth factor
0
10
20
30
40
50
60
-log10(P)
