# Supplementary material for: Transcriptional profiling of Hutchinson-Gilford Progeria syndrome fibroblasts reveals deficits in mesenchymal stem cell commitment to differentiation related to early events in endochondral ossification
Source: eLife. 2022 Dec 29;11:e81290. doi: 10.7554/eLife.81290 (PMC9833827; doi:10.7554/eLife.81290)
Supplement: Table 1—source data 1. — Childhood and teenaged patient samples compared to middle aged and older adults. [file elife-81290-table1-data1.zip › Table_1_Source_Data_1/0-7_M_UP574.zip!/0-7_M_UP574/Enrichment_heatmap/HeatmapSelectedGOTop100.pdf]

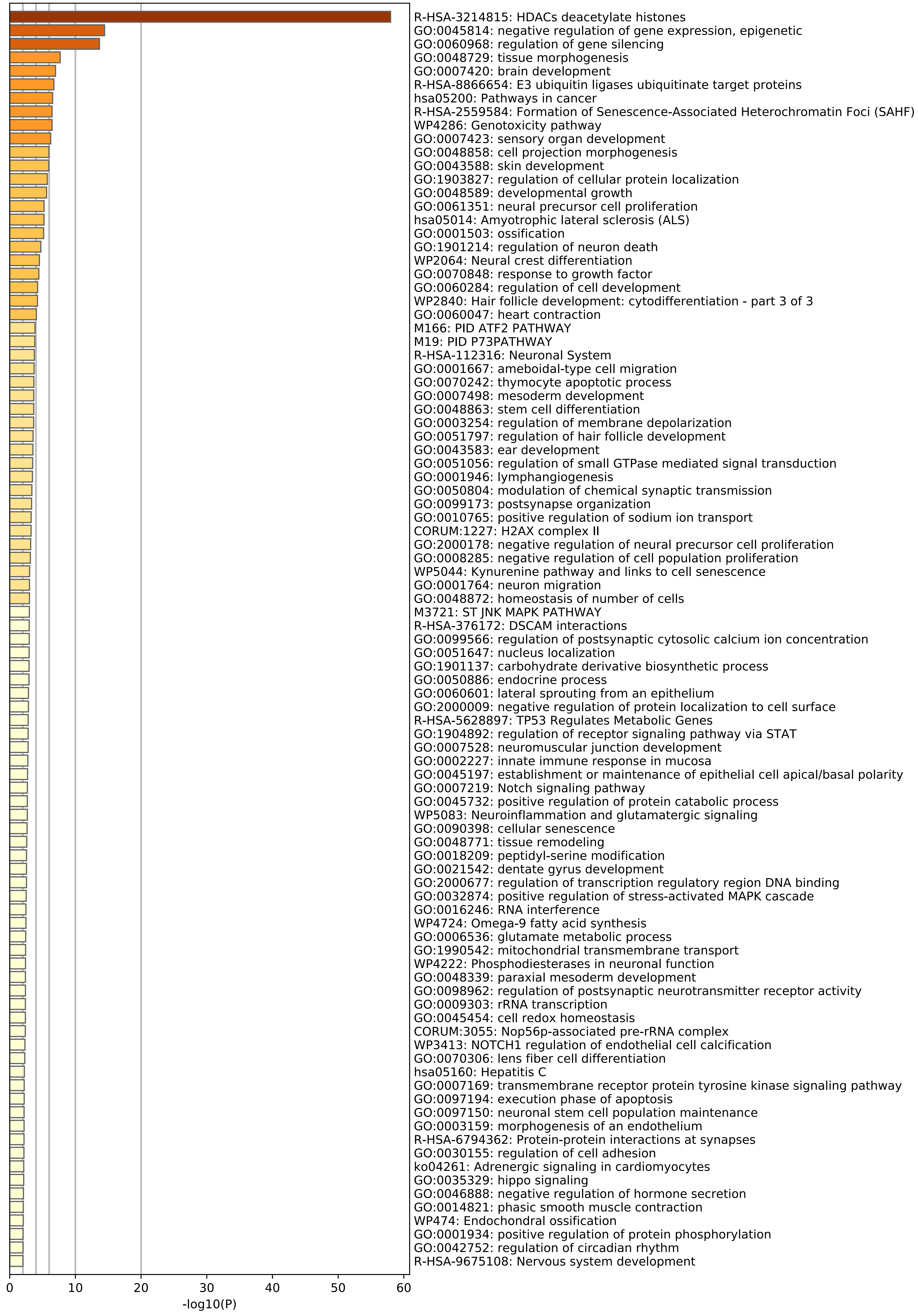
R-HSA-3214815: HDACs deacetylate histones
GO:0045814: negative regulation of gene expression, epigenetic
GO:0060968: regulation of gene silencing
GO:0048729: tissue morphogenesis
GO:0007420: brain development
R-HSA-8866654: E3 ubiquitin ligases ubiquitinate target proteins
hsa05200: Pathways in cancer
R-HSA-2559584: Formation of Senescence-Associated Heterochromatin Foci (SAHF)
WP4286: Genotoxicity pathway
GO:0007423: sensory organ development
GO:0048858: cell projection morphogenesis
GO:0043588: skin development
GO:1903827: regulation of cellular protein localization
GO:0048589: developmental growth
GO:0061351: neural precursor cell proliferation
hsa05014: Amyotrophic lateral sclerosis (ALS)
GO:0001503: ossification
GO:1901214: regulation of neuron death
WP2064: Neural crest differentiation
GO:0070848: response to growth factor
GO:0060284: regulation of cell development
WP2840: Hair follicle development: cytodifferentiation - part 3 of 3
GO:0060047: heart contraction
M166: PID ATF2 PATHWAY
M19: PID P73PATHWAY
R-HSA-112316: Neuronal System
GO:0001667: ameboidal-type cell migration
GO:0070242: thymocyte apoptotic process
GO:0007498: mesoderm development
GO:0048863: stem cell differentiation
GO:0003254: regulation of membrane depolarization
GO:0051797: regulation of hair follicle development
GO:0043583: ear development
GO:0051056: regulation of small GTPase mediated signal transduction
GO:0001946: lymphangiogenesis
GO:0050804: modulation of chemical synaptic transmission
GO:0099173: postsynapse organization
GO:0010765: positive regulation of sodium ion transport
CORUM:1227: H2AX complex II
GO:2000178: negative regulation of neural precursor cell proliferation
GO:0008285: negative regulation of cell population proliferation
WP5044: Kynurenine pathway and links to cell senescence
GO:0001764: neuron migration
GO:0048872: homeostasis of number of cells
M3721: ST JNK MAPK PATHWAY
R-HSA-376172: DSCAM interactions
GO:0099566: regulation of postsynaptic cytosolic calcium ion concentration
GO:0051647: nucleus localization
GO:1901137: carbohydrate derivative biosynthetic process
GO:0050886: endocrine process
GO:0060601: lateral sprouting from an epithelium
GO:2000009: negative regulation of protein localization to cell surface
R-HSA-5628897: TP53 Regulates Metabolic Genes
GO:1904892: regulation of receptor signaling pathway via STAT
GO:0007528: neuromuscular junction development
GO:0002227: innate immune response in mucosa
GO:0045197: establishment or maintenance of epithelial cell apical/basal polarity
GO:0007219: Notch signaling pathway
GO:0045732: positive regulation of protein catabolic process
WP5083: Neuroinflammation and glutamatergic signaling
GO:0090398: cellular senescence
GO:0048771: tissue remodeling
GO:0018209: peptidyl-serine modification
GO:0021542: dentate gyrus development
GO:2000677: regulation of transcription regulatory region DNA binding
GO:0032874: positive regulation of stress-activated MAPK cascade
GO:0016246: RNA interference
WP4724: Omega-9 fatty acid synthesis
GO:0006536: glutamate metabolic process
GO:1990542: mitochondrial transmembrane transport
WP4222: Phosphodiesterases in neuronal function
GO:0048339: paraxial mesoderm development
GO:0098962: regulation of postsynaptic neurotransmitter receptor activity
GO:0009303: rRNA transcription
GO:0045454: cell redox homeostasis
CORUM:3055: Nop56p-associated pre-rRNA complex
WP3413: NOTCH1 regulation of endothelial cell calcification
GO:0070306: lens fiber cell differentiation
hsa05160: Hepatitis C
GO:0007169: transmembrane receptor protein tyrosine kinase signaling pathway
GO:0097194: execution phase of apoptosis
GO:0097150: neuronal stem cell population maintenance
GO:0003159: morphogenesis of an endothelium
R-HSA-6794362: Protein-protein interactions at synapses
GO:0030155: regulation of cell adhesion
ko04261: Adrenergic signaling in cardiomyocytes
GO:0035329: hippo signaling
GO:0046888: negative regulation of hormone secretion
GO:0014821: phasic smooth muscle contraction
WP474: Endochondral ossification
GO:0001934: positive regulation of protein phosphorylation
GO:0042752: regulation of circadian rhythm
R-HSA-9675108: Nervous system development
0
10
20
30
40
50
60
-log10(P)
