# Supplementary material for: Transcriptional profiling of Hutchinson-Gilford Progeria syndrome fibroblasts reveals deficits in mesenchymal stem cell commitment to differentiation related to early events in endochondral ossification
Source: eLife. 2022 Dec 29;11:e81290. doi: 10.7554/eLife.81290 (PMC9833827; doi:10.7554/eLife.81290)
Supplement: Table 1—source data 1. — Childhood and teenaged patient samples compared to middle aged and older adults. [file elife-81290-table1-data1.zip › Table_1_Source_Data_1/0-7_O_DOWN_435.zip!/0-7_O_DOWN_435/Enrichment_GO/ColorByCluster.pdf]

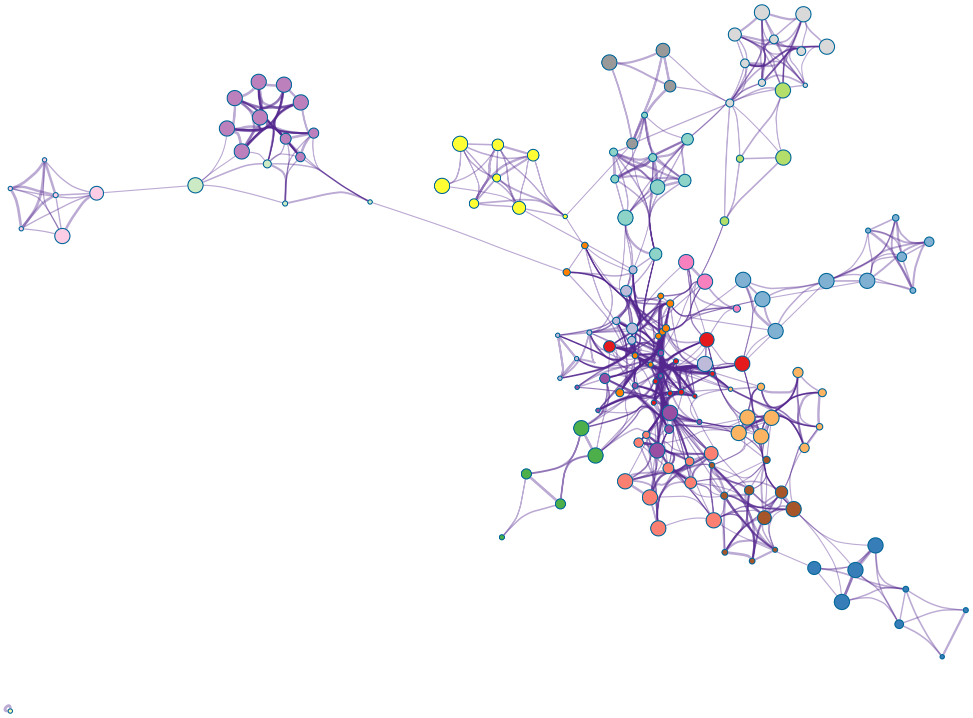

- EGF/EGFR signaling pathway
- extracellular matrix organization
- cell projection assembly
- lamellipodium organization
- PID ANGIOPOIETIN RECEPTOR PATHWAY
- lipid biosynthetic process
- negative regulation of cell-matrix adhesion
- Signaling by Interleukins
- localization within membrane
- Platelet activation, signaling and aggregation
- blood vessel maturation
- Rap1 signaling pathway
- supramolecular fiber organization
- cellular response to hormone stimulus
- positive regulation of locomotion
- endocytosis
- lipid localization
- negative regulation of transport
- ion homeostasis
- Transport of small molecules

created by
http://metascape.org
