# Supplementary material for: Transcriptional profiling of Hutchinson-Gilford Progeria syndrome fibroblasts reveals deficits in mesenchymal stem cell commitment to differentiation related to early events in endochondral ossification
Source: eLife. 2022 Dec 29;11:e81290. doi: 10.7554/eLife.81290 (PMC9833827; doi:10.7554/eLife.81290)
Supplement: Table 1—source data 1. — Childhood and teenaged patient samples compared to middle aged and older adults. [file elife-81290-table1-data1.zip › Table_1_Source_Data_1/0-7_O_DOWN_435.zip!/0-7_O_DOWN_435/Enrichment_heatmap/HeatmapSelectedGO.pdf]

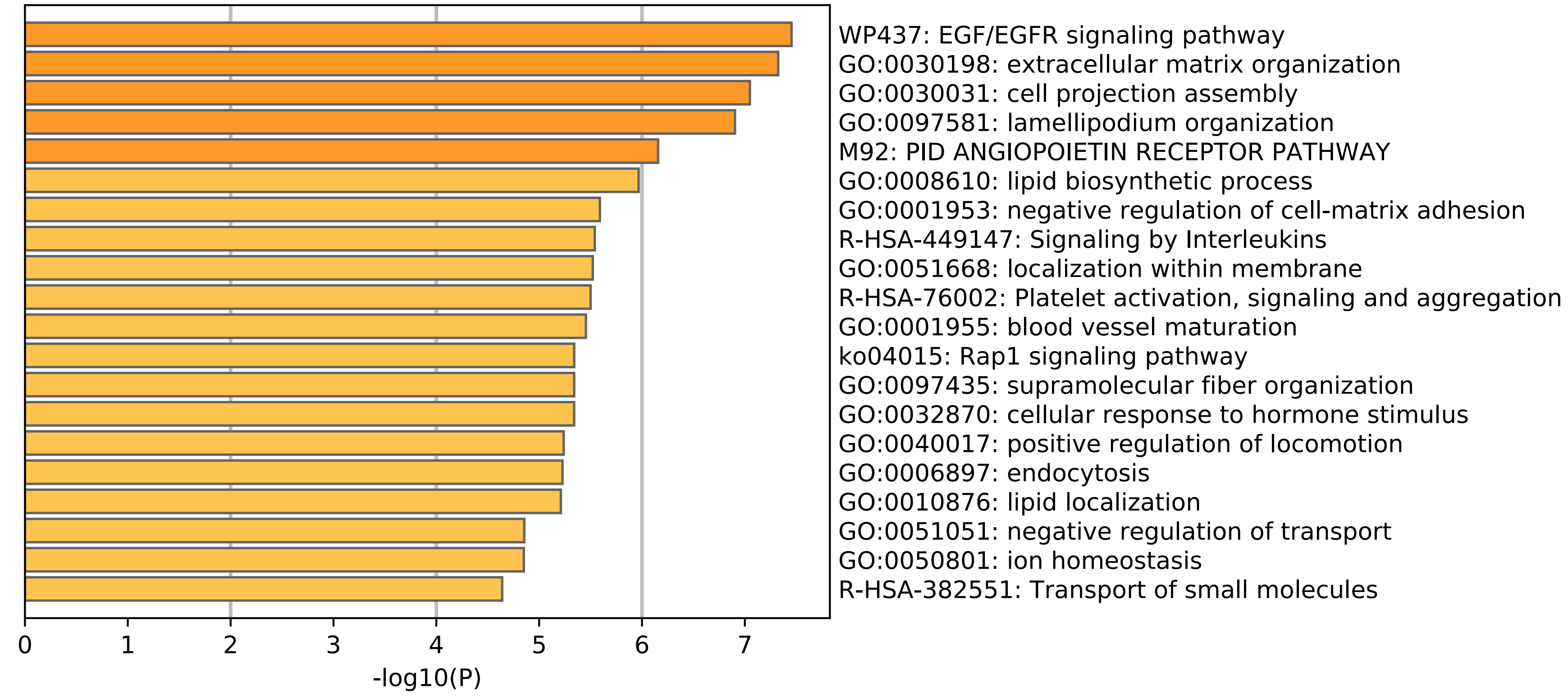
WP437: EGF/EGFR signaling pathway
GO:0030198: extracellular matrix organization
GO:0030031: cell projection assembly
GO:0097581: lamellipodium organization
M92: PID ANGIOPOIETIN RECEPTOR PATHWAY
GO:0008610: lipid biosynthetic process
GO:0001953: negative regulation of cell-matrix adhesion
R-HSA-449147: Signaling by Interleukins
GO:0051668: localization within membrane
R-HSA-76002: Platelet activation, signaling and aggregation
GO:0001955: blood vessel maturation
ko04015: Rap1 signaling pathway
GO:0097435: supramolecular fiber organization
GO:0032870: cellular response to hormone stimulus
GO:0040017: positive regulation of locomotion
GO:0006897: endocytosis
GO:0010876: lipid localization
GO:0051051: negative regulation of transport
GO:0050801: ion homeostasis
R-HSA-382551: Transport of small molecules
0
1
2
3
4
5
6
7
-log10(P)
