# Supplementary material for: Transcriptional profiling of Hutchinson-Gilford Progeria syndrome fibroblasts reveals deficits in mesenchymal stem cell commitment to differentiation related to early events in endochondral ossification
Source: eLife. 2022 Dec 29;11:e81290. doi: 10.7554/eLife.81290 (PMC9833827; doi:10.7554/eLife.81290)
Supplement: Table 1—source data 1. — Childhood and teenaged patient samples compared to middle aged and older adults. [file elife-81290-table1-data1.zip › Table_1_Source_Data_1/0-7_O_DOWN_435.zip!/0-7_O_DOWN_435/Enrichment_heatmap/HeatmapSelectedGOParent.pdf]

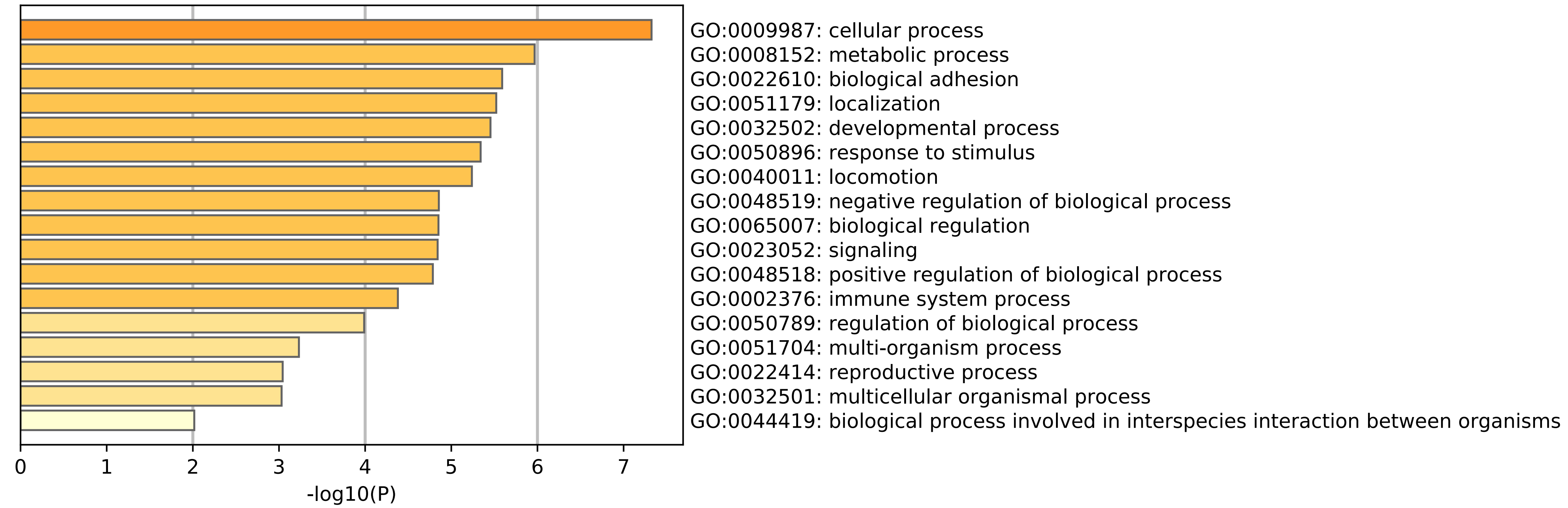
GO:0009987: cellular process
GO:0008152: metabolic process
GO:0022610: biological adhesion
GO:0051179: localization
GO:0032502: developmental process
GO:0050896: response to stimulus
GO:0040011: locomotion
GO:0048519: negative regulation of biological process
GO:0065007: biological regulation
GO:0023052: signaling
GO:0048518: positive regulation of biological process
GO:0002376: immune system process
GO:0050789: regulation of biological process
GO:0051704: multi-organism process
GO:0022414: reproductive process
GO:0032501: multicellular organismal process
GO:0044419: biological process involved in interspecies interaction between organisms
0
1
2
3
4
5
6
7
-log10(P)
