# Supplementary material for: Transcriptional profiling of Hutchinson-Gilford Progeria syndrome fibroblasts reveals deficits in mesenchymal stem cell commitment to differentiation related to early events in endochondral ossification
Source: eLife. 2022 Dec 29;11:e81290. doi: 10.7554/eLife.81290 (PMC9833827; doi:10.7554/eLife.81290)
Supplement: Table 1—source data 1. — Childhood and teenaged patient samples compared to middle aged and older adults. [file elife-81290-table1-data1.zip › Table_1_Source_Data_1/0-7_O_DOWN_435.zip!/0-7_O_DOWN_435/Enrichment_heatmap/HeatmapSelectedGOTop100.pdf]

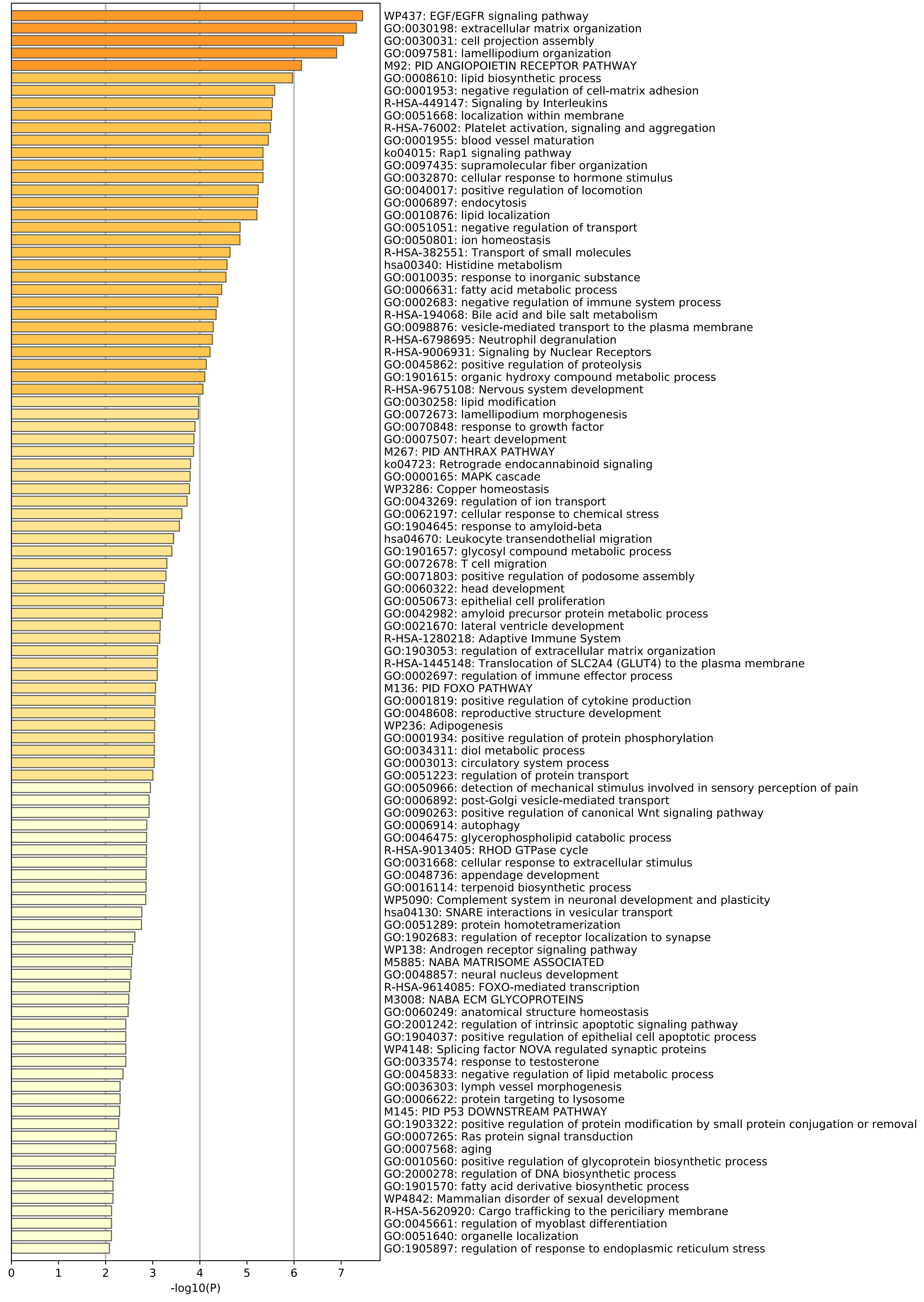
WP437: EGF/EGFR signaling pathway
GO:0030198: extracellular matrix organization
GO:0030031: cell projection assembly
GO:0097581: lamellipodium organization
M92: PID ANGIOPOIETIN RECEPTOR PATHWAY
GO:0008610: lipid biosynthetic process
GO:0001953: negative regulation of cell-matrix adhesion
R-HSA-449147: Signaling by Interleukins
GO:0051668: localization within membrane
R-HSA-76002: Platelet activation, signaling and aggregation
GO:0001955: blood vessel maturation
ko04015: Rap1 signaling pathway
GO:0097435: supramolecular fiber organization
GO:0032870: cellular response to hormone stimulus
GO:0040017: positive regulation of locomotion
GO:0006897: endocytosis
GO:0010876: lipid localization
GO:0051051: negative regulation of transport
GO:0050801: ion homeostasis
R-HSA-382551: Transport of small molecules
hsa00340: Histidine metabolism
GO:0010035: response to inorganic substance
GO:0006631: fatty acid metabolic process
GO:0002683: negative regulation of immune system process
R-HSA-194068: Bile acid and bile salt metabolism
GO:0098876: vesicle-mediated transport to the plasma membrane
R-HSA-6798695: Neutrophil degranulation
R-HSA-9006931: Signaling by Nuclear Receptors
GO:0045862: positive regulation of proteolysis
GO:1901615: organic hydroxy compound metabolic process
R-HSA-9675108: Nervous system development
GO:0030258: lipid modification
GO:0072673: lamellipodium morphogenesis
GO:0070848: response to growth factor
GO:0007507: heart development
M267: PID ANTHRAX PATHWAY
ko04723: Retrograde endocannabinoid signaling
GO:0000165: MAPK cascade
WP3286: Copper homeostasis
GO:0043269: regulation of ion transport
GO:0062197: cellular response to chemical stress
GO:1904645: response to amyloid-beta
hsa04670: Leukocyte transendothelial migration
GO:1901657: glycosyl compound metabolic process
GO:0072678: T cell migration
GO:0071803: positive regulation of podosome assembly
GO:0060322: head development
GO:0050673: epithelial cell proliferation
GO:0042982: amyloid precursor protein metabolic process
GO:0021670: lateral ventricle development
R-HSA-1280218: Adaptive Immune System
GO:1903053: regulation of extracellular matrix organization
R-HSA-1445148: Translocation of SLC2A4 (GLUT4) to the plasma membrane
GO:0002697: regulation of immune effector process
M136: PID FOXO PATHWAY
GO:0001819: positive regulation of cytokine production
GO:0048608: reproductive structure development
WP236: Adipogenesis
GO:0001934: positive regulation of protein phosphorylation
GO:0034311: diol metabolic process
GO:0003013: circulatory system process
GO:0051223: regulation of protein transport
GO:0050966: detection of mechanical stimulus involved in sensory perception of pain
GO:0006892: post-Golgi vesicle-mediated transport
GO:0090263: positive regulation of canonical Wnt signaling pathway
GO:0006914: autophagy
GO:0046475: glycerophospholipid catabolic process
R-HSA-9013405: RHOD GTPase cycle
GO:0031668: cellular response to extracellular stimulus
GO:0048736: appendage development
GO:0016114: terpenoid biosynthetic process
WP5090: Complement system in neuronal development and plasticity
hsa04130: SNARE interactions in vesicular transport
GO:0051289: protein homotetramerization
GO:1902683: regulation of receptor localization to synapse
WP138: Androgen receptor signaling pathway
M5885: NABA MATRISOME ASSOCIATED
GO:0048857: neural nucleus development
R-HSA-9614085: FOXO-mediated transcription
M3008: NABA ECM GLYCOPROTEINS
GO:0060249: anatomical structure homeostasis
GO:2001242: regulation of intrinsic apoptotic signaling pathway
GO:1904037: positive regulation of epithelial cell apoptotic process
WP4148: Splicing factor NOVA regulated synaptic proteins
GO:0033574: response to testosterone
GO:0045833: negative regulation of lipid metabolic process
GO:0036303: lymph vessel morphogenesis
GO:0006622: protein targeting to lysosome
M145: PID P53 DOWNSTREAM PATHWAY
GO:1903322: positive regulation of protein modification by small protein conjugation or removal
GO:0007265: Ras protein signal transduction
GO:0007568: aging
GO:0010560: positive regulation of glycoprotein biosynthetic process
GO:2000278: regulation of DNA biosynthetic process
GO:1901570: fatty acid derivative biosynthetic process
WP4842: Mammalian disorder of sexual development
R-HSA-5620920: Cargo trafficking to the periciliary membrane
GO:0045661: regulation of myoblast differentiation
GO:0051640: organelle localization
GO:1905897: regulation of response to endoplasmic reticulum stress
0
1
2
3
4
5
6
7
-log10(P)
