# Supplementary material for: Transcriptional profiling of Hutchinson-Gilford Progeria syndrome fibroblasts reveals deficits in mesenchymal stem cell commitment to differentiation related to early events in endochondral ossification
Source: eLife. 2022 Dec 29;11:e81290. doi: 10.7554/eLife.81290 (PMC9833827; doi:10.7554/eLife.81290)
Supplement: Table 1—source data 1. — Childhood and teenaged patient samples compared to middle aged and older adults. [file elife-81290-table1-data1.zip › Table_1_Source_Data_1/0-7_O_UP_metascape_984.zip!/0-7_O_UP_metascape_984/Enrichment_GO/ColorByCluster.pdf]

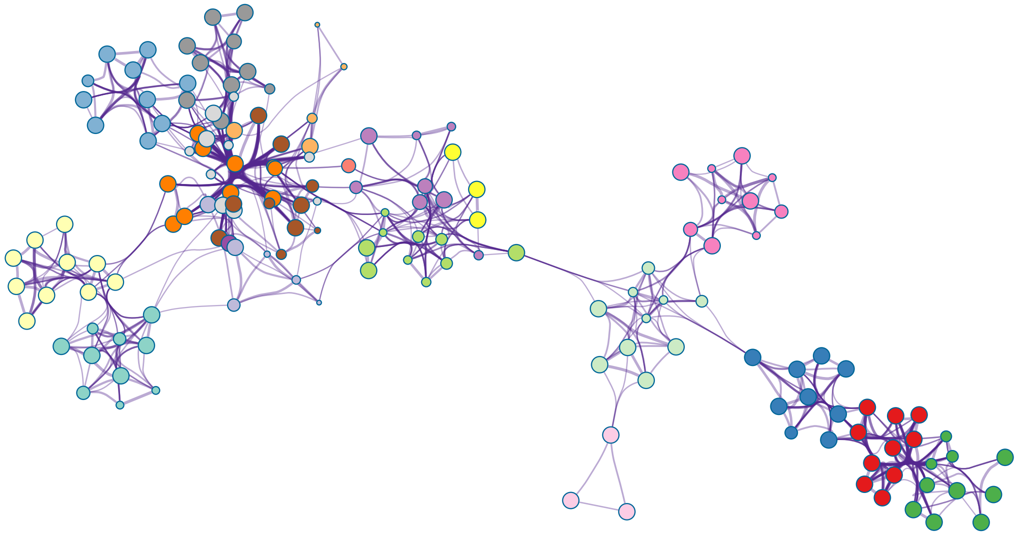

HDACs deacetylate histones
Ub-specific processing proteases
regulation of gene silencing
negative regulation of cell differentiation
tissue morphogenesis
Pathways in cancer
sensory organ development
Nervous system development
muscle structure development
brain development
cellular component morphogenesis
skeletal system development
Neural crest differentiation
ameboidal-type cell migration
ossification
Hippo signaling pathway
transmembrane receptor protein tyrosine kinase sign
stem cell differentiation
Breast cancer
MAPK family signaling cascades

created by
http://metascape.org
