# Supplementary material for: Transcriptional profiling of Hutchinson-Gilford Progeria syndrome fibroblasts reveals deficits in mesenchymal stem cell commitment to differentiation related to early events in endochondral ossification
Source: eLife. 2022 Dec 29;11:e81290. doi: 10.7554/eLife.81290 (PMC9833827; doi:10.7554/eLife.81290)
Supplement: Table 1—source data 1. — Childhood and teenaged patient samples compared to middle aged and older adults. [file elife-81290-table1-data1.zip › Table_1_Source_Data_1/0-7_O_UP_metascape_984.zip!/0-7_O_UP_metascape_984/Enrichment_heatmap/HeatmapSelectedGO.pdf]

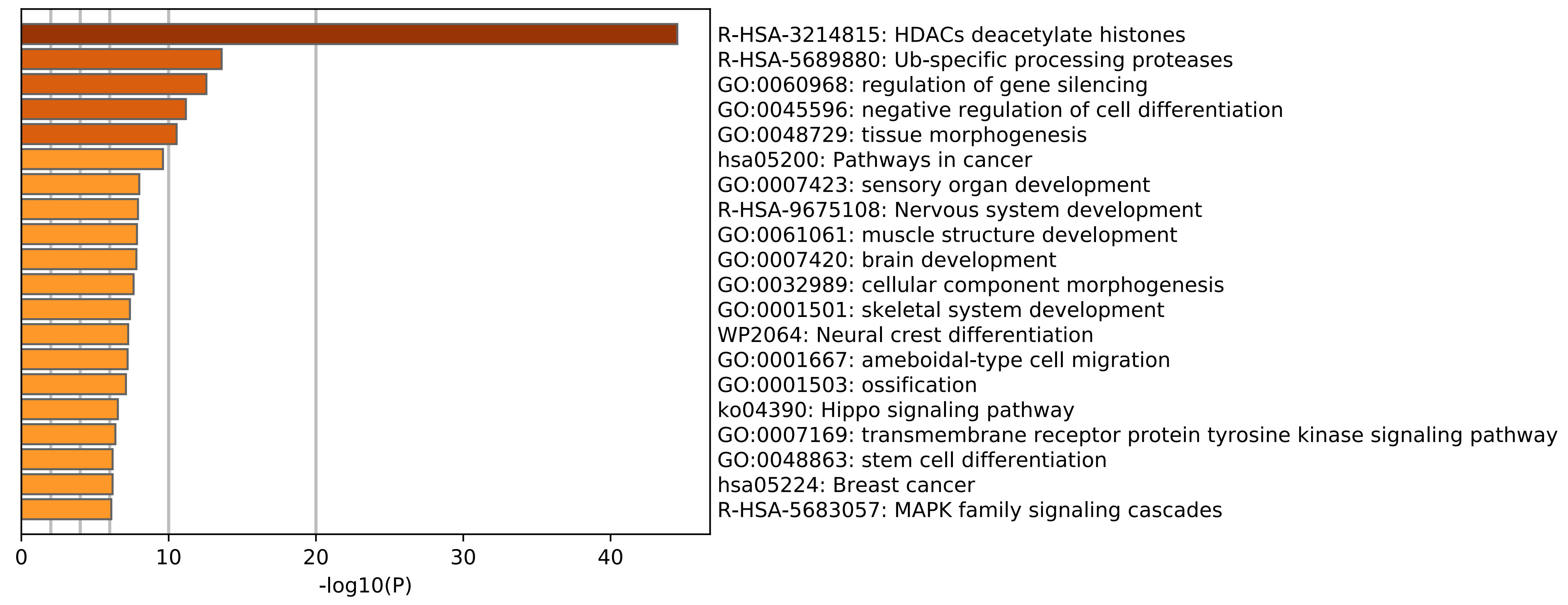
R-HSA-3214815: HDACs deacetylate histones
R-HSA-5689880: Ub-specific processing proteases
GO:0060968: regulation of gene silencing
GO:0045596: negative regulation of cell differentiation
GO:0048729: tissue morphogenesis
hsa05200: Pathways in cancer
GO:0007423: sensory organ development
R-HSA-9675108: Nervous system development
GO:0061061: muscle structure development
GO:0007420: brain development
GO:0032989: cellular component morphogenesis
GO:0001501: skeletal system development
WP2064: Neural crest differentiation
GO:0001667: ameboidal-type cell migration
GO:0001503: ossification
ko04390: Hippo signaling pathway
GO:0007169: transmembrane receptor protein tyrosine kinase signaling pathway
GO:0048863: stem cell differentiation
hsa05224: Breast cancer
R-HSA-5683057: MAPK family signaling cascades
0
10
20
30
40
-log10(P)
