# Supplementary material for: Transcriptional profiling of Hutchinson-Gilford Progeria syndrome fibroblasts reveals deficits in mesenchymal stem cell commitment to differentiation related to early events in endochondral ossification
Source: eLife. 2022 Dec 29;11:e81290. doi: 10.7554/eLife.81290 (PMC9833827; doi:10.7554/eLife.81290)
Supplement: Table 1—source data 1. — Childhood and teenaged patient samples compared to middle aged and older adults. [file elife-81290-table1-data1.zip › Table_1_Source_Data_1/0-7_O_UP_metascape_984.zip!/0-7_O_UP_metascape_984/Enrichment_heatmap/HeatmapSelectedGOTop100.pdf]

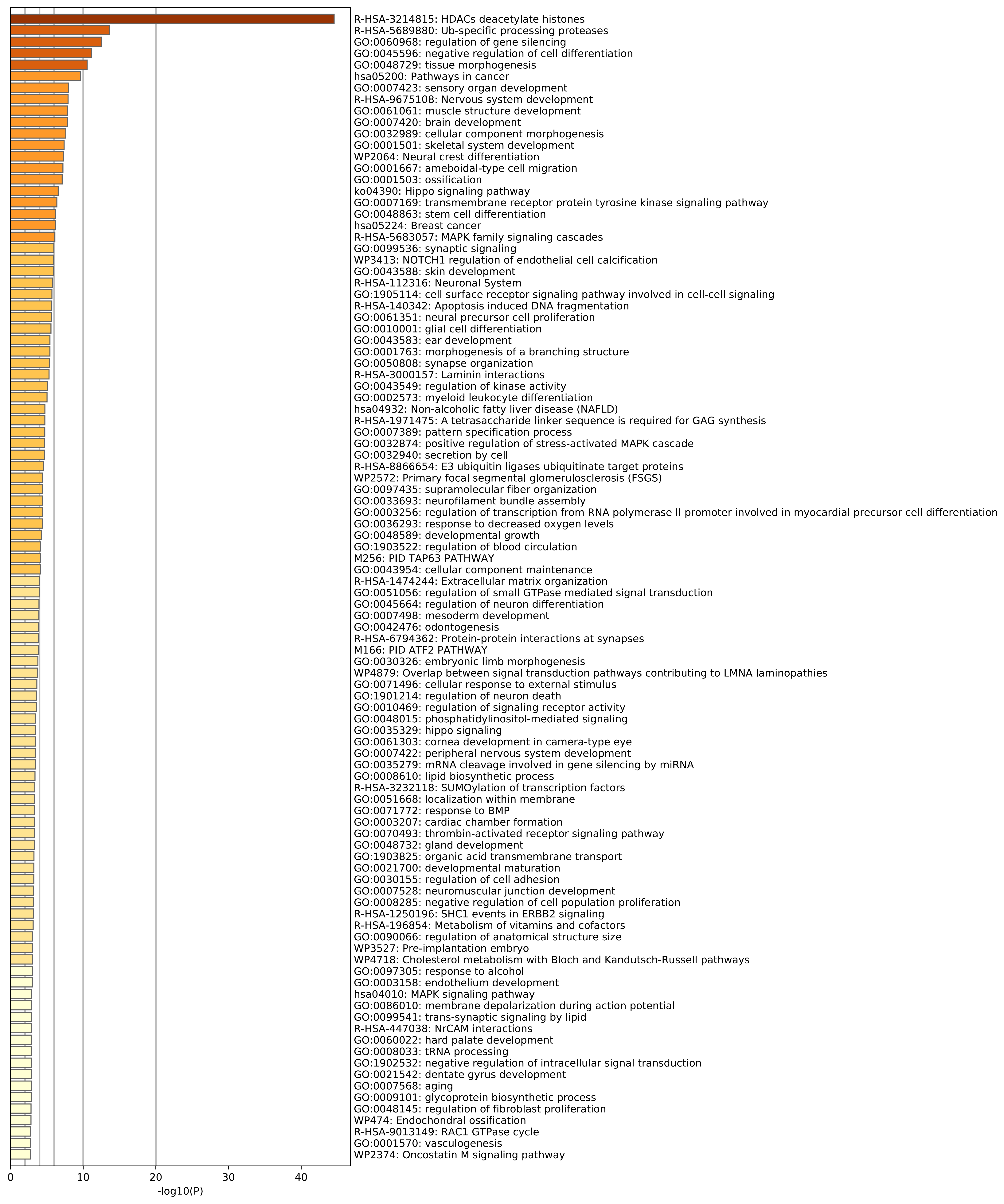
R-HSA-3214815: HDACs deacetylate histones
R-HSA-5689880: Ub-specific processing proteases
GO:0060968: regulation of gene silencing
GO:0045596: negative regulation of cell differentiation
GO:0048729: tissue morphogenesis
hsa05200: Pathways in cancer
GO:0007423: sensory organ development
R-HSA-9675108: Nervous system development
GO:0061061: muscle structure development
GO:0007420: brain development
GO:0032989: cellular component morphogenesis
GO:0001501: skeletal system development
WP2064: Neural crest differentiation
GO:0001667: ameboidal-type cell migration
GO:0001503: ossification
ko04390: Hippo signaling pathway
GO:0007169: transmembrane receptor protein tyrosine kinase signaling pathway
GO:0048863: stem cell differentiation
hsa05224: Breast cancer
R-HSA-5683057: MAPK family signaling cascades
GO:0099536: synaptic signaling
WP3413: NOTCH1 regulation of endothelial cell calcification
GO:0043588: skin development
R-HSA-112316: Neuronal System
GO:1905114: cell surface receptor signaling pathway involved in cell-cell signaling
R-HSA-140342: Apoptosis induced DNA fragmentation
GO:0061351: neural precursor cell proliferation
GO:0010001: glial cell differentiation
GO:0043583: ear development
GO:0001763: morphogenesis of a branching structure
GO:0050808: synapse organization
R-HSA-3000157: Laminin interactions
GO:0043549: regulation of kinase activity
GO:0002573: myeloid leukocyte differentiation
hsa04932: Non-alcoholic fatty liver disease (NAFLD)
R-HSA-1971475: A tetrasaccharide linker sequence is required for GAG synthesis
GO:0007389: pattern specification process
GO:0032874: positive regulation of stress-activated MAPK cascade
GO:0032940: secretion by cell
R-HSA-8866654: E3 ubiquitin ligases ubiquitinate target proteins
WP2572: Primary focal segmental glomerulosclerosis (FSGS)
GO:0097435: supramolecular fiber organization
GO:0033693: neurofilament bundle assembly
GO:0003256: regulation of transcription from RNA polymerase II promoter involved in myocardial precursor cell differentiation
GO:0036293: response to decreased oxygen levels
GO:0048589: developmental growth
GO:1903522: regulation of blood circulation
M256: PID TAP63 PATHWAY
GO:0043954: cellular component maintenance
R-HSA-1474244: Extracellular matrix organization
GO:0051056: regulation of small GTPase mediated signal transduction
GO:0045664: regulation of neuron differentiation
GO:0007498: mesoderm development
GO:0042476: odontogenesis
R-HSA-6794362: Protein-protein interactions at synapses
M166: PID ATF2 PATHWAY
GO:0030326: embryonic limb morphogenesis
WP4879: Overlap between signal transduction pathways contributing to LMNA laminopathies
GO:0071496: cellular response to external stimulus
GO:1901214: regulation of neuron death
GO:0010469: regulation of signaling receptor activity
GO:0048015: phosphatidylinositol-mediated signaling
GO:0035329: hippo signaling
GO:0061303: cornea development in camera-type eye
GO:0007422: peripheral nervous system development
GO:0035279: mRNA cleavage involved in gene silencing by miRNA
GO:0008610: lipid biosynthetic process
R-HSA-3232118: SUMOylation of transcription factors
GO:0051668: localization within membrane
GO:0071772: response to BMP
GO:0003207: cardiac chamber formation
GO:0070493: thrombin-activated receptor signaling pathway
GO:0048732: gland development
GO:1903825: organic acid transmembrane transport
GO:0021700: developmental maturation
GO:0030155: regulation of cell adhesion
GO:0007528: neuromuscular junction development
GO:0008285: negative regulation of cell population proliferation
R-HSA-1250196: SHC1 events in ERBB2 signaling
R-HSA-196854: Metabolism of vitamins and cofactors
GO:0090066: regulation of anatomical structure size
WP3527: Pre-implantation embryo
WP4718: Cholesterol metabolism with Bloch and Kandutsch-Russell pathways
GO:0097305: response to alcohol
GO:0003158: endothelium development
hsa04010: MAPK signaling pathway
GO:0086010: membrane depolarization during action potential
GO:0099541: trans-synaptic signaling by lipid
R-HSA-447038: NrCAM interactions
GO:0060022: hard palate development
GO:0008033: tRNA processing
GO:1902532: negative regulation of intracellular signal transduction
GO:0021542: dentate gyrus development
GO:0007568: aging
GO:0009101: glycoprotein biosynthetic process
GO:0048145: regulation of fibroblast proliferation
WP474: Endochondral ossification
R-HSA-9013149: RAC1 GTPase cycle
GO:0001570: vasculogenesis
WP2374: Oncostatin M signaling pathway
0
10
20
30
40
-log10(P)
