# Supplementary material for: Transcriptional profiling of Hutchinson-Gilford Progeria syndrome fibroblasts reveals deficits in mesenchymal stem cell commitment to differentiation related to early events in endochondral ossification
Source: eLife. 2022 Dec 29;11:e81290. doi: 10.7554/eLife.81290 (PMC9833827; doi:10.7554/eLife.81290)
Supplement: Table 1—source data 1. — Childhood and teenaged patient samples compared to middle aged and older adults. [file elife-81290-table1-data1.zip › Table_1_Source_Data_1/0-7_O_UP_metascape_984.zip!/0-7_O_UP_metascape_984/Enrichment_QC/HeatmapSelectedGO_COVID.pdf]

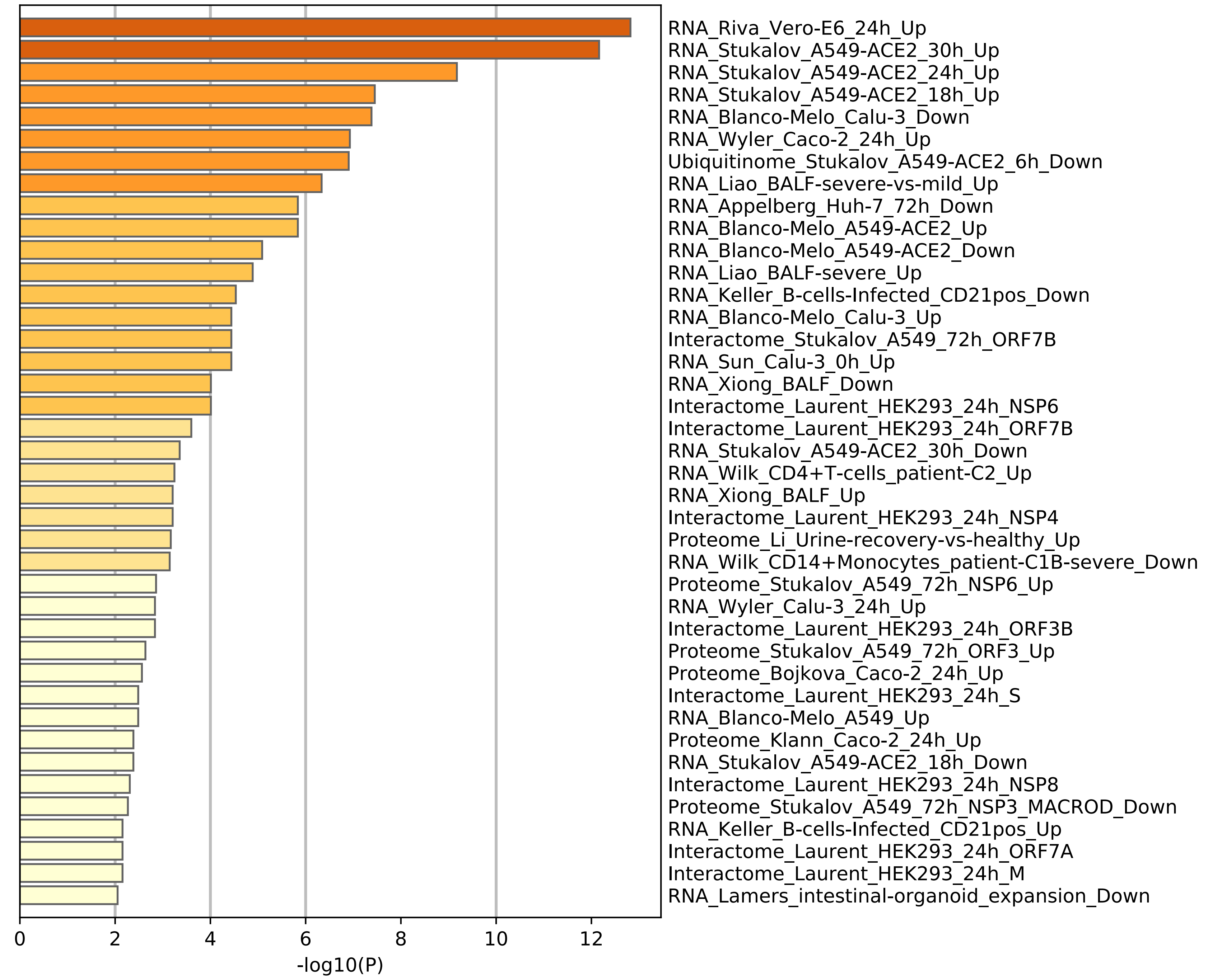

RNA_Riva_Vero-E6_24h_Up
RNA_Stukalov_A549-ACE2_30h_Up
RNA_Stukalov_A549-ACE2_24h_Up
RNA_Stukalov_A549-ACE2_18h_Up
RNA_Blanco-Melo_Calu-3_Down
RNA_Wyler_Caco-2_24h_Up
Ubiquitinome_Stukalov_A549-ACE2_6h_Down
RNA_Liao_BALF-severe-vs-mild_Up
RNA_Appelberg_Huh-7_72h_Down
RNA_Blanco-Melo_A549-ACE2_Up
RNA_Blanco-Melo_A549-ACE2_Down
RNA_Liao_BALF-severe_Up
RNA_Keller_B-cells-Infected_CD21pos_Down
RNA_Blanco-Melo_Calu-3_Up
Interactome_Stukalov_A549_72h_ORF7B
RNA_Sun_Calu-3_0h_Up
RNA_Xiong_BALF_Down
Interactome_Laurent_HEK293_24h_NSP6
Interactome_Laurent_HEK293_24h_ORF7B
RNA_Stukalov_A549-ACE2_30h_Down
RNA_Wilk_CD4+T-cells_patient-C2_Up
RNA_Xiong_BALF_Up
Interactome_Laurent_HEK293_24h_NSP4
Proteome_Li_Urine-recovery-vs-healthy_Up
RNA_Wilk_CD14+Monocytes_patient-C1B-severe_Down
Proteome_Stukalov_A549_72h_NSP6_Up
RNA_Wyler_Calu-3_24h_Up
Interactome_Laurent_HEK293_24h_ORF3B
Proteome_Stukalov_A549_72h_ORF3_Up
Proteome_Bojkova_Caco-2_24h_Up
Interactome_Laurent_HEK293_24h_S
RNA_Blanco-Melo_A549_Up
Proteome_Klann_Caco-2_24h_Up
RNA_Stukalov_A549-ACE2_18h_Down
Interactome_Laurent_HEK293_24h_NSP8
Proteome_Stukalov_A549_72h_NSP3_MACROD_Down
RNA_Keller_B-cells-Infected_CD21pos_Up
Interactome_Laurent_HEK293_24h_ORF7A
Interactome_Laurent_HEK293_24h_M
RNA_Lamers_intestinal-organoid_expansion_Down
0
2
4
6
8
10
12
-log10(P)
