# Supplementary material for: Transcriptional profiling of Hutchinson-Gilford Progeria syndrome fibroblasts reveals deficits in mesenchymal stem cell commitment to differentiation related to early events in endochondral ossification
Source: eLife. 2022 Dec 29;11:e81290. doi: 10.7554/eLife.81290 (PMC9833827; doi:10.7554/eLife.81290)
Supplement: Table 1—source data 1. — Childhood and teenaged patient samples compared to middle aged and older adults. [file elife-81290-table1-data1.zip › Table_1_Source_Data_1/0-7_O_UP_metascape_984.zip!/0-7_O_UP_metascape_984/Enrichment_QC/HeatmapSelectedGO_DisGeNET.pdf]

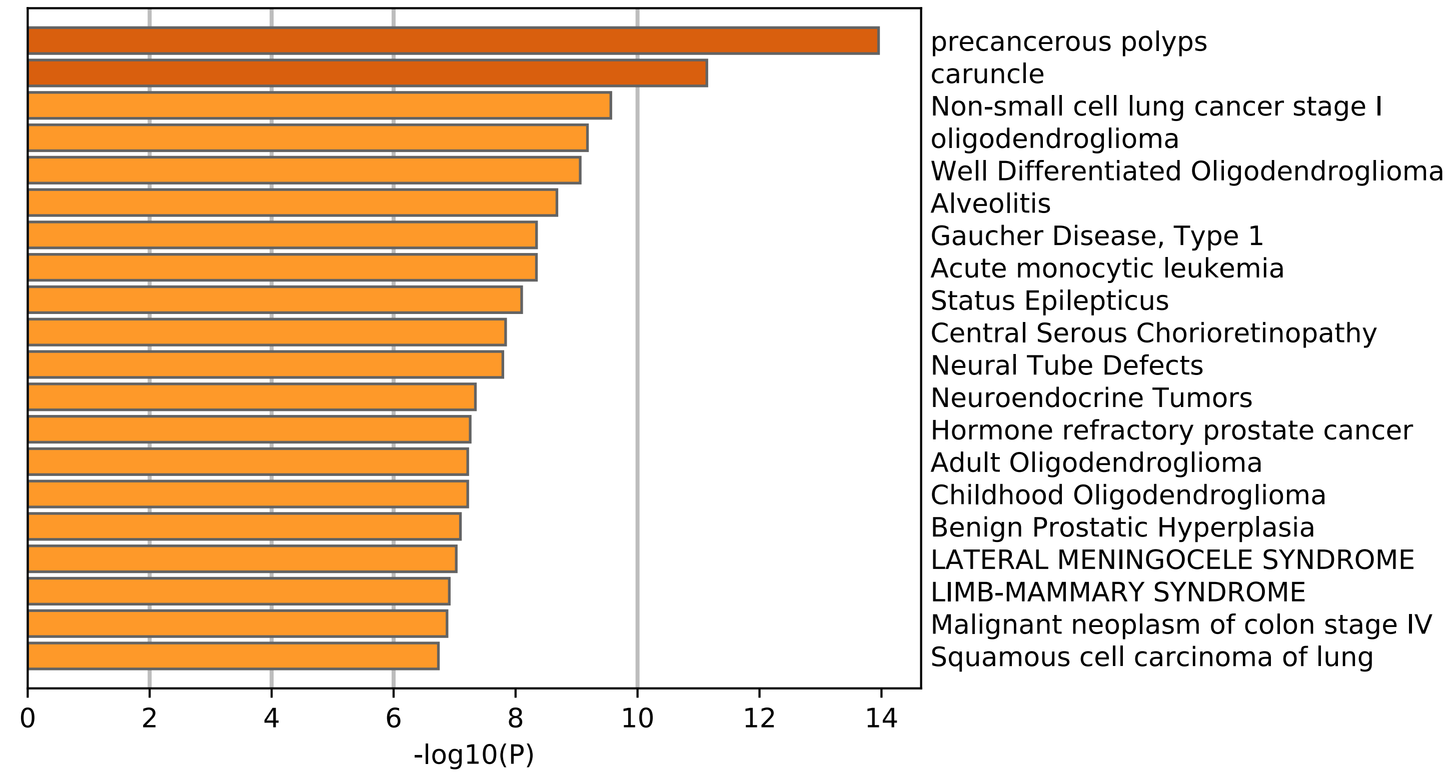
precancerous polyps
caruncle
Non-small cell lung cancer stage I
oligodendroglioma
Well Differentiated Oligodendroglioma
Alveolitis
Gaucher Disease, Type 1
Acute monocytic leukemia
Status Epilepticus
Central Serous Chorioretinopathy
Neural Tube Defects
Neuroendocrine Tumors
Hormone refractory prostate cancer
Adult Oligodendroglioma
Childhood Oligodendroglioma
Benign Prostatic Hyperplasia
LATERAL MENINGOCELE SYNDROME
LIMB-MAMMARY SYNDROME
Malignant neoplasm of colon stage IV
Squamous cell carcinoma of lung
0
2
4
6
8
10
12
14
-log10(P)
