# Supplementary material for: Transcriptional profiling of Hutchinson-Gilford Progeria syndrome fibroblasts reveals deficits in mesenchymal stem cell commitment to differentiation related to early events in endochondral ossification
Source: eLife. 2022 Dec 29;11:e81290. doi: 10.7554/eLife.81290 (PMC9833827; doi:10.7554/eLife.81290)
Supplement: Table 1—source data 1. — Childhood and teenaged patient samples compared to middle aged and older adults. [file elife-81290-table1-data1.zip › Table_1_Source_Data_1/0-7_O_UP_metascape_984.zip!/0-7_O_UP_metascape_984/Enrichment_QC/HeatmapSelectedGO_PaGenBase.pdf]

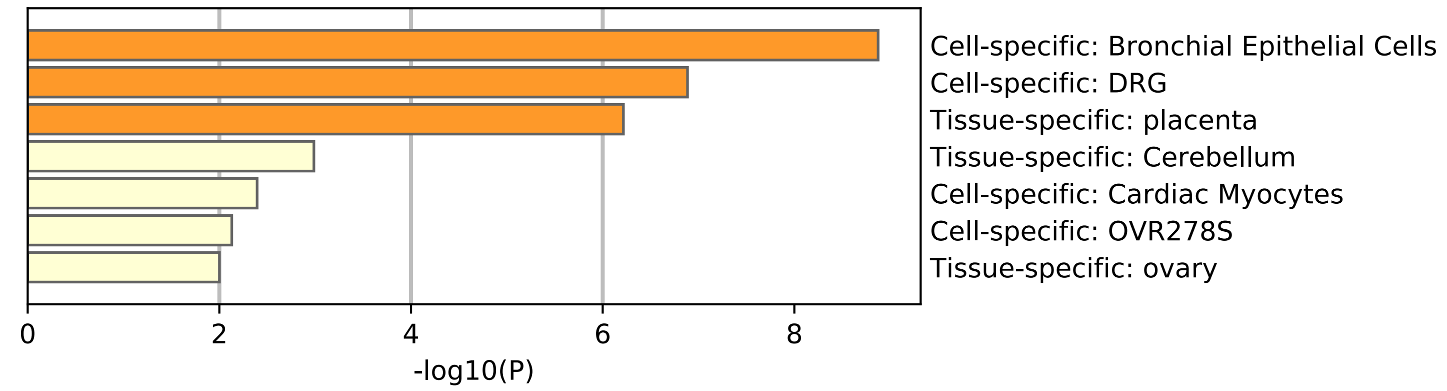

Cell-specific: Bronchial Epithelial Cells
Cell-specific: DRG
Tissue-specific: placenta
Tissue-specific: Cerebellum
Cell-specific: Cardiac Myocytes
Cell-specific: OVR278S
Tissue-specific: ovary
0
2
4
6
8
-log10(P)
