# Supplementary material for: Transcriptional profiling of Hutchinson-Gilford Progeria syndrome fibroblasts reveals deficits in mesenchymal stem cell commitment to differentiation related to early events in endochondral ossification
Source: eLife. 2022 Dec 29;11:e81290. doi: 10.7554/eLife.81290 (PMC9833827; doi:10.7554/eLife.81290)
Supplement: Table 1—source data 1. — Childhood and teenaged patient samples compared to middle aged and older adults. [file elife-81290-table1-data1.zip › Table_1_Source_Data_1/0-7_O_UP_metascape_984.zip!/0-7_O_UP_metascape_984/Enrichment_QC/HeatmapSelectedGO_Transcription_Factor_Targets.pdf]

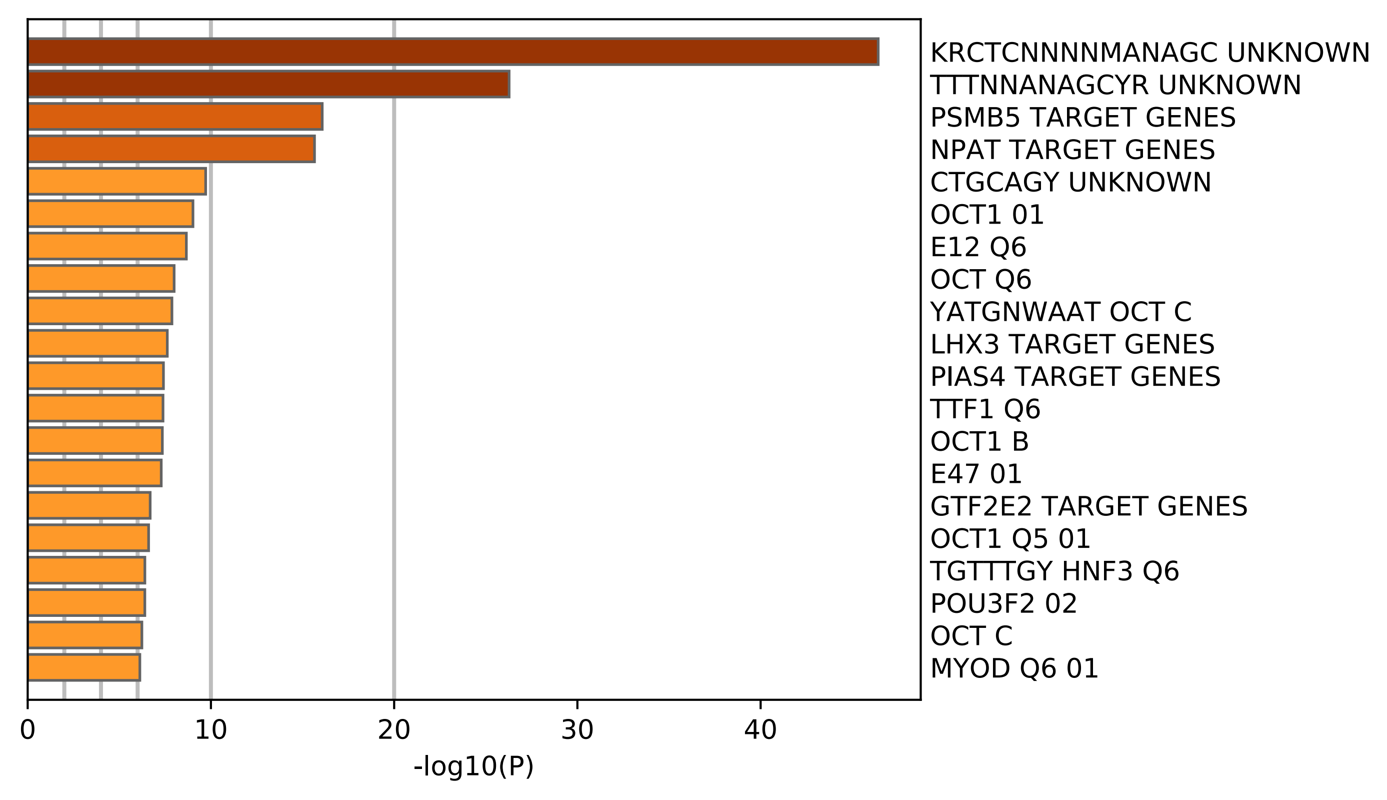
KRCTCNNNNMANAGC UNKNOWN
TTTNNANAGCYR UNKNOWN
PSMB5 TARGET GENES
NPAT TARGET GENES
CTGCAGY UNKNOWN
OCT1 01
E12 Q6
OCT Q6
YATGNWAAT OCT C
LHX3 TARGET GENES
PIAS4 TARGET GENES
TTF1 Q6
OCT1 B
E47 01
GTF2E2 TARGET GENES
OCT1 Q5 01
TGTTTGY HNF3 Q6
POU3F2 02
OCT C
MYOD Q6 01
0
10
20
30
40
-log10(P)
