# Supplementary material for: Transcriptional profiling of Hutchinson-Gilford Progeria syndrome fibroblasts reveals deficits in mesenchymal stem cell commitment to differentiation related to early events in endochondral ossification
Source: eLife. 2022 Dec 29;11:e81290. doi: 10.7554/eLife.81290 (PMC9833827; doi:10.7554/eLife.81290)
Supplement: Table 1—source data 1. — Childhood and teenaged patient samples compared to middle aged and older adults. [file elife-81290-table1-data1.zip › Table_1_Source_Data_1/0-7_O_UP_metascape_984.zip!/0-7_O_UP_metascape_984/Enrichment_QC/HeatmapSelectedGO_TRRUST.pdf]

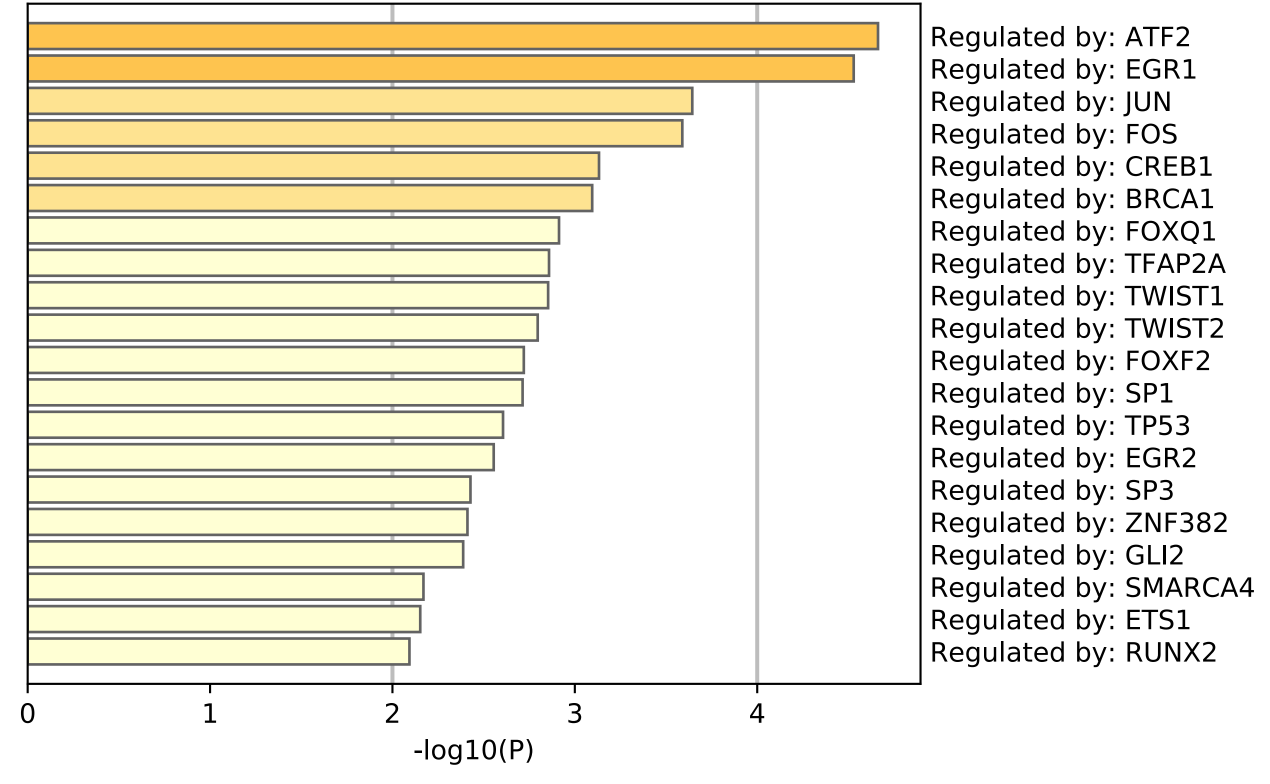
Regulated by: ATF2
Regulated by: EGR1
Regulated by: JUN
Regulated by: FOS
Regulated by: CREB1
Regulated by: BRCA1
Regulated by: FOXQ1
Regulated by: TFAP2A
Regulated by: TWIST1
Regulated by: TWIST2
Regulated by: FOXF2
Regulated by: SP1
Regulated by: TP53
Regulated by: EGR2
Regulated by: SP3
Regulated by: ZNF382
Regulated by: GLI2
Regulated by: SMARCA4
Regulated by: ETS1
Regulated by: RUNX2
0
1
2
3
4
-log10(P)
