# Supplementary material for: Transcriptional profiling of Hutchinson-Gilford Progeria syndrome fibroblasts reveals deficits in mesenchymal stem cell commitment to differentiation related to early events in endochondral ossification
Source: eLife. 2022 Dec 29;11:e81290. doi: 10.7554/eLife.81290 (PMC9833827; doi:10.7554/eLife.81290)
Supplement: Table 1—source data 1. — Childhood and teenaged patient samples compared to middle aged and older adults. [file elife-81290-table1-data1.zip › Table_1_Source_Data_1/Teen_agematched_Down81.zip!/Teen_agematched_Down81/Enrichment_GO/ColorByCluster.pdf]

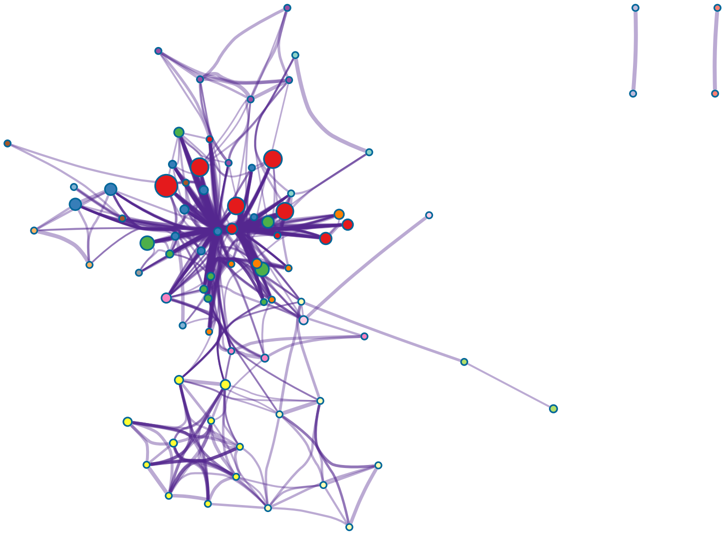

- mitotic cell cycle process
- Polo-like kinase mediated events
- nuclear division
- PID AURORA B PATHWAY
- establishment of organelle localization
- negative regulation of organelle organization
- Retinoblastoma gene in cancer
- positive regulation of transferase activity
- regulation of meiotic cell cycle
- HTLV-I infection
- Calcium regulation in cardiac cells
- sensory perception of sound
- Melanogenesis
- peptidyl-tyrosine dephosphorylation
- G1/S transition of mitotic cell cycle
- DNA repair
- regulation of proteolysis

created by
http://metascape.org
