# Supplementary material for: Transcriptional profiling of Hutchinson-Gilford Progeria syndrome fibroblasts reveals deficits in mesenchymal stem cell commitment to differentiation related to early events in endochondral ossification
Source: eLife. 2022 Dec 29;11:e81290. doi: 10.7554/eLife.81290 (PMC9833827; doi:10.7554/eLife.81290)
Supplement: Table 1—source data 1. — Childhood and teenaged patient samples compared to middle aged and older adults. [file elife-81290-table1-data1.zip › Table_1_Source_Data_1/Teen_agematched_Down81.zip!/Teen_agematched_Down81/Enrichment_heatmap/HeatmapSelectedGO.pdf]

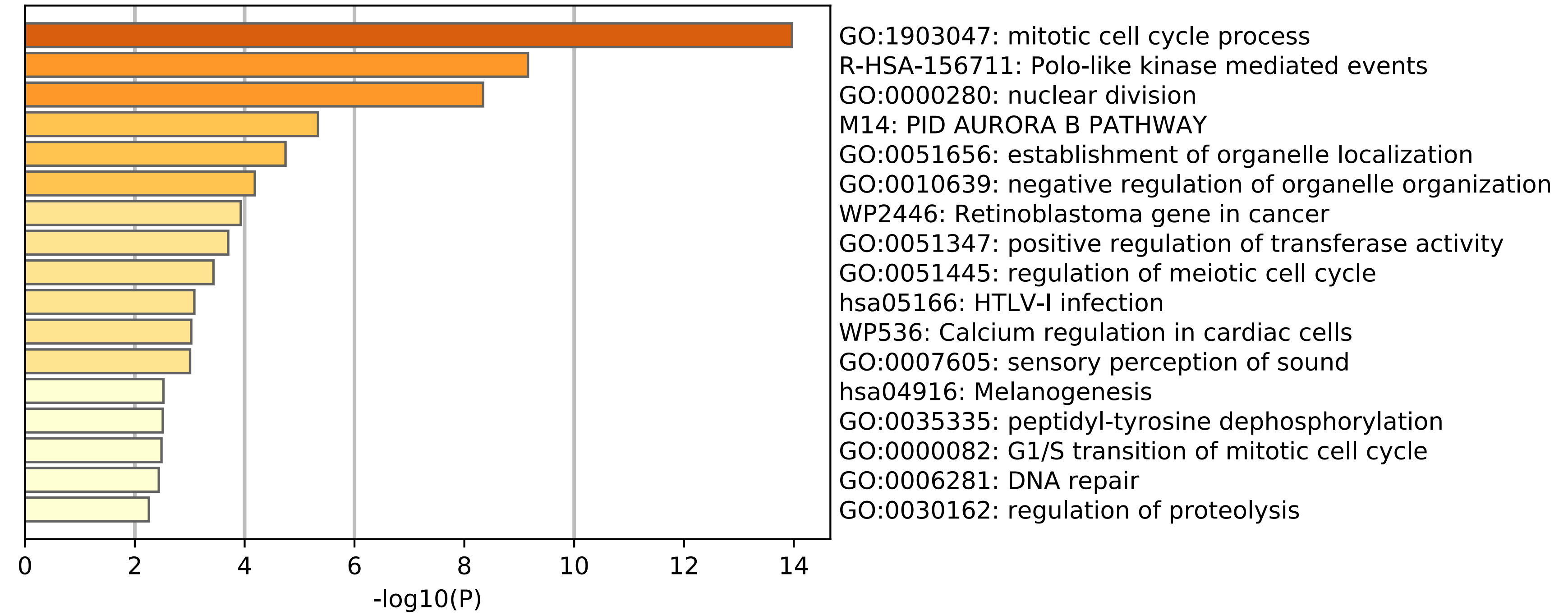
GO:1903047: mitotic cell cycle process
R-HSA-156711: Polo-like kinase mediated events
GO:0000280: nuclear division
M14: PID AURORA B PATHWAY
GO:0051656: establishment of organelle localization
GO:0010639: negative regulation of organelle organization
WP2446: Retinoblastoma gene in cancer
GO:0051347: positive regulation of transferase activity
GO:0051445: regulation of meiotic cell cycle
hsa05166: HTLV-I infection
WP536: Calcium regulation in cardiac cells
GO:0007605: sensory perception of sound
hsa04916: Melanogenesis
GO:0035335: peptidyl-tyrosine dephosphorylation
GO:0000082: G1/S transition of mitotic cell cycle
GO:0006281: DNA repair
GO:0030162: regulation of proteolysis
0
2
4
6
8
10
12
14
-log10(P)
