# Supplementary material for: Transcriptional profiling of Hutchinson-Gilford Progeria syndrome fibroblasts reveals deficits in mesenchymal stem cell commitment to differentiation related to early events in endochondral ossification
Source: eLife. 2022 Dec 29;11:e81290. doi: 10.7554/eLife.81290 (PMC9833827; doi:10.7554/eLife.81290)
Supplement: Table 1—source data 1. — Childhood and teenaged patient samples compared to middle aged and older adults. [file elife-81290-table1-data1.zip › Table_1_Source_Data_1/Teen_agematched_UP237.zip!/Teen_agematched_UP237/Enrichment_GO/ColorByCluster.pdf]

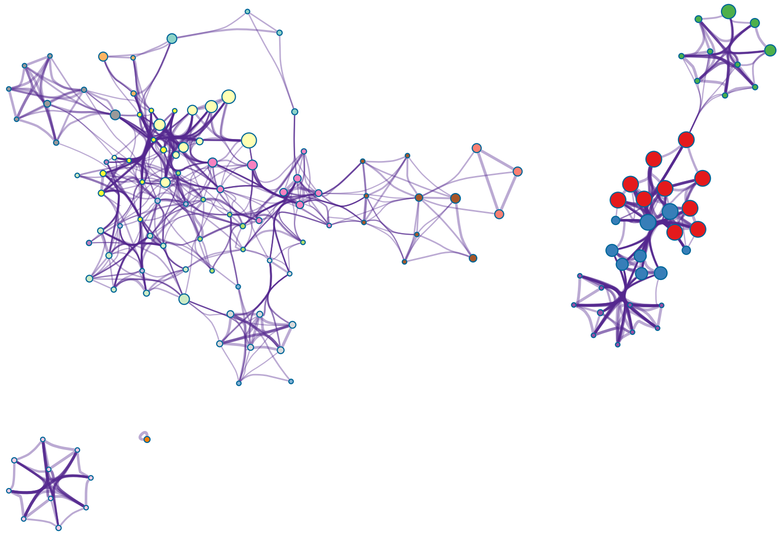

HDACs deacetylate histones
chromatin silencing
negative regulation of megakaryocyte differentiation
negative regulation of gene silencing
Genotoxicity pathway
Lung fibrosis
NABA CORE MATRISOME
Senescence and autophagy in cancer
antimicrobial humoral response
maintenance of location
blood vessel development
PID AP1 PATHWAY
extracellular matrix organization
Extracellular vesicles in the crosstalk of cardiac cells
lipid storage
maintenance of blood-brain barrier
Nop56p-associated pre-rRNA complex
cellular response to hypoxia
response to retinoic acid
response to mechanical stimulus

created by
http://metascape.org
