# Supplementary material for: Transcriptional profiling of Hutchinson-Gilford Progeria syndrome fibroblasts reveals deficits in mesenchymal stem cell commitment to differentiation related to early events in endochondral ossification
Source: eLife. 2022 Dec 29;11:e81290. doi: 10.7554/eLife.81290 (PMC9833827; doi:10.7554/eLife.81290)
Supplement: Table 1—source data 1. — Childhood and teenaged patient samples compared to middle aged and older adults. [file elife-81290-table1-data1.zip › Table_1_Source_Data_1/Teen_agematched_UP237.zip!/Teen_agematched_UP237/Enrichment_heatmap/HeatmapSelectedGO.pdf]

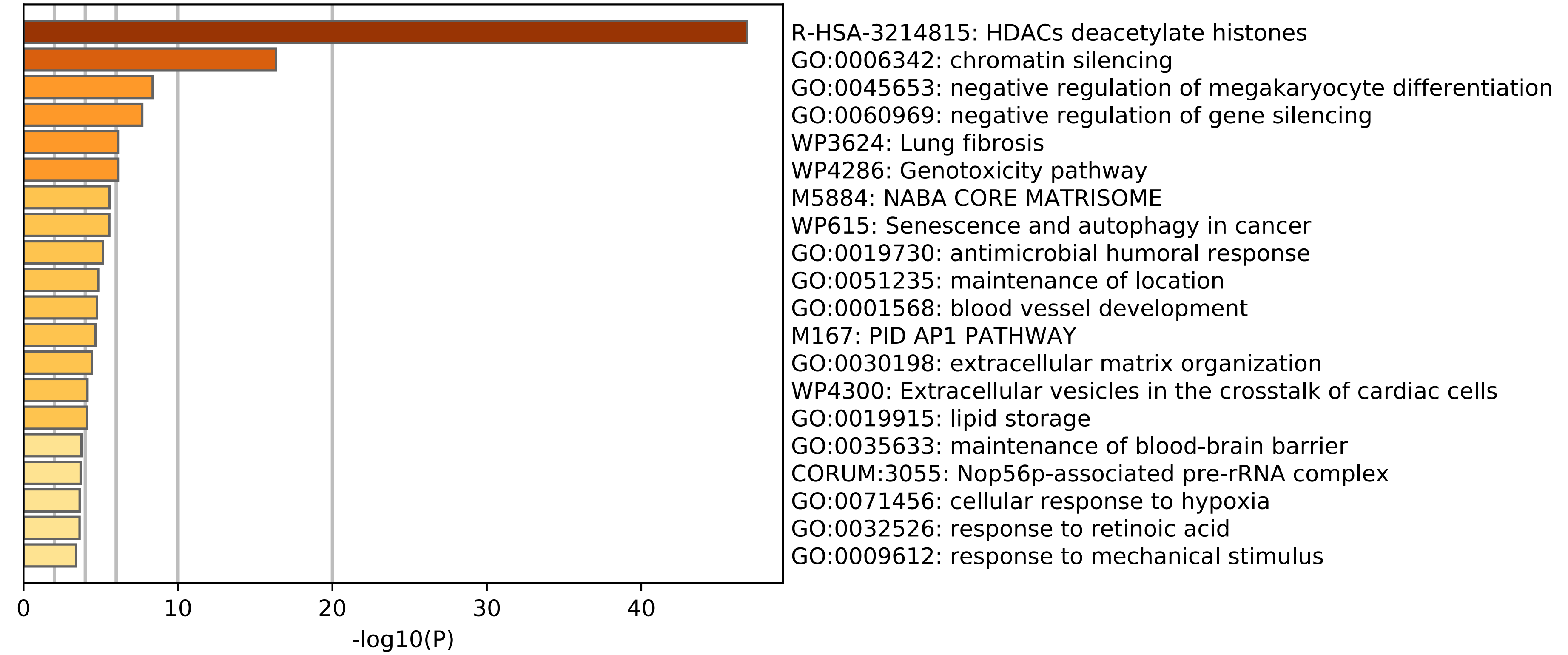
R-HSA-3214815: HDACs deacetylate histones
GO:0006342: chromatin silencing
GO:0045653: negative regulation of megakaryocyte differentiation
GO:0060969: negative regulation of gene silencing
WP3624: Lung fibrosis
WP4286: Genotoxicity pathway
M5884: NABA CORE MATRISOME
WP615: Senescence and autophagy in cancer
GO:0019730: antimicrobial humoral response
GO:0051235: maintenance of location
GO:0001568: blood vessel development
M167: PID AP1 PATHWAY
GO:0030198: extracellular matrix organization
WP4300: Extracellular vesicles in the crosstalk of cardiac cells
GO:0019915: lipid storage
GO:0035633: maintenance of blood-brain barrier
CORUM:3055: Nop56p-associated pre-rRNA complex
GO:0071456: cellular response to hypoxia
GO:0032526: response to retinoic acid
GO:0009612: response to mechanical stimulus
0
10
20
30
40
-log10(P)
