# Supplementary material for: Transcriptional profiling of Hutchinson-Gilford Progeria syndrome fibroblasts reveals deficits in mesenchymal stem cell commitment to differentiation related to early events in endochondral ossification
Source: eLife. 2022 Dec 29;11:e81290. doi: 10.7554/eLife.81290 (PMC9833827; doi:10.7554/eLife.81290)
Supplement: Table 1—source data 1. — Childhood and teenaged patient samples compared to middle aged and older adults. [file elife-81290-table1-data1.zip › Table_1_Source_Data_1/Teen_agematched_UP237.zip!/Teen_agematched_UP237/Enrichment_heatmap/HeatmapSelectedGOTop100.pdf]

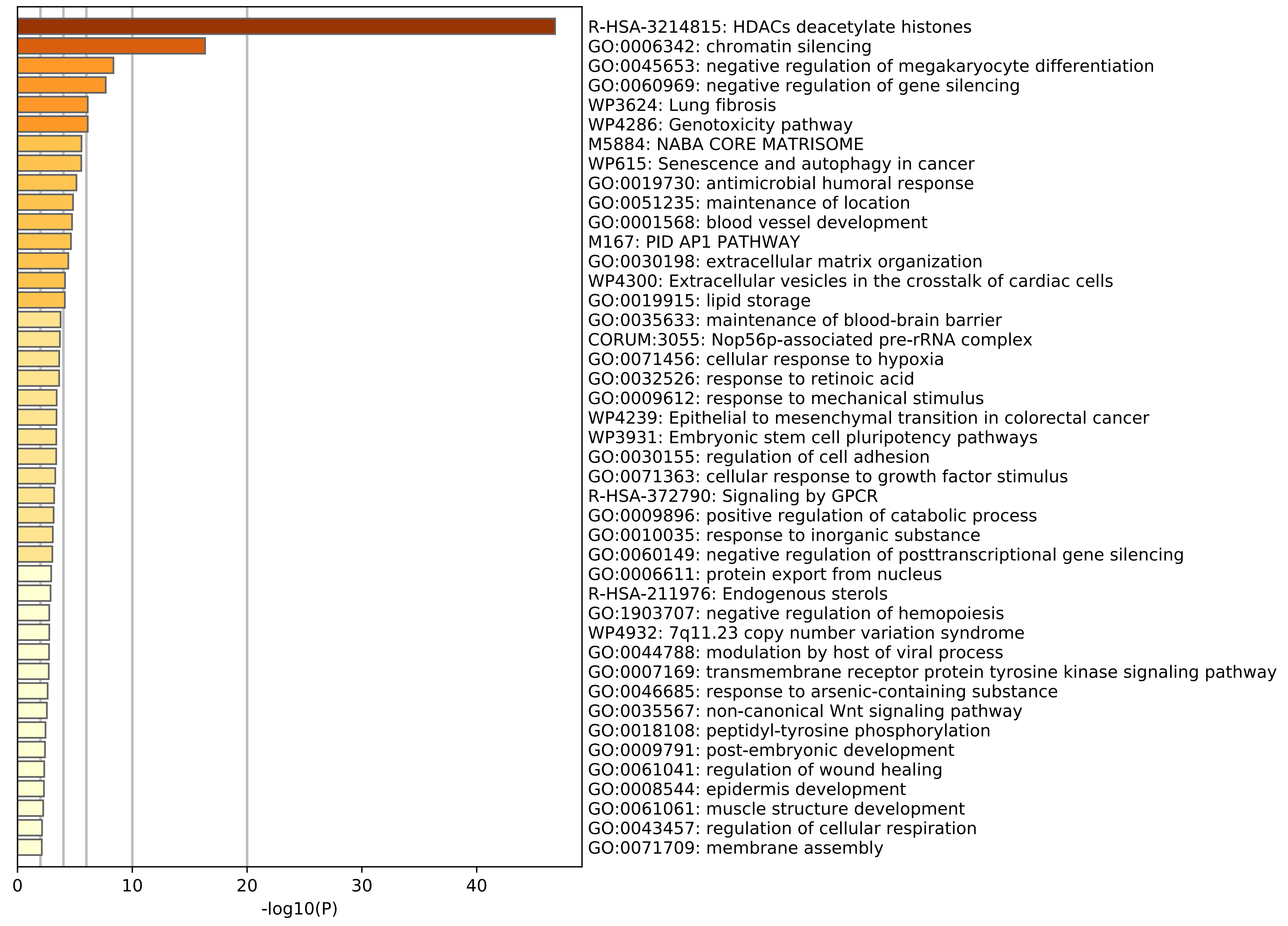
R-HSA-3214815: HDACs deacetylate histones
GO:0006342: chromatin silencing
GO:0045653: negative regulation of megakaryocyte differentiation
GO:0060969: negative regulation of gene silencing
WP3624: Lung fibrosis
WP4286: Genotoxicity pathway
M5884: NABA CORE MATRISOME
WP615: Senescence and autophagy in cancer
GO:0019730: antimicrobial humoral response
GO:0051235: maintenance of location
GO:0001568: blood vessel development
M167: PID AP1 PATHWAY
GO:0030198: extracellular matrix organization
WP4300: Extracellular vesicles in the crosstalk of cardiac cells
GO:0019915: lipid storage
GO:0035633: maintenance of blood-brain barrier
CORUM:3055: Nop56p-associated pre-rRNA complex
GO:0071456: cellular response to hypoxia
GO:0032526: response to retinoic acid
GO:0009612: response to mechanical stimulus
WP4239: Epithelial to mesenchymal transition in colorectal cancer
WP3931: Embryonic stem cell pluripotency pathways
GO:0030155: regulation of cell adhesion
GO:0071363: cellular response to growth factor stimulus
R-HSA-372790: Signaling by GPCR
GO:0009896: positive regulation of catabolic process
GO:0010035: response to inorganic substance
GO:0060149: negative regulation of posttranscriptional gene silencing
GO:0006611: protein export from nucleus
R-HSA-211976: Endogenous sterols
GO:1903707: negative regulation of hemopoiesis
WP4932: 7q11.23 copy number variation syndrome
GO:0044788: modulation by host of viral process
GO:0007169: transmembrane receptor protein tyrosine kinase signaling pathway
GO:0046685: response to arsenic-containing substance
GO:0035567: non-canonical Wnt signaling pathway
GO:0018108: peptidyl-tyrosine phosphorylation
GO:0009791: post-embryonic development
GO:0061041: regulation of wound healing
GO:0008544: epidermis development
GO:0061061: muscle structure development
GO:0043457: regulation of cellular respiration
GO:0071709: membrane assembly
0
10
20
30
40
-log10(P)
