# Supplementary material for: Transcriptional profiling of Hutchinson-Gilford Progeria syndrome fibroblasts reveals deficits in mesenchymal stem cell commitment to differentiation related to early events in endochondral ossification
Source: eLife. 2022 Dec 29;11:e81290. doi: 10.7554/eLife.81290 (PMC9833827; doi:10.7554/eLife.81290)
Supplement: Table 1—source data 1. — Childhood and teenaged patient samples compared to middle aged and older adults. [file elife-81290-table1-data1.zip › Table_1_Source_Data_1/Teen_M_DOWN_1138.zip!/Teen_M_DOWN_1138/Enrichment_GO/ColorByCluster.pdf]

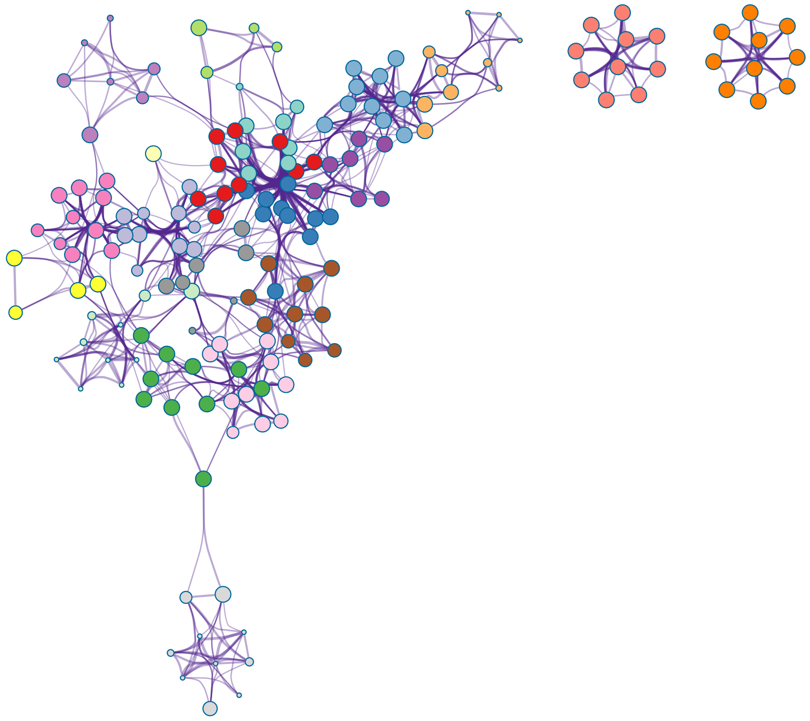

- Cell Cycle
- mitotic cell cycle process
- cellular response to DNA damage stimulus
- microtubule cytoskeleton organization
- mRNA metabolic process
- DNA replication
- meiotic cell cycle
- Retinoblastoma gene in cancer
- positive regulation of cell cycle process
- metaphase plate congression
- PID PLK1 PATHWAY
- Cell cycle
- histone modification
- G2/M Transition
- centrosome cycle
- protein localization to chromosome
- regulation of cell cycle G2/M phase transition
- PID AURORA B PATHWAY
- CEN complex
- sister chromatid cohesion

created by
http://metascape.org
