# Supplementary material for: Transcriptional profiling of Hutchinson-Gilford Progeria syndrome fibroblasts reveals deficits in mesenchymal stem cell commitment to differentiation related to early events in endochondral ossification
Source: eLife. 2022 Dec 29;11:e81290. doi: 10.7554/eLife.81290 (PMC9833827; doi:10.7554/eLife.81290)
Supplement: Table 1—source data 1. — Childhood and teenaged patient samples compared to middle aged and older adults. [file elife-81290-table1-data1.zip › Table_1_Source_Data_1/Teen_M_DOWN_1138.zip!/Teen_M_DOWN_1138/Enrichment_heatmap/HeatmapSelectedGO.pdf]

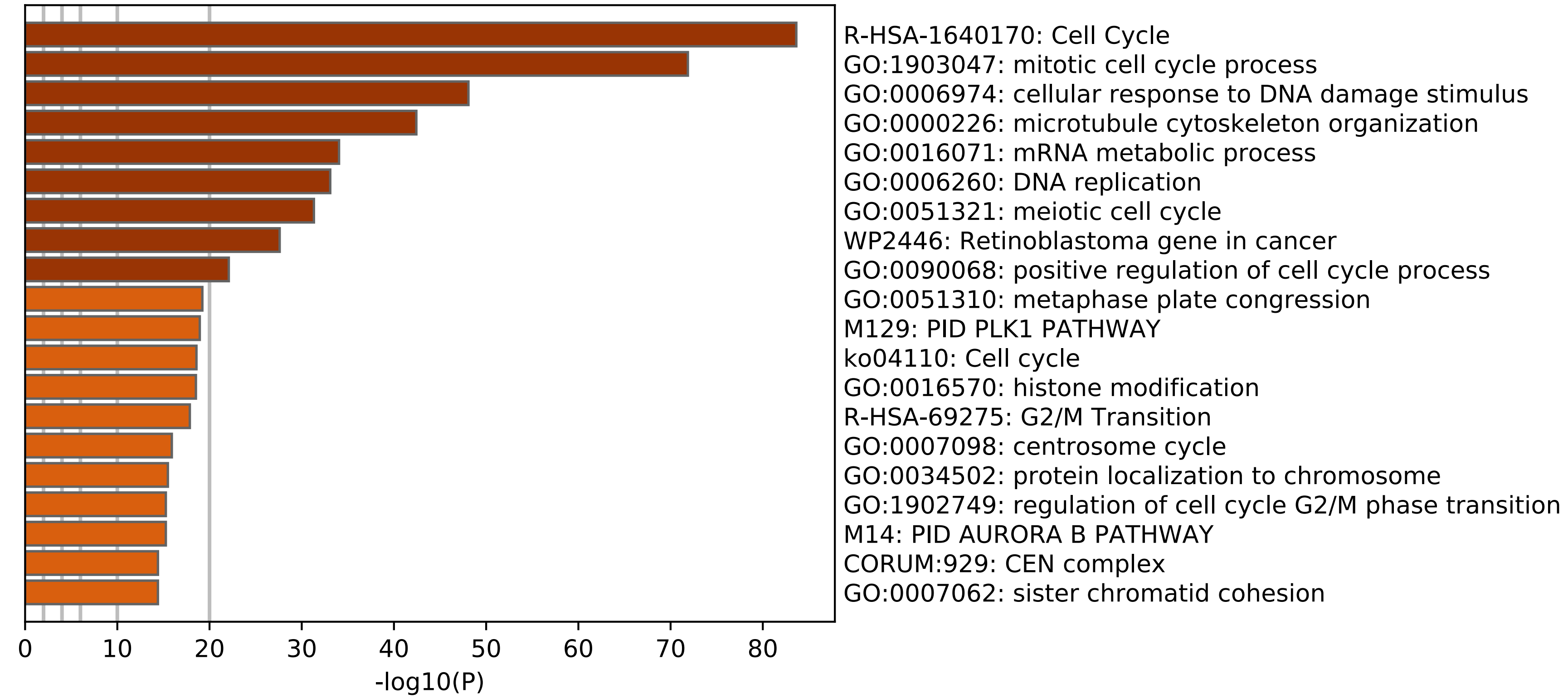

R-HSA-1640170: Cell Cycle
GO:1903047: mitotic cell cycle process
GO:0006974: cellular response to DNA damage stimulus
GO:0000226: microtubule cytoskeleton organization
GO:0016071: mRNA metabolic process
GO:0006260: DNA replication
GO:0051321: meiotic cell cycle
WP2446: Retinoblastoma gene in cancer
GO:0090068: positive regulation of cell cycle process
GO:0051310: metaphase plate congression
M129: PID PLK1 PATHWAY
ko04110: Cell cycle
GO:0016570: histone modification
R-HSA-69275: G2/M Transition
GO:0007098: centrosome cycle
GO:0034502: protein localization to chromosome
GO:1902749: regulation of cell cycle G2/M phase transition
M14: PID AURORA B PATHWAY
CORUM:929: CEN complex
GO:0007062: sister chromatid cohesion
0
10
20
30
40
50
60
70
80
-log10(P)
