# Supplementary material for: Transcriptional profiling of Hutchinson-Gilford Progeria syndrome fibroblasts reveals deficits in mesenchymal stem cell commitment to differentiation related to early events in endochondral ossification
Source: eLife. 2022 Dec 29;11:e81290. doi: 10.7554/eLife.81290 (PMC9833827; doi:10.7554/eLife.81290)
Supplement: Table 1—source data 1. — Childhood and teenaged patient samples compared to middle aged and older adults. [file elife-81290-table1-data1.zip › Table_1_Source_Data_1/Teen_M_DOWN_1138.zip!/Teen_M_DOWN_1138/Enrichment_heatmap/HeatmapSelectedGOTop100.pdf]

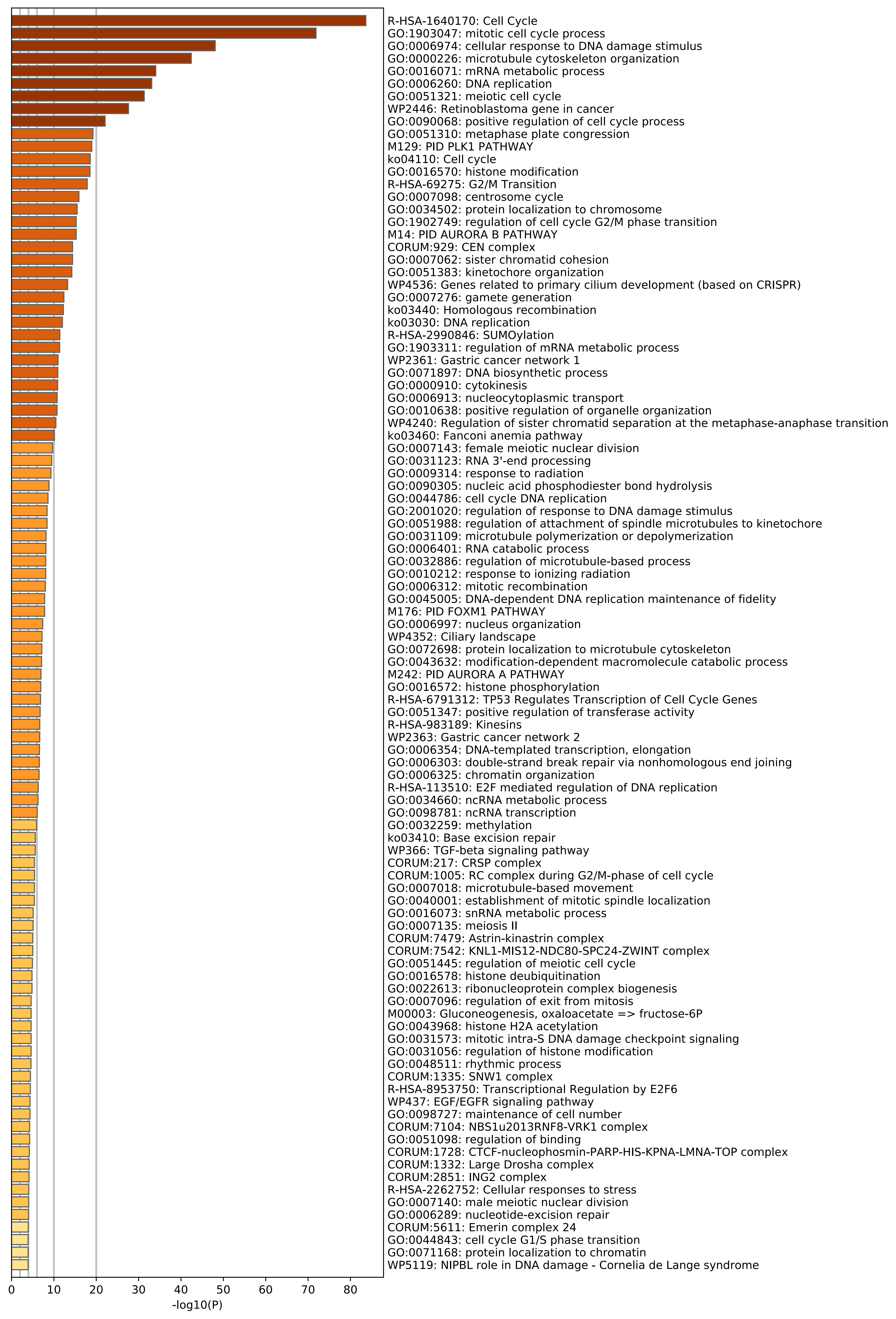
R-HSA-1640170: Cell Cycle
GO:1903047: mitotic cell cycle process
GO:0006974: cellular response to DNA damage stimulus
GO:0000226: microtubule cytoskeleton organization
GO:0016071: mRNA metabolic process
GO:0006260: DNA replication
GO:0051321: meiotic cell cycle
WP2446: Retinoblastoma gene in cancer
GO:0090068: positive regulation of cell cycle process
GO:0051310: metaphase plate congression
M129: PID PLK1 PATHWAY
ko04110: Cell cycle
GO:0016570: histone modification
R-HSA-69275: G2/M Transition
GO:0007098: centrosome cycle
GO:0034502: protein localization to chromosome
GO:1902749: regulation of cell cycle G2/M phase transition
M14: PID AURORA B PATHWAY
CORUM:929: CEN complex
GO:0007062: sister chromatid cohesion
GO:0051383: kinetochore organization
WP4536: Genes related to primary cilium development (based on CRISPR)
GO:0007276: gamete generation
ko03440: Homologous recombination
ko03030: DNA replication
R-HSA-2990846: SUMOylation
GO:1903311: regulation of mRNA metabolic process
WP2361: Gastric cancer network 1
GO:0071897: DNA biosynthetic process
GO:0000910: cytokinesis
GO:0006913: nucleocytoplasmic transport
GO:0010638: positive regulation of organelle organization
WP4240: Regulation of sister chromatid separation at the metaphase-anaphase transition
ko03460: Fanconi anemia pathway
GO:0007143: female meiotic nuclear division
GO:0031123: RNA 3'-end processing
GO:0009314: response to radiation
GO:0090305: nucleic acid phosphodiester bond hydrolysis
GO:0044786: cell cycle DNA replication
GO:2001020: regulation of response to DNA damage stimulus
GO:0051988: regulation of attachment of spindle microtubules to kinetochore
GO:0031109: microtubule polymerization or depolymerization
GO:0006401: RNA catabolic process
GO:0032886: regulation of microtubule-based process
GO:0010212: response to ionizing radiation
GO:0006312: mitotic recombination
GO:0045005: DNA-dependent DNA replication maintenance of fidelity
M176: PID FOXM1 PATHWAY
GO:0006997: nucleus organization
WP4352: Ciliary landscape
GO:0072698: protein localization to microtubule cytoskeleton
GO:0043632: modification-dependent macromolecule catabolic process
M242: PID AURORA A PATHWAY
GO:0016572: histone phosphorylation
R-HSA-6791312: TP53 Regulates Transcription of Cell Cycle Genes
GO:0051347: positive regulation of transferase activity
R-HSA-983189: Kinesins
WP2363: Gastric cancer network 2
GO:0006354: DNA-templated transcription, elongation
GO:0006303: double-strand break repair via nonhomologous end joining
GO:0006325: chromatin organization
R-HSA-113510: E2F mediated regulation of DNA replication
GO:0034660: ncRNA metabolic process
GO:0098781: ncRNA transcription
GO:0032259: methylation
ko03410: Base excision repair
WP366: TGF-beta signaling pathway
CORUM:217: CRSP complex
CORUM:1005: RC complex during G2/M-phase of cell cycle
GO:0007018: microtubule-based movement
GO:0040001: establishment of mitotic spindle localization
GO:0016073: snRNA metabolic process
GO:0007135: meiosis II
CORUM:7479: Astrin-kinastrin complex
CORUM:7542: KNL1-MIS12-NDC80-SPC24-ZWINT complex
GO:0051445: regulation of meiotic cell cycle
GO:0016578: histone deubiquitination
GO:0022613: ribonucleoprotein complex biogenesis
GO:0007096: regulation of exit from mitosis
M00003: Gluconeogenesis, oxaloacetate => fructose-6P
GO:0043968: histone H2A acetylation
GO:0031573: mitotic intra-S DNA damage checkpoint signaling
GO:0031056: regulation of histone modification
GO:0048511: rhythmic process
CORUM:1335: SNW1 complex
R-HSA-8953750: Transcriptional Regulation by E2F6
WP437: EGF/EGFR signaling pathway
GO:0098727: maintenance of cell number
CORUM:7104: NBS1u2013RNF8-VRK1 complex
GO:0051098: regulation of binding
CORUM:1728: CTCF-nucleophosmin-PARP-HIS-KPNA-LMNA-TOP complex
CORUM:1332: Large Drosha complex
CORUM:2851: ING2 complex
R-HSA-2262752: Cellular responses to stress
GO:0007140: male meiotic nuclear division
GO:0006289: nucleotide-excision repair
CORUM:5611: Emerin complex 24
GO:0044843: cell cycle G1/S phase transition
GO:0071168: protein localization to chromatin
WP5119: NIPBL role in DNA damage - Cornelia de Lange syndrome
0
10
20
30
40
50
60
70
80
-log10(P)
