# Supplementary material for: Transcriptional profiling of Hutchinson-Gilford Progeria syndrome fibroblasts reveals deficits in mesenchymal stem cell commitment to differentiation related to early events in endochondral ossification
Source: eLife. 2022 Dec 29;11:e81290. doi: 10.7554/eLife.81290 (PMC9833827; doi:10.7554/eLife.81290)
Supplement: Table 1—source data 1. — Childhood and teenaged patient samples compared to middle aged and older adults. [file elife-81290-table1-data1.zip › Table_1_Source_Data_1/Teen_M_UP_1873.zip!/Teen_M_UP_1873/Enrichment_GO/ColorByCluster.pdf]

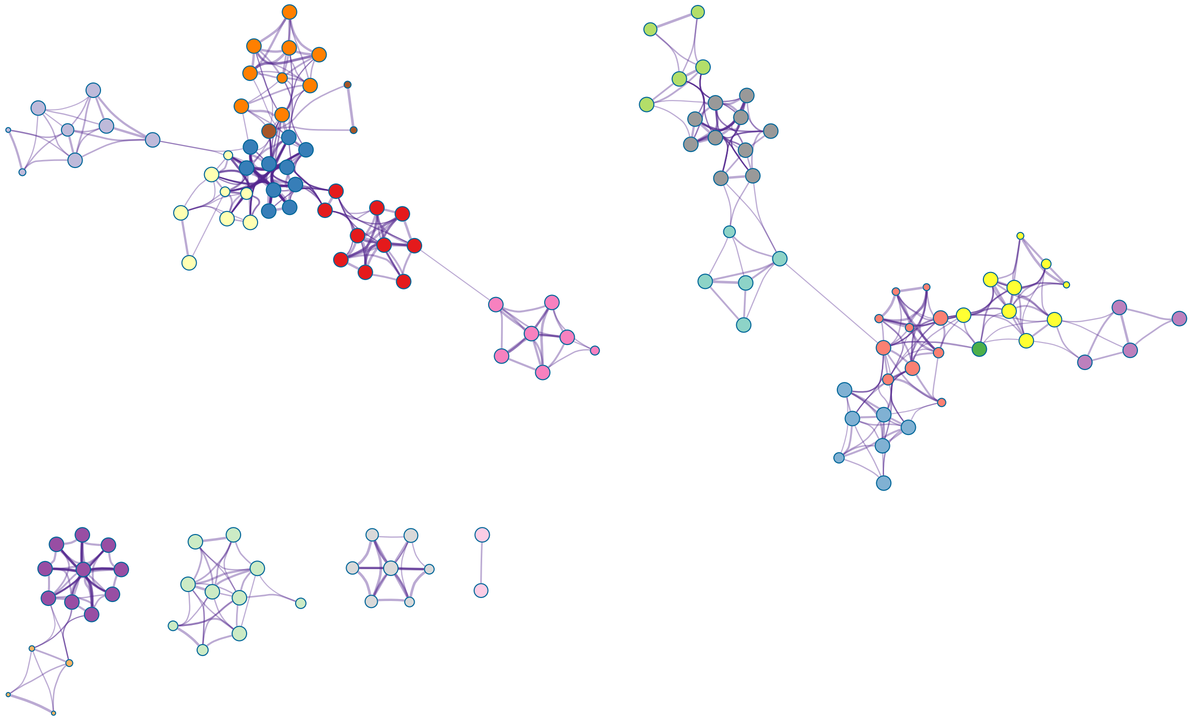

Cellular responses to stress
HDACs deacetylate histones
mitochondrion organization
ATP metabolic process
Deubiquitination
mitochondrial transport
regulation of gene silencing
ncRNA metabolic process
proteolysis involved in cellular protein catabolic proce
autophagy
Effect of progerin on genes involved in Hutchinson-Gi
regulation of myeloid cell differentiation
mitochondrial membrane organization
apoptotic signaling pathway
Respiratory chain complex I (lambda subunit) mitoch
Antigen processing: Ubiquitination & Proteasome deg
Transcriptional Regulation by TP53
sulfur compound metabolic process
localization within membrane
regulation of growth

created by
http://metascape.org
