# Supplementary material for: Transcriptional profiling of Hutchinson-Gilford Progeria syndrome fibroblasts reveals deficits in mesenchymal stem cell commitment to differentiation related to early events in endochondral ossification
Source: eLife. 2022 Dec 29;11:e81290. doi: 10.7554/eLife.81290 (PMC9833827; doi:10.7554/eLife.81290)
Supplement: Table 1—source data 1. — Childhood and teenaged patient samples compared to middle aged and older adults. [file elife-81290-table1-data1.zip › Table_1_Source_Data_1/Teen_M_UP_1873.zip!/Teen_M_UP_1873/Enrichment_heatmap/HeatmapSelectedGO.pdf]

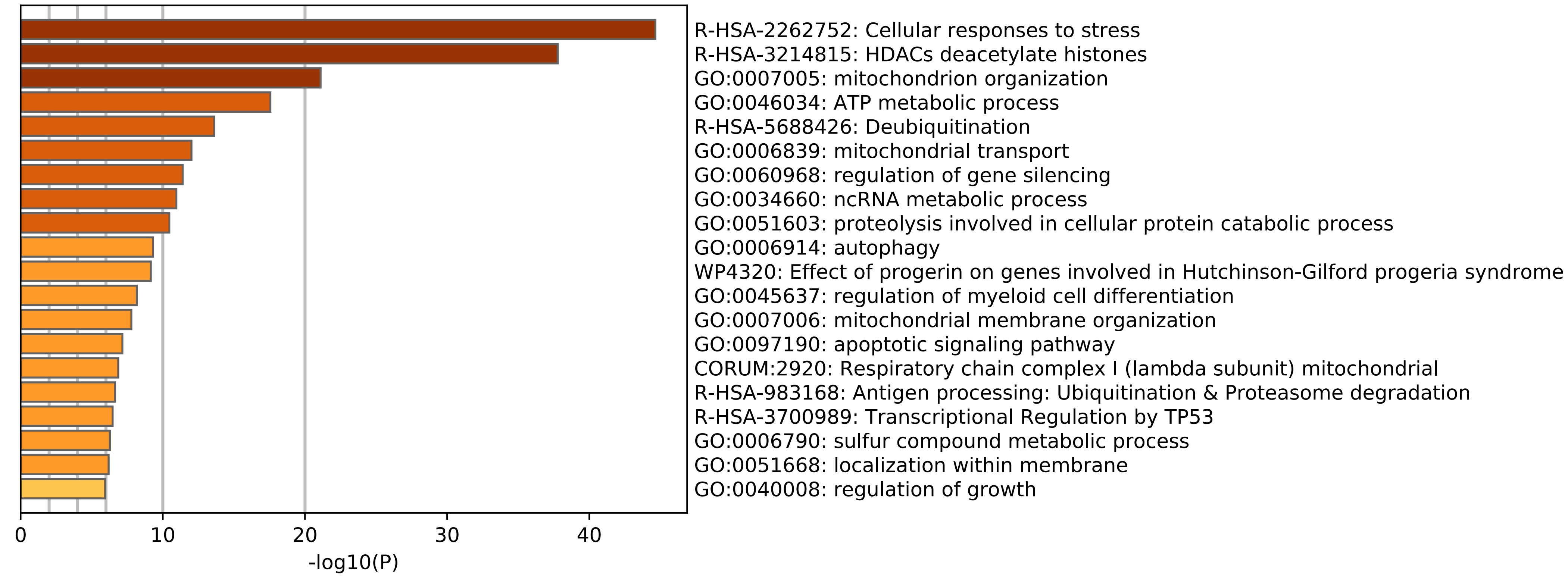
R-HSA-2262752: Cellular responses to stress
R-HSA-3214815: HDACs deacetylate histones
GO:0007005: mitochondrion organization
GO:0046034: ATP metabolic process
R-HSA-5688426: Deubiquitination
GO:0006839: mitochondrial transport
GO:0060968: regulation of gene silencing
GO:0034660: ncRNA metabolic process
GO:0051603: proteolysis involved in cellular protein catabolic process
GO:0006914: autophagy
WP4320: Effect of progerin on genes involved in Hutchinson-Gilford progeria syndrome
GO:0045637: regulation of myeloid cell differentiation
GO:0007006: mitochondrial membrane organization
GO:0097190: apoptotic signaling pathway
CORUM:2920: Respiratory chain complex I (lambda subunit) mitochondrial
R-HSA-983168: Antigen processing: Ubiquitination & Proteasome degradation
R-HSA-3700989: Transcriptional Regulation by TP53
GO:0006790: sulfur compound metabolic process
GO:0051668: localization within membrane
GO:0040008: regulation of growth
0
10
20
30
40
-log10(P)
