# Supplementary material for: Transcriptional profiling of Hutchinson-Gilford Progeria syndrome fibroblasts reveals deficits in mesenchymal stem cell commitment to differentiation related to early events in endochondral ossification
Source: eLife. 2022 Dec 29;11:e81290. doi: 10.7554/eLife.81290 (PMC9833827; doi:10.7554/eLife.81290)
Supplement: Table 1—source data 1. — Childhood and teenaged patient samples compared to middle aged and older adults. [file elife-81290-table1-data1.zip › Table_1_Source_Data_1/Teen_M_UP_1873.zip!/Teen_M_UP_1873/Enrichment_heatmap/HeatmapSelectedGOTop100.pdf]

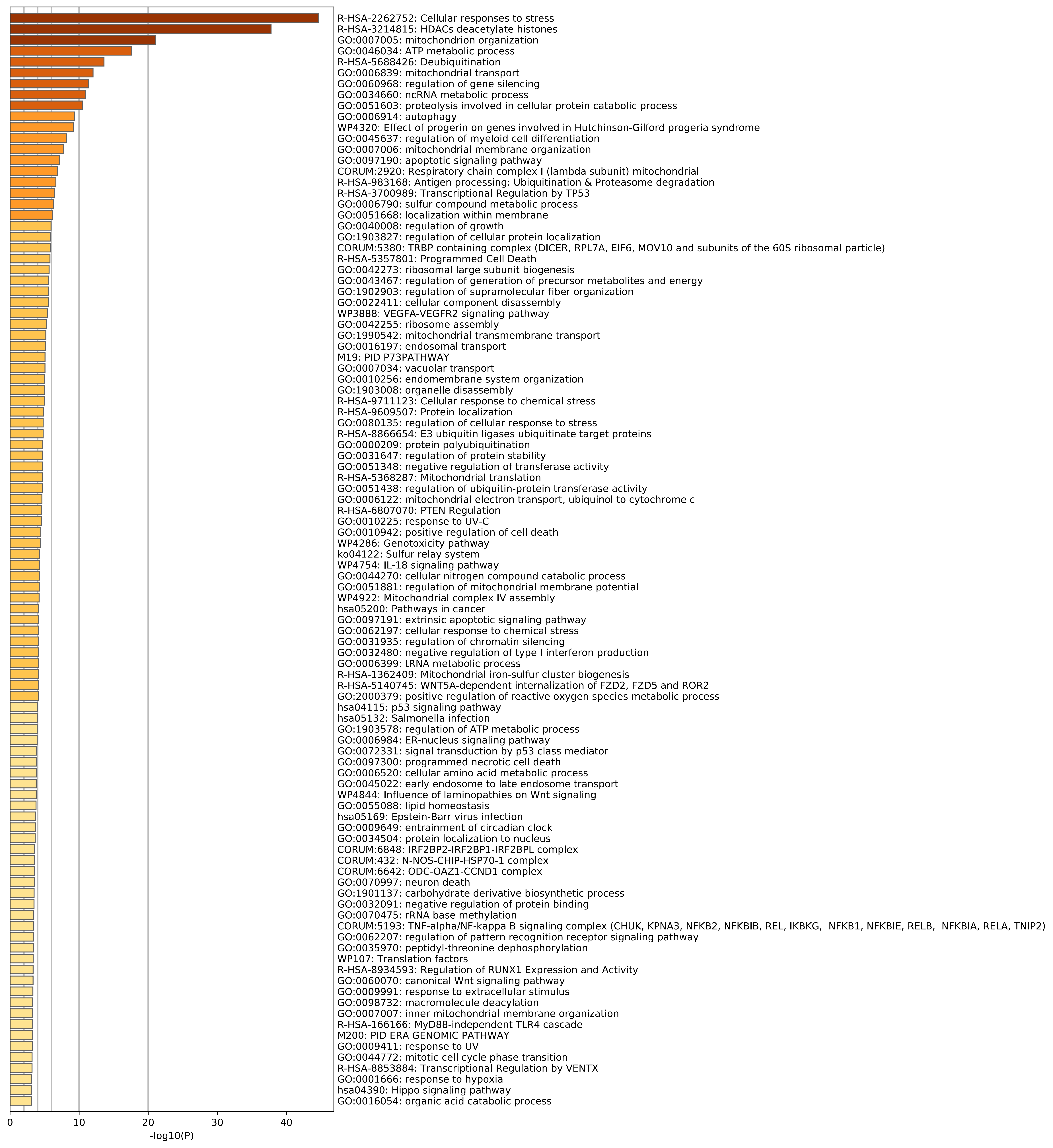
R-HSA-2262752: Cellular responses to stress
R-HSA-3214815: HDACs deacetylate histones
GO:0007005: mitochondrion organization
GO:0046034: ATP metabolic process
R-HSA-5688426: Deubiquitination
GO:0006839: mitochondrial transport
GO:0060968: regulation of gene silencing
GO:0034660: ncRNA metabolic process
GO:0051603: proteolysis involved in cellular protein catabolic process
GO:0006914: autophagy
WP4320: Effect of progerin on genes involved in Hutchinson-Gilford progeria syndrome
GO:0045637: regulation of myeloid cell differentiation
GO:0007006: mitochondrial membrane organization
GO:0097190: apoptotic signaling pathway
CORUM:2920: Respiratory chain complex I (lambda subunit) mitochondrial
R-HSA-983168: Antigen processing: Ubiquitination & Proteasome degradation
R-HSA-3700989: Transcriptional Regulation by TP53
GO:0006790: sulfur compound metabolic process
GO:0051668: localization within membrane
GO:0040008: regulation of growth
GO:1903827: regulation of cellular protein localization
CORUM:5380: TRBP containing complex (DICER, RPL7A, EIF6, MOV10 and subunits of the 60S ribosomal particle)
R-HSA-5357801: Programmed Cell Death
GO:0042273: ribosomal large subunit biogenesis
GO:0043467: regulation of generation of precursor metabolites and energy
GO:1902903: regulation of supramolecular fiber organization
GO:0022411: cellular component disassembly
WP3888: VEGFA-VEGFR2 signaling pathway
GO:0042255: ribosome assembly
GO:1990542: mitochondrial transmembrane transport
GO:0016197: endosomal transport
M19: PID P73PATHWAY
GO:0007034: vacuolar transport
GO:0010256: endomembrane system organization
GO:1903008: organelle disassembly
R-HSA-9711123: Cellular response to chemical stress
R-HSA-9609507: Protein localization
GO:0080135: regulation of cellular response to stress
R-HSA-8866654: E3 ubiquitin ligases ubiquitinate target proteins
GO:0000209: protein polyubiquitination
GO:0031647: regulation of protein stability
GO:0051348: negative regulation of transferase activity
R-HSA-5368287: Mitochondrial translation
GO:0051438: regulation of ubiquitin-protein transferase activity
GO:0006122: mitochondrial electron transport, ubiquinol to cytochrome c
R-HSA-6807070: PTEN Regulation
GO:0010225: response to UV-C
GO:0010942: positive regulation of cell death
WP4286: Genotoxicity pathway
ko04122: Sulfur relay system
WP4754: IL-18 signaling pathway
GO:0044270: cellular nitrogen compound catabolic process
GO:0051881: regulation of mitochondrial membrane potential
WP4922: Mitochondrial complex IV assembly
hsa05200: Pathways in cancer
GO:0097191: extrinsic apoptotic signaling pathway
GO:0062197: cellular response to chemical stress
GO:0031935: regulation of chromatin silencing
GO:0032480: negative regulation of type I interferon production
GO:0006399: tRNA metabolic process
R-HSA-1362409: Mitochondrial iron-sulfur cluster biogenesis
R-HSA-5140745: WNT5A-dependent internalization of FZD2, FZD5 and ROR2
GO:2000379: positive regulation of reactive oxygen species metabolic process
hsa04115: p53 signaling pathway
hsa05132: Salmonella infection
GO:1903578: regulation of ATP metabolic process
GO:0006984: ER-nucleus signaling pathway
GO:0072331: signal transduction by p53 class mediator
GO:0097300: programmed necrotic cell death
GO:0006520: cellular amino acid metabolic process
GO:0045022: early endosome to late endosome transport
WP4844: Influence of laminopathies on Wnt signaling
GO:0055088: lipid homeostasis
hsa05169: Epstein-Barr virus infection
GO:0009649: entrainment of circadian clock
GO:0034504: protein localization to nucleus
CORUM:6848: IRF2BP2-IRF2BP1-IRF2BPL complex
CORUM:432: N-NOS-CHIP-HSP70-1 complex
CORUM:6642: ODC-OAZ1-CCND1 complex
GO:0070997: neuron death
GO:1901137: carbohydrate derivative biosynthetic process
GO:0032091: negative regulation of protein binding
GO:0070475: rRNA base methylation
CORUM:5193: TNF-alpha/NF-kappa B signaling complex (CHUK, KPNA3, NFKB2, NFKBIB, REL, IKBKG, NFKB1, NFKBIE, RELB, NFKBIA, RELA, TNIP2)
GO:0062207: regulation of pattern recognition receptor signaling pathway
GO:0035970: peptidyl-threonine dephosphorylation
WP107: Translation factors
R-HSA-8934593: Regulation of RUNX1 Expression and Activity
GO:0060070: canonical Wnt signaling pathway
GO:0009991: response to extracellular stimulus
GO:0098732: macromolecule deacylation
GO:0007007: inner mitochondrial membrane organization
R-HSA-166166: MyD88-independent TLR4 cascade
M200: PID ERA GENOMIC PATHWAY
GO:0009411: response to UV
GO:0044772: mitotic cell cycle phase transition
R-HSA-8853884: Transcriptional Regulation by VENTX
GO:0001666: response to hypoxia
hsa04390: Hippo signaling pathway
GO:0016054: organic acid catabolic process
0
10
20
30
40
-log10(P)
