# Supplementary material for: Transcriptional profiling of Hutchinson-Gilford Progeria syndrome fibroblasts reveals deficits in mesenchymal stem cell commitment to differentiation related to early events in endochondral ossification
Source: eLife. 2022 Dec 29;11:e81290. doi: 10.7554/eLife.81290 (PMC9833827; doi:10.7554/eLife.81290)
Supplement: Table 1—source data 1. — Childhood and teenaged patient samples compared to middle aged and older adults. [file elife-81290-table1-data1.zip › Table_1_Source_Data_1/Teen_O_DOWN_1022.zip!/Teen_O_DOWN_1022/Enrichment_GO/ColorByCluster.pdf]

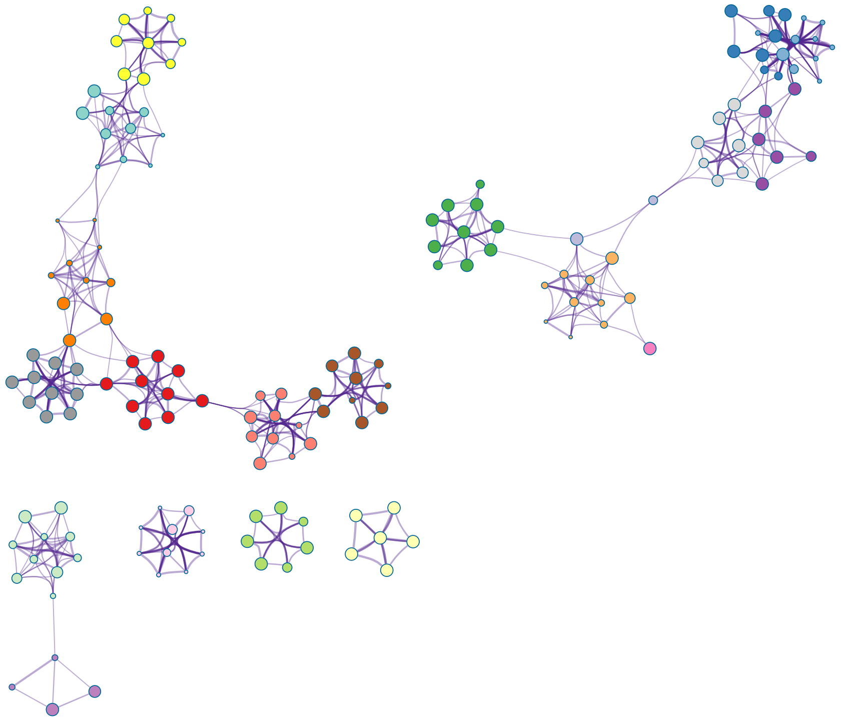

mRNA metabolic process
cellular response to DNA damage stimulus
Genes related to primary cilium development (based
cell division
regulation of mRNA metabolic process
histone modification
nucleocytoplasmic transport
positive regulation of organelle organization
mRNA catabolic process
macromolecule methylation
proteolysis involved in cellular protein catabolic proce
microtubule cytoskeleton organization
mRNA transport
DNA Double-Strand Break Repair
centriole replication
gamete generation
PPARA activates gene expression
organelle fission
EGF/EGFR signaling pathway
Toll-like Receptor Cascades

created by
http://metascape.org
