# Supplementary material for: Transcriptional profiling of Hutchinson-Gilford Progeria syndrome fibroblasts reveals deficits in mesenchymal stem cell commitment to differentiation related to early events in endochondral ossification
Source: eLife. 2022 Dec 29;11:e81290. doi: 10.7554/eLife.81290 (PMC9833827; doi:10.7554/eLife.81290)
Supplement: Table 1—source data 1. — Childhood and teenaged patient samples compared to middle aged and older adults. [file elife-81290-table1-data1.zip › Table_1_Source_Data_1/Teen_O_DOWN_1022.zip!/Teen_O_DOWN_1022/Enrichment_GO/ColorByPValue.pdf]

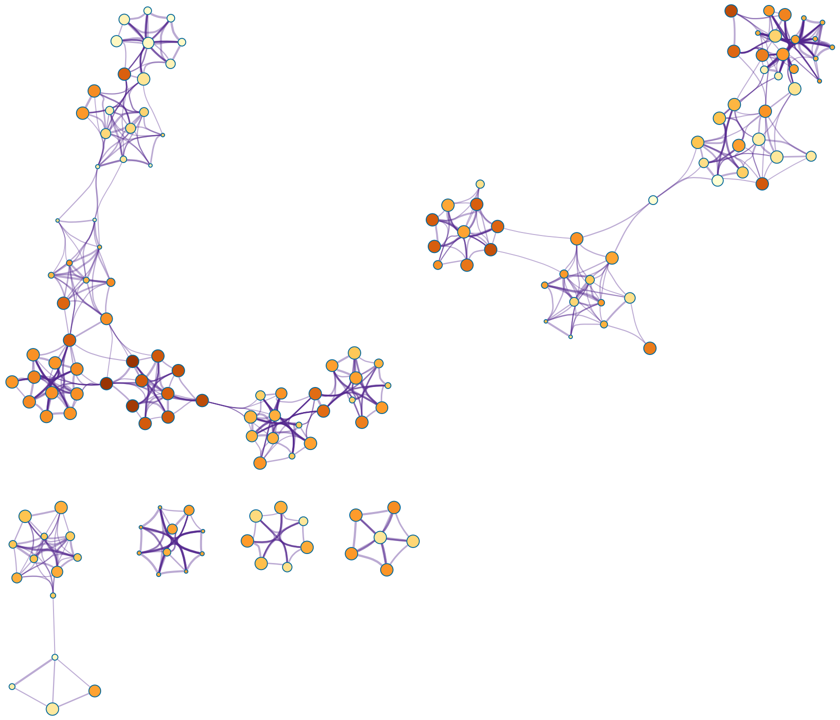

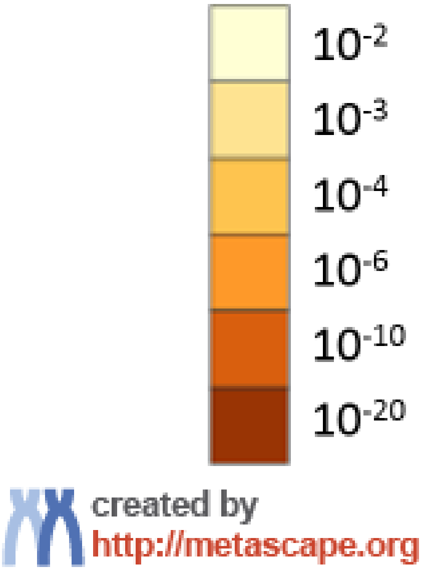
10^{-2}
10^{-3}
10^{-4}
10^{-6}
10^{-10}
10^{-20}
created by
http://metascape.org
