# Supplementary material for: Transcriptional profiling of Hutchinson-Gilford Progeria syndrome fibroblasts reveals deficits in mesenchymal stem cell commitment to differentiation related to early events in endochondral ossification
Source: eLife. 2022 Dec 29;11:e81290. doi: 10.7554/eLife.81290 (PMC9833827; doi:10.7554/eLife.81290)
Supplement: Table 1—source data 1. — Childhood and teenaged patient samples compared to middle aged and older adults. [file elife-81290-table1-data1.zip › Table_1_Source_Data_1/Teen_O_DOWN_1022.zip!/Teen_O_DOWN_1022/Enrichment_heatmap/HeatmapSelectedGO.pdf]

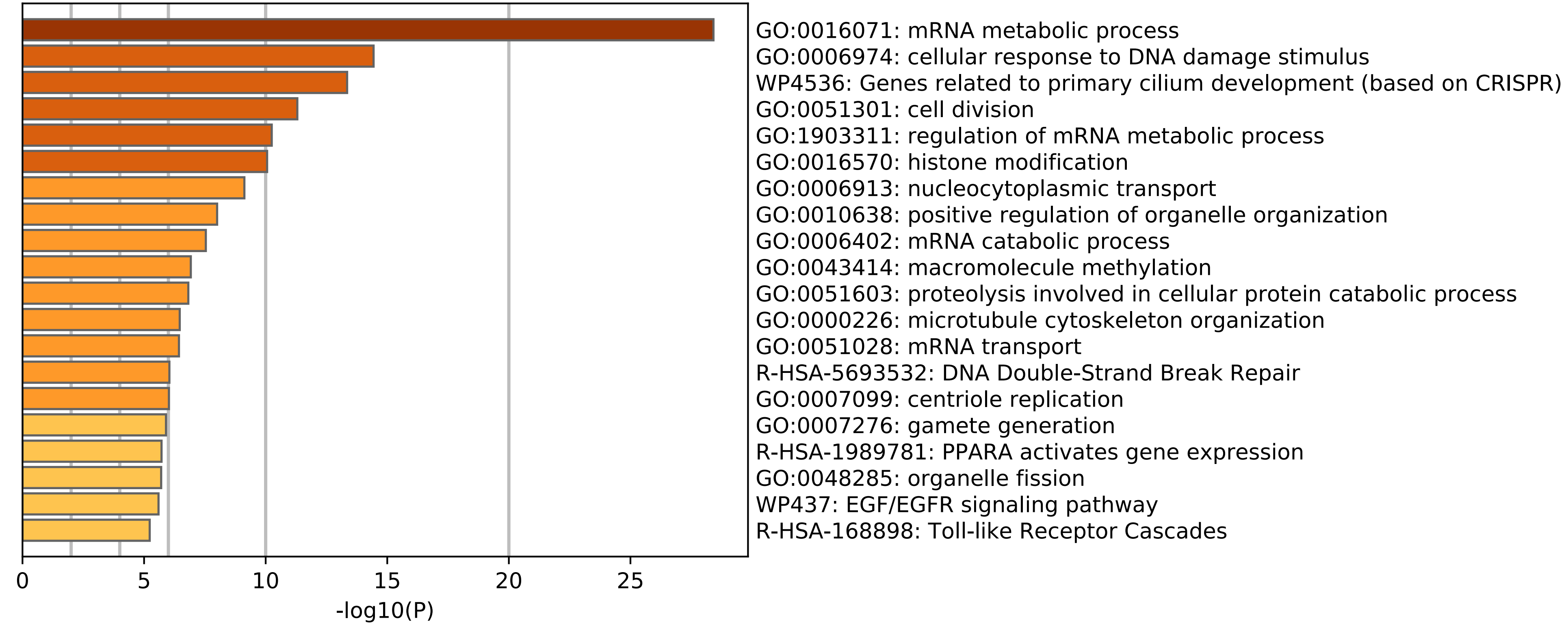
GO:0016071: mRNA metabolic process
GO:0006974: cellular response to DNA damage stimulus
WP4536: Genes related to primary cilium development (based on CRISPR)
GO:0051301: cell division
GO:1903311: regulation of mRNA metabolic process
GO:0016570: histone modification
GO:0006913: nucleocytoplasmic transport
GO:0010638: positive regulation of organelle organization
GO:0006402: mRNA catabolic process
GO:0043414: macromolecule methylation
GO:0051603: proteolysis involved in cellular protein catabolic process
GO:0000226: microtubule cytoskeleton organization
GO:0051028: mRNA transport
R-HSA-5693532: DNA Double-Strand Break Repair
GO:0007099: centriole replication
GO:0007276: gamete generation
R-HSA-1989781: PPARA activates gene expression
GO:0048285: organelle fission
WP437: EGF/EGFR signaling pathway
R-HSA-168898: Toll-like Receptor Cascades
0
5
10
15
20
25
-log10(P)
