# Supplementary material for: Transcriptional profiling of Hutchinson-Gilford Progeria syndrome fibroblasts reveals deficits in mesenchymal stem cell commitment to differentiation related to early events in endochondral ossification
Source: eLife. 2022 Dec 29;11:e81290. doi: 10.7554/eLife.81290 (PMC9833827; doi:10.7554/eLife.81290)
Supplement: Table 1—source data 1. — Childhood and teenaged patient samples compared to middle aged and older adults. [file elife-81290-table1-data1.zip › Table_1_Source_Data_1/Teen_O_DOWN_1022.zip!/Teen_O_DOWN_1022/Enrichment_heatmap/HeatmapSelectedGOTop100.pdf]

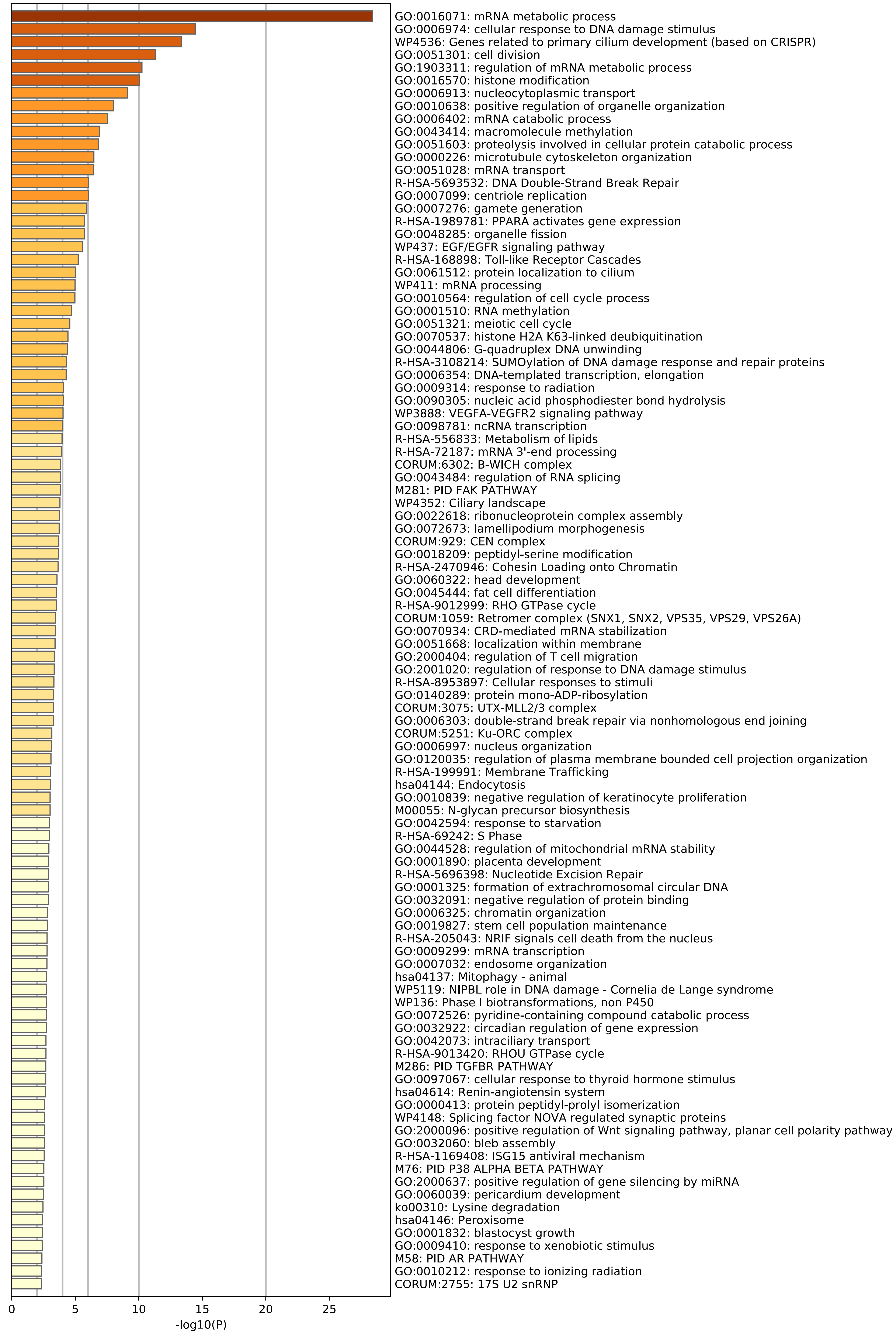
GO:0016071: mRNA metabolic process
GO:0006974: cellular response to DNA damage stimulus
WP4536: Genes related to primary cilium development (based on CRISPR)
GO:0051301: cell division
GO:1903311: regulation of mRNA metabolic process
GO:0016570: histone modification
GO:0006913: nucleocytoplasmic transport
GO:0010638: positive regulation of organelle organization
GO:0006402: mRNA catabolic process
GO:0043414: macromolecule methylation
GO:0051603: proteolysis involved in cellular protein catabolic process
GO:0000226: microtubule cytoskeleton organization
GO:0051028: mRNA transport
R-HSA-5693532: DNA Double-Strand Break Repair
GO:0007099: centriole replication
GO:0007276: gamete generation
R-HSA-1989781: PPARA activates gene expression
GO:0048285: organelle fission
WP437: EGF/EGFR signaling pathway
R-HSA-168898: Toll-like Receptor Cascades
GO:0061512: protein localization to cilium
WP411: mRNA processing
GO:0010564: regulation of cell cycle process
GO:0001510: RNA methylation
GO:0051321: meiotic cell cycle
GO:0070537: histone H2A K63-linked deubiquitination
GO:0044806: G-quadruplex DNA unwinding
R-HSA-3108214: SUMOylation of DNA damage response and repair proteins
GO:0006354: DNA-templated transcription, elongation
GO:0009314: response to radiation
GO:0090305: nucleic acid phosphodiester bond hydrolysis
WP3888: VEGFA-VEGFR2 signaling pathway
GO:0098781: ncRNA transcription
R-HSA-556833: Metabolism of lipids
R-HSA-72187: mRNA 3'-end processing
CORUM:6302: B-WICH complex
GO:0043484: regulation of RNA splicing
M281: PID FAK PATHWAY
WP4352: Ciliary landscape
GO:0022618: ribonucleoprotein complex assembly
GO:0072673: lamellipodium morphogenesis
CORUM:929: CEN complex
GO:0018209: peptidyl-serine modification
R-HSA-2470946: Cohesin Loading onto Chromatin
GO:0060322: head development
GO:0045444: fat cell differentiation
R-HSA-9012999: RHO GTPase cycle
CORUM:1059: Retromer complex (SNX1, SNX2, VPS35, VPS29, VPS26A)
GO:0070934: CRD-mediated mRNA stabilization
GO:0051668: localization within membrane
GO:2000404: regulation of T cell migration
GO:2001020: regulation of response to DNA damage stimulus
R-HSA-8953897: Cellular responses to stimuli
GO:0140289: protein mono-ADP-ribosylation
CORUM:3075: UTX-MLL2/3 complex
GO:0006303: double-strand break repair via nonhomologous end joining
CORUM:5251: Ku-ORC complex
GO:0006997: nucleus organization
GO:0120035: regulation of plasma membrane bounded cell projection organization
R-HSA-199991: Membrane Trafficking
hsa04144: Endocytosis
GO:0010839: negative regulation of keratinocyte proliferation
M00055: N-glycan precursor biosynthesis
GO:0042594: response to starvation
R-HSA-69242: S Phase
GO:0044528: regulation of mitochondrial mRNA stability
GO:0001890: placenta development
R-HSA-5696398: Nucleotide Excision Repair
GO:0001325: formation of extrachromosomal circular DNA
GO:0032091: negative regulation of protein binding
GO:0006325: chromatin organization
GO:0019827: stem cell population maintenance
R-HSA-205043: NRIF signals cell death from the nucleus
GO:0009299: mRNA transcription
GO:0007032: endosome organization
hsa04137: Mitophagy - animal
WP5119: NIPBL role in DNA damage - Cornelia de Lange syndrome
WP136: Phase I biotransformations, non P450
GO:0072526: pyridine-containing compound catabolic process
GO:0032922: circadian regulation of gene expression
GO:0042073: intraciliary transport
R-HSA-9013420: RHOU GTPase cycle
M286: PID TGFBR PATHWAY
GO:0097067: cellular response to thyroid hormone stimulus
hsa04614: Renin-angiotensin system
GO:0000413: protein peptidyl-prolyl isomerization
WP4148: Splicing factor NOVA regulated synaptic proteins
GO:2000096: positive regulation of Wnt signaling pathway, planar cell polarity pathway
GO:0032060: bleb assembly
R-HSA-1169408: ISG15 antiviral mechanism
M76: PID P38 ALPHA BETA PATHWAY
GO:2000637: positive regulation of gene silencing by miRNA
GO:0060039: pericardium development
ko00310: Lysine degradation
hsa04146: Peroxisome
GO:0001832: blastocyst growth
GO:0009410: response to xenobiotic stimulus
M58: PID AR PATHWAY
GO:0010212: response to ionizing radiation
CORUM:2755: 17S U2 snRNP
0
5
10
15
20
25
-log10(P)
