# Supplementary material for: Transcriptional profiling of Hutchinson-Gilford Progeria syndrome fibroblasts reveals deficits in mesenchymal stem cell commitment to differentiation related to early events in endochondral ossification
Source: eLife. 2022 Dec 29;11:e81290. doi: 10.7554/eLife.81290 (PMC9833827; doi:10.7554/eLife.81290)
Supplement: Table 1—source data 1. — Childhood and teenaged patient samples compared to middle aged and older adults. [file elife-81290-table1-data1.zip › Table_1_Source_Data_1/Teen-O_UP_600.zip!/Teen-O_UP_600/Enrichment_GO/ColorByCluster.pdf]

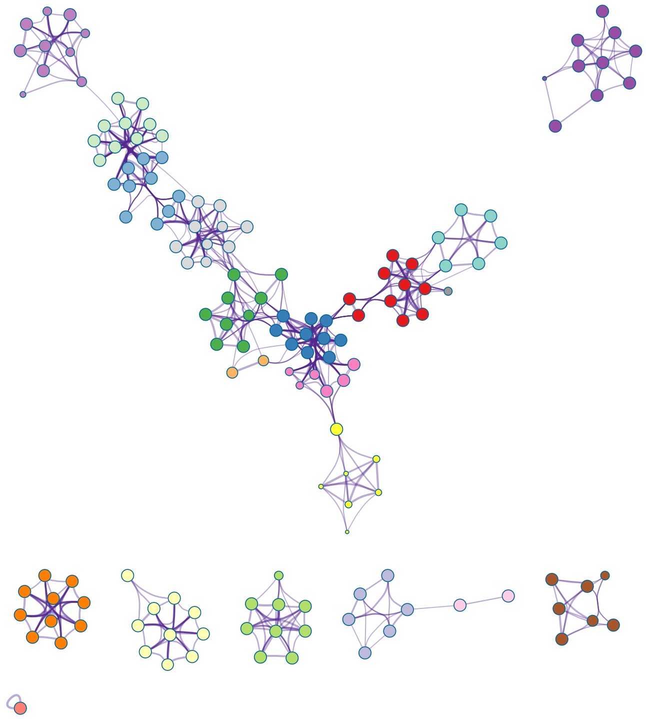

Cellular responses to stress
HDACs deacetylate histones
Deubiquitination
mitochondrion organization
Respiratory electron transport, ATP synthesis by cher
regulation of gene silencing
myeloid cell differentiation
Effect of progerin on genes involved in Hutchinson-Gi
TRBP containing complex (DICER, RPL7A, EIF6, MO
ribonucleoprotein complex biogenesis
muscle structure development
apoptotic signaling pathway
VEGFA-VEGFR2 signaling pathway
Neddylation
E3 ubiquitin ligases ubiquitinate target proteins
Wnt signaling pathway
regulation of cellular response to stress
CLEC7A (Dectin-1) signaling
IL-17 signaling pathway
regulation of protein catabolic process

created by
http://metascape.org
