# Supplementary material for: Transcriptional profiling of Hutchinson-Gilford Progeria syndrome fibroblasts reveals deficits in mesenchymal stem cell commitment to differentiation related to early events in endochondral ossification
Source: eLife. 2022 Dec 29;11:e81290. doi: 10.7554/eLife.81290 (PMC9833827; doi:10.7554/eLife.81290)
Supplement: Table 1—source data 1. — Childhood and teenaged patient samples compared to middle aged and older adults. [file elife-81290-table1-data1.zip › Table_1_Source_Data_1/Teen-O_UP_600.zip!/Teen-O_UP_600/Enrichment_heatmap/HeatmapSelectedGO.pdf]

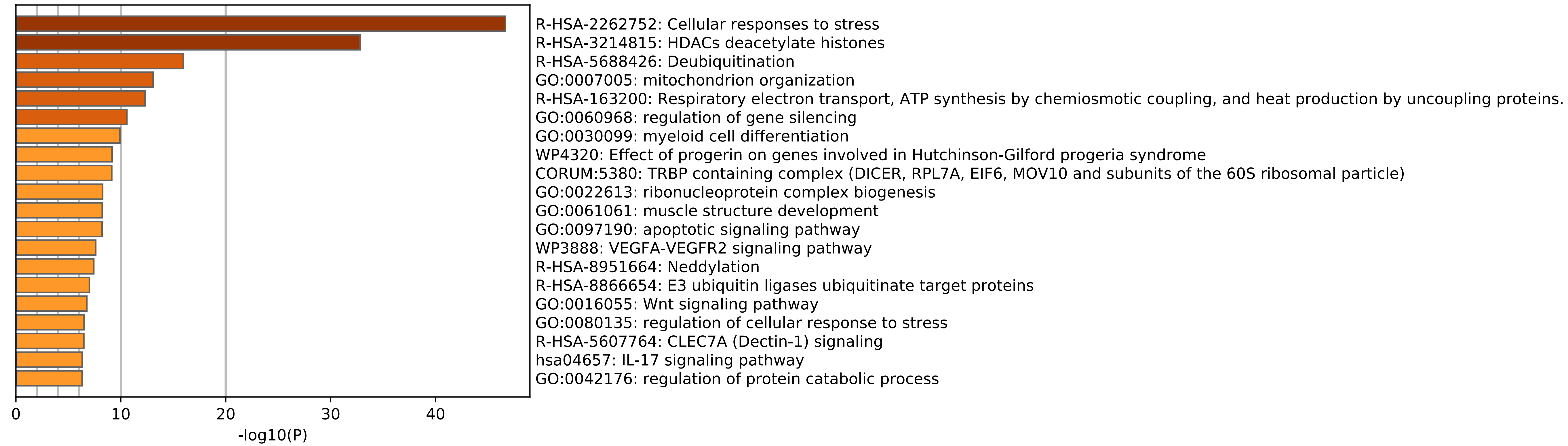

R-HSA-2262752: Cellular responses to stress
R-HSA-3214815: HDACs deacetylate histones
R-HSA-5688426: Deubiquitination
GO:0007005: mitochondrion organization
R-HSA-163200: Respiratory electron transport, ATP synthesis by chemiosmotic coupling, and heat production by uncoupling proteins.
GO:0060968: regulation of gene silencing
GO:0030099: myeloid cell differentiation
WP4320: Effect of progerin on genes involved in Hutchinson-Gilford progeria syndrome
CORUM:5380: TRBP containing complex (DICER, RPL7A, EIF6, MOV10 and subunits of the 60S ribosomal particle)
GO:0022613: ribonucleoprotein complex biogenesis
GO:0061061: muscle structure development
GO:0097190: apoptotic signaling pathway
WP3888: VEGFA-VEGFR2 signaling pathway
R-HSA-8951664: Neddylation
R-HSA-8866654: E3 ubiquitin ligases ubiquitinate target proteins
GO:0016055: Wnt signaling pathway
GO:0080135: regulation of cellular response to stress
R-HSA-5607764: CLEC7A (Dectin-1) signaling
hsa04657: IL-17 signaling pathway
GO:0042176: regulation of protein catabolic process
0
10
20
30
40
-log10(P)
