# Supplementary material for: Transcriptional profiling of Hutchinson-Gilford Progeria syndrome fibroblasts reveals deficits in mesenchymal stem cell commitment to differentiation related to early events in endochondral ossification
Source: eLife. 2022 Dec 29;11:e81290. doi: 10.7554/eLife.81290 (PMC9833827; doi:10.7554/eLife.81290)
Supplement: Table 1—source data 1. — Childhood and teenaged patient samples compared to middle aged and older adults. [file elife-81290-table1-data1.zip › Table_1_Source_Data_1/Teen-O_UP_600.zip!/Teen-O_UP_600/Enrichment_heatmap/HeatmapSelectedGOTop100.pdf]

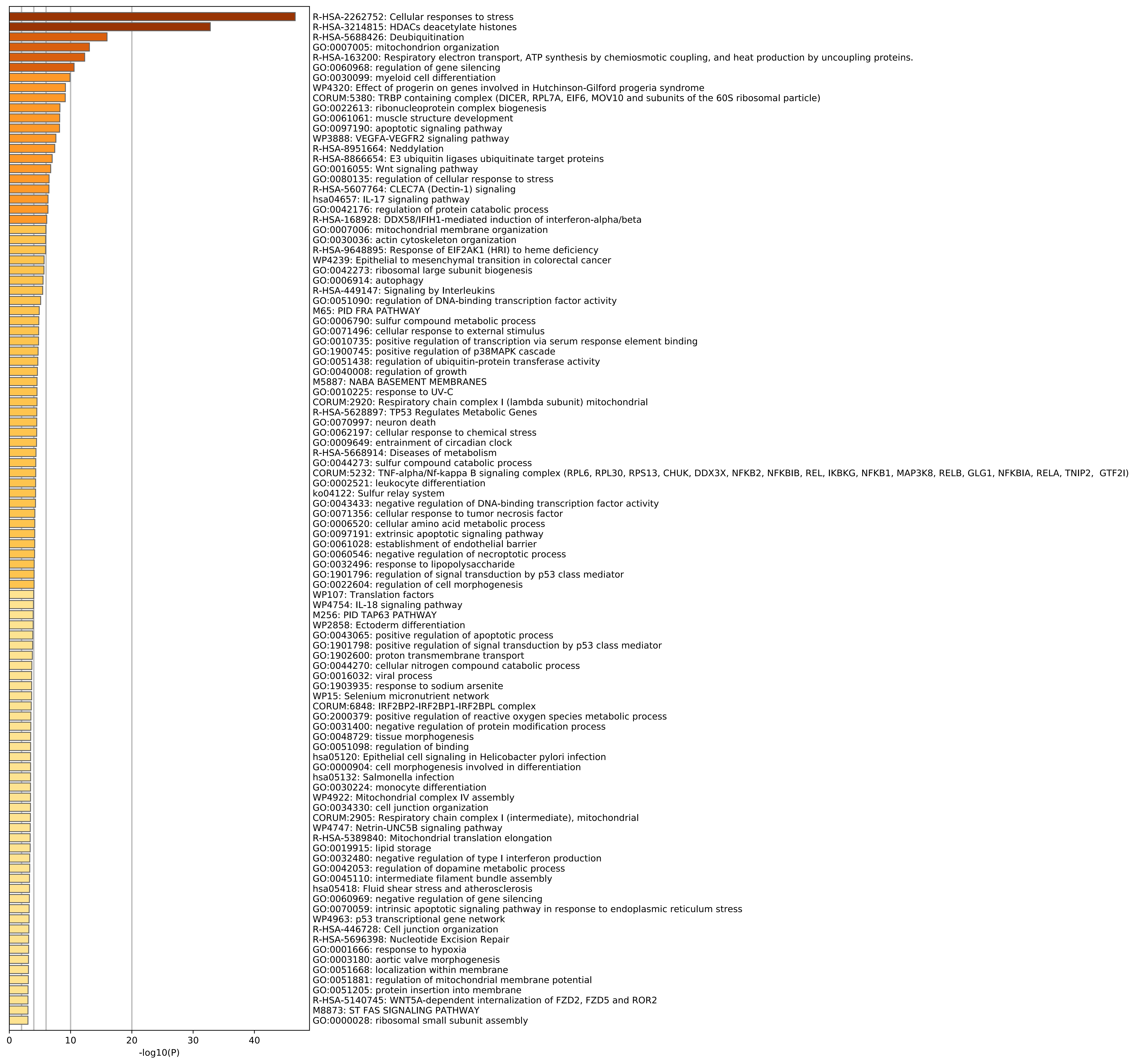
R-HSA-2262752: Cellular responses to stress
R-HSA-3214815: HDACs deacetylate histones
R-HSA-5688426: Deubiquitination
GO:0007005: mitochondrion organization
R-HSA-163200: Respiratory electron transport, ATP synthesis by chemiosmotic coupling, and heat production by uncoupling proteins.
GO:0060968: regulation of gene silencing
GO:0030099: myeloid cell differentiation
WP4320: Effect of progerin on genes involved in Hutchinson-Gilford progeria syndrome
CORUM:5380: TRBP containing complex (DICER, RPL7A, EIF6, MOV10 and subunits of the 60S ribosomal particle)
GO:0022613: ribonucleoprotein complex biogenesis
GO:0061061: muscle structure development
GO:0097190: apoptotic signaling pathway
WP3888: VEGFA-VEGFR2 signaling pathway
R-HSA-8951664: Neddylation
R-HSA-8866654: E3 ubiquitin ligases ubiquitinate target proteins
GO:0016055: Wnt signaling pathway
GO:0080135: regulation of cellular response to stress
R-HSA-5607764: CLEC7A (Dectin-1) signaling
hsa04657: IL-17 signaling pathway
GO:0042176: regulation of protein catabolic process
R-HSA-168928: DDX58/IFIH1-mediated induction of interferon-alpha/beta
GO:0007006: mitochondrial membrane organization
GO:0030036: actin cytoskeleton organization
R-HSA-9648895: Response of EIF2AK1 (HRI) to heme deficiency
WP4239: Epithelial to mesenchymal transition in colorectal cancer
GO:0042273: ribosomal large subunit biogenesis
GO:0006914: autophagy
R-HSA-449147: Signaling by Interleukins
GO:0051090: regulation of DNA-binding transcription factor activity
M65: PID FRA PATHWAY
GO:0006790: sulfur compound metabolic process
GO:0071496: cellular response to external stimulus
GO:0010735: positive regulation of transcription via serum response element binding
GO:1900745: positive regulation of p38MAPK cascade
GO:0051438: regulation of ubiquitin-protein transferase activity
GO:0040008: regulation of growth
M5887: NABA BASEMENT MEMBRANES
GO:0010225: response to UV-C
CORUM:2920: Respiratory chain complex I (lambda subunit) mitochondrial
R-HSA-5628897: TP53 Regulates Metabolic Genes
GO:0070997: neuron death
GO:0062197: cellular response to chemical stress
GO:0009649: entrainment of circadian clock
R-HSA-5668914: Diseases of metabolism
GO:0044273: sulfur compound catabolic process
CORUM:5232: TNF-alpha/Nf-kappa B signaling complex (RPL6, RPL30, RPS13, CHUK, DDX3X, NFKB2, NFKBIB, REL, IKBKG, NFKB1, MAP3K8, RELB, GLG1, NFKBIA, RELA, TNIP2, GTF2I)
GO:0002521: leukocyte differentiation
ko04122: Sulfur relay system
GO:0043433: negative regulation of DNA-binding transcription factor activity
GO:0071356: cellular response to tumor necrosis factor
GO:0006520: cellular amino acid metabolic process
GO:0097191: extrinsic apoptotic signaling pathway
GO:0061028: establishment of endothelial barrier
GO:0060546: negative regulation of necroptotic process
GO:0032496: response to lipopolysaccharide
GO:1901796: regulation of signal transduction by p53 class mediator
GO:0022604: regulation of cell morphogenesis
WP107: Translation factors
WP4754: IL-18 signaling pathway
M256: PID TAP63 PATHWAY
WP2858: Ectoderm differentiation
GO:0043065: positive regulation of apoptotic process
GO:1901798: positive regulation of signal transduction by p53 class mediator
GO:1902600: proton transmembrane transport
GO:0044270: cellular nitrogen compound catabolic process
GO:0016032: viral process
GO:1903935: response to sodium arsenite
WP15: Selenium micronutrient network
CORUM:6848: IRF2BP2-IRF2BP1-IRF2BPL complex
GO:2000379: positive regulation of reactive oxygen species metabolic process
GO:0031400: negative regulation of protein modification process
GO:0048729: tissue morphogenesis
GO:0051098: regulation of binding
hsa05120: Epithelial cell signaling in Helicobacter pylori infection
GO:0000904: cell morphogenesis involved in differentiation
hsa05132: Salmonella infection
GO:0030224: monocyte differentiation
WP4922: Mitochondrial complex IV assembly
GO:0034330: cell junction organization
CORUM:2905: Respiratory chain complex I (intermediate), mitochondrial
WP4747: Netrin-UNC5B signaling pathway
R-HSA-5389840: Mitochondrial translation elongation
GO:0019915: lipid storage
GO:0032480: negative regulation of type I interferon production
GO:0042053: regulation of dopamine metabolic process
GO:0045110: intermediate filament bundle assembly
hsa05418: Fluid shear stress and atherosclerosis
GO:0060969: negative regulation of gene silencing
GO:0070059: intrinsic apoptotic signaling pathway in response to endoplasmic reticulum stress
WP4963: p53 transcriptional gene network
R-HSA-446728: Cell junction organization
R-HSA-5696398: Nucleotide Excision Repair
GO:0001666: response to hypoxia
GO:0003180: aortic valve morphogenesis
GO:0051668: localization within membrane
GO:0051881: regulation of mitochondrial membrane potential
GO:0051205: protein insertion into membrane
R-HSA-5140745: WNT5A-dependent internalization of FZD2, FZD5 and ROR2
M8873: ST FAS SIGNALING PATHWAY
GO:0000028: ribosomal small subunit assembly
0
10
20
30
40
-log10(P)
