# Supplementary material for: Transcriptional profiling of Hutchinson-Gilford Progeria syndrome fibroblasts reveals deficits in mesenchymal stem cell commitment to differentiation related to early events in endochondral ossification
Source: eLife. 2022 Dec 29;11:e81290. doi: 10.7554/eLife.81290 (PMC9833827; doi:10.7554/eLife.81290)
Supplement: Table 1—source data 1. — Childhood and teenaged patient samples compared to middle aged and older adults. [file elife-81290-table1-data1.zip › Table_1_Source_Data_1/Teen-O_UP_600.zip!/Teen-O_UP_600/Enrichment_QC/HeatmapSelectedGO_COVID.pdf]

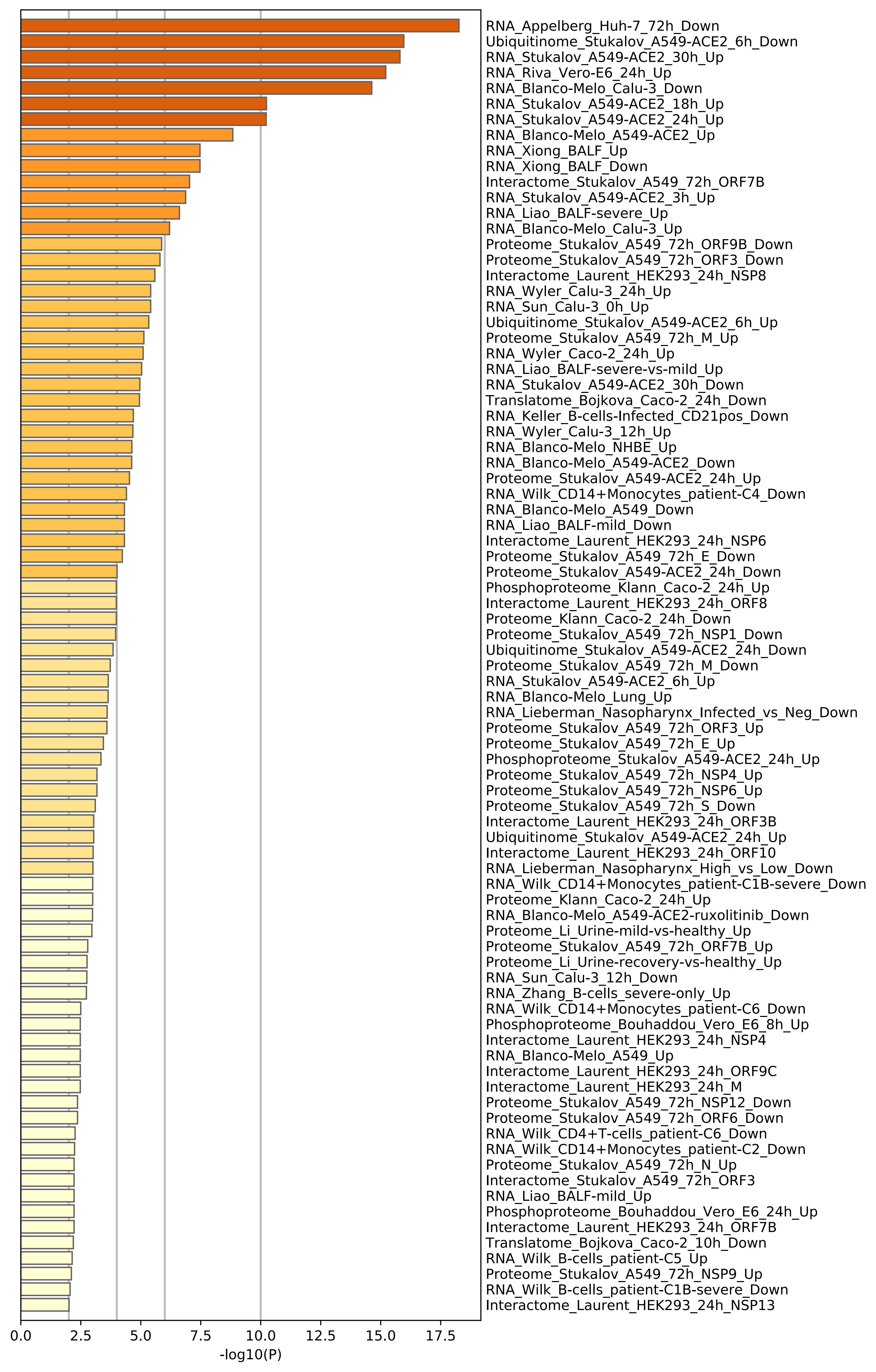
RNA_Appelberg_Huh-7_72h_Down
Ubiquitinome_Stukalov_A549-ACE2_6h_Down
RNA_Stukalov_A549-ACE2_30h_Up
RNA_Riva_Vero-E6_24h_Up
RNA_Blanco-Melo_Calu-3_Down
RNA_Stukalov_A549-ACE2_18h_Up
RNA_Stukalov_A549-ACE2_24h_Up
RNA_Blanco-Melo_A549-ACE2_Up
RNA_Xiong_BALF_Up
RNA_Xiong_BALF_Down
Interactome_Stukalov_A549_72h_ORF7B
RNA_Stukalov_A549-ACE2_3h_Up
RNA_Liao_BALF-severe_Up
RNA_Blanco-Melo_Calu-3_Up
Proteome_Stukalov_A549_72h_ORF9B_Down
Proteome_Stukalov_A549_72h_ORF3_Down
Interactome_Laurent_HEK293_24h_NSP8
RNA_Wyler_Calu-3_24h_Up
RNA_Sun_Calu-3_0h_Up
Ubiquitinome_Stukalov_A549-ACE2_6h_Up
Proteome_Stukalov_A549_72h_M_Up
RNA_Wyler_Caco-2_24h_Up
RNA_Liao_BALF-severe-vs-mild_Up
RNA_Stukalov_A549-ACE2_30h_Down
Translatome_Bojkova_Caco-2_24h_Down
RNA_Keller_B-cells-Infected_CD21pos_Down
RNA_Wyler_Calu-3_12h_Up
RNA_Blanco-Melo_NHBE_Up
RNA_Blanco-Melo_A549-ACE2_Down
Proteome_Stukalov_A549-ACE2_24h_Up
RNA_Wilk_CD14+Monocytes_patient-C4_Down
RNA_Blanco-Melo_A549_Down
RNA_Liao_BALF-mild_Down
Interactome_Laurent_HEK293_24h_NSP6
Proteome_Stukalov_A549_72h_E_Down
Proteome_Stukalov_A549-ACE2_24h_Down
Phosphoproteome_Klann_Caco-2_24h_Up
Interactome_Laurent_HEK293_24h_ORF8
Proteome_Klann_Caco-2_24h_Down
Proteome_Stukalov_A549_72h_NSP1_Down
Ubiquitinome_Stukalov_A549-ACE2_24h_Down
Proteome_Stukalov_A549_72h_M_Down
RNA_Stukalov_A549-ACE2_6h_Up
RNA_Blanco-Melo_Lung_Up
RNA_Lieberman_Nasopharynx_Infected_vs_Neg_Down
Proteome_Stukalov_A549_72h_ORF3_Up
Proteome_Stukalov_A549_72h_E_Up
Phosphoproteome_Stukalov_A549-ACE2_24h_Up
Proteome_Stukalov_A549_72h_NSP4_Up
Proteome_Stukalov_A549_72h_NSP6_Up
Proteome_Stukalov_A549_72h_S_Down
Interactome_Laurent_HEK293_24h_ORF3B
Ubiquitinome_Stukalov_A549-ACE2_24h_Up
Interactome_Laurent_HEK293_24h_ORF10
RNA_Lieberman_Nasopharynx_High_vs_Low_Down
RNA_Wilk_CD14+Monocytes_patient-C1B-severe_Down
Proteome_Klann_Caco-2_24h_Up
RNA_Blanco-Melo_A549-ACE2-ruxolitinib_Down
Proteome_Li_Urine-mild-vs-healthy_Up
Proteome_Stukalov_A549_72h_ORF7B_Up
Proteome_Li_Urine-recovery-vs-healthy_Up
RNA_Sun_Calu-3_12h_Down
RNA_Zhang_B-cells_severe-only_Up
RNA_Wilk_CD14+Monocytes_patient-C6_Down
Phosphoproteome_Bouhaddou_Vero_E6_8h_Up
Interactome_Laurent_HEK293_24h_NSP4
RNA_Blanco-Melo_A549_Up
Interactome_Laurent_HEK293_24h_ORF9C
Interactome_Laurent_HEK293_24h_M
Proteome_Stukalov_A549_72h_NSP12_Down
Proteome_Stukalov_A549_72h_ORF6_Down
RNA_Wilk_CD4+T-cells_patient-C6_Down
RNA_Wilk_CD14+Monocytes_patient-C2_Down
Proteome_Stukalov_A549_72h_N_Up
Interactome_Stukalov_A549_72h_ORF3
RNA_Liao_BALF-mild_Up
Phosphoproteome_Bouhaddou_Vero_E6_24h_Up
Interactome_Laurent_HEK293_24h_ORF7B
Translatome_Bojkova_Caco-2_10h_Down
RNA_Wilk_B-cells_patient-C5_Up
Proteome_Stukalov_A549_72h_NSP9_Up
RNA_Wilk_B-cells_patient-C1B-severe_Down
Interactome_Laurent_HEK293_24h_NSP13
0.0
2.5
5.0
7.5
10.0
12.5
15.0
17.5
-log10(P)
