# Supplementary material for: Transcriptional profiling of Hutchinson-Gilford Progeria syndrome fibroblasts reveals deficits in mesenchymal stem cell commitment to differentiation related to early events in endochondral ossification
Source: eLife. 2022 Dec 29;11:e81290. doi: 10.7554/eLife.81290 (PMC9833827; doi:10.7554/eLife.81290)
Supplement: Table 1—source data 1. — Childhood and teenaged patient samples compared to middle aged and older adults. [file elife-81290-table1-data1.zip › Table_1_Source_Data_1/Teen-O_UP_600.zip!/Teen-O_UP_600/Enrichment_QC/HeatmapSelectedGO_DisGeNET.pdf]

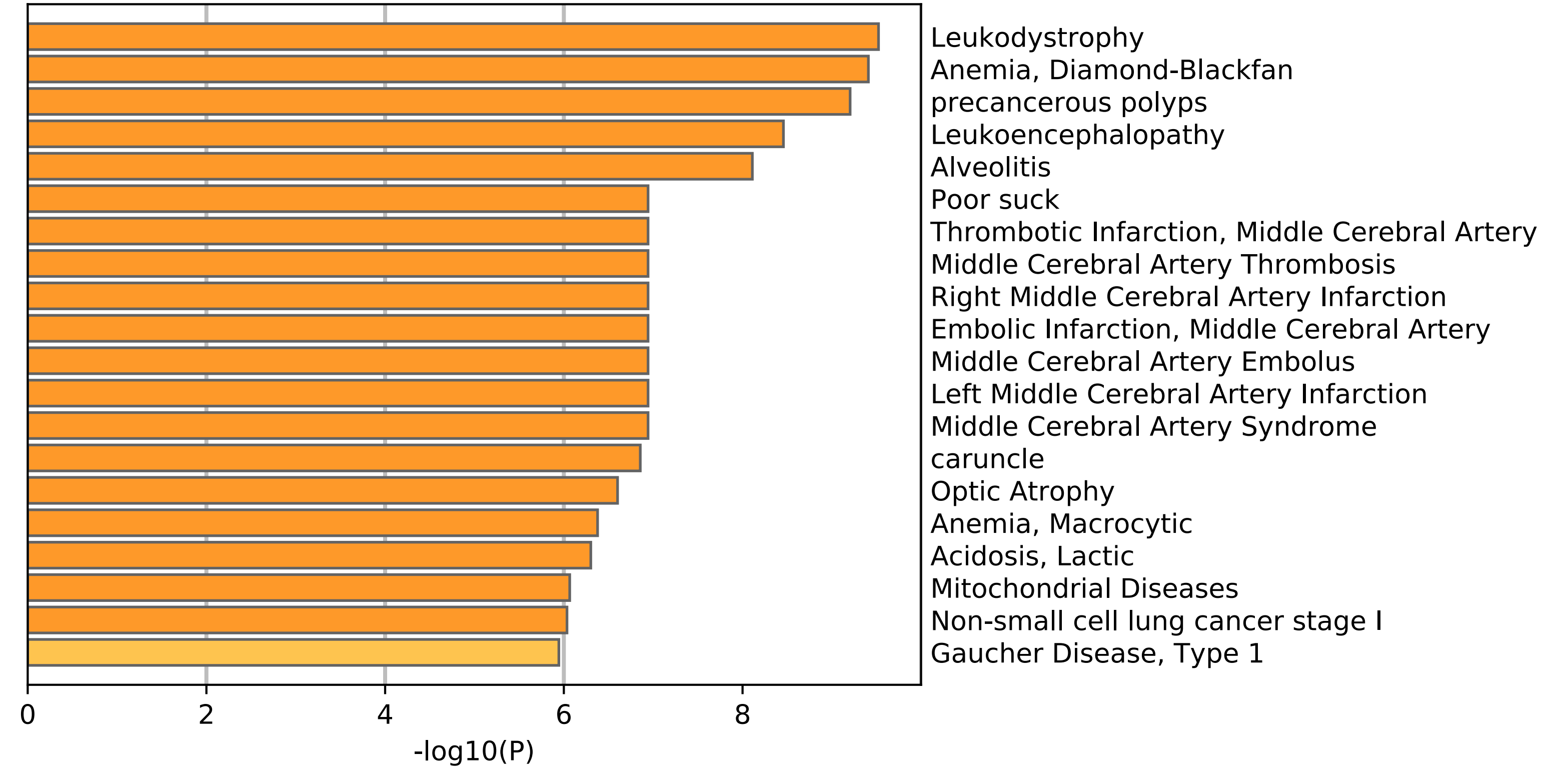
Leukodystrophy
Anemia, Diamond-Blackfan
precancerous polyps
Leukoencephalopathy
Alveolitis
Poor suck
Thrombotic Infarction, Middle Cerebral Artery
Middle Cerebral Artery Thrombosis
Right Middle Cerebral Artery Infarction
Embolic Infarction, Middle Cerebral Artery
Middle Cerebral Artery Embolus
Left Middle Cerebral Artery Infarction
Middle Cerebral Artery Syndrome
caruncle
Optic Atrophy
Anemia, Macrocytic
Acidosis, Lactic
Mitochondrial Diseases
Non-small cell lung cancer stage I
Gaucher Disease, Type 1
0
2
4
6
8
-log10(P)
