# Supplementary material for: Transcriptional profiling of Hutchinson-Gilford Progeria syndrome fibroblasts reveals deficits in mesenchymal stem cell commitment to differentiation related to early events in endochondral ossification
Source: eLife. 2022 Dec 29;11:e81290. doi: 10.7554/eLife.81290 (PMC9833827; doi:10.7554/eLife.81290)
Supplement: Table 1—source data 1. — Childhood and teenaged patient samples compared to middle aged and older adults. [file elife-81290-table1-data1.zip › Table_1_Source_Data_1/Teen-O_UP_600.zip!/Teen-O_UP_600/Enrichment_QC/HeatmapSelectedGO_Transcription_Factor_Targets.pdf]

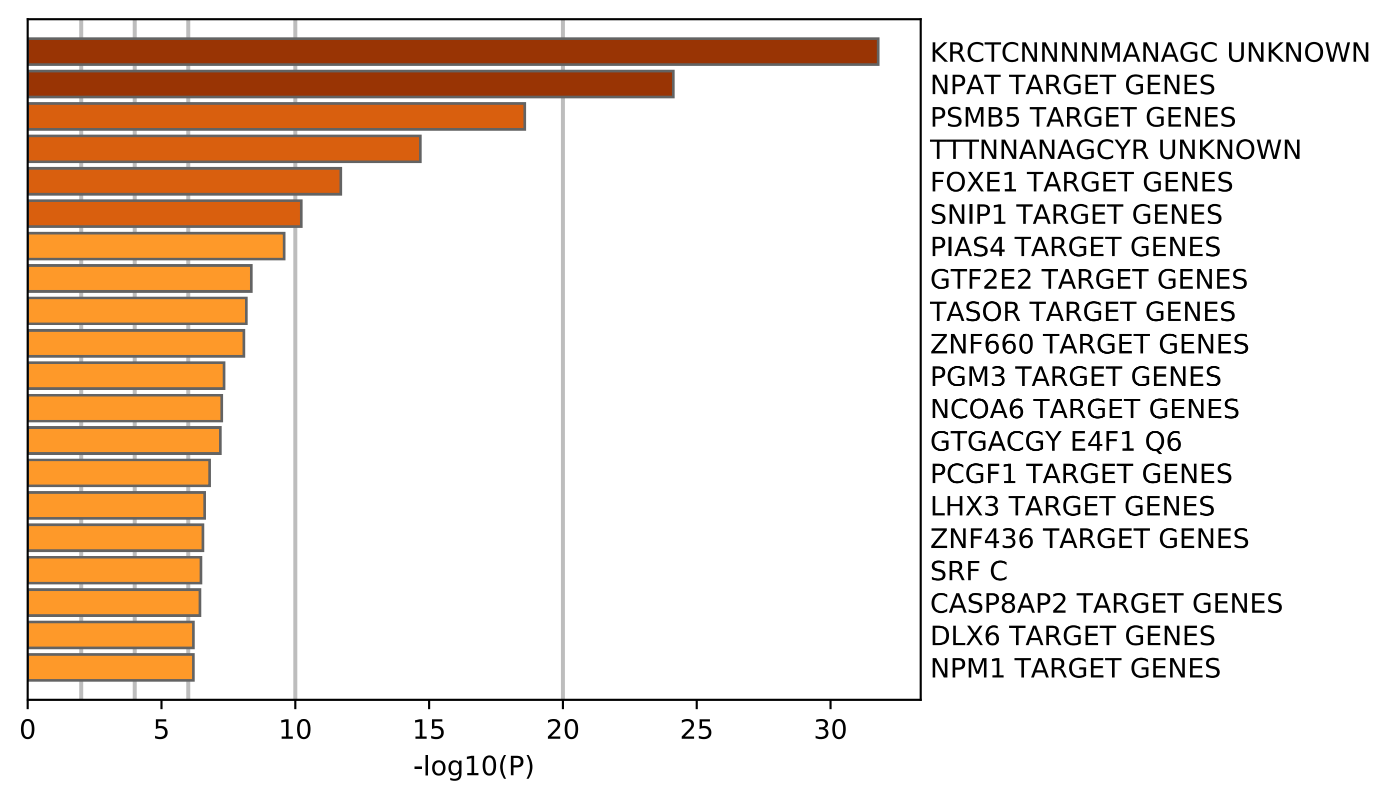
KRCTCNNNNMANAGC UNKNOWN
NPAT TARGET GENES
PSMB5 TARGET GENES
TTTNNANAGCYR UNKNOWN
FOXE1 TARGET GENES
SNIP1 TARGET GENES
PIAS4 TARGET GENES
GTF2E2 TARGET GENES
TASOR TARGET GENES
ZNF660 TARGET GENES
PGM3 TARGET GENES
NCOA6 TARGET GENES
GTGACGY E4F1 Q6
PCGF1 TARGET GENES
LHX3 TARGET GENES
ZNF436 TARGET GENES
SRF C
CASP8AP2 TARGET GENES
DLX6 TARGET GENES
NPM1 TARGET GENES
0
5
10
15
20
25
30
-log10(P)
