# Supplementary material for: Transcriptional profiling of Hutchinson-Gilford Progeria syndrome fibroblasts reveals deficits in mesenchymal stem cell commitment to differentiation related to early events in endochondral ossification
Source: eLife. 2022 Dec 29;11:e81290. doi: 10.7554/eLife.81290 (PMC9833827; doi:10.7554/eLife.81290)
Supplement: Table 1—source data 1. — Childhood and teenaged patient samples compared to middle aged and older adults. [file elife-81290-table1-data1.zip › Table_1_Source_Data_1/Teen-O_UP_600.zip!/Teen-O_UP_600/Enrichment_QC/HeatmapSelectedGO_TRRUST.pdf]

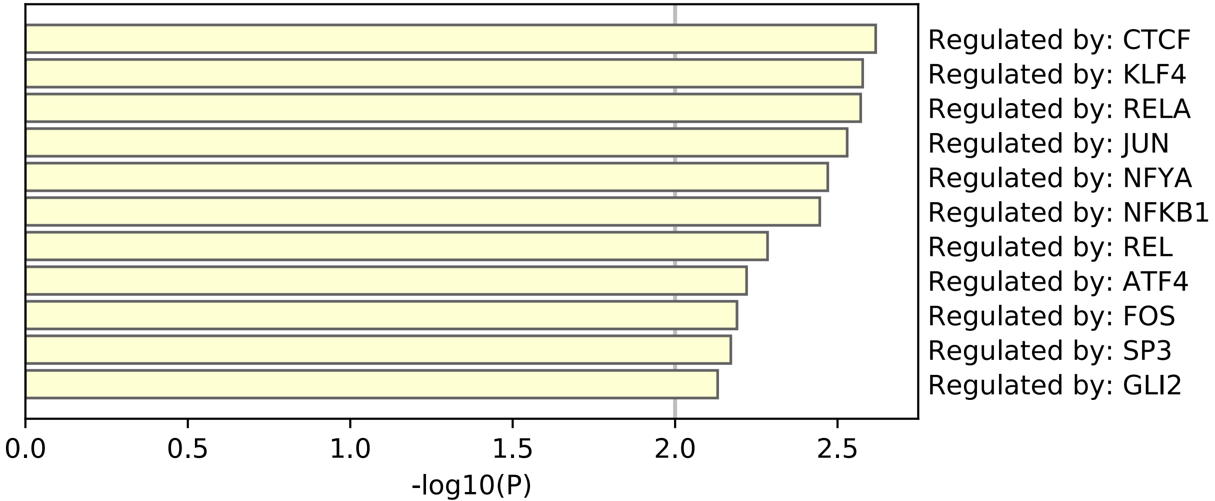
Regulated by: CTCF
Regulated by: KLF4
Regulated by: RELA
Regulated by: JUN
Regulated by: NFYA
Regulated by: NFKB1
Regulated by: REL
Regulated by: ATF4
Regulated by: FOS
Regulated by: SP3
Regulated by: GLI2
0.0
0.5
1.0
1.5
2.0
2.5
-log10(P)
