# Supplementary material for: Transcriptional profiling of Hutchinson-Gilford Progeria syndrome fibroblasts reveals deficits in mesenchymal stem cell commitment to differentiation related to early events in endochondral ossification
Source: eLife. 2022 Dec 29;11:e81290. doi: 10.7554/eLife.81290 (PMC9833827; doi:10.7554/eLife.81290)
Supplement: Table 2—source data 1. — Early and late childhood patient samples compared to healthy children, middle aged, and older adults. [file elife-81290-table2-data1.zip › Table_2-Source_Data_1/0-3-AM-DOWN-25.zip!/Enrichment_heatmap/HeatmapSelectedGO.pdf]

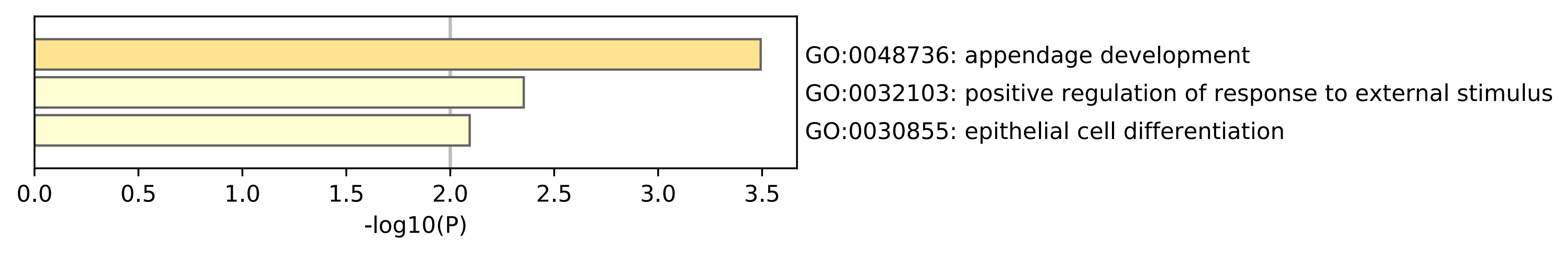
GO:0048736: appendage development
GO:0032103: positive regulation of response to external stimulus
GO:0030855: epithelial cell differentiation
0.0
0.5
1.0
1.5
2.0
2.5
3.0
3.5
-log10(P)
