# Supplementary material for: Transcriptional profiling of Hutchinson-Gilford Progeria syndrome fibroblasts reveals deficits in mesenchymal stem cell commitment to differentiation related to early events in endochondral ossification
Source: eLife. 2022 Dec 29;11:e81290. doi: 10.7554/eLife.81290 (PMC9833827; doi:10.7554/eLife.81290)
Supplement: Table 2—source data 1. — Early and late childhood patient samples compared to healthy children, middle aged, and older adults. [file elife-81290-table2-data1.zip › Table_2-Source_Data_1/0-3-AM-UP-113.zip!/Enrichment_GO/ColorByCluster.pdf]

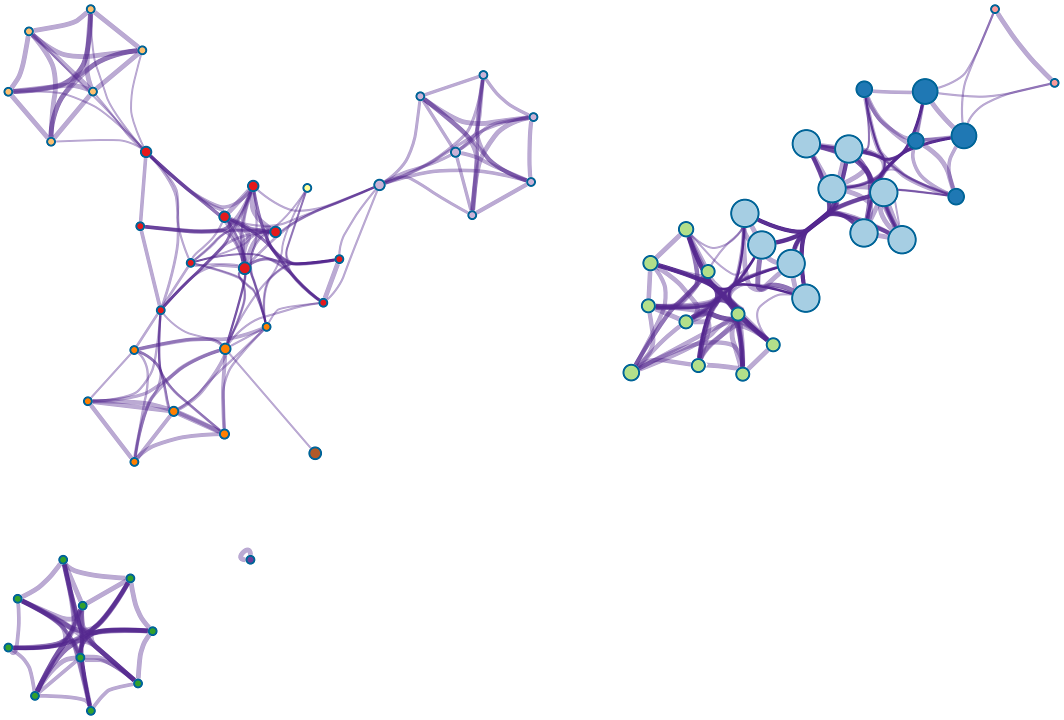

HDACs deacetylate histones
Ub-specific processing proteases
Interleukin-7 signaling
Apoptosis induced DNA fragmentation
E3 ubiquitin ligases ubiquitinate target proteins
positive regulation of protein kinase activity
positive regulation of cysteine-type endopeptidase ac
regulation of chemotaxis
BMP signaling pathway
Cardiac muscle contraction
spinal cord development
negative regulation of cell population proliferation

created by
http://metascape.org
