# Supplementary material for: Transcriptional profiling of Hutchinson-Gilford Progeria syndrome fibroblasts reveals deficits in mesenchymal stem cell commitment to differentiation related to early events in endochondral ossification
Source: eLife. 2022 Dec 29;11:e81290. doi: 10.7554/eLife.81290 (PMC9833827; doi:10.7554/eLife.81290)
Supplement: Table 2—source data 1. — Early and late childhood patient samples compared to healthy children, middle aged, and older adults. [file elife-81290-table2-data1.zip › Table_2-Source_Data_1/0-3-AM-UP-113.zip!/Enrichment_heatmap/HeatmapSelectedGO.pdf]

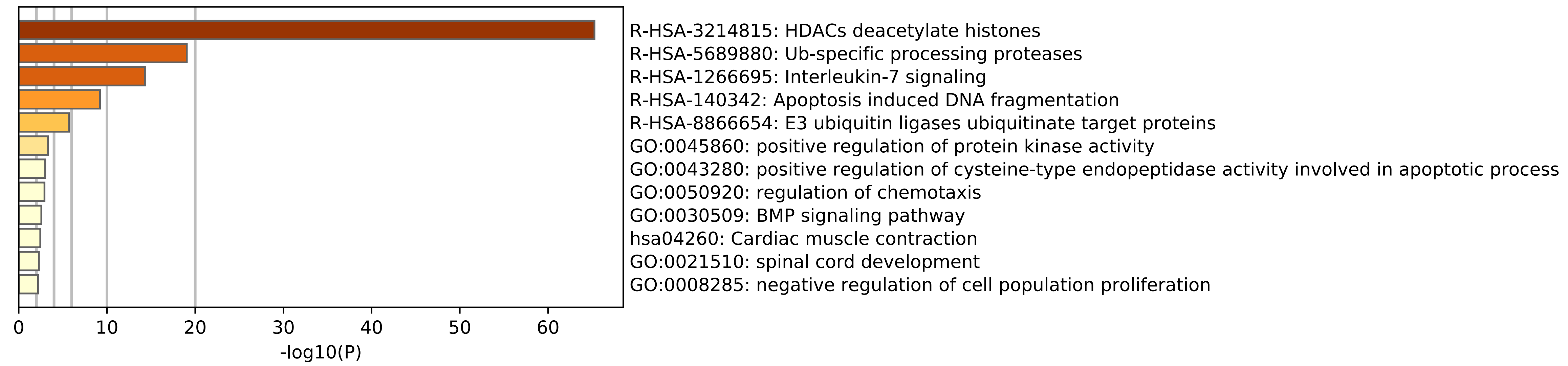
R-HSA-3214815: HDACs deacetylate histones
R-HSA-5689880: Ub-specific processing proteases
R-HSA-1266695: Interleukin-7 signaling
R-HSA-140342: Apoptosis induced DNA fragmentation
R-HSA-8866654: E3 ubiquitin ligases ubiquitinate target proteins
GO:0045860: positive regulation of protein kinase activity
GO:0043280: positive regulation of cysteine-type endopeptidase activity involved in apoptotic process
GO:0050920: regulation of chemotaxis
GO:0030509: BMP signaling pathway
hsa04260: Cardiac muscle contraction
GO:0021510: spinal cord development
GO:0008285: negative regulation of cell population proliferation
0
10
20
30
40
50
60
-log10(P)
