# Supplementary material for: Transcriptional profiling of Hutchinson-Gilford Progeria syndrome fibroblasts reveals deficits in mesenchymal stem cell commitment to differentiation related to early events in endochondral ossification
Source: eLife. 2022 Dec 29;11:e81290. doi: 10.7554/eLife.81290 (PMC9833827; doi:10.7554/eLife.81290)
Supplement: Table 2—source data 1. — Early and late childhood patient samples compared to healthy children, middle aged, and older adults. [file elife-81290-table2-data1.zip › Table_2-Source_Data_1/0-3-M-DOWN-172.zip!/Enrichment_GO/ColorByCluster.pdf]

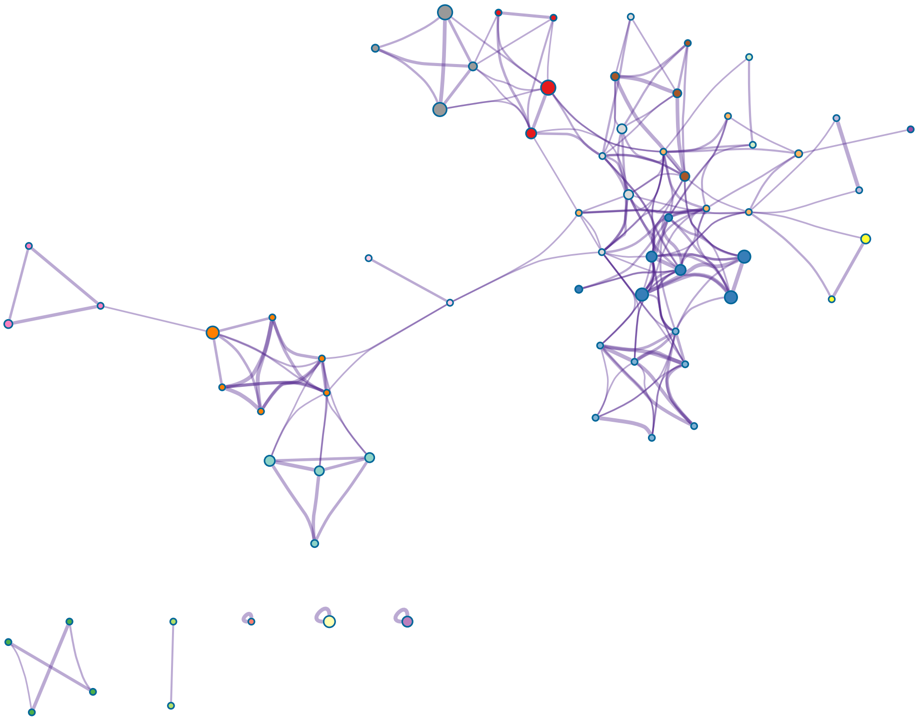

- Cytokine Signaling in Immune system
- positive regulation of apoptotic process
- response to muscle activity
- Inflammatory mediator regulation of TRP channels
- negative regulation of intracellular signal transductior
- Signaling by Nuclear Receptors
- response to oxygen levels
- negative regulation of immune effector process
- response to cytokine
- regionalization
- cell-cell adhesion
- dsRNA processing
- Prion disease pathway
- endodermal cell differentiation
- Melatonin metabolism and effects
- protein targeting to membrane
- Neural crest differentiation
- PID ANGIOPOIETIN RECEPTOR PATHWAY
- supramolecular fiber organization
- Neurotransmitter release cycle

created by
http://metascape.org
