# Supplementary material for: Transcriptional profiling of Hutchinson-Gilford Progeria syndrome fibroblasts reveals deficits in mesenchymal stem cell commitment to differentiation related to early events in endochondral ossification
Source: eLife. 2022 Dec 29;11:e81290. doi: 10.7554/eLife.81290 (PMC9833827; doi:10.7554/eLife.81290)
Supplement: Table 2—source data 1. — Early and late childhood patient samples compared to healthy children, middle aged, and older adults. [file elife-81290-table2-data1.zip › Table_2-Source_Data_1/0-3-M-DOWN-172.zip!/Enrichment_heatmap/HeatmapSelectedGOTop100.pdf]

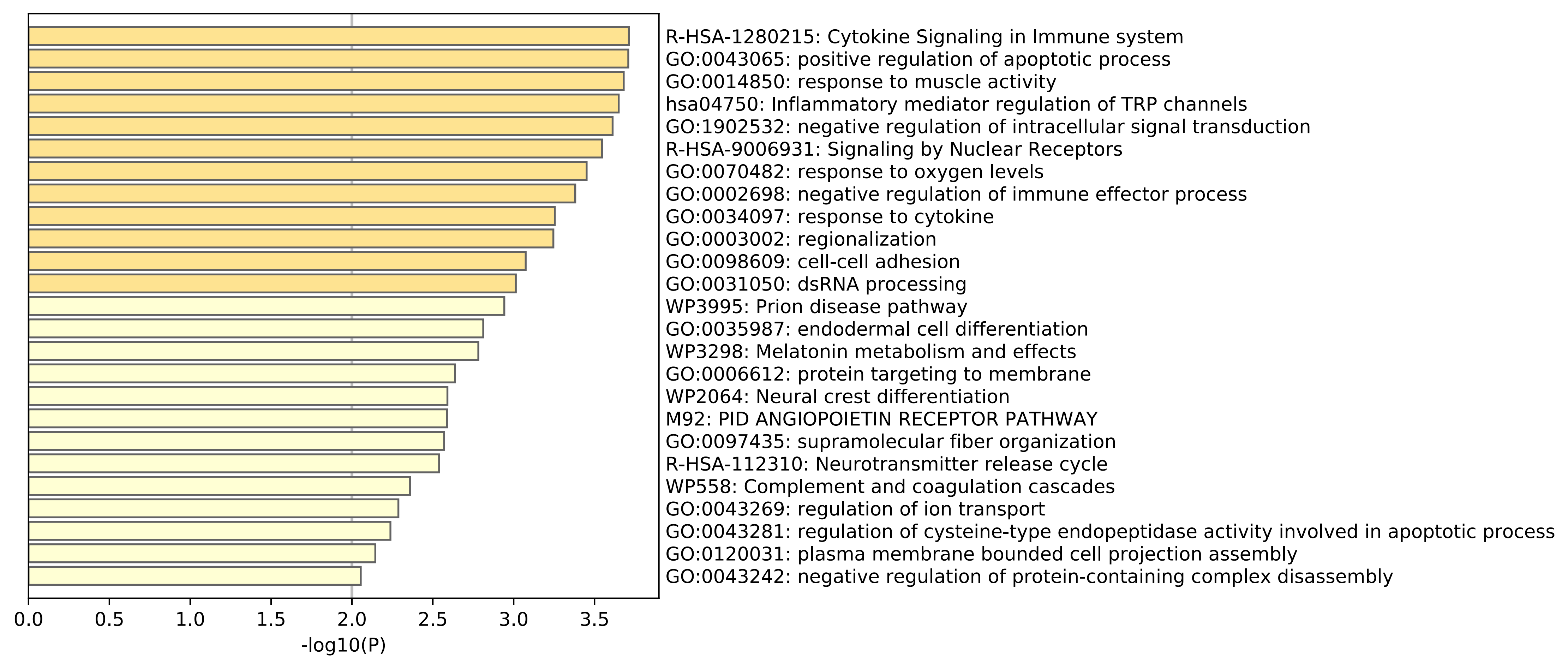
R-HSA-1280215: Cytokine Signaling in Immune system
GO:0043065: positive regulation of apoptotic process
GO:0014850: response to muscle activity
hsa04750: Inflammatory mediator regulation of TRP channels
GO:1902532: negative regulation of intracellular signal transduction
R-HSA-9006931: Signaling by Nuclear Receptors
GO:0070482: response to oxygen levels
GO:0002698: negative regulation of immune effector process
GO:0034097: response to cytokine
GO:0003002: regionalization
GO:0098609: cell-cell adhesion
GO:0031050: dsRNA processing
WP3995: Prion disease pathway
GO:0035987: endodermal cell differentiation
WP3298: Melatonin metabolism and effects
GO:0006612: protein targeting to membrane
WP2064: Neural crest differentiation
M92: PID ANGIOPOIETIN RECEPTOR PATHWAY
GO:0097435: supramolecular fiber organization
R-HSA-112310: Neurotransmitter release cycle
WP558: Complement and coagulation cascades
GO:0043269: regulation of ion transport
GO:0043281: regulation of cysteine-type endopeptidase activity involved in apoptotic process
GO:0120031: plasma membrane bounded cell projection assembly
GO:0043242: negative regulation of protein-containing complex disassembly
0.0
0.5
1.0
1.5
2.0
2.5
3.0
3.5
-log10(P)
