# Supplementary material for: Transcriptional profiling of Hutchinson-Gilford Progeria syndrome fibroblasts reveals deficits in mesenchymal stem cell commitment to differentiation related to early events in endochondral ossification
Source: eLife. 2022 Dec 29;11:e81290. doi: 10.7554/eLife.81290 (PMC9833827; doi:10.7554/eLife.81290)
Supplement: Table 2—source data 1. — Early and late childhood patient samples compared to healthy children, middle aged, and older adults. [file elife-81290-table2-data1.zip › Table_2-Source_Data_1/0-3-M-UP-540.zip!/Enrichment_GO/ColorByCluster.pdf]

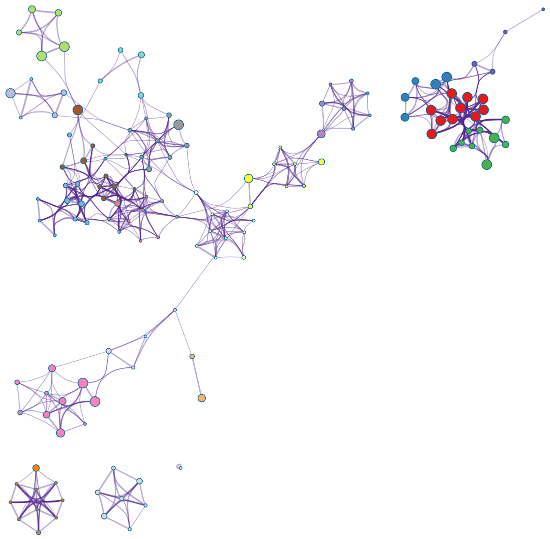

HDACs deacetylate histones
Ub-specific processing proteases
Interleukin-7 signaling
E3 ubiquitin ligases ubiquitinate target proteins
Apoptosis induced DNA fragmentation
TP53 Regulates Metabolic Genes
cardiac septum morphogenesis
peptide metabolic process
Pathways in cancer
Hippo signaling pathway
Ca2+ pathway
Neuronal System
mesenchyme morphogenesis
appendage development
regulation of neuron death
telencephalon development
cell redox homeostasis
regulation of synapse assembly
protein localization to mitochondrion
Amyotrophic lateral sclerosis (ALS)

created by
http://metascape.org
