# Supplementary material for: Transcriptional profiling of Hutchinson-Gilford Progeria syndrome fibroblasts reveals deficits in mesenchymal stem cell commitment to differentiation related to early events in endochondral ossification
Source: eLife. 2022 Dec 29;11:e81290. doi: 10.7554/eLife.81290 (PMC9833827; doi:10.7554/eLife.81290)
Supplement: Table 2—source data 1. — Early and late childhood patient samples compared to healthy children, middle aged, and older adults. [file elife-81290-table2-data1.zip › Table_2-Source_Data_1/0-3-M-UP-540.zip!/Enrichment_heatmap/HeatmapSelectedGO.pdf]

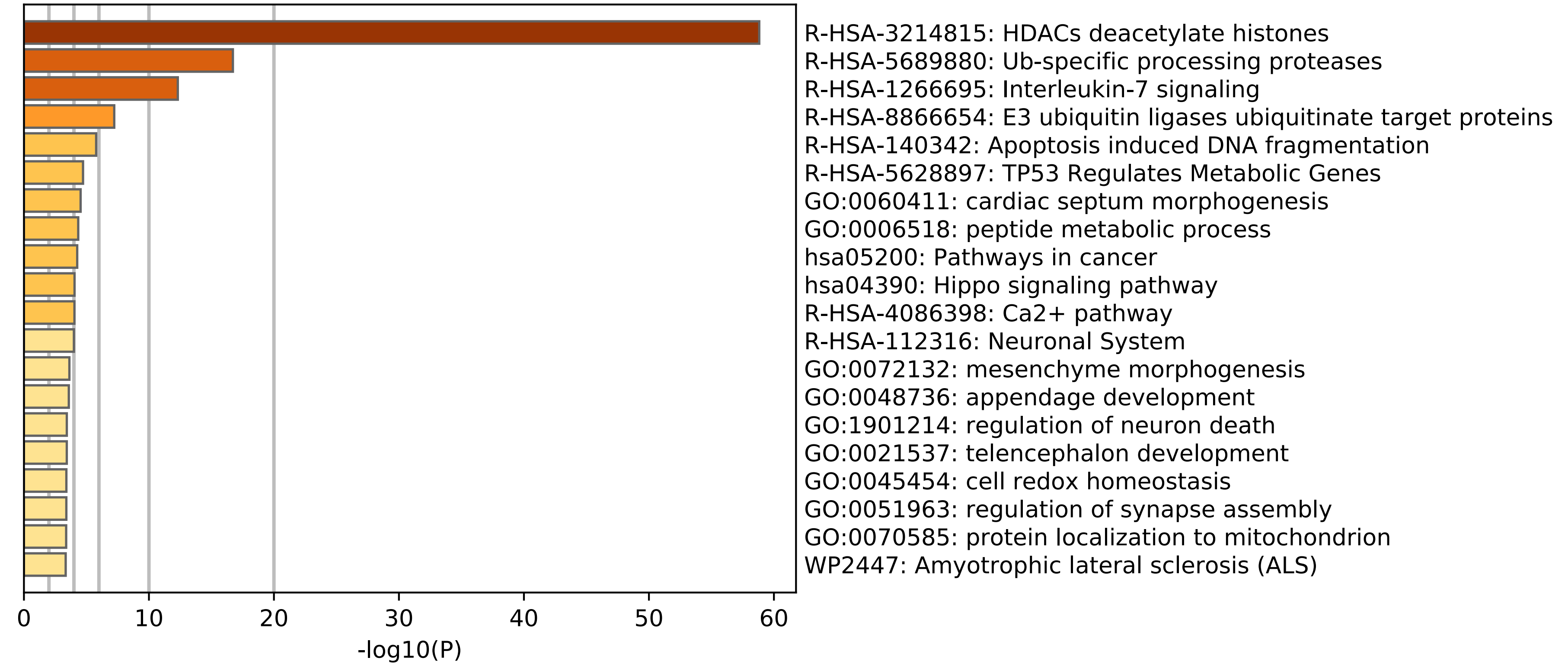
R-HSA-3214815: HDACs deacetylate histones
R-HSA-5689880: Ub-specific processing proteases
R-HSA-1266695: Interleukin-7 signaling
R-HSA-8866654: E3 ubiquitin ligases ubiquitinate target proteins
R-HSA-140342: Apoptosis induced DNA fragmentation
R-HSA-5628897: TP53 Regulates Metabolic Genes
GO:0060411: cardiac septum morphogenesis
GO:0006518: peptide metabolic process
hsa05200: Pathways in cancer
hsa04390: Hippo signaling pathway
R-HSA-4086398: Ca2+ pathway
R-HSA-112316: Neuronal System
GO:0072132: mesenchyme morphogenesis
GO:0048736: appendage development
GO:1901214: regulation of neuron death
GO:0021537: telencephalon development
GO:0045454: cell redox homeostasis
GO:0051963: regulation of synapse assembly
GO:0070585: protein localization to mitochondrion
WP2447: Amyotrophic lateral sclerosis (ALS)
0
10
20
30
40
50
60
-log10(P)
