# Supplementary material for: Transcriptional profiling of Hutchinson-Gilford Progeria syndrome fibroblasts reveals deficits in mesenchymal stem cell commitment to differentiation related to early events in endochondral ossification
Source: eLife. 2022 Dec 29;11:e81290. doi: 10.7554/eLife.81290 (PMC9833827; doi:10.7554/eLife.81290)
Supplement: Table 2—source data 1. — Early and late childhood patient samples compared to healthy children, middle aged, and older adults. [file elife-81290-table2-data1.zip › Table_2-Source_Data_1/0-3-M-UP-540.zip!/Enrichment_heatmap/HeatmapSelectedGOTop100.pdf]

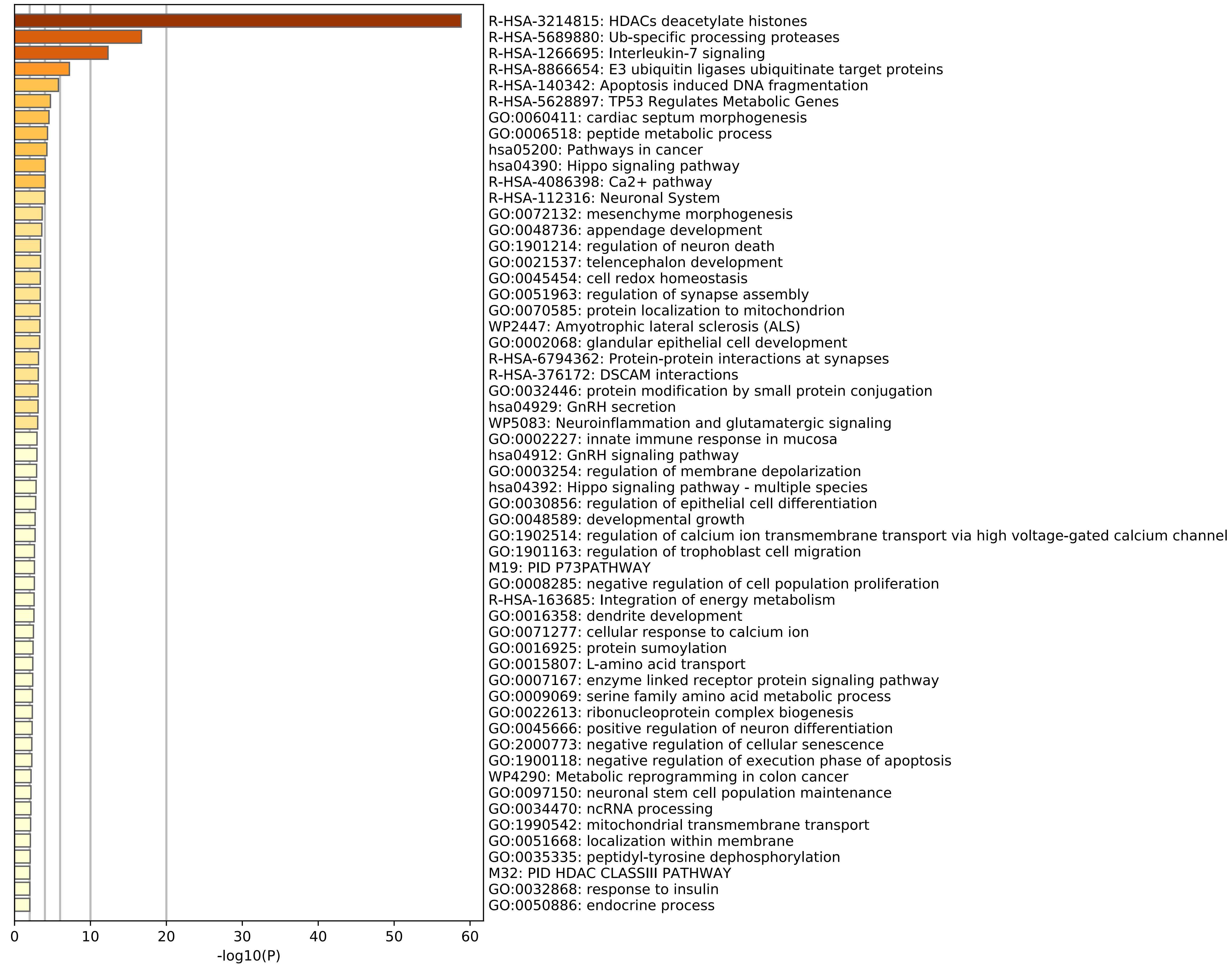
R-HSA-3214815: HDACs deacetylate histones
R-HSA-5689880: Ub-specific processing proteases
R-HSA-1266695: Interleukin-7 signaling
R-HSA-8866654: E3 ubiquitin ligases ubiquitinate target proteins
R-HSA-140342: Apoptosis induced DNA fragmentation
R-HSA-5628897: TP53 Regulates Metabolic Genes
GO:0060411: cardiac septum morphogenesis
GO:0006518: peptide metabolic process
hsa05200: Pathways in cancer
hsa04390: Hippo signaling pathway
R-HSA-4086398: Ca2+ pathway
R-HSA-112316: Neuronal System
GO:0072132: mesenchyme morphogenesis
GO:0048736: appendage development
GO:1901214: regulation of neuron death
GO:0021537: telencephalon development
GO:0045454: cell redox homeostasis
GO:0051963: regulation of synapse assembly
GO:0070585: protein localization to mitochondrion
WP2447: Amyotrophic lateral sclerosis (ALS)
GO:0002068: glandular epithelial cell development
R-HSA-6794362: Protein-protein interactions at synapses
R-HSA-376172: DSCAM interactions
GO:0032446: protein modification by small protein conjugation
hsa04929: GnRH secretion
WP5083: Neuroinflammation and glutamatergic signaling
GO:0002227: innate immune response in mucosa
hsa04912: GnRH signaling pathway
GO:0003254: regulation of membrane depolarization
hsa04392: Hippo signaling pathway - multiple species
GO:0030856: regulation of epithelial cell differentiation
GO:0048589: developmental growth
GO:1902514: regulation of calcium ion transmembrane transport via high voltage-gated calcium channel
GO:1901163: regulation of trophoblast cell migration
M19: PID P73PATHWAY
GO:0008285: negative regulation of cell population proliferation
R-HSA-163685: Integration of energy metabolism
GO:0016358: dendrite development
GO:0071277: cellular response to calcium ion
GO:0016925: protein sumoylation
GO:0015807: L-amino acid transport
GO:0007167: enzyme linked receptor protein signaling pathway
GO:0009069: serine family amino acid metabolic process
GO:0022613: ribonucleoprotein complex biogenesis
GO:0045666: positive regulation of neuron differentiation
GO:2000773: negative regulation of cellular senescence
GO:1900118: negative regulation of execution phase of apoptosis
WP4290: Metabolic reprogramming in colon cancer
GO:0097150: neuronal stem cell population maintenance
GO:0034470: ncRNA processing
GO:1990542: mitochondrial transmembrane transport
GO:0051668: localization within membrane
GO:0035335: peptidyl-tyrosine dephosphorylation
M32: PID HDAC CLASSIII PATHWAY
GO:0032868: response to insulin
GO:0050886: endocrine process
0
10
20
30
40
50
60
-log10(P)
