# Supplementary material for: Transcriptional profiling of Hutchinson-Gilford Progeria syndrome fibroblasts reveals deficits in mesenchymal stem cell commitment to differentiation related to early events in endochondral ossification
Source: eLife. 2022 Dec 29;11:e81290. doi: 10.7554/eLife.81290 (PMC9833827; doi:10.7554/eLife.81290)
Supplement: Table 2—source data 1. — Early and late childhood patient samples compared to healthy children, middle aged, and older adults. [file elife-81290-table2-data1.zip › Table_2-Source_Data_1/0-3-O-DOWN_388.zip!/Enrichment_GO/ColorByCluster.pdf]

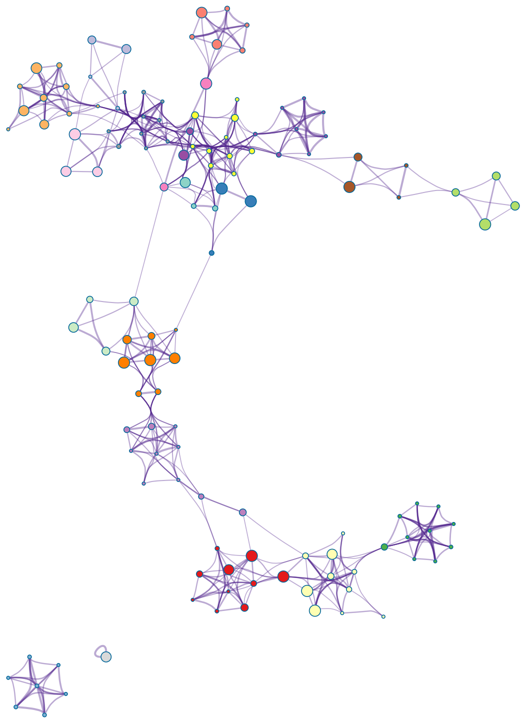

negative regulation of transport
Signaling by Interleukins
neurotransmitter metabolic process
EGF/EGFR signaling pathway
blood vessel maturation
NOD-like receptor signaling pathway
Platelet activation, signaling and aggregation
enzyme linked receptor protein signaling pathway
actin polymerization or depolymerization
VEGFA-VEGFR2 signaling pathway
regulation of vesicle-mediated transport
lamellipodium organization
response to growth factor
Sarcoglycan-sarcospan complex SG-SPN
regulation of leukocyte migration
regulation of proteolysis
actin filament-based process
Neutrophil degranulation
multi-multicellular organism process
pattern specification process

created by
http://metascape.org
