# Supplementary material for: Transcriptional profiling of Hutchinson-Gilford Progeria syndrome fibroblasts reveals deficits in mesenchymal stem cell commitment to differentiation related to early events in endochondral ossification
Source: eLife. 2022 Dec 29;11:e81290. doi: 10.7554/eLife.81290 (PMC9833827; doi:10.7554/eLife.81290)
Supplement: Table 2—source data 1. — Early and late childhood patient samples compared to healthy children, middle aged, and older adults. [file elife-81290-table2-data1.zip › Table_2-Source_Data_1/0-3-O-DOWN_388.zip!/Enrichment_heatmap/HeatmapSelectedGO.pdf]

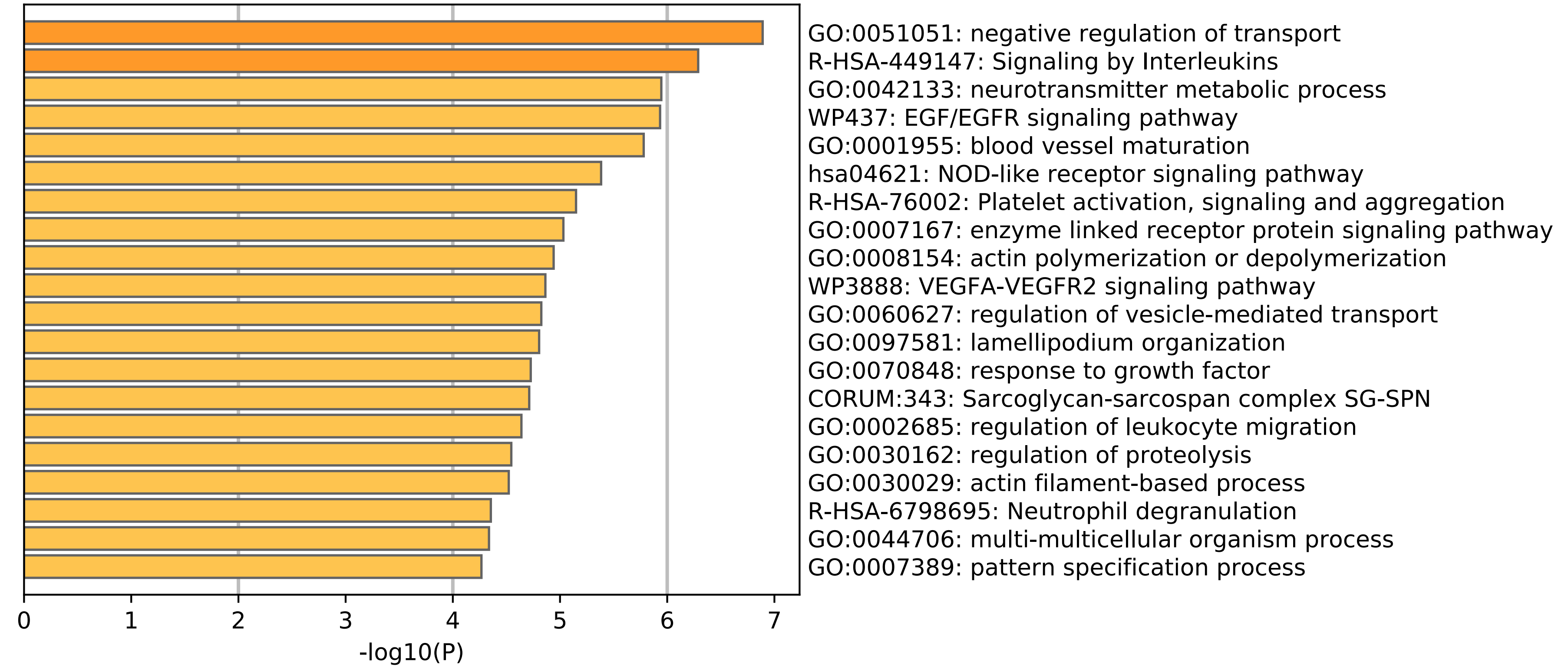
GO:0051051: negative regulation of transport
R-HSA-449147: Signaling by Interleukins
GO:0042133: neurotransmitter metabolic process
WP437: EGF/EGFR signaling pathway
GO:0001955: blood vessel maturation
hsa04621: NOD-like receptor signaling pathway
R-HSA-76002: Platelet activation, signaling and aggregation
GO:0007167: enzyme linked receptor protein signaling pathway
GO:0008154: actin polymerization or depolymerization
WP3888: VEGFA-VEGFR2 signaling pathway
GO:0060627: regulation of vesicle-mediated transport
GO:0097581: lamellipodium organization
GO:0070848: response to growth factor
CORUM:343: Sarcoglycan-sarcospan complex SG-SPN
GO:0002685: regulation of leukocyte migration
GO:0030162: regulation of proteolysis
GO:0030029: actin filament-based process
R-HSA-6798695: Neutrophil degranulation
GO:0044706: multi-multicellular organism process
GO:0007389: pattern specification process
0
1
2
3
4
5
6
7
-log10(P)
