# Supplementary material for: Transcriptional profiling of Hutchinson-Gilford Progeria syndrome fibroblasts reveals deficits in mesenchymal stem cell commitment to differentiation related to early events in endochondral ossification
Source: eLife. 2022 Dec 29;11:e81290. doi: 10.7554/eLife.81290 (PMC9833827; doi:10.7554/eLife.81290)
Supplement: Table 2—source data 1. — Early and late childhood patient samples compared to healthy children, middle aged, and older adults. [file elife-81290-table2-data1.zip › Table_2-Source_Data_1/0-3-O-DOWN_388.zip!/Enrichment_heatmap/HeatmapSelectedGOTop100.pdf]

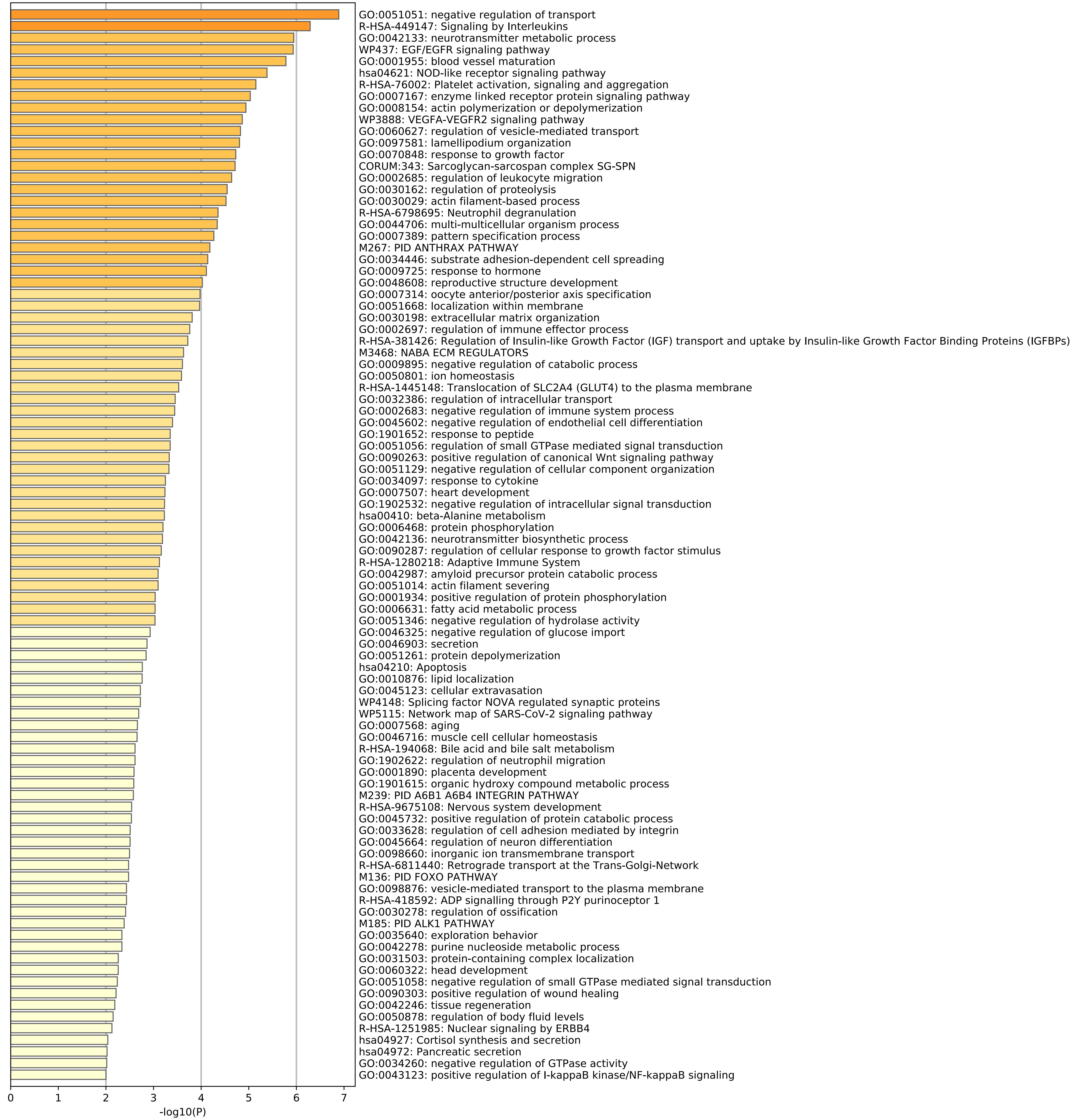
GO:0051051: negative regulation of transport
R-HSA-449147: Signaling by Interleukins
GO:0042133: neurotransmitter metabolic process
WP437: EGF/EGFR signaling pathway
GO:0001955: blood vessel maturation
hsa04621: NOD-like receptor signaling pathway
R-HSA-76002: Platelet activation, signaling and aggregation
GO:0007167: enzyme linked receptor protein signaling pathway
GO:0008154: actin polymerization or depolymerization
WP3888: VEGFA-VEGFR2 signaling pathway
GO:0060627: regulation of vesicle-mediated transport
GO:0097581: lamellipodium organization
GO:0070848: response to growth factor
CORUM:343: Sarcoglycan-sarcospan complex SG-SPN
GO:0002685: regulation of leukocyte migration
GO:0030162: regulation of proteolysis
GO:0030029: actin filament-based process
R-HSA-6798695: Neutrophil degranulation
GO:0044706: multi-multicellular organism process
GO:0007389: pattern specification process
M267: PID ANTHRAX PATHWAY
GO:0034446: substrate adhesion-dependent cell spreading
GO:0009725: response to hormone
GO:0048608: reproductive structure development
GO:0007314: oocyte anterior/posterior axis specification
GO:0051668: localization within membrane
GO:0030198: extracellular matrix organization
GO:0002697: regulation of immune effector process
R-HSA-381426: Regulation of Insulin-like Growth Factor (IGF) transport and uptake by Insulin-like Growth Factor Binding Proteins (IGFBPs)
M3468: NABA ECM REGULATORS
GO:0009895: negative regulation of catabolic process
GO:0050801: ion homeostasis
R-HSA-1445148: Translocation of SLC2A4 (GLUT4) to the plasma membrane
GO:0032386: regulation of intracellular transport
GO:0002683: negative regulation of immune system process
GO:0045602: negative regulation of endothelial cell differentiation
GO:1901652: response to peptide
GO:0051056: regulation of small GTPase mediated signal transduction
GO:0090263: positive regulation of canonical Wnt signaling pathway
GO:0051129: negative regulation of cellular component organization
GO:0034097: response to cytokine
GO:0007507: heart development
GO:1902532: negative regulation of intracellular signal transduction
hsa00410: beta-Alanine metabolism
GO:0006468: protein phosphorylation
GO:0042136: neurotransmitter biosynthetic process
GO:0090287: regulation of cellular response to growth factor stimulus
R-HSA-1280218: Adaptive Immune System
GO:0042987: amyloid precursor protein catabolic process
GO:0051014: actin filament severing
GO:0001934: positive regulation of protein phosphorylation
GO:0006631: fatty acid metabolic process
GO:0051346: negative regulation of hydrolase activity
GO:0046325: negative regulation of glucose import
GO:0046903: secretion
GO:0051261: protein depolymerization
hsa04210: Apoptosis
GO:0010876: lipid localization
GO:0045123: cellular extravasation
WP4148: Splicing factor NOVA regulated synaptic proteins
WP5115: Network map of SARS-CoV-2 signaling pathway
GO:0007568: aging
GO:0046716: muscle cell cellular homeostasis
R-HSA-194068: Bile acid and bile salt metabolism
GO:1902622: regulation of neutrophil migration
GO:0001890: placenta development
GO:1901615: organic hydroxy compound metabolic process
M239: PID A6B1 A6B4 INTEGRIN PATHWAY
R-HSA-9675108: Nervous system development
GO:0045732: positive regulation of protein catabolic process
GO:0033628: regulation of cell adhesion mediated by integrin
GO:0045664: regulation of neuron differentiation
GO:0098660: inorganic ion transmembrane transport
R-HSA-6811440: Retrograde transport at the Trans-Golgi-Network
M136: PID FOXO PATHWAY
GO:0098876: vesicle-mediated transport to the plasma membrane
R-HSA-418592: ADP signalling through P2Y purinoceptor 1
GO:0030278: regulation of ossification
M185: PID ALK1 PATHWAY
GO:0035640: exploration behavior
GO:0042278: purine nucleoside metabolic process
GO:0031503: protein-containing complex localization
GO:0060322: head development
GO:0051058: negative regulation of small GTPase mediated signal transduction
GO:0090303: positive regulation of wound healing
GO:0042246: tissue regeneration
GO:0050878: regulation of body fluid levels
R-HSA-1251985: Nuclear signaling by ERBB4
hsa04927: Cortisol synthesis and secretion
hsa04972: Pancreatic secretion
GO:0034260: negative regulation of GTPase activity
GO:0043123: positive regulation of I-kappaB kinase/NF-kappaB signaling
0
1
2
3
4
5
6
7
-log10(P)
