# Supplementary material for: Transcriptional profiling of Hutchinson-Gilford Progeria syndrome fibroblasts reveals deficits in mesenchymal stem cell commitment to differentiation related to early events in endochondral ossification
Source: eLife. 2022 Dec 29;11:e81290. doi: 10.7554/eLife.81290 (PMC9833827; doi:10.7554/eLife.81290)
Supplement: Table 2—source data 1. — Early and late childhood patient samples compared to healthy children, middle aged, and older adults. [file elife-81290-table2-data1.zip › Table_2-Source_Data_1/0-3-O-UP_646.zip!/Enrichment_GO/ColorByCluster.pdf]

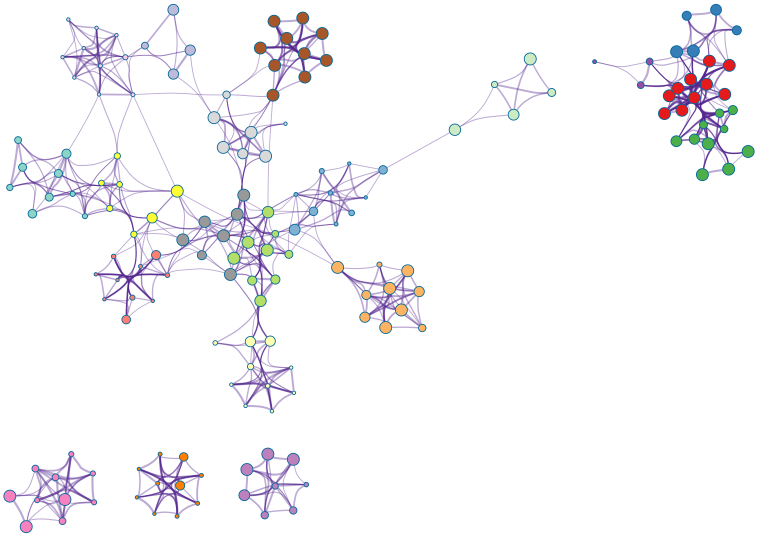

- HDACs deacetylate histones
- Metalloprotease DUBs
- Interleukin-7 signaling
- E3 ubiquitin ligases ubiquitinate target proteins
- Apoptosis induced DNA fragmentation
- Pathways in cancer
- cell projection morphogenesis
- Axon guidance
- transmembrane receptor protein tyrosine kinase sign[illegible]
- Hippo signaling pathway
- RUNX1 interacts with co-factors whose precise effect
- ossification
- NABA BASEMENT MEMBRANES
- thrombin-activated receptor signaling pathway
- cell activation
- MAPK family signaling cascades
- Hair follicle development: organogenesis - part 2 of 3
- blood vessel morphogenesis
- regulation of ion transmembrane transport
- negative regulation of developmental growth

created by
http://metascape.org
