# Supplementary material for: Transcriptional profiling of Hutchinson-Gilford Progeria syndrome fibroblasts reveals deficits in mesenchymal stem cell commitment to differentiation related to early events in endochondral ossification
Source: eLife. 2022 Dec 29;11:e81290. doi: 10.7554/eLife.81290 (PMC9833827; doi:10.7554/eLife.81290)
Supplement: Table 2—source data 1. — Early and late childhood patient samples compared to healthy children, middle aged, and older adults. [file elife-81290-table2-data1.zip › Table_2-Source_Data_1/0-3-O-UP_646.zip!/Enrichment_QC/HeatmapSelectedGO_Cell_Type_Signatures.pdf]

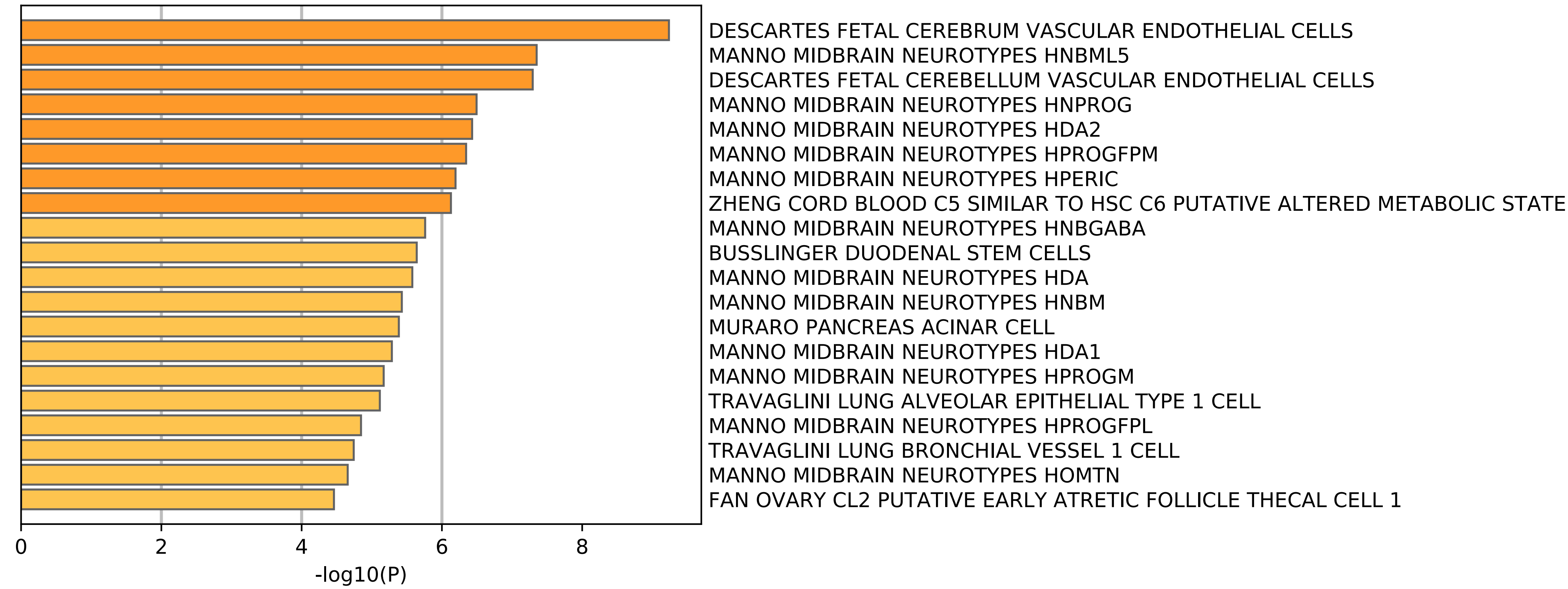
DESCARTES FETAL CEREBRUM VASCULAR ENDOTHELIAL CELLS
MANNO MIDBRAIN NEUROTYPES HNBML5
DESCARTES FETAL CEREBELLUM VASCULAR ENDOTHELIAL CELLS
MANNO MIDBRAIN NEUROTYPES HNPROG
MANNO MIDBRAIN NEUROTYPES HDA2
MANNO MIDBRAIN NEUROTYPES HPROGFPM
MANNO MIDBRAIN NEUROTYPES HPERIC
ZHENG CORD BLOOD C5 SIMILAR TO HSC C6 PUTATIVE ALTERED METABOLIC STATE
MANNO MIDBRAIN NEUROTYPES HNBGABA
BUSSLINGER DUODENAL STEM CELLS
MANNO MIDBRAIN NEUROTYPES HDA
MANNO MIDBRAIN NEUROTYPES HNBM
MURARO PANCREAS ACINAR CELL
MANNO MIDBRAIN NEUROTYPES HDA1
MANNO MIDBRAIN NEUROTYPES HPROGM
TRAVAGLINI LUNG ALVEOLAR EPITHELIAL TYPE 1 CELL
MANNO MIDBRAIN NEUROTYPES HPROGFPL
TRAVAGLINI LUNG BRONCHIAL VESSEL 1 CELL
MANNO MIDBRAIN NEUROTYPES HOMTN
FAN OVARY CL2 PUTATIVE EARLY ATRETIC FOLLICLE THECAL CELL 1
0
2
4
6
8
-log10(P)
