# Supplementary material for: Transcriptional profiling of Hutchinson-Gilford Progeria syndrome fibroblasts reveals deficits in mesenchymal stem cell commitment to differentiation related to early events in endochondral ossification
Source: eLife. 2022 Dec 29;11:e81290. doi: 10.7554/eLife.81290 (PMC9833827; doi:10.7554/eLife.81290)
Supplement: Table 2—source data 1. — Early and late childhood patient samples compared to healthy children, middle aged, and older adults. [file elife-81290-table2-data1.zip › Table_2-Source_Data_1/0-3-O-UP_646.zip!/Enrichment_QC/HeatmapSelectedGO_Transcription_Factor_Targets.pdf]

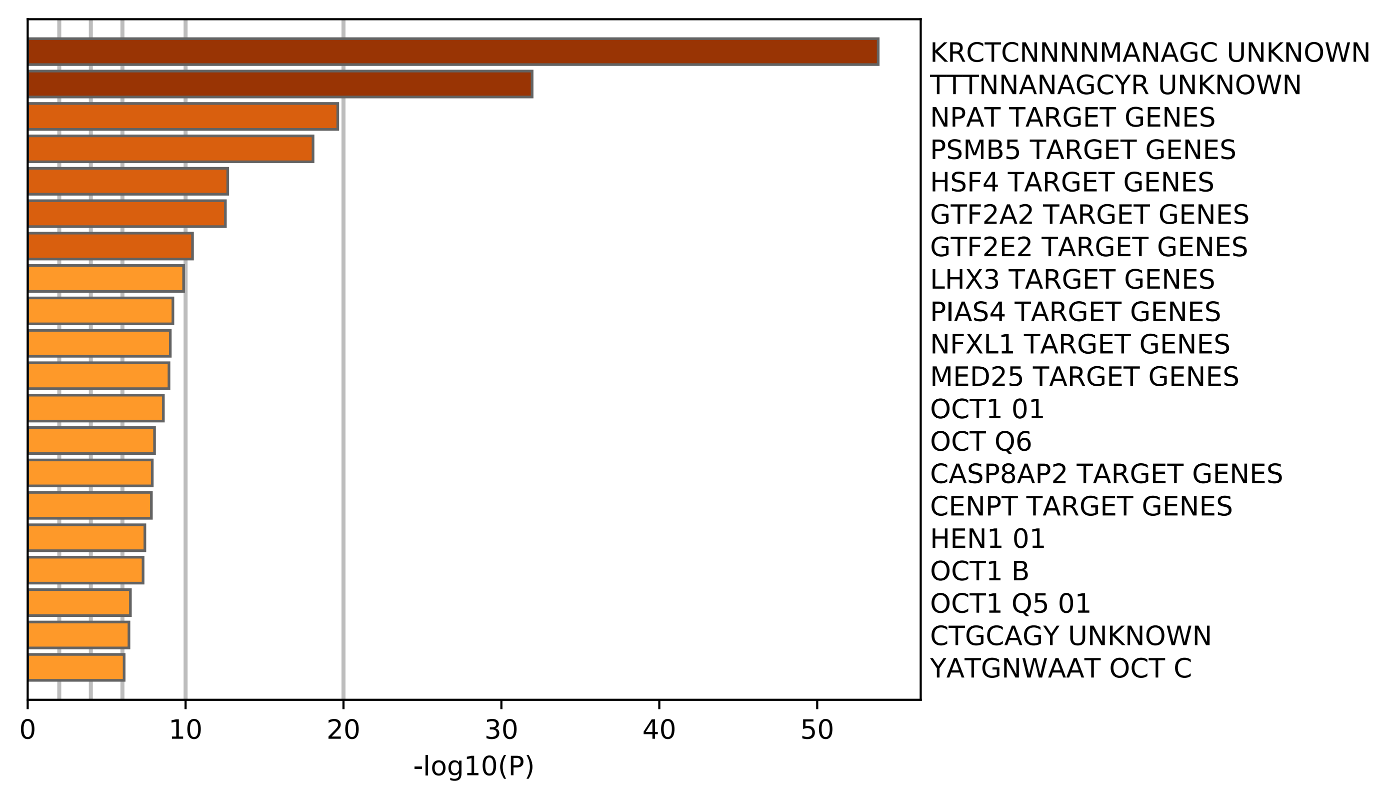
KRCTCNNNNMANAGC UNKNOWN
TTTNNANAGCYR UNKNOWN
NPAT TARGET GENES
PSMB5 TARGET GENES
HSF4 TARGET GENES
GTF2A2 TARGET GENES
GTF2E2 TARGET GENES
LHX3 TARGET GENES
PIAS4 TARGET GENES
NFXL1 TARGET GENES
MED25 TARGET GENES
OCT1 01
OCT Q6
CASP8AP2 TARGET GENES
CENPT TARGET GENES
HEN1 01
OCT1 B
OCT1 Q5 01
CTGCAGY UNKNOWN
YATGNWAAT OCT C
0
10
20
30
40
50
-log10(P)
