# Supplementary material for: Transcriptional profiling of Hutchinson-Gilford Progeria syndrome fibroblasts reveals deficits in mesenchymal stem cell commitment to differentiation related to early events in endochondral ossification
Source: eLife. 2022 Dec 29;11:e81290. doi: 10.7554/eLife.81290 (PMC9833827; doi:10.7554/eLife.81290)
Supplement: Table 2—source data 1. — Early and late childhood patient samples compared to healthy children, middle aged, and older adults. [file elife-81290-table2-data1.zip › Table_2-Source_Data_1/0-3-O-UP_646.zip!/Enrichment_QC/HeatmapSelectedGO_TRRUST.pdf]

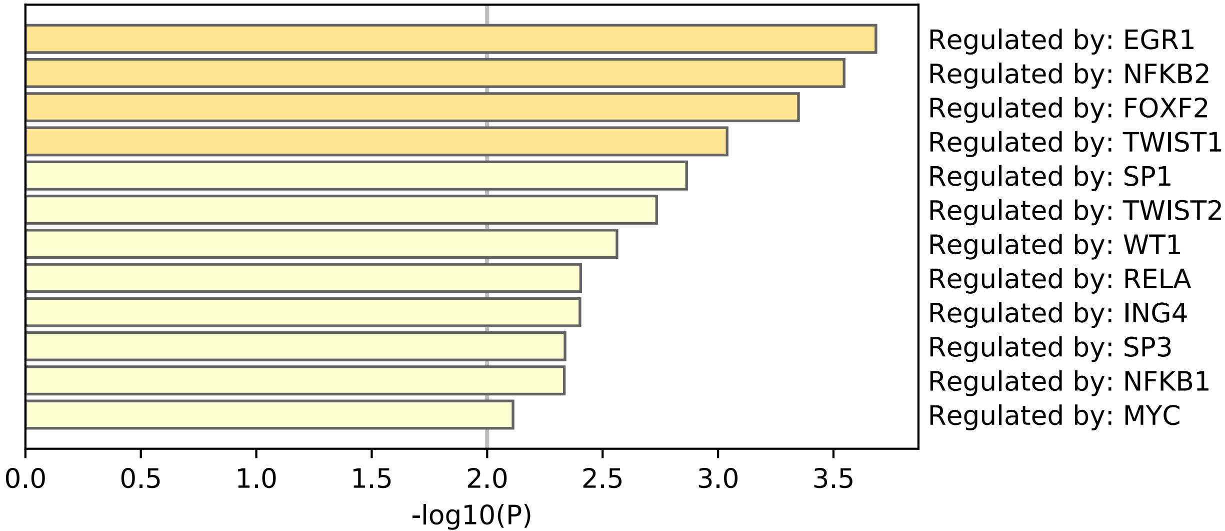
Regulated by: EGR1
Regulated by: NFKB2
Regulated by: FOXF2
Regulated by: TWIST1
Regulated by: SP1
Regulated by: TWIST2
Regulated by: WT1
Regulated by: RELA
Regulated by: ING4
Regulated by: SP3
Regulated by: NFKB1
Regulated by: MYC
0.0
0.5
1.0
1.5
2.0
2.5
3.0
3.5
-log10(P)
