# Supplementary material for: Transcriptional profiling of Hutchinson-Gilford Progeria syndrome fibroblasts reveals deficits in mesenchymal stem cell commitment to differentiation related to early events in endochondral ossification
Source: eLife. 2022 Dec 29;11:e81290. doi: 10.7554/eLife.81290 (PMC9833827; doi:10.7554/eLife.81290)
Supplement: Table 2—source data 1. — Early and late childhood patient samples compared to healthy children, middle aged, and older adults. [file elife-81290-table2-data1.zip › Table_2-Source_Data_1/0-3-O-UP_646.zip!/Enrichment_QC/HeatmapSelectedGO_DisGeNET.pdf]

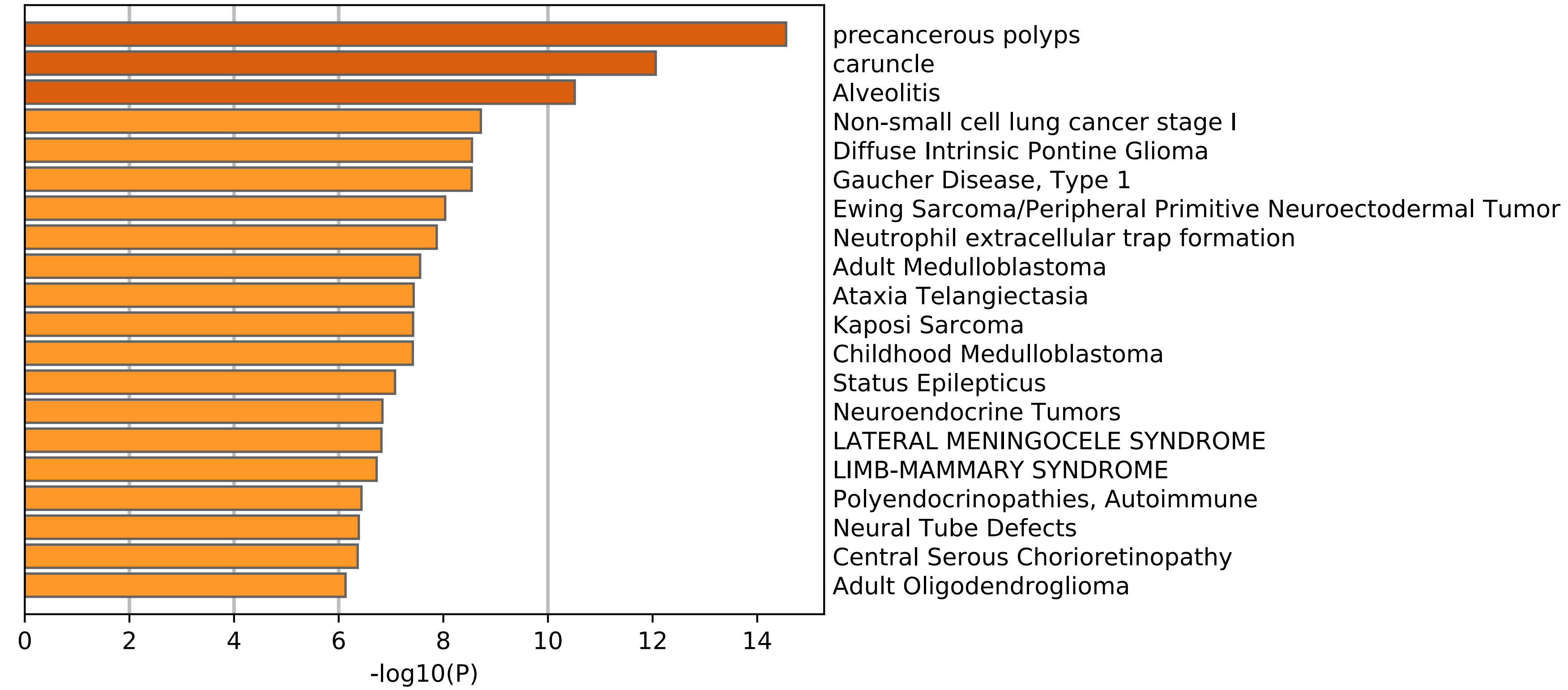
precancerous polyps
caruncle
Alveolitis
Non-small cell lung cancer stage I
Diffuse Intrinsic Pontine Glioma
Gaucher Disease, Type 1
Ewing Sarcoma/Peripheral Primitive Neuroectodermal Tumor
Neutrophil extracellular trap formation
Adult Medulloblastoma
Ataxia Telangiectasia
Kaposi Sarcoma
Childhood Medulloblastoma
Status Epilepticus
Neuroendocrine Tumors
LATERAL MENINGOCELE SYNDROME
LIMB-MAMMARY SYNDROME
Polyendocrinopathies, Autoimmune
Neural Tube Defects
Central Serous Chorioretinopathy
Adult Oligodendroglioma
0
2
4
6
8
10
12
14
-log10(P)
