# Supplementary material for: Transcriptional profiling of Hutchinson-Gilford Progeria syndrome fibroblasts reveals deficits in mesenchymal stem cell commitment to differentiation related to early events in endochondral ossification
Source: eLife. 2022 Dec 29;11:e81290. doi: 10.7554/eLife.81290 (PMC9833827; doi:10.7554/eLife.81290)
Supplement: Table 2—source data 1. — Early and late childhood patient samples compared to healthy children, middle aged, and older adults. [file elife-81290-table2-data1.zip › Table_2-Source_Data_1/0-3-O-UP_646.zip!/Enrichment_heatmap/HeatmapSelectedGO.pdf]

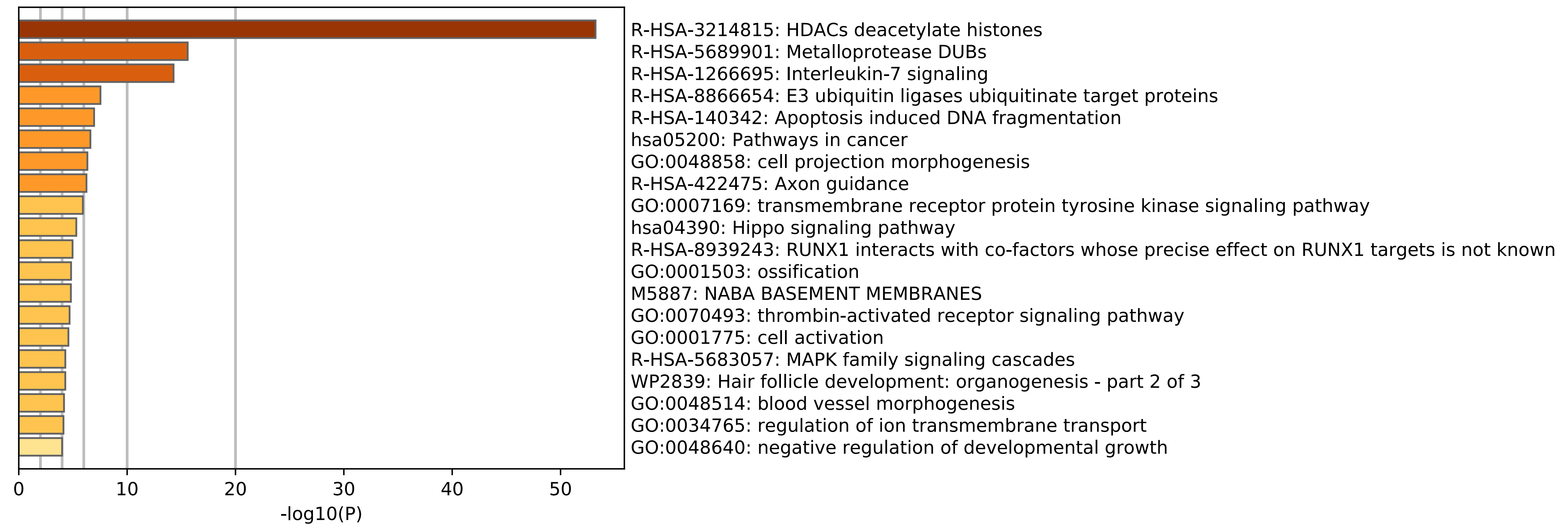
R-HSA-3214815: HDACs deacetylate histones
R-HSA-5689901: Metalloprotease DUBs
R-HSA-1266695: Interleukin-7 signaling
R-HSA-8866654: E3 ubiquitin ligases ubiquitinate target proteins
R-HSA-140342: Apoptosis induced DNA fragmentation
hsa05200: Pathways in cancer
GO:0048858: cell projection morphogenesis
R-HSA-422475: Axon guidance
GO:0007169: transmembrane receptor protein tyrosine kinase signaling pathway
hsa04390: Hippo signaling pathway
R-HSA-8939243: RUNX1 interacts with co-factors whose precise effect on RUNX1 targets is not known
GO:0001503: ossification
M5887: NABA BASEMENT MEMBRANES
GO:0070493: thrombin-activated receptor signaling pathway
GO:0001775: cell activation
R-HSA-5683057: MAPK family signaling cascades
WP2839: Hair follicle development: organogenesis - part 2 of 3
GO:0048514: blood vessel morphogenesis
GO:0034765: regulation of ion transmembrane transport
GO:0048640: negative regulation of developmental growth
0
10
20
30
40
50
-log10(P)
