# Supplementary material for: Transcriptional profiling of Hutchinson-Gilford Progeria syndrome fibroblasts reveals deficits in mesenchymal stem cell commitment to differentiation related to early events in endochondral ossification
Source: eLife. 2022 Dec 29;11:e81290. doi: 10.7554/eLife.81290 (PMC9833827; doi:10.7554/eLife.81290)
Supplement: Table 2—source data 1. — Early and late childhood patient samples compared to healthy children, middle aged, and older adults. [file elife-81290-table2-data1.zip › Table_2-Source_Data_1/0-3-O-UP_646.zip!/Enrichment_heatmap/HeatmapSelectedGOTop100.pdf]

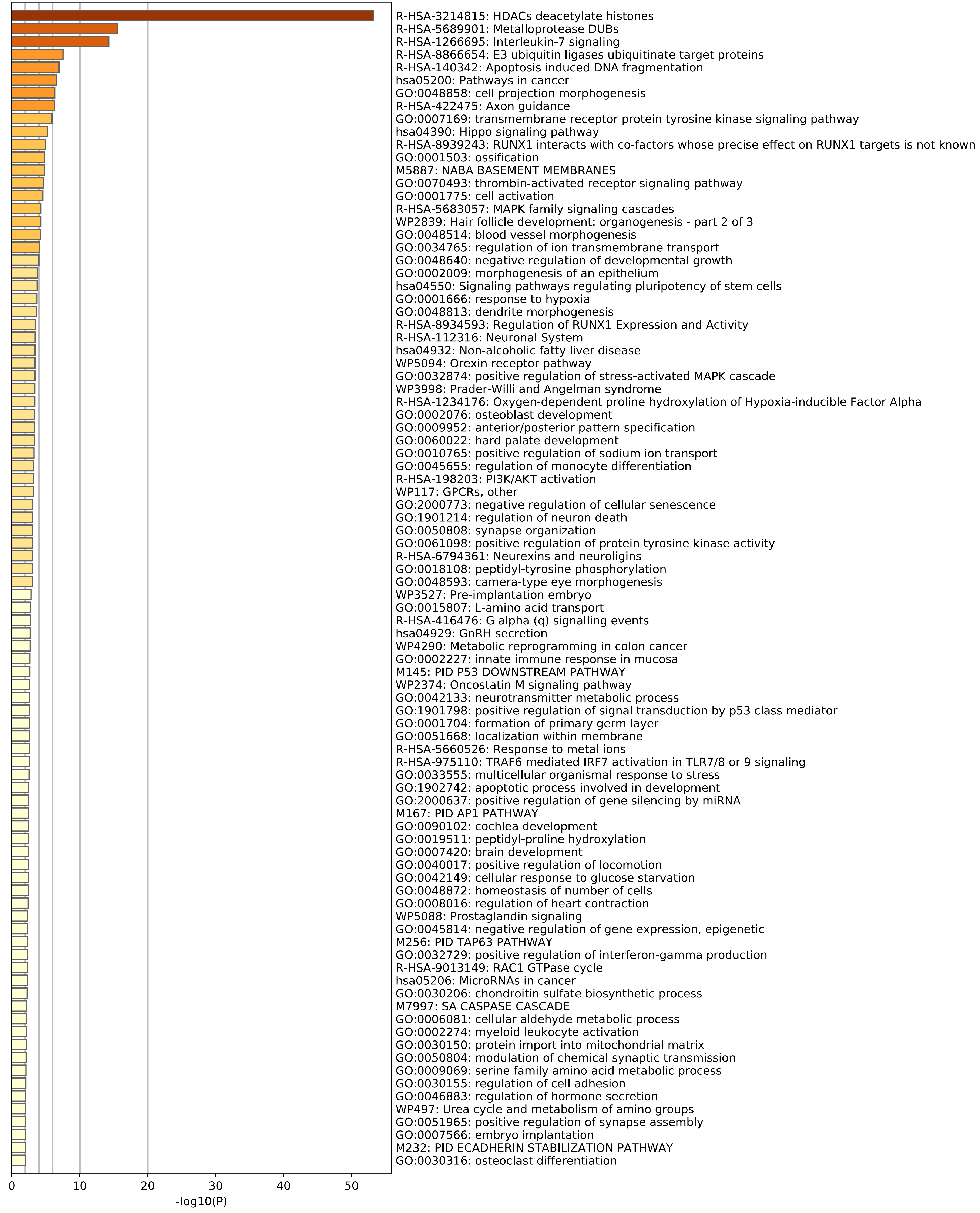
R-HSA-3214815: HDACs deacetylate histones
R-HSA-5689901: Metalloprotease DUBs
R-HSA-1266695: Interleukin-7 signaling
R-HSA-8866654: E3 ubiquitin ligases ubiquitinate target proteins
R-HSA-140342: Apoptosis induced DNA fragmentation
hsa05200: Pathways in cancer
GO:0048858: cell projection morphogenesis
R-HSA-422475: Axon guidance
GO:0007169: transmembrane receptor protein tyrosine kinase signaling pathway
hsa04390: Hippo signaling pathway
R-HSA-8939243: RUNX1 interacts with co-factors whose precise effect on RUNX1 targets is not known
GO:0001503: ossification
M5887: NABA BASEMENT MEMBRANES
GO:0070493: thrombin-activated receptor signaling pathway
GO:0001775: cell activation
R-HSA-5683057: MAPK family signaling cascades
WP2839: Hair follicle development: organogenesis - part 2 of 3
GO:0048514: blood vessel morphogenesis
GO:0034765: regulation of ion transmembrane transport
GO:0048640: negative regulation of developmental growth
GO:0002009: morphogenesis of an epithelium
hsa04550: Signaling pathways regulating pluripotency of stem cells
GO:0001666: response to hypoxia
GO:0048813: dendrite morphogenesis
R-HSA-8934593: Regulation of RUNX1 Expression and Activity
R-HSA-112316: Neuronal System
hsa04932: Non-alcoholic fatty liver disease
WP5094: Orexin receptor pathway
GO:0032874: positive regulation of stress-activated MAPK cascade
WP3998: Prader-Willi and Angelman syndrome
R-HSA-1234176: Oxygen-dependent proline hydroxylation of Hypoxia-inducible Factor Alpha
GO:0002076: osteoblast development
GO:0009952: anterior/posterior pattern specification
GO:0060022: hard palate development
GO:0010765: positive regulation of sodium ion transport
GO:0045655: regulation of monocyte differentiation
R-HSA-198203: PI3K/AKT activation
WP117: GPCRs, other
GO:2000773: negative regulation of cellular senescence
GO:1901214: regulation of neuron death
GO:0050808: synapse organization
GO:0061098: positive regulation of protein tyrosine kinase activity
R-HSA-6794361: Neurexins and neuroligins
GO:0018108: peptidyl-tyrosine phosphorylation
GO:0048593: camera-type eye morphogenesis
WP3527: Pre-implantation embryo
GO:0015807: L-amino acid transport
R-HSA-416476: G alpha (q) signalling events
hsa04929: GnRH secretion
WP4290: Metabolic reprogramming in colon cancer
GO:0002227: innate immune response in mucosa
M145: PID P53 DOWNSTREAM PATHWAY
WP2374: Oncostatin M signaling pathway
GO:0042133: neurotransmitter metabolic process
GO:1901798: positive regulation of signal transduction by p53 class mediator
GO:0001704: formation of primary germ layer
GO:0051668: localization within membrane
R-HSA-5660526: Response to metal ions
R-HSA-975110: TRAF6 mediated IRF7 activation in TLR7/8 or 9 signaling
GO:0033555: multicellular organismal response to stress
GO:1902742: apoptotic process involved in development
GO:2000637: positive regulation of gene silencing by miRNA
M167: PID AP1 PATHWAY
GO:0090102: cochlea development
GO:0019511: peptidyl-proline hydroxylation
GO:0007420: brain development
GO:0040017: positive regulation of locomotion
GO:0042149: cellular response to glucose starvation
GO:0048872: homeostasis of number of cells
GO:0008016: regulation of heart contraction
WP5088: Prostaglandin signaling
GO:0045814: negative regulation of gene expression, epigenetic
M256: PID TAP63 PATHWAY
GO:0032729: positive regulation of interferon-gamma production
R-HSA-9013149: RAC1 GTPase cycle
hsa05206: MicroRNAs in cancer
GO:0030206: chondroitin sulfate biosynthetic process
M7997: SA CASPASE CASCADE
GO:0006081: cellular aldehyde metabolic process
GO:0002274: myeloid leukocyte activation
GO:0030150: protein import into mitochondrial matrix
GO:0050804: modulation of chemical synaptic transmission
GO:0009069: serine family amino acid metabolic process
GO:0030155: regulation of cell adhesion
GO:0046883: regulation of hormone secretion
WP497: Urea cycle and metabolism of amino groups
GO:0051965: positive regulation of synapse assembly
GO:0007566: embryo implantation
M232: PID ECADHERIN STABILIZATION PATHWAY
GO:0030316: osteoclast differentiation
0
10
20
30
40
50
-log10(P)
