# Supplementary material for: Transcriptional profiling of Hutchinson-Gilford Progeria syndrome fibroblasts reveals deficits in mesenchymal stem cell commitment to differentiation related to early events in endochondral ossification
Source: eLife. 2022 Dec 29;11:e81290. doi: 10.7554/eLife.81290 (PMC9833827; doi:10.7554/eLife.81290)
Supplement: Table 2—source data 1. — Early and late childhood patient samples compared to healthy children, middle aged, and older adults. [file elife-81290-table2-data1.zip › Table_2-Source_Data_1/4-7-AM-DOWN_102.zip!/Enrichment_GO/ColorByCluster.pdf]

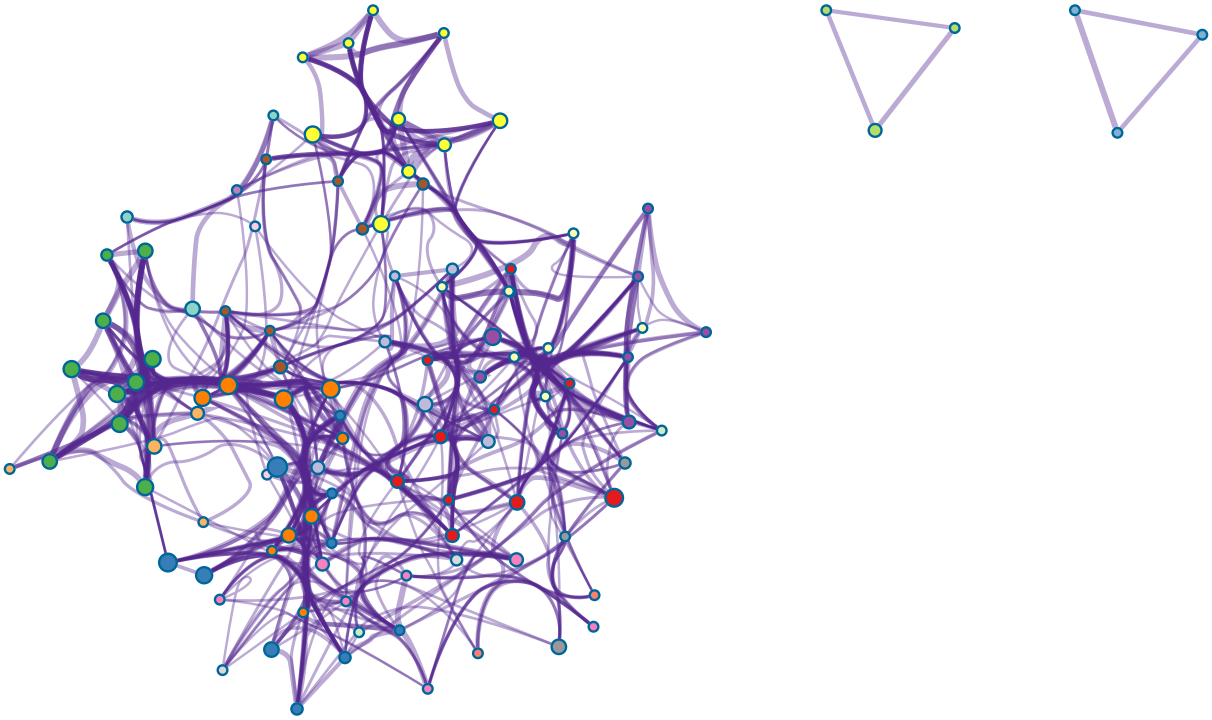

ossification
regulation of secretion
calcium ion homeostasis
embryonic organ development
positive regulation of cell motility
regulation of actin filament organization
endothelial cell differentiation
negative regulation of cell development
Extracellular matrix organization
positive regulation of hydrolase activity
positive regulation of epithelial to mesenchymal trans
angiogenesis
osteoblast differentiation
carboxylic acid transport
regulation of G protein-coupled receptor signaling pa
nucleic acid phosphodiester bond hydrolysis
IL-18 signaling pathway
regulation of nucleotide metabolic process
Axon guidance
regulation of cell division

created by
http://metascape.org
