# Supplementary material for: Transcriptional profiling of Hutchinson-Gilford Progeria syndrome fibroblasts reveals deficits in mesenchymal stem cell commitment to differentiation related to early events in endochondral ossification
Source: eLife. 2022 Dec 29;11:e81290. doi: 10.7554/eLife.81290 (PMC9833827; doi:10.7554/eLife.81290)
Supplement: Table 2—source data 1. — Early and late childhood patient samples compared to healthy children, middle aged, and older adults. [file elife-81290-table2-data1.zip › Table_2-Source_Data_1/4-7-AM-DOWN_102.zip!/Enrichment_heatmap/HeatmapSelectedGO.pdf]

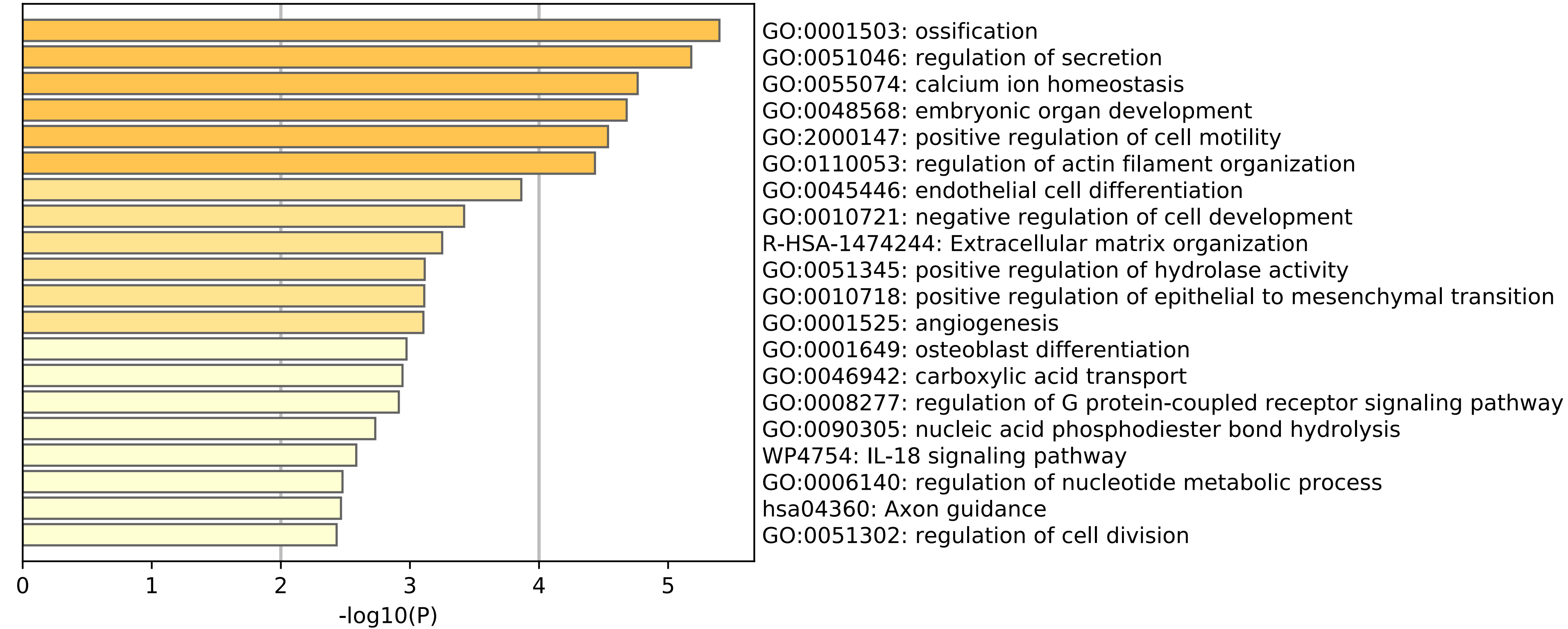
GO:0001503: ossification
GO:0051046: regulation of secretion
GO:0055074: calcium ion homeostasis
GO:0048568: embryonic organ development
GO:2000147: positive regulation of cell motility
GO:0110053: regulation of actin filament organization
GO:0045446: endothelial cell differentiation
GO:0010721: negative regulation of cell development
R-HSA-1474244: Extracellular matrix organization
GO:0051345: positive regulation of hydrolase activity
GO:0010718: positive regulation of epithelial to mesenchymal transition
GO:0001525: angiogenesis
GO:0001649: osteoblast differentiation
GO:0046942: carboxylic acid transport
GO:0008277: regulation of G protein-coupled receptor signaling pathway
GO:0090305: nucleic acid phosphodiester bond hydrolysis
WP4754: IL-18 signaling pathway
GO:0006140: regulation of nucleotide metabolic process
hsa04360: Axon guidance
GO:0051302: regulation of cell division
0
1
2
3
4
5
-log10(P)
