# Supplementary material for: Transcriptional profiling of Hutchinson-Gilford Progeria syndrome fibroblasts reveals deficits in mesenchymal stem cell commitment to differentiation related to early events in endochondral ossification
Source: eLife. 2022 Dec 29;11:e81290. doi: 10.7554/eLife.81290 (PMC9833827; doi:10.7554/eLife.81290)
Supplement: Table 2—source data 1. — Early and late childhood patient samples compared to healthy children, middle aged, and older adults. [file elife-81290-table2-data1.zip › Table_2-Source_Data_1/4-7-AM-UP-203.zip!/Enrichment_GO/ColorByCluster.pdf]

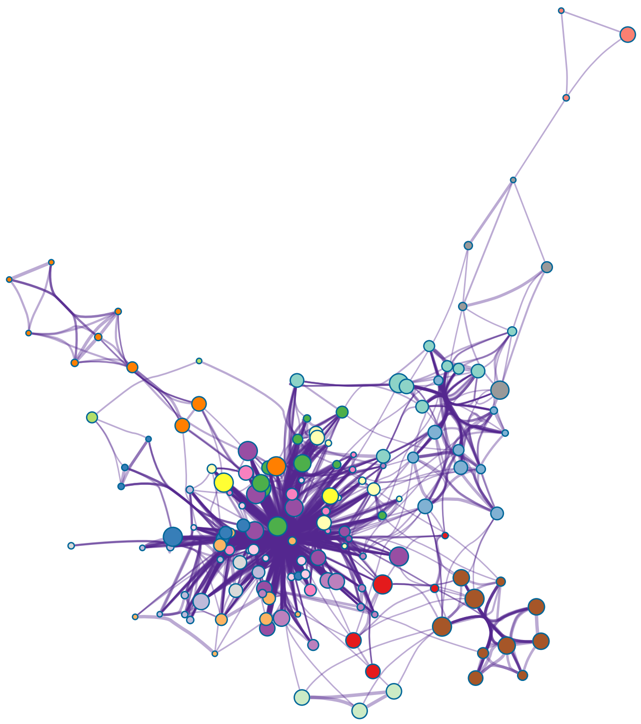

- enzyme linked receptor protein signaling pathway
- muscle structure development
- tube morphogenesis
- tissue morphogenesis
- regulation of cell adhesion
- Pathways in cancer
- positive regulation of protein phosphorylation
- stem cell differentiation
- cell-cell adhesion
- cell morphogenesis
- negative regulation of cellular component movement
- skeletal system development
- Malignant pleural mesothelioma
- positive regulation of cell development
- renal system development
- response to mechanical stimulus
- cardiac chamber morphogenesis
- developmental growth
- homeostasis of number of cells
- positive regulation of apoptotic process

created by
http://metascape.org
