# Supplementary material for: Transcriptional profiling of Hutchinson-Gilford Progeria syndrome fibroblasts reveals deficits in mesenchymal stem cell commitment to differentiation related to early events in endochondral ossification
Source: eLife. 2022 Dec 29;11:e81290. doi: 10.7554/eLife.81290 (PMC9833827; doi:10.7554/eLife.81290)
Supplement: Table 2—source data 1. — Early and late childhood patient samples compared to healthy children, middle aged, and older adults. [file elife-81290-table2-data1.zip › Table_2-Source_Data_1/4-7-AM-UP-203.zip!/Enrichment_heatmap/HeatmapSelectedGO.pdf]

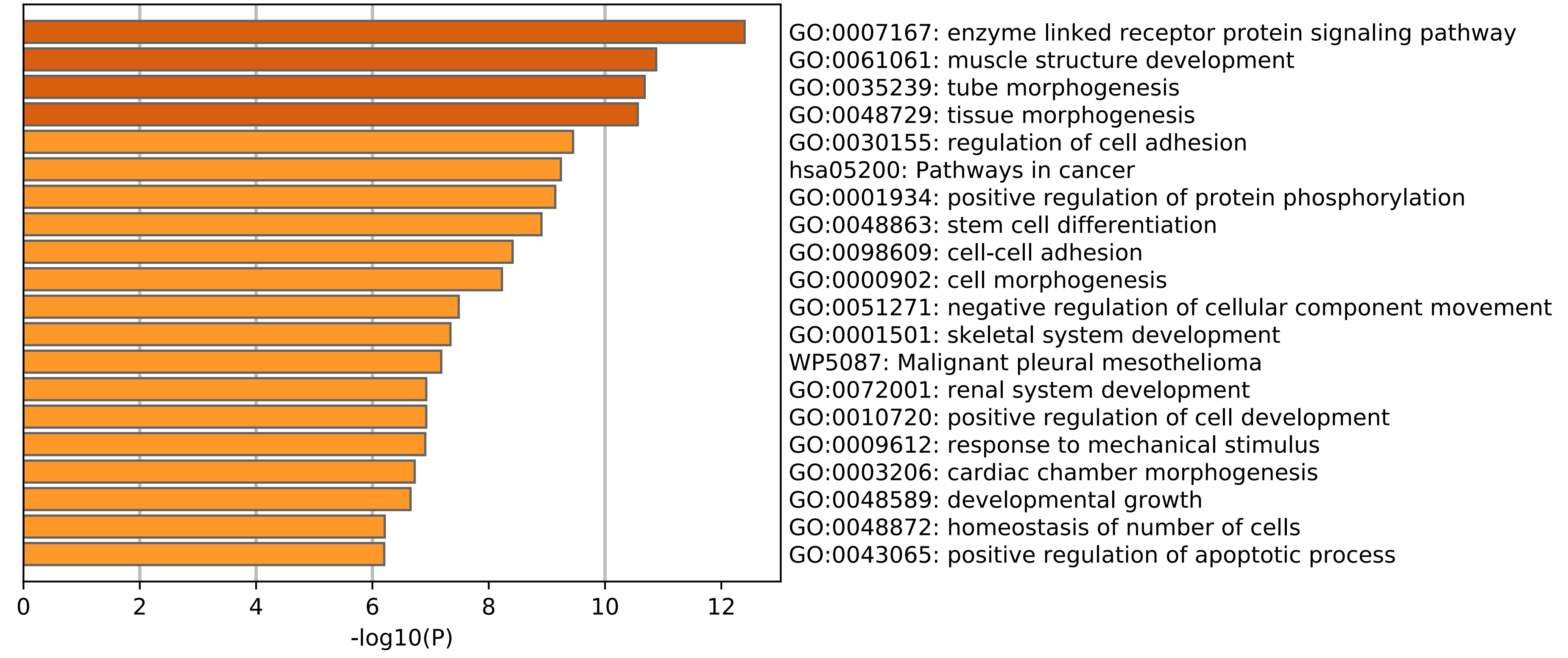
GO:0007167: enzyme linked receptor protein signaling pathway
GO:0061061: muscle structure development
GO:0035239: tube morphogenesis
GO:0048729: tissue morphogenesis
GO:0030155: regulation of cell adhesion
hsa05200: Pathways in cancer
GO:0001934: positive regulation of protein phosphorylation
GO:0048863: stem cell differentiation
GO:0098609: cell-cell adhesion
GO:0000902: cell morphogenesis
GO:0051271: negative regulation of cellular component movement
GO:0001501: skeletal system development
WP5087: Malignant pleural mesothelioma
GO:0072001: renal system development
GO:0010720: positive regulation of cell development
GO:0009612: response to mechanical stimulus
GO:0003206: cardiac chamber morphogenesis
GO:0048589: developmental growth
GO:0048872: homeostasis of number of cells
GO:0043065: positive regulation of apoptotic process
0
2
4
6
8
10
12
-log10(P)
