# Supplementary material for: Transcriptional profiling of Hutchinson-Gilford Progeria syndrome fibroblasts reveals deficits in mesenchymal stem cell commitment to differentiation related to early events in endochondral ossification
Source: eLife. 2022 Dec 29;11:e81290. doi: 10.7554/eLife.81290 (PMC9833827; doi:10.7554/eLife.81290)
Supplement: Table 2—source data 1. — Early and late childhood patient samples compared to healthy children, middle aged, and older adults. [file elife-81290-table2-data1.zip › Table_2-Source_Data_1/4-7-AM-UP-203.zip!/Enrichment_heatmap/HeatmapSelectedGOTop100.pdf]

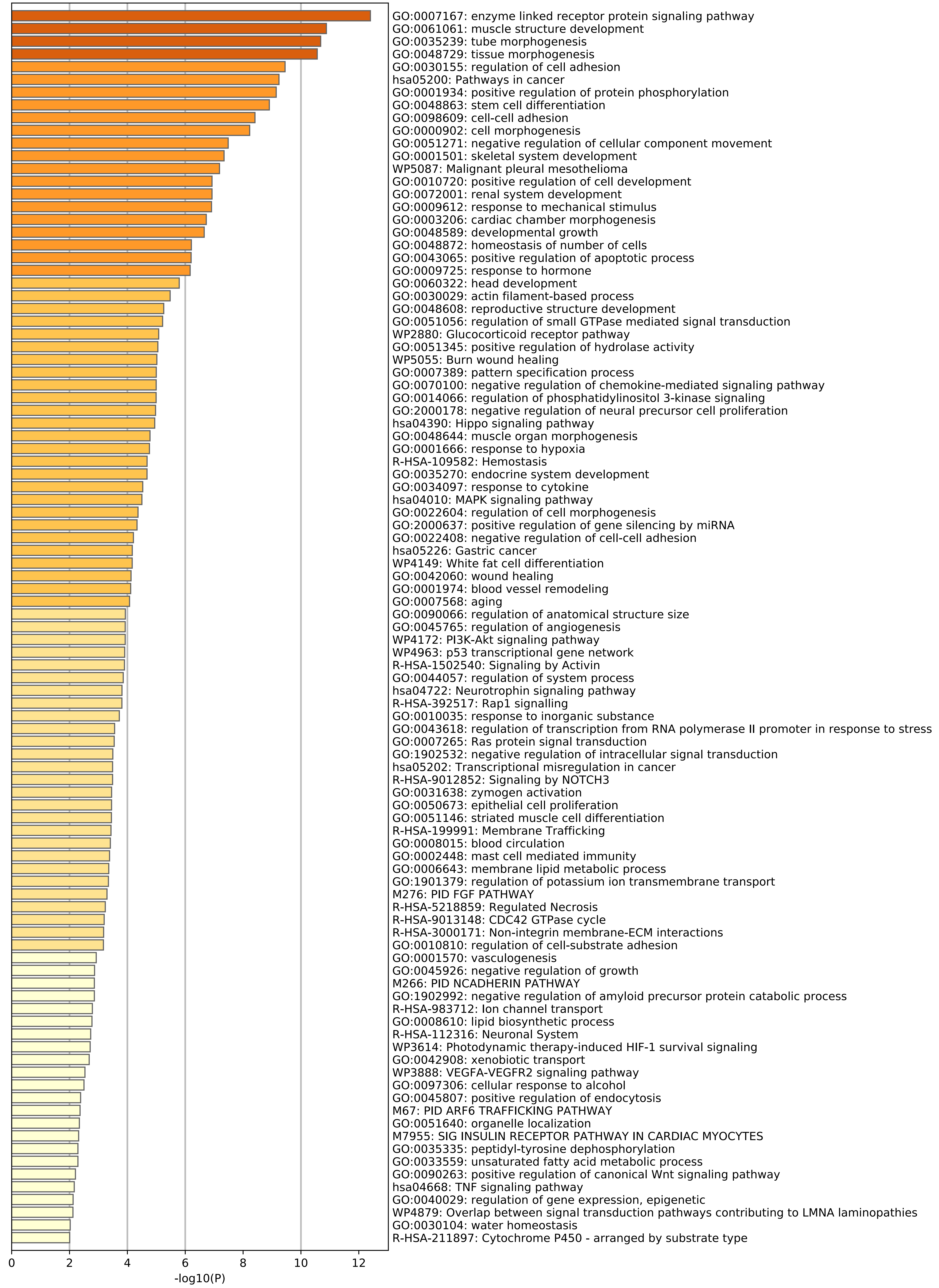
GO:0007167: enzyme linked receptor protein signaling pathway
GO:0061061: muscle structure development
GO:0035239: tube morphogenesis
GO:0048729: tissue morphogenesis
GO:0030155: regulation of cell adhesion
hsa05200: Pathways in cancer
GO:0001934: positive regulation of protein phosphorylation
GO:0048863: stem cell differentiation
GO:0098609: cell-cell adhesion
GO:0000902: cell morphogenesis
GO:0051271: negative regulation of cellular component movement
GO:0001501: skeletal system development
WP5087: Malignant pleural mesothelioma
GO:0010720: positive regulation of cell development
GO:0072001: renal system development
GO:0009612: response to mechanical stimulus
GO:0003206: cardiac chamber morphogenesis
GO:0048589: developmental growth
GO:0048872: homeostasis of number of cells
GO:0043065: positive regulation of apoptotic process
GO:0009725: response to hormone
GO:0060322: head development
GO:0030029: actin filament-based process
GO:0048608: reproductive structure development
GO:0051056: regulation of small GTPase mediated signal transduction
WP2880: Glucocorticoid receptor pathway
GO:0051345: positive regulation of hydrolase activity
WP5055: Burn wound healing
GO:0007389: pattern specification process
GO:0070100: negative regulation of chemokine-mediated signaling pathway
GO:0014066: regulation of phosphatidylinositol 3-kinase signaling
GO:2000178: negative regulation of neural precursor cell proliferation
hsa04390: Hippo signaling pathway
GO:0048644: muscle organ morphogenesis
GO:0001666: response to hypoxia
R-HSA-109582: Hemostasis
GO:0035270: endocrine system development
GO:0034097: response to cytokine
hsa04010: MAPK signaling pathway
GO:0022604: regulation of cell morphogenesis
GO:2000637: positive regulation of gene silencing by miRNA
GO:0022408: negative regulation of cell-cell adhesion
hsa05226: Gastric cancer
WP4149: White fat cell differentiation
GO:0042060: wound healing
GO:0001974: blood vessel remodeling
GO:0007568: aging
GO:0090066: regulation of anatomical structure size
GO:0045765: regulation of angiogenesis
WP4172: PI3K-Akt signaling pathway
WP4963: p53 transcriptional gene network
R-HSA-1502540: Signaling by Activin
GO:0044057: regulation of system process
hsa04722: Neurotrophin signaling pathway
R-HSA-392517: Rap1 signalling
GO:0010035: response to inorganic substance
GO:0043618: regulation of transcription from RNA polymerase II promoter in response to stress
GO:0007265: Ras protein signal transduction
GO:1902532: negative regulation of intracellular signal transduction
hsa05202: Transcriptional misregulation in cancer
R-HSA-9012852: Signaling by NOTCH3
GO:0031638: zymogen activation
GO:0050673: epithelial cell proliferation
GO:0051146: striated muscle cell differentiation
R-HSA-199991: Membrane Trafficking
GO:0008015: blood circulation
GO:0002448: mast cell mediated immunity
GO:0006643: membrane lipid metabolic process
GO:1901379: regulation of potassium ion transmembrane transport
M276: PID FGF PATHWAY
R-HSA-5218859: Regulated Necrosis
R-HSA-9013148: CDC42 GTPase cycle
R-HSA-3000171: Non-integrin membrane-ECM interactions
GO:0010810: regulation of cell-substrate adhesion
GO:0001570: vasculogenesis
GO:0045926: negative regulation of growth
M266: PID NCADHERIN PATHWAY
GO:1902992: negative regulation of amyloid precursor protein catabolic process
R-HSA-983712: Ion channel transport
GO:0008610: lipid biosynthetic process
R-HSA-112316: Neuronal System
WP3614: Photodynamic therapy-induced HIF-1 survival signaling
GO:0042908: xenobiotic transport
WP3888: VEGFA-VEGFR2 signaling pathway
GO:0097306: cellular response to alcohol
GO:0045807: positive regulation of endocytosis
M67: PID ARF6 TRAFFICKING PATHWAY
GO:0051640: organelle localization
M7955: SIG INSULIN RECEPTOR PATHWAY IN CARDIAC MYOCYTES
GO:0035335: peptidyl-tyrosine dephosphorylation
GO:0033559: unsaturated fatty acid metabolic process
GO:0090263: positive regulation of canonical Wnt signaling pathway
hsa04668: TNF signaling pathway
GO:0040029: regulation of gene expression, epigenetic
WP4879: Overlap between signal transduction pathways contributing to LMNA laminopathies
GO:0030104: water homeostasis
R-HSA-211897: Cytochrome P450 - arranged by substrate type
0
2
4
6
8
10
12
-log10(P)
