# Supplementary material for: Transcriptional profiling of Hutchinson-Gilford Progeria syndrome fibroblasts reveals deficits in mesenchymal stem cell commitment to differentiation related to early events in endochondral ossification
Source: eLife. 2022 Dec 29;11:e81290. doi: 10.7554/eLife.81290 (PMC9833827; doi:10.7554/eLife.81290)
Supplement: Table 2—source data 1. — Early and late childhood patient samples compared to healthy children, middle aged, and older adults. [file elife-81290-table2-data1.zip › Table_2-Source_Data_1/4-7-M-DOWN-184.zip!/Enrichment_GO/ColorByCluster.pdf]

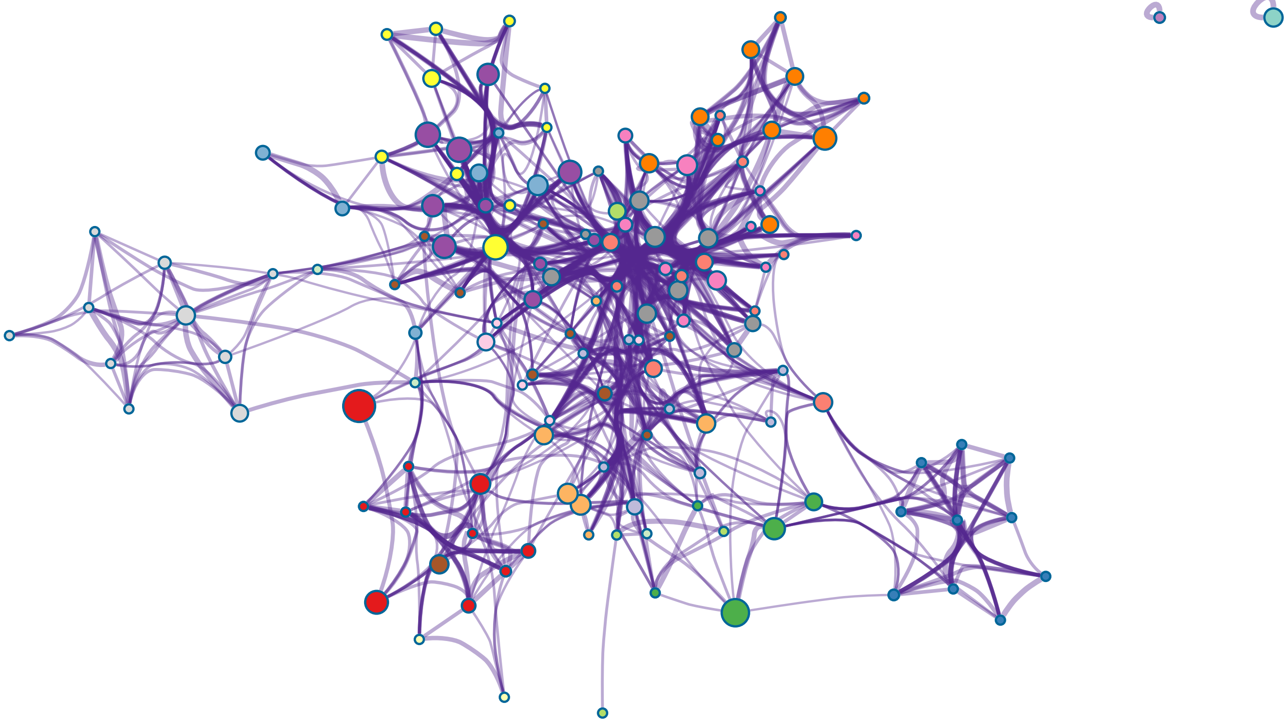

- NABA MATRISOME ASSOCIATED
- Prostaglandin synthesis and regulation
- response to hormone
- positive regulation of cellular component movement
- sensory organ morphogenesis
- negative regulation of cell differentiation
- negative regulation of lipid biosynthetic process
- pattern specification process
- morphogenesis of an epithelium
- Neutrophil degranulation
- Collagen formation
- regulation of fat cell differentiation
- reproductive structure development
- negative regulation of response to external stimulus
- positive regulation of cell death
- Copper homeostasis
- Malignant pleural mesothelioma
- Signaling by Interleukins
- carbohydrate derivative catabolic process
- Interleukin-4 and Interleukin-13 signaling

created by
http://metascape.org
