# Supplementary material for: Transcriptional profiling of Hutchinson-Gilford Progeria syndrome fibroblasts reveals deficits in mesenchymal stem cell commitment to differentiation related to early events in endochondral ossification
Source: eLife. 2022 Dec 29;11:e81290. doi: 10.7554/eLife.81290 (PMC9833827; doi:10.7554/eLife.81290)
Supplement: Table 2—source data 1. — Early and late childhood patient samples compared to healthy children, middle aged, and older adults. [file elife-81290-table2-data1.zip › Table_2-Source_Data_1/4-7-M-DOWN-184.zip!/Enrichment_heatmap/HeatmapSelectedGO.pdf]

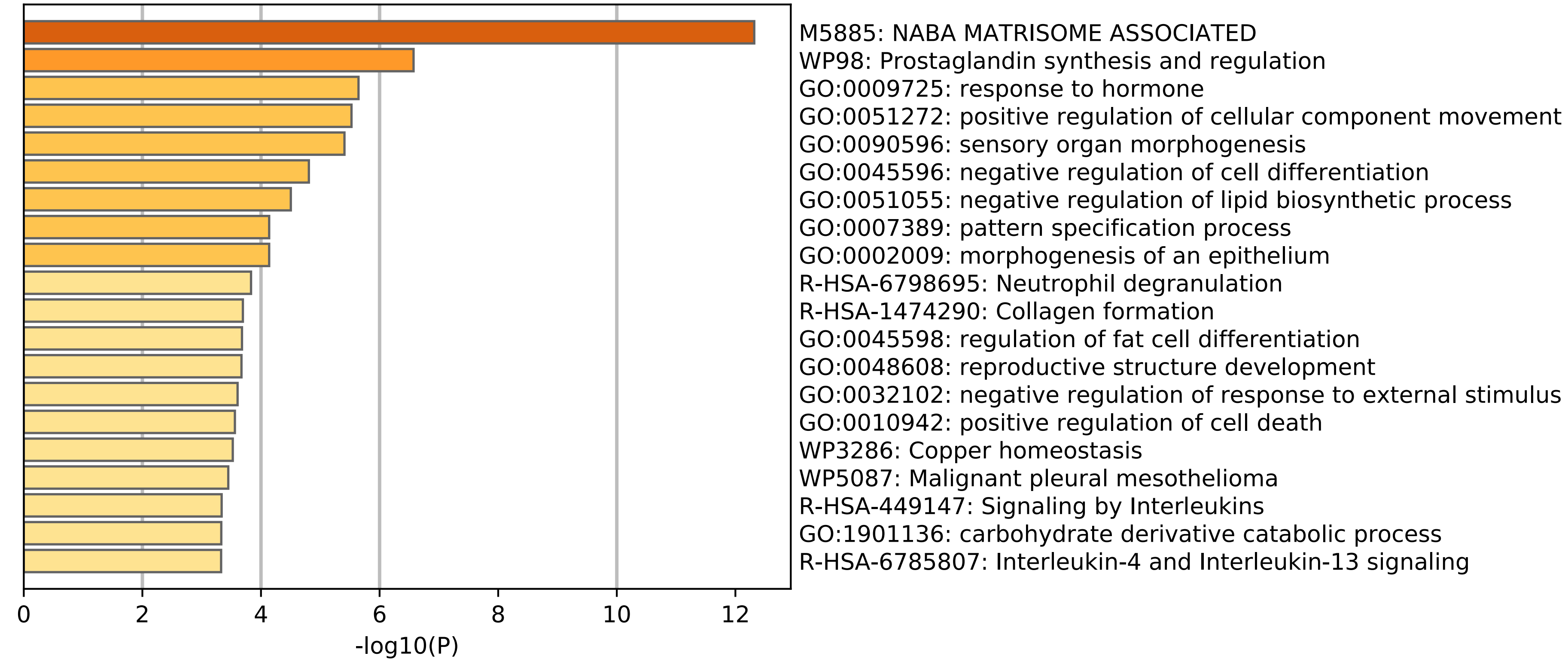
M5885: NABA MATRISOME ASSOCIATED
WP98: Prostaglandin synthesis and regulation
GO:0009725: response to hormone
GO:0051272: positive regulation of cellular component movement
GO:0090596: sensory organ morphogenesis
GO:0045596: negative regulation of cell differentiation
GO:0051055: negative regulation of lipid biosynthetic process
GO:0007389: pattern specification process
GO:0002009: morphogenesis of an epithelium
R-HSA-6798695: Neutrophil degranulation
R-HSA-1474290: Collagen formation
GO:0045598: regulation of fat cell differentiation
GO:0048608: reproductive structure development
GO:0032102: negative regulation of response to external stimulus
GO:0010942: positive regulation of cell death
WP3286: Copper homeostasis
WP5087: Malignant pleural mesothelioma
R-HSA-449147: Signaling by Interleukins
GO:1901136: carbohydrate derivative catabolic process
R-HSA-6785807: Interleukin-4 and Interleukin-13 signaling
0
2
4
6
8
10
12
-log10(P)
