# Supplementary material for: Transcriptional profiling of Hutchinson-Gilford Progeria syndrome fibroblasts reveals deficits in mesenchymal stem cell commitment to differentiation related to early events in endochondral ossification
Source: eLife. 2022 Dec 29;11:e81290. doi: 10.7554/eLife.81290 (PMC9833827; doi:10.7554/eLife.81290)
Supplement: Table 2—source data 1. — Early and late childhood patient samples compared to healthy children, middle aged, and older adults. [file elife-81290-table2-data1.zip › Table_2-Source_Data_1/4-7-M-DOWN-184.zip!/Enrichment_heatmap/HeatmapSelectedGOTop100.pdf]

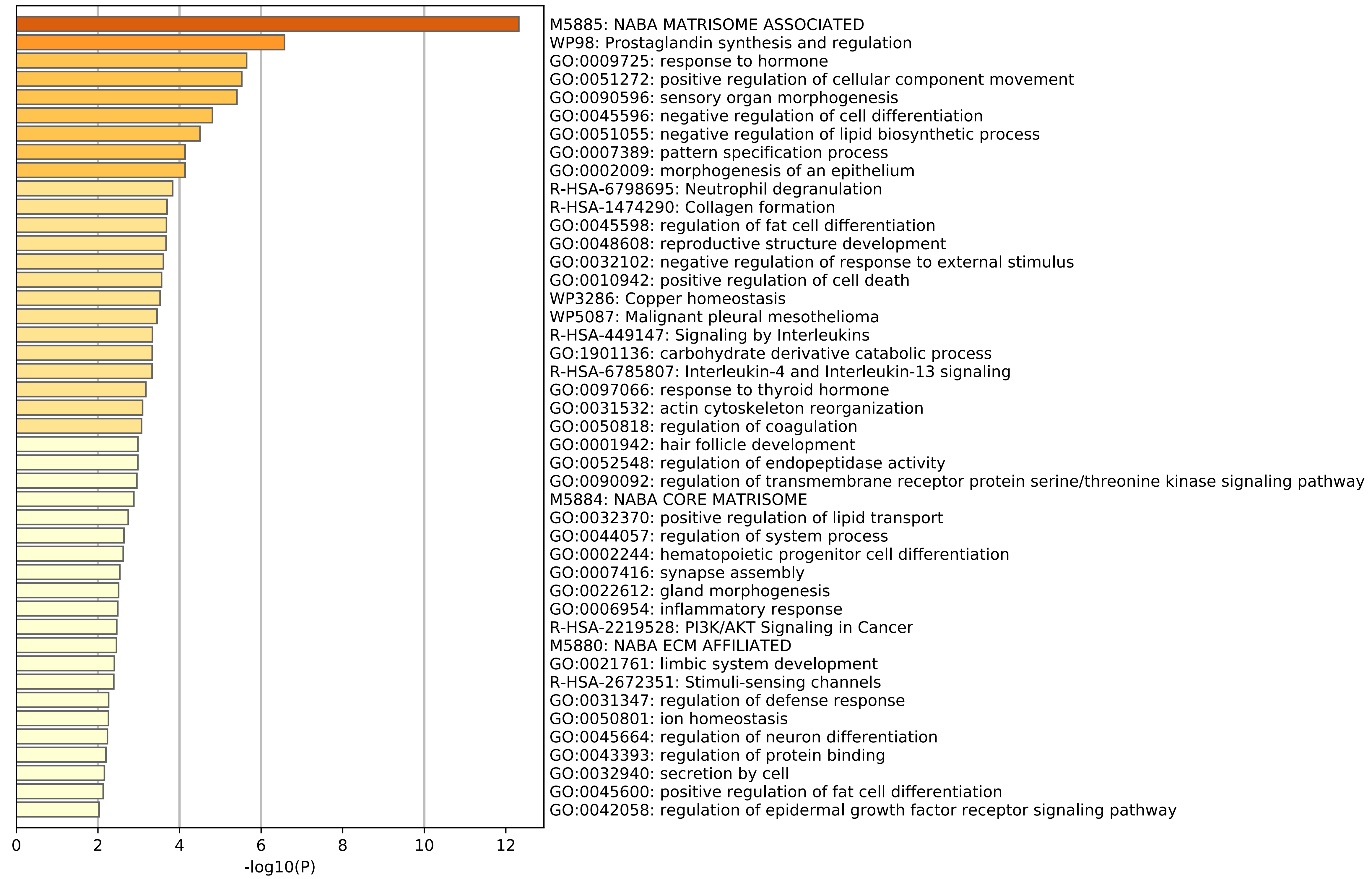

M5885: NABA MATRISOME ASSOCIATED
WP98: Prostaglandin synthesis and regulation
GO:0009725: response to hormone
GO:0051272: positive regulation of cellular component movement
GO:0090596: sensory organ morphogenesis
GO:0045596: negative regulation of cell differentiation
GO:0051055: negative regulation of lipid biosynthetic process
GO:0007389: pattern specification process
GO:0002009: morphogenesis of an epithelium
R-HSA-6798695: Neutrophil degranulation
R-HSA-1474290: Collagen formation
GO:0045598: regulation of fat cell differentiation
GO:0048608: reproductive structure development
GO:0032102: negative regulation of response to external stimulus
GO:0010942: positive regulation of cell death
WP3286: Copper homeostasis
WP5087: Malignant pleural mesothelioma
R-HSA-449147: Signaling by Interleukins
GO:1901136: carbohydrate derivative catabolic process
R-HSA-6785807: Interleukin-4 and Interleukin-13 signaling
GO:0097066: response to thyroid hormone
GO:0031532: actin cytoskeleton reorganization
GO:0050818: regulation of coagulation
GO:0001942: hair follicle development
GO:0052548: regulation of endopeptidase activity
GO:0090092: regulation of transmembrane receptor protein serine/threonine kinase signaling pathway
M5884: NABA CORE MATRISOME
GO:0032370: positive regulation of lipid transport
GO:0044057: regulation of system process
GO:0002244: hematopoietic progenitor cell differentiation
GO:0007416: synapse assembly
GO:0022612: gland morphogenesis
GO:0006954: inflammatory response
R-HSA-2219528: PI3K/AKT Signaling in Cancer
M5880: NABA ECM AFFILIATED
GO:0021761: limbic system development
R-HSA-2672351: Stimuli-sensing channels
GO:0031347: regulation of defense response
GO:0050801: ion homeostasis
GO:0045664: regulation of neuron differentiation
GO:0043393: regulation of protein binding
GO:0032940: secretion by cell
GO:0045600: positive regulation of fat cell differentiation
GO:0042058: regulation of epidermal growth factor receptor signaling pathway
0
2
4
6
8
10
12
-log10(P)
