# Supplementary material for: Transcriptional profiling of Hutchinson-Gilford Progeria syndrome fibroblasts reveals deficits in mesenchymal stem cell commitment to differentiation related to early events in endochondral ossification
Source: eLife. 2022 Dec 29;11:e81290. doi: 10.7554/eLife.81290 (PMC9833827; doi:10.7554/eLife.81290)
Supplement: Table 2—source data 1. — Early and late childhood patient samples compared to healthy children, middle aged, and older adults. [file elife-81290-table2-data1.zip › Table_2-Source_Data_1/4-7-M-UP-173.zip!/Enrichment_GO/ColorByCluster.pdf]

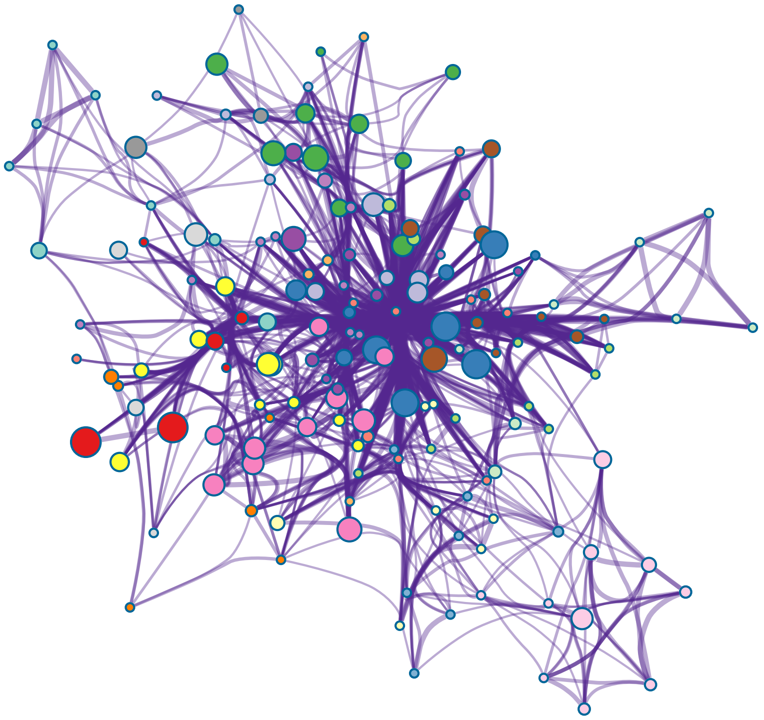

brain development
skeletal system development
Malignant pleural mesothelioma
Pathways in cancer
Amyotrophic lateral sclerosis (ALS)
regulation of cell development
tube morphogenesis
neuron projection morphogenesis
response to inorganic substance
regulation of neuron death
establishment or maintenance of cell polarity
enzyme linked receptor protein signaling pathway
membranous septum morphogenesis
Dilated cardiomyopathy
Hippo signaling regulation pathways
regulation of stem cell differentiation
positive regulation of protein localization
response to cytokine
cellular response to external stimulus
skin development

created by
http://metascape.org
