# Supplementary material for: Transcriptional profiling of Hutchinson-Gilford Progeria syndrome fibroblasts reveals deficits in mesenchymal stem cell commitment to differentiation related to early events in endochondral ossification
Source: eLife. 2022 Dec 29;11:e81290. doi: 10.7554/eLife.81290 (PMC9833827; doi:10.7554/eLife.81290)
Supplement: Table 2—source data 1. — Early and late childhood patient samples compared to healthy children, middle aged, and older adults. [file elife-81290-table2-data1.zip › Table_2-Source_Data_1/4-7-M-UP-173.zip!/Enrichment_heatmap/HeatmapSelectedGO.pdf]

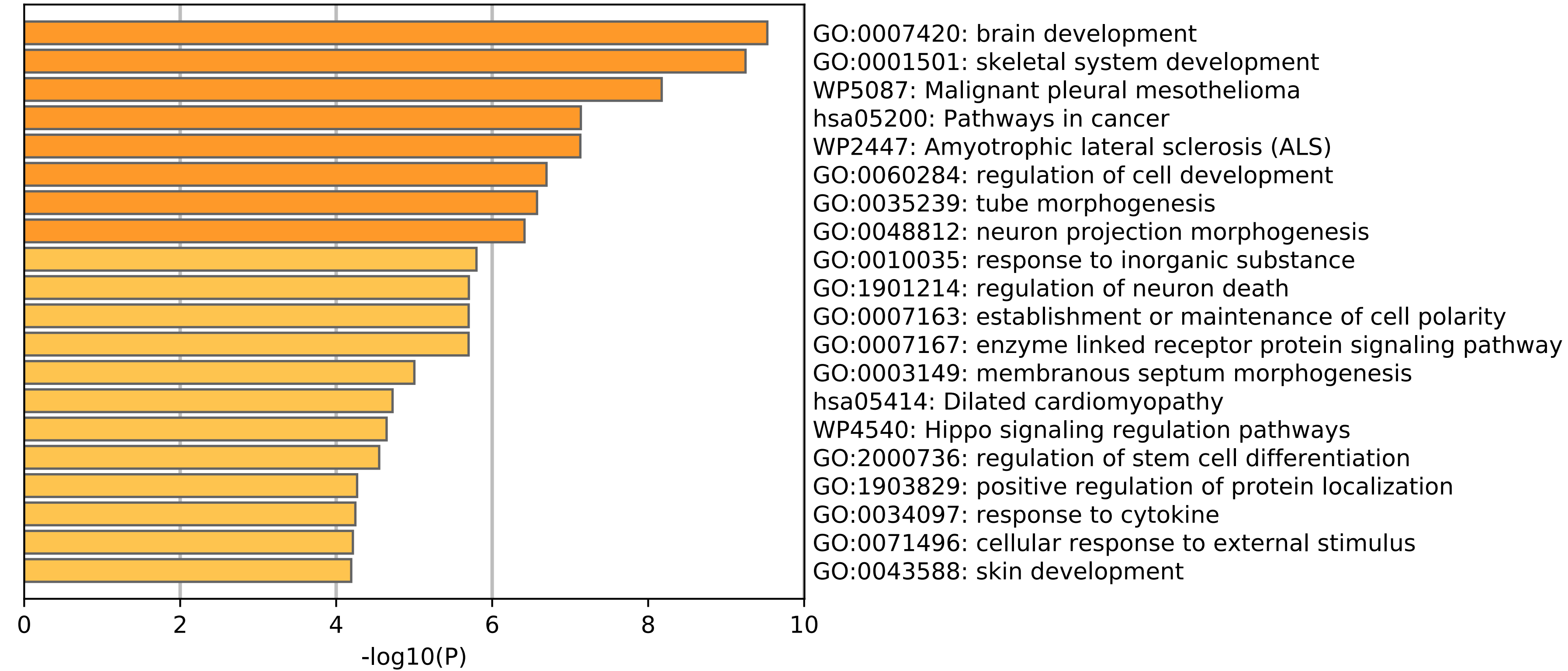
GO:0007420: brain development
GO:0001501: skeletal system development
WP5087: Malignant pleural mesothelioma
hsa05200: Pathways in cancer
WP2447: Amyotrophic lateral sclerosis (ALS)
GO:0060284: regulation of cell development
GO:0035239: tube morphogenesis
GO:0048812: neuron projection morphogenesis
GO:0010035: response to inorganic substance
GO:1901214: regulation of neuron death
GO:0007163: establishment or maintenance of cell polarity
GO:0007167: enzyme linked receptor protein signaling pathway
GO:0003149: membranous septum morphogenesis
hsa05414: Dilated cardiomyopathy
WP4540: Hippo signaling regulation pathways
GO:2000736: regulation of stem cell differentiation
GO:1903829: positive regulation of protein localization
GO:0034097: response to cytokine
GO:0071496: cellular response to external stimulus
GO:0043588: skin development
0
2
4
6
8
10
-log10(P)
