# Supplementary material for: Transcriptional profiling of Hutchinson-Gilford Progeria syndrome fibroblasts reveals deficits in mesenchymal stem cell commitment to differentiation related to early events in endochondral ossification
Source: eLife. 2022 Dec 29;11:e81290. doi: 10.7554/eLife.81290 (PMC9833827; doi:10.7554/eLife.81290)
Supplement: Table 2—source data 1. — Early and late childhood patient samples compared to healthy children, middle aged, and older adults. [file elife-81290-table2-data1.zip › Table_2-Source_Data_1/4-7-M-UP-173.zip!/Enrichment_heatmap/HeatmapSelectedGOTop100.pdf]

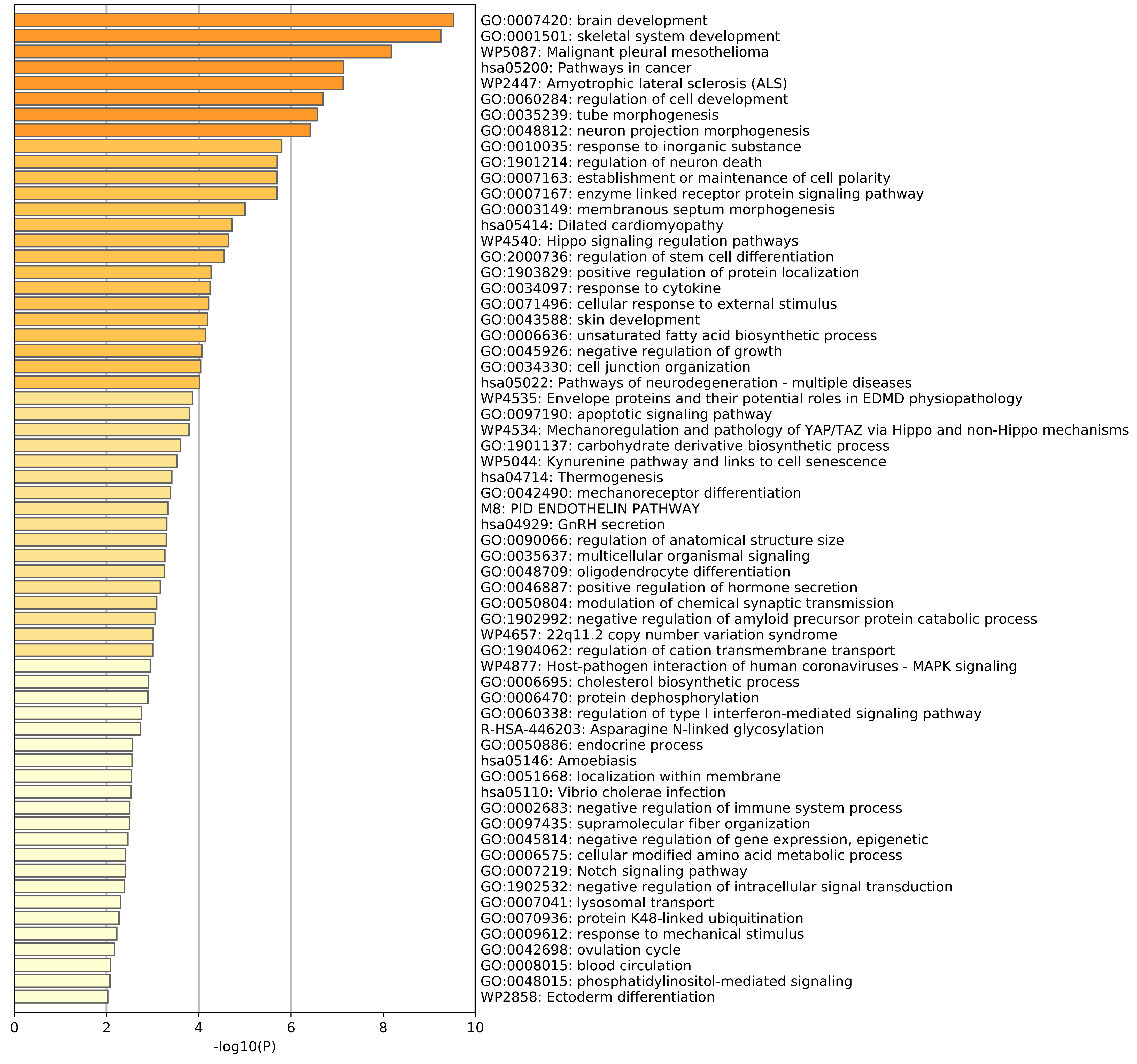
GO:0007420: brain development
GO:0001501: skeletal system development
WP5087: Malignant pleural mesothelioma
hsa05200: Pathways in cancer
WP2447: Amyotrophic lateral sclerosis (ALS)
GO:0060284: regulation of cell development
GO:0035239: tube morphogenesis
GO:0048812: neuron projection morphogenesis
GO:0010035: response to inorganic substance
GO:1901214: regulation of neuron death
GO:0007163: establishment or maintenance of cell polarity
GO:0007167: enzyme linked receptor protein signaling pathway
GO:0003149: membranous septum morphogenesis
hsa05414: Dilated cardiomyopathy
WP4540: Hippo signaling regulation pathways
GO:2000736: regulation of stem cell differentiation
GO:1903829: positive regulation of protein localization
GO:0034097: response to cytokine
GO:0071496: cellular response to external stimulus
GO:0043588: skin development
GO:0006636: unsaturated fatty acid biosynthetic process
GO:0045926: negative regulation of growth
GO:0034330: cell junction organization
hsa05022: Pathways of neurodegeneration - multiple diseases
WP4535: Envelope proteins and their potential roles in EDMD physiopathology
GO:0097190: apoptotic signaling pathway
WP4534: Mechanoregulation and pathology of YAP/TAZ via Hippo and non-Hippo mechanisms
GO:1901137: carbohydrate derivative biosynthetic process
WP5044: Kynurenine pathway and links to cell senescence
hsa04714: Thermogenesis
GO:0042490: mechanoreceptor differentiation
M8: PID ENDOTHELIN PATHWAY
hsa04929: GnRH secretion
GO:0090066: regulation of anatomical structure size
GO:0035637: multicellular organismal signaling
GO:0048709: oligodendrocyte differentiation
GO:0046887: positive regulation of hormone secretion
GO:0050804: modulation of chemical synaptic transmission
GO:1902992: negative regulation of amyloid precursor protein catabolic process
WP4657: 22q11.2 copy number variation syndrome
GO:1904062: regulation of cation transmembrane transport
WP4877: Host-pathogen interaction of human coronaviruses - MAPK signaling
GO:0006695: cholesterol biosynthetic process
GO:0006470: protein dephosphorylation
GO:0060338: regulation of type I interferon-mediated signaling pathway
R-HSA-446203: Asparagine N-linked glycosylation
GO:0050886: endocrine process
hsa05146: Amoebiasis
GO:0051668: localization within membrane
hsa05110: Vibrio cholerae infection
GO:0002683: negative regulation of immune system process
GO:0097435: supramolecular fiber organization
GO:0045814: negative regulation of gene expression, epigenetic
GO:0006575: cellular modified amino acid metabolic process
GO:0007219: Notch signaling pathway
GO:1902532: negative regulation of intracellular signal transduction
GO:0007041: lysosomal transport
GO:0070936: protein K48-linked ubiquitination
GO:0009612: response to mechanical stimulus
GO:0042698: ovulation cycle
GO:0008015: blood circulation
GO:0048015: phosphatidylinositol-mediated signaling
WP2858: Ectoderm differentiation
0
2
4
6
8
10
-log10(P)
