# Supplementary material for: Transcriptional profiling of Hutchinson-Gilford Progeria syndrome fibroblasts reveals deficits in mesenchymal stem cell commitment to differentiation related to early events in endochondral ossification
Source: eLife. 2022 Dec 29;11:e81290. doi: 10.7554/eLife.81290 (PMC9833827; doi:10.7554/eLife.81290)
Supplement: Table 2—source data 1. — Early and late childhood patient samples compared to healthy children, middle aged, and older adults. [file elife-81290-table2-data1.zip › Table_2-Source_Data_1/4-7-O_UP_305.zip!/Enrichment_GO/ColorByCluster.pdf]

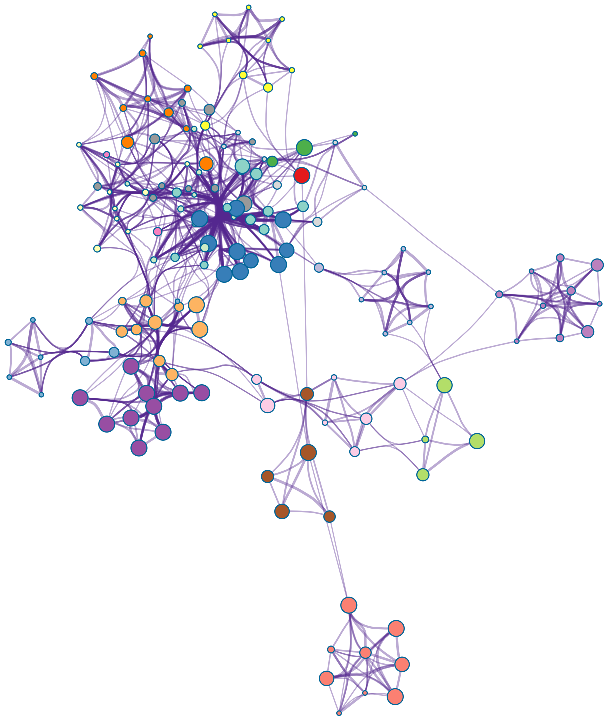

Malignant pleural mesothelioma
tissue morphogenesis
muscle structure development
cellular component morphogenesis
skin development
Hippo-Merlin signaling dysregulation
enzyme linked receptor protein signaling pathway
Neural crest differentiation
Pathways in cancer
cardiac chamber development
regulation of morphogenesis of an epithelium
neurofilament bundle assembly
regulation of kinase activity
glial cell differentiation
telencephalon development
supramolecular fiber organization
regulation of anatomical structure size
ameboidal-type cell migration
multicellular organismal signaling
neural tube development

created by
http://metascape.org
