# Supplementary material for: Transcriptional profiling of Hutchinson-Gilford Progeria syndrome fibroblasts reveals deficits in mesenchymal stem cell commitment to differentiation related to early events in endochondral ossification
Source: eLife. 2022 Dec 29;11:e81290. doi: 10.7554/eLife.81290 (PMC9833827; doi:10.7554/eLife.81290)
Supplement: Table 2—source data 1. — Early and late childhood patient samples compared to healthy children, middle aged, and older adults. [file elife-81290-table2-data1.zip › Table_2-Source_Data_1/4-7-O_UP_305.zip!/Enrichment_GO/ColorByPValue.pdf]

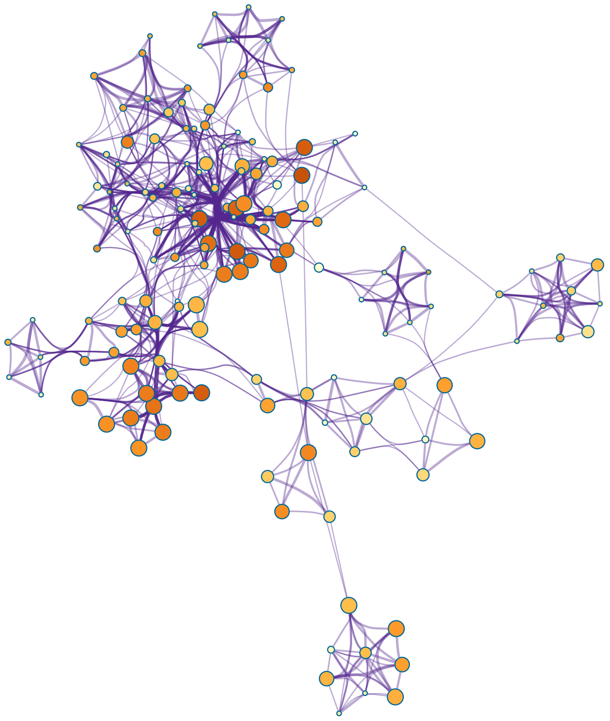

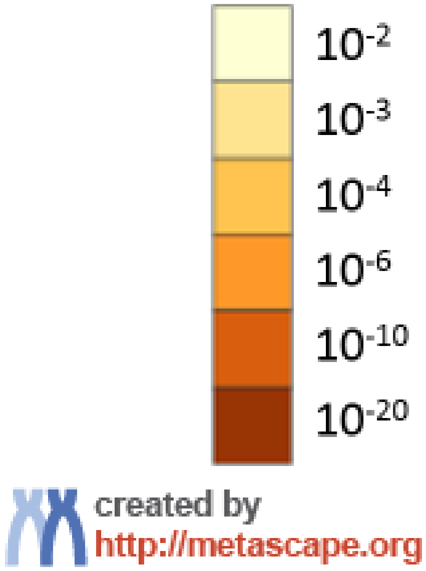

10-2
10-3
10-4
10-6
10-10
10-20
created by
http://metascape.org
