# Supplementary material for: Transcriptional profiling of Hutchinson-Gilford Progeria syndrome fibroblasts reveals deficits in mesenchymal stem cell commitment to differentiation related to early events in endochondral ossification
Source: eLife. 2022 Dec 29;11:e81290. doi: 10.7554/eLife.81290 (PMC9833827; doi:10.7554/eLife.81290)
Supplement: Table 2—source data 1. — Early and late childhood patient samples compared to healthy children, middle aged, and older adults. [file elife-81290-table2-data1.zip › Table_2-Source_Data_1/4-7-O_UP_305.zip!/Enrichment_heatmap/HeatmapSelectedGO.pdf]

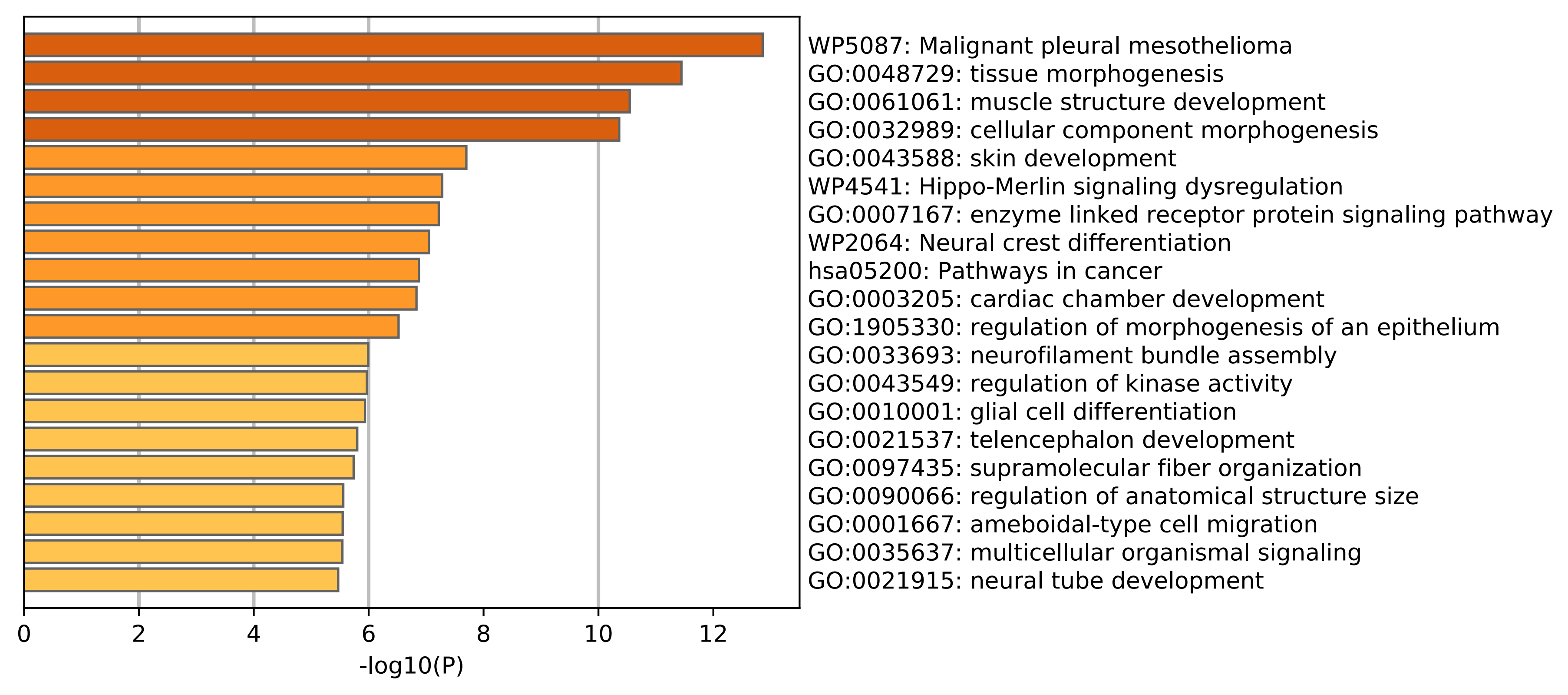
WP5087: Malignant pleural mesothelioma
GO:0048729: tissue morphogenesis
GO:0061061: muscle structure development
GO:0032989: cellular component morphogenesis
GO:0043588: skin development
WP4541: Hippo-Merlin signaling dysregulation
GO:0007167: enzyme linked receptor protein signaling pathway
WP2064: Neural crest differentiation
hsa05200: Pathways in cancer
GO:0003205: cardiac chamber development
GO:1905330: regulation of morphogenesis of an epithelium
GO:0033693: neurofilament bundle assembly
GO:0043549: regulation of kinase activity
GO:0010001: glial cell differentiation
GO:0021537: telencephalon development
GO:0097435: supramolecular fiber organization
GO:0090066: regulation of anatomical structure size
GO:0001667: ameboidal-type cell migration
GO:0035637: multicellular organismal signaling
GO:0021915: neural tube development
0
2
4
6
8
10
12
-log10(P)
