# Supplementary material for: Transcriptional profiling of Hutchinson-Gilford Progeria syndrome fibroblasts reveals deficits in mesenchymal stem cell commitment to differentiation related to early events in endochondral ossification
Source: eLife. 2022 Dec 29;11:e81290. doi: 10.7554/eLife.81290 (PMC9833827; doi:10.7554/eLife.81290)
Supplement: Table 2—source data 1. — Early and late childhood patient samples compared to healthy children, middle aged, and older adults. [file elife-81290-table2-data1.zip › Table_2-Source_Data_1/4-7-O_UP_305.zip!/Enrichment_heatmap/HeatmapSelectedGOTop100.pdf]

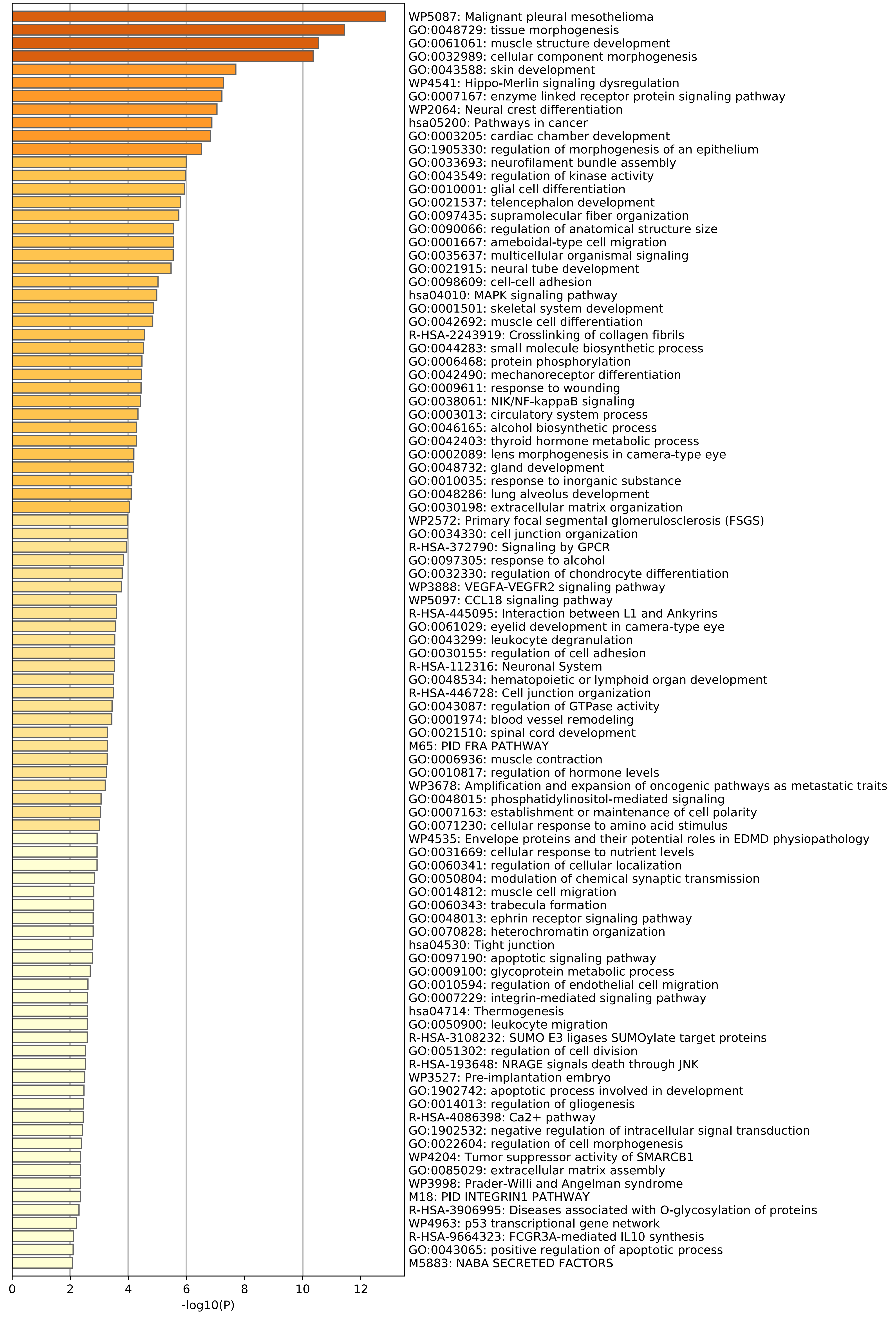
WP5087: Malignant pleural mesothelioma
GO:0048729: tissue morphogenesis
GO:0061061: muscle structure development
GO:0032989: cellular component morphogenesis
GO:0043588: skin development
WP4541: Hippo-Merlin signaling dysregulation
GO:0007167: enzyme linked receptor protein signaling pathway
WP2064: Neural crest differentiation
hsa05200: Pathways in cancer
GO:0003205: cardiac chamber development
GO:1905330: regulation of morphogenesis of an epithelium
GO:0033693: neurofilament bundle assembly
GO:0043549: regulation of kinase activity
GO:0010001: glial cell differentiation
GO:0021537: telencephalon development
GO:0097435: supramolecular fiber organization
GO:0090066: regulation of anatomical structure size
GO:0001667: ameboidal-type cell migration
GO:0035637: multicellular organismal signaling
GO:0021915: neural tube development
GO:0098609: cell-cell adhesion
hsa04010: MAPK signaling pathway
GO:0001501: skeletal system development
GO:0042692: muscle cell differentiation
R-HSA-2243919: Crosslinking of collagen fibrils
GO:0044283: small molecule biosynthetic process
GO:0006468: protein phosphorylation
GO:0042490: mechanoreceptor differentiation
GO:0009611: response to wounding
GO:0038061: NIK/NF-kappaB signaling
GO:0003013: circulatory system process
GO:0046165: alcohol biosynthetic process
GO:0042403: thyroid hormone metabolic process
GO:0002089: lens morphogenesis in camera-type eye
GO:0048732: gland development
GO:0010035: response to inorganic substance
GO:0048286: lung alveolus development
GO:0030198: extracellular matrix organization
WP2572: Primary focal segmental glomerulosclerosis (FSGS)
GO:0034330: cell junction organization
R-HSA-372790: Signaling by GPCR
GO:0097305: response to alcohol
GO:0032330: regulation of chondrocyte differentiation
WP3888: VEGFA-VEGFR2 signaling pathway
WP5097: CCL18 signaling pathway
R-HSA-445095: Interaction between L1 and Ankyrins
GO:0061029: eyelid development in camera-type eye
GO:0043299: leukocyte degranulation
GO:0030155: regulation of cell adhesion
R-HSA-112316: Neuronal System
GO:0048534: hematopoietic or lymphoid organ development
R-HSA-446728: Cell junction organization
GO:0043087: regulation of GTPase activity
GO:0001974: blood vessel remodeling
GO:0021510: spinal cord development
M65: PID FRA PATHWAY
GO:0006936: muscle contraction
GO:0010817: regulation of hormone levels
WP3678: Amplification and expansion of oncogenic pathways as metastatic traits
GO:0048015: phosphatidylinositol-mediated signaling
GO:0007163: establishment or maintenance of cell polarity
GO:0071230: cellular response to amino acid stimulus
WP4535: Envelope proteins and their potential roles in EDMD physiopathology
GO:0031669: cellular response to nutrient levels
GO:0060341: regulation of cellular localization
GO:0050804: modulation of chemical synaptic transmission
GO:0014812: muscle cell migration
GO:0060343: trabecula formation
GO:0048013: ephrin receptor signaling pathway
GO:0070828: heterochromatin organization
hsa04530: Tight junction
GO:0097190: apoptotic signaling pathway
GO:0009100: glycoprotein metabolic process
GO:0010594: regulation of endothelial cell migration
GO:0007229: integrin-mediated signaling pathway
hsa04714: Thermogenesis
GO:0050900: leukocyte migration
R-HSA-3108232: SUMO E3 ligases SUMOylate target proteins
GO:0051302: regulation of cell division
R-HSA-193648: NRAGE signals death through JNK
WP3527: Pre-implantation embryo
GO:1902742: apoptotic process involved in development
GO:0014013: regulation of gliogenesis
R-HSA-4086398: Ca2+ pathway
GO:1902532: negative regulation of intracellular signal transduction
GO:0022604: regulation of cell morphogenesis
WP4204: Tumor suppressor activity of SMARCB1
GO:0085029: extracellular matrix assembly
WP3998: Prader-Willi and Angelman syndrome
M18: PID INTEGRIN1 PATHWAY
R-HSA-3906995: Diseases associated with O-glycosylation of proteins
WP4963: p53 transcriptional gene network
R-HSA-9664323: FCGR3A-mediated IL10 synthesis
GO:0043065: positive regulation of apoptotic process
M5883: NABA SECRETED FACTORS
0
2
4
6
8
10
12
-log10(P)
