# Supplementary material for: Transcriptional profiling of Hutchinson-Gilford Progeria syndrome fibroblasts reveals deficits in mesenchymal stem cell commitment to differentiation related to early events in endochondral ossification
Source: eLife. 2022 Dec 29;11:e81290. doi: 10.7554/eLife.81290 (PMC9833827; doi:10.7554/eLife.81290)
Supplement: Table 2—source data 1. — Early and late childhood patient samples compared to healthy children, middle aged, and older adults. [file elife-81290-table2-data1.zip › Table_2-Source_Data_1/4-7-O-DOWN_394.zip!/Enrichment_GO/ColorByCluster.pdf]

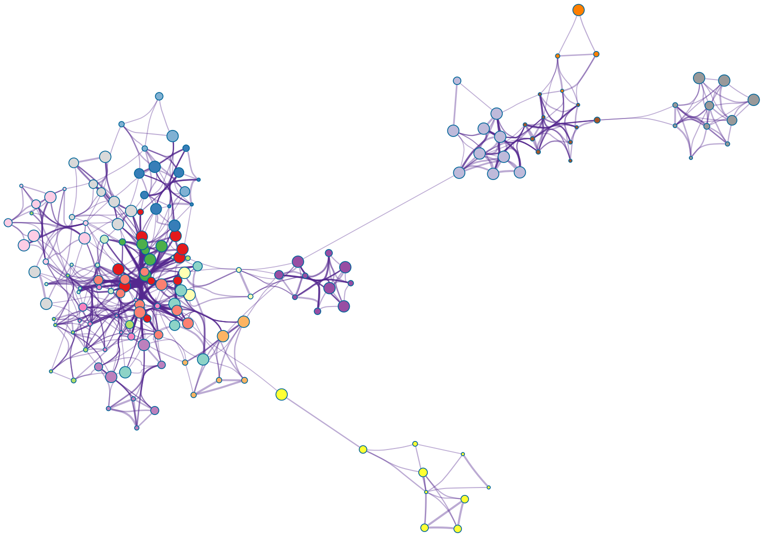

- positive regulation of protein phosphorylation
- regulation of cell adhesion
- positive regulation of locomotion
- cellular response to lipid
- Transport of small molecules
- NABA MATRISOME ASSOCIATED
- Ferroptosis
- EGF/EGFR signaling pathway
- organic hydroxy compound metabolic process
- vasculature development
- response to growth factor
- ion homeostasis
- MAPK signaling pathway
- negative regulation of cell adhesion
- reproductive structure development
- Rap1 signaling pathway
- Signaling by Interleukins
- chemotaxis
- response to wounding
- regulation of leukocyte migration

created by
http://metascape.org
