# Supplementary material for: Transcriptional profiling of Hutchinson-Gilford Progeria syndrome fibroblasts reveals deficits in mesenchymal stem cell commitment to differentiation related to early events in endochondral ossification
Source: eLife. 2022 Dec 29;11:e81290. doi: 10.7554/eLife.81290 (PMC9833827; doi:10.7554/eLife.81290)
Supplement: Table 2—source data 1. — Early and late childhood patient samples compared to healthy children, middle aged, and older adults. [file elife-81290-table2-data1.zip › Table_2-Source_Data_1/4-7-O-DOWN_394.zip!/Enrichment_heatmap/HeatmapSelectedGO.pdf]

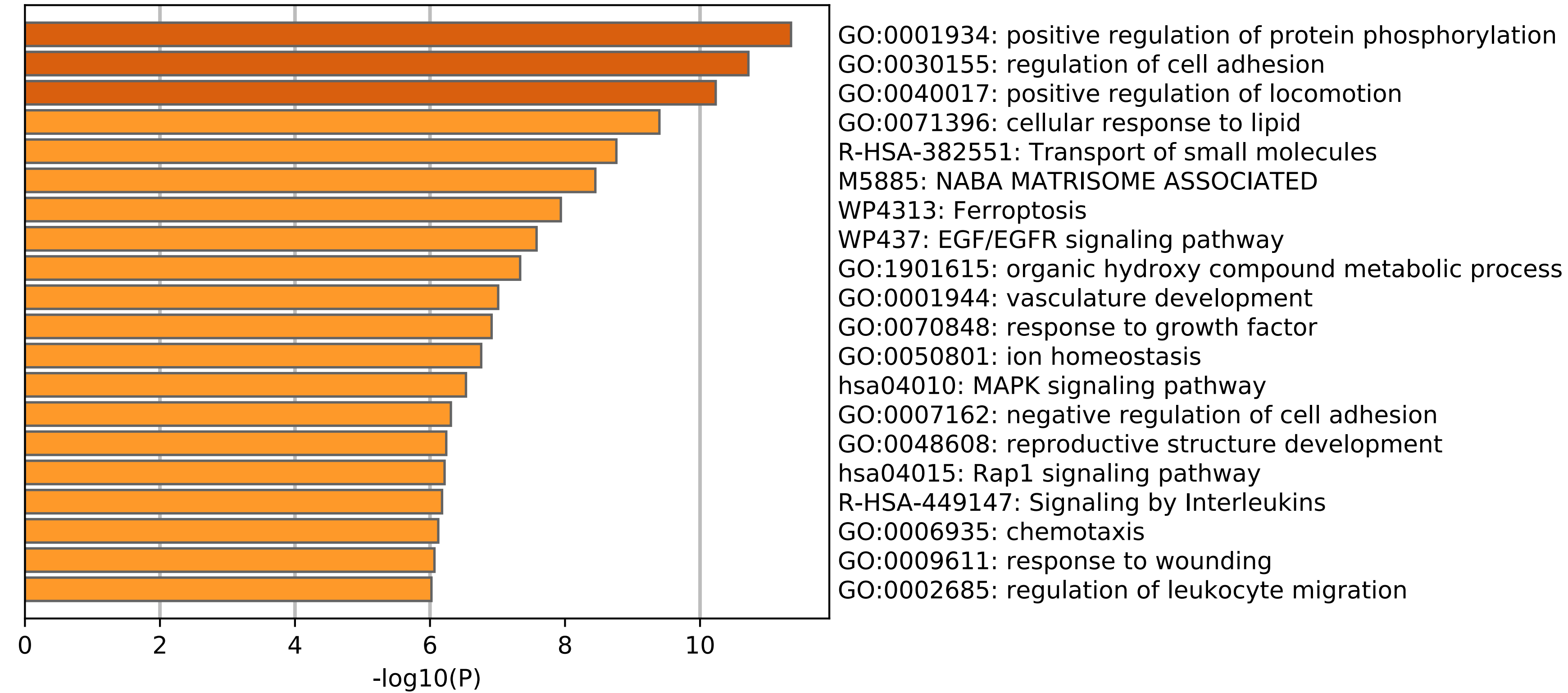

GO:0001934: positive regulation of protein phosphorylation
GO:0030155: regulation of cell adhesion
GO:0040017: positive regulation of locomotion
GO:0071396: cellular response to lipid
R-HSA-382551: Transport of small molecules
M5885: NABA MATRISOME ASSOCIATED
WP4313: Ferroptosis
WP437: EGF/EGFR signaling pathway
GO:1901615: organic hydroxy compound metabolic process
GO:0001944: vasculature development
GO:0070848: response to growth factor
GO:0050801: ion homeostasis
hsa04010: MAPK signaling pathway
GO:0007162: negative regulation of cell adhesion
GO:0048608: reproductive structure development
hsa04015: Rap1 signaling pathway
R-HSA-449147: Signaling by Interleukins
GO:0006935: chemotaxis
GO:0009611: response to wounding
GO:0002685: regulation of leukocyte migration
0
2
4
6
8
10
-log10(P)
