# Supplementary material for: Transcriptional profiling of Hutchinson-Gilford Progeria syndrome fibroblasts reveals deficits in mesenchymal stem cell commitment to differentiation related to early events in endochondral ossification
Source: eLife. 2022 Dec 29;11:e81290. doi: 10.7554/eLife.81290 (PMC9833827; doi:10.7554/eLife.81290)
Supplement: Table 2—source data 1. — Early and late childhood patient samples compared to healthy children, middle aged, and older adults. [file elife-81290-table2-data1.zip › Table_2-Source_Data_1/4-7-O-DOWN_394.zip!/Enrichment_heatmap/HeatmapSelectedGOTop100.pdf]

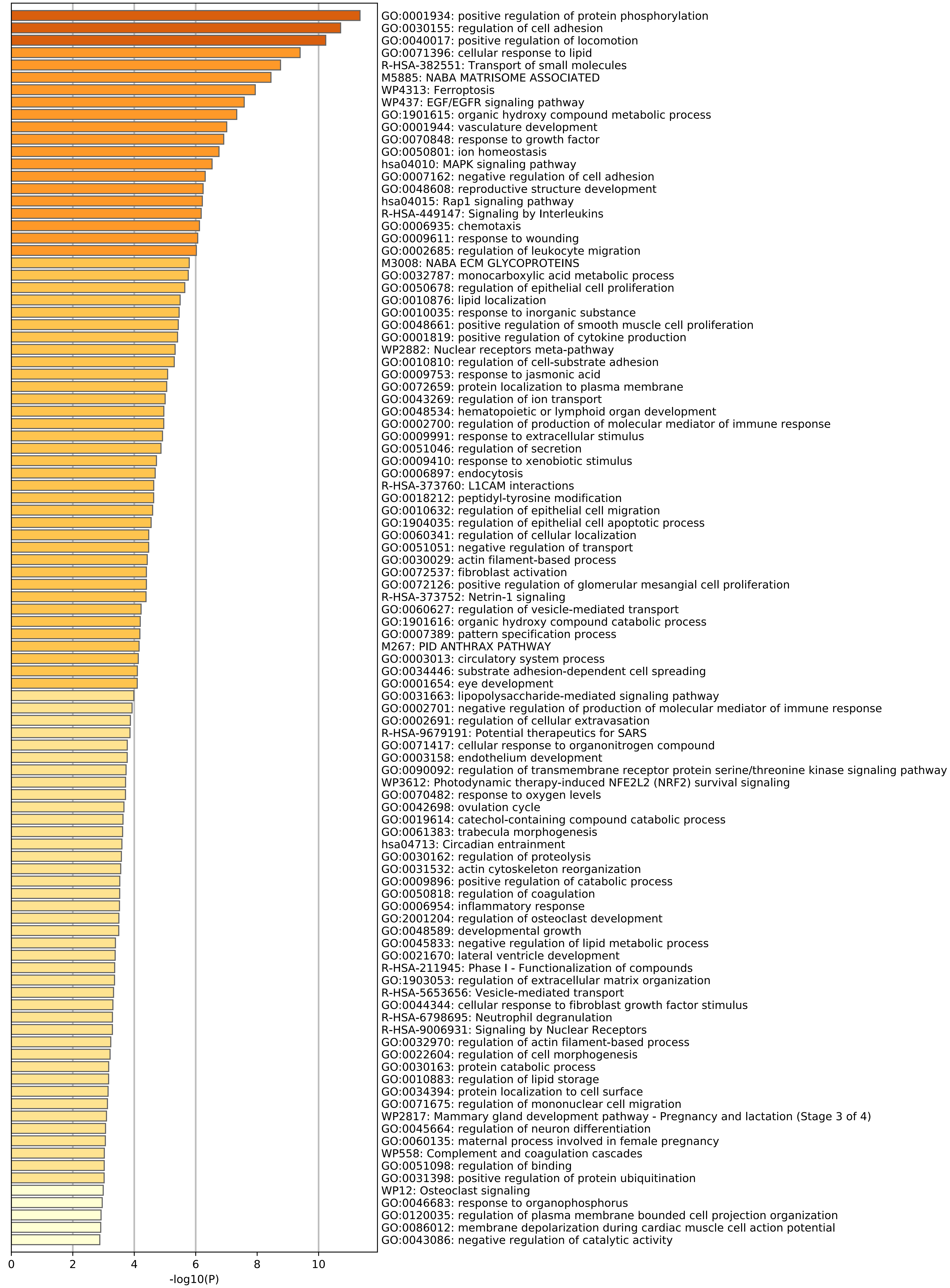
GO:0001934: positive regulation of protein phosphorylation
GO:0030155: regulation of cell adhesion
GO:0040017: positive regulation of locomotion
GO:0071396: cellular response to lipid
R-HSA-382551: Transport of small molecules
M5885: NABA MATRISOME ASSOCIATED
WP4313: Ferroptosis
WP437: EGF/EGFR signaling pathway
GO:1901615: organic hydroxy compound metabolic process
GO:0001944: vasculature development
GO:0070848: response to growth factor
GO:0050801: ion homeostasis
hsa04010: MAPK signaling pathway
GO:0007162: negative regulation of cell adhesion
GO:0048608: reproductive structure development
hsa04015: Rap1 signaling pathway
R-HSA-449147: Signaling by Interleukins
GO:0006935: chemotaxis
GO:0009611: response to wounding
GO:0002685: regulation of leukocyte migration
M3008: NABA ECM GLYCOPROTEINS
GO:0032787: monocarboxylic acid metabolic process
GO:0050678: regulation of epithelial cell proliferation
GO:0010876: lipid localization
GO:0010035: response to inorganic substance
GO:0048661: positive regulation of smooth muscle cell proliferation
GO:0001819: positive regulation of cytokine production
WP2882: Nuclear receptors meta-pathway
GO:0010810: regulation of cell-substrate adhesion
GO:0009753: response to jasmonic acid
GO:0072659: protein localization to plasma membrane
GO:0043269: regulation of ion transport
GO:0048534: hematopoietic or lymphoid organ development
GO:0002700: regulation of production of molecular mediator of immune response
GO:0009991: response to extracellular stimulus
GO:0051046: regulation of secretion
GO:0009410: response to xenobiotic stimulus
GO:0006897: endocytosis
R-HSA-373760: L1CAM interactions
GO:0018212: peptidyl-tyrosine modification
GO:0010632: regulation of epithelial cell migration
GO:1904035: regulation of epithelial cell apoptotic process
GO:0060341: regulation of cellular localization
GO:0051051: negative regulation of transport
GO:0030029: actin filament-based process
GO:0072537: fibroblast activation
GO:0072126: positive regulation of glomerular mesangial cell proliferation
R-HSA-373752: Netrin-1 signaling
GO:0060627: regulation of vesicle-mediated transport
GO:1901616: organic hydroxy compound catabolic process
GO:0007389: pattern specification process
M267: PID ANTHRAX PATHWAY
GO:0003013: circulatory system process
GO:0034446: substrate adhesion-dependent cell spreading
GO:0001654: eye development
GO:0031663: lipopolysaccharide-mediated signaling pathway
GO:0002701: negative regulation of production of molecular mediator of immune response
GO:0002691: regulation of cellular extravasation
R-HSA-9679191: Potential therapeutics for SARS
GO:0071417: cellular response to organonitrogen compound
GO:0003158: endothelium development
GO:0090092: regulation of transmembrane receptor protein serine/threonine kinase signaling pathway
WP3612: Photodynamic therapy-induced NFE2L2 (NRF2) survival signaling
GO:0070482: response to oxygen levels
GO:0042698: ovulation cycle
GO:0019614: catechol-containing compound catabolic process
GO:0061383: trabecula morphogenesis
hsa04713: Circadian entrainment
GO:0030162: regulation of proteolysis
GO:0031532: actin cytoskeleton reorganization
GO:0009896: positive regulation of catabolic process
GO:0050818: regulation of coagulation
GO:0006954: inflammatory response
GO:2001204: regulation of osteoclast development
GO:0048589: developmental growth
GO:0045833: negative regulation of lipid metabolic process
GO:0021670: lateral ventricle development
R-HSA-211945: Phase I - Functionalization of compounds
GO:1903053: regulation of extracellular matrix organization
R-HSA-5653656: Vesicle-mediated transport
GO:0044344: cellular response to fibroblast growth factor stimulus
R-HSA-6798695: Neutrophil degranulation
R-HSA-9006931: Signaling by Nuclear Receptors
GO:0032970: regulation of actin filament-based process
GO:0022604: regulation of cell morphogenesis
GO:0030163: protein catabolic process
GO:0010883: regulation of lipid storage
GO:0034394: protein localization to cell surface
GO:0071675: regulation of mononuclear cell migration
WP2817: Mammary gland development pathway - Pregnancy and lactation (Stage 3 of 4)
GO:0045664: regulation of neuron differentiation
GO:0060135: maternal process involved in female pregnancy
WP558: Complement and coagulation cascades
GO:0051098: regulation of binding
GO:0031398: positive regulation of protein ubiquitination
WP12: Osteoclast signaling
GO:0046683: response to organophosphorus
GO:0120035: regulation of plasma membrane bounded cell projection organization
GO:0086012: membrane depolarization during cardiac muscle cell action potential
GO:0043086: negative regulation of catalytic activity
0
2
4
6
8
10
-log10(P)
